# AI in Point-of-Care Imaging for Clinical Decision Support: Systematic Review of Diagnostic Accuracy, Task-Shifting, and Explainability

**DOI:** 10.2196/80928

**Published:** 2026-04-27

**Authors:** Peter Wadie, Bishoy Zakher, Khalid Elgazzar, Abdulhamid Alsbakhi, Abdul-Mohsen G Alhejaily

**Affiliations:** 1 Department of Electrical, Computer, and Software Engineering, Faculty of Engineering and Applied Science, Ontario Tech University, 2000 Simcoe Street North, Oshawa, ON, L1G 0C5, Canada, 1 9059246707; 2 Department of Biomedical Engineering, Faculty of Engineering, University of Alberta, Edmonton, AB, Canada; 3 Canadian University Dubai, School of Engineering, Applied Science and Technology, Dubai, United Arab Emirates; 4 Riyadh Second Health Cluster, Riyadh, Saudi Arabia

**Keywords:** systematic reviews as topic, machine learning, diagnostic imaging, explainability, task shifting, artificial intelligence, AI, explainable AI, XAI, clinical decision support systems, CDSS, point-of-care systems, POC, mobile phone

## Abstract

**Background:**

Artificial intelligence (AI) integrated with point-of-care imaging is a promising approach to expand access in settings with limited specialist availability. However, no systematic review has comprehensively evaluated AI-assisted clinical decision support across multiple point-of-care imaging modalities, assessed explainability implementation, or quantified clinical impact evidence gaps.

**Objective:**

We aim to systematically evaluate and synthesize evidence on AI-based clinical decision support systems using point-of-care imaging.

**Methods:**

We searched PubMed, Scopus, IEEE Xplore, and Web of Science (January 2018 to November 2025). We included research studies evaluating AI or machine learning systems applied to point-of-care–capable imaging modalities in clinical settings with clinical decision support outputs. Two reviewers independently screened studies, extracted data across 15 domains, and assessed methodological quality using QUADAS-2 (Quality Assessment of Diagnostic Accuracy Studies 2). Proposed frameworks were developed to evaluate explainability implementation and clinical impact evidence. Narrative synthesis was performed due to substantial data heterogeneity.

**Results:**

Of 2113 records identified, 20 studies met inclusion criteria, encompassing approximately 78,000 patients across 15 countries. Studies evaluated tuberculosis (n=5), breast cancer (n=3), deep vein thrombosis (DVT) (n=2), and 9 other conditions using ultrasound (7/20, 35%), chest x-ray (5/20, 25%), photography-based and colposcopic imaging (3/20, 15%), fundus photography (2/20, 10%), microscopy (2/20, 10%), and dermoscopy (1/20, 5%). Median sensitivity was 93.6% (IQR 87%-98%), and median specificity was 90.6% (IQR 74.5%-96.7%). Task-shifting was demonstrated in 65% (13/20) of studies, with nonspecialists achieving specialist-level performance after a median of 1 hour of training (range 30 minutes to 6 months; n=6 studies reporting specific durations). The explainable artificial intelligence (XAI) implementation cascade revealed critical gaps: 75% (15/20) of studies did not mention explainability, 10% (2/20) provided explanations to users, and none evaluated whether clinicians understood explanations or whether XAI influenced decisions. The clinical impact pyramid showed 15% (3/20) of studies reported technical accuracy only, 65% (13/20) reported process outcomes, 20% (4/20) documented clinical actions, and none measured patient outcomes. Methodological quality was concerning, as 70% (14/20) of studies were at high or very high risk of bias, with verification bias (14/20, 70%) and selection bias (10/20, 50%) being the most common. The overall certainty of evidence was very low—GRADE (Grading of Recommendations, Assessment, Development, and Evaluation) ⊕◯◯◯, primarily due to risk of bias, heterogeneity, and imprecision.

**Conclusions:**

AI-assisted point-of-care imaging demonstrates promising diagnostic accuracy and enables meaningful task-shifting with minimal training requirements. However, critical evidence gaps remain, including absent patient outcome measurement, inadequate explainability evaluation, regulatory misalignment, and lack of cross-context validation despite claims of global applicability. Addressing these gaps requires implementation research with patient-outcome end points, rigorous XAI evaluation, and multicontext validation before widespread adoption. Limitations include restriction to English-language publications, gray literature exclusion, and heterogeneity precluding meta-analysis.

## Introduction

The intersection of artificial intelligence (AI), point-of-care imaging, and clinical decision support systems (CDSSs) represents a rapidly evolving field with significant potential to transform health care delivery, particularly in settings where specialist access is limited. Point-of-care imaging devices—including handheld ultrasound, smartphone-based dermoscopy, portable fundus cameras, and mobile x-ray systems—are proliferating worldwide; however, their diagnostic potential is often limited by the scarcity of trained specialists to interpret images. AI-based clinical decision support offers a potential solution by analyzing medical images in real time, providing automated diagnostic classifications, highlighting regions of interest, and generating confidence scores that enable nonspecialist health care workers to obtain accurate diagnostic interpretations at the point of care.

To contextualize this review within the existing evidence base, we searched for prior systematic reviews addressing similar questions by applying our PubMed search strategy with filters isolating systematic reviews and meta-analyses, yielding 4 relevant publications. Kossoff et al [1] reviewed automated lung ultrasound analysis for pneumothorax detection, which is limited to a single modality and condition, without requiring point-of-care validation. Cold et al [2] examined AI in bronchoscopy, focusing on specialist procedural settings rather than point-of-care contexts. Rambabu et al [3] evaluated AI for papilloedema detection using fundus photography, although the studies were predominantly retrospective and lacked clinical outcome assessment. Sunny et al [4] reviewed biomarker-integrated cytopathology for oral lesion detection; however, this laboratory-based methodology differs fundamentally from the deployment of point-of-care imaging. Critically, none evaluated task-shifting potential, explainable artificial intelligence (XAI) implementation frameworks, or clinical decision support integration—essential considerations for resource-limited settings where such technologies hold the most significant promise.

The rationale for this systematic review emerges from 3 converging factors. First, regulatory frameworks increasingly mandate explainability for high-risk AI systems. The European Union’s AI Act classifies medical diagnostic systems as high-risk applications requiring transparency and interpretability [5]. The US Food and Drug Administration’s (FDA’s) 2021 action plan for AI- or machine learning (ML)–based medical devices emphasizes the need for transparency regarding algorithmic decision-making logic [6]. Subsequent 2024 guidance explicitly defines “explainability” as the degree to which AI logic can be understood [7]. The 21st Century Cures Act specifies that clinical decision support tools must enable health care professionals to independently review the basis for recommendations to qualify for regulatory exemption [8,9]. This evolving regulatory landscape creates an urgent need for systematic evidence synthesis regarding whether XAI is being implemented in clinical practice and, critically, whether the explanations implemented are evaluated for their clinical utility. Second, the field is rapidly changing, with most relevant studies published since 2018 reflecting advances in deep learning architectures. Third, there is a critical need to characterize methodological quality, particularly in terms of external validation and clinical outcome assessment beyond diagnostic accuracy.

This review addresses identified gaps through five key differentiators: (1) multimodality scope that synthesizes evidence across all point-of-care-capable imaging modalities; (2) explicit CDSS framework assessment that examines how AI outputs integrate into clinical workflows; (3) systematic explainability (XAI) evaluation using a proposed cascade framework; (4) strict point-of-care setting requirement that excludes traditional radiology contexts; and (5) rigorous quality assessment using QUADAS-2 (Quality Assessment of Diagnostic Accuracy Studies 2) with attention to AI-specific bias concerns. These methodological features enable a comprehensive evaluation of both diagnostic performance and implementation readiness.

This systematic review aimed to evaluate AI-based CDSSs using point-of-care imaging for diagnostic purposes comprehensively. Using the PCC (Population, Concept, Context) framework, our primary objective was to systematically identify, evaluate, and synthesize evidence on these systems in human participants (population) across point-of-care clinical settings (context), with particular focus on diagnostic accuracy, clinical integration, and real-world implementation. Secondary objectives were to (1) characterize AI architectures, imaging modalities, and clinical domains; (2) assess reported diagnostic performance; (3) evaluate methodological quality using QUADAS-2; (4) examine the extent of XAI implementation and validation; and (5) identify evidence gaps to inform future research, practice, and policy.

## Methods

### Study Design

This systematic review was conducted following the PRISMA (Preferred Reporting Items for Systematic Reviews and Meta-Analyses) 2020 guidelines (Checklist 1). The review was not prospectively registered due to time constraints. A detailed protocol was developed a priori and is available from the corresponding author upon request.

### Eligibility Criteria

#### Overview

We used the PCC framework to define eligibility. The population comprised primary research studies (January 2018 to November 2025) evaluating AI or ML systems in human participants. The concept was AI-based clinical decision support using point-of-care imaging for diagnostic, classification, triage, or risk stratification purposes. The context included point-of-care clinical settings, such as emergency departments, intensive care units, primary care facilities, prehospital settings, low-resource environments, community health centers, mobile screening units, home care settings, and telemedicine applications.

#### Inclusion Criteria Required

Inclusion criteria required (1) original primary research or peer-reviewed full conference papers (greater than or equal to four pages with medical imaging focus); (2) full text being available in English; (3) publication year 2018‐2025; (4) evaluation of AI or ML systems (deep learning architectures or traditional ML methods); (5) point-of-care-capable imaging modalities (including ultrasound, dermoscopy, fundus photography, portable x-ray, smartphone-based imaging and portable microscopy); (6) actual deployment or validation in point-of-care settings (not aspirational point-of-care claims); (7) clinical decision support outputs (diagnosis, classification, triage, or screening—not purely technical image improvement); and (8) human participants research with clinical validation on patients.

#### Exclusion Criteria Applied

Exclusion criteria applied to (1) systematic reviews, meta-analyses, scoping reviews, literature reviews, editorials, commentaries, letters, book chapters, case reports with fewer than three patients, workshop papers, extended abstracts under four pages, poster abstracts, or preprints without subsequent peer-reviewed publication; (2) non-English language publications; (3) publications from 2017 or earlier; (4) the absence of AI or ML components, including studies with only statistical analysis, threshold-based algorithms, or pure image processing without ML; (5) non–point-of-care imaging modalities (computed tomography, magnetic resonance imaging, PET, SPECT, traditional mammography, fixed x-ray, or laboratory-based histopathology); (6) studies conducted in non–point-of-care settings such as radiology departments, pathology laboratories, or specialist referral centers without point-of-care validation; (7) purely technical outputs including image enhancement, denoising, super-resolution, reconstruction, registration, or segmentation without clinical interpretation; (8) animal-only studies, phantom-only studies, or purely synthetic or simulated data without human validation; and (9) full text being unavailable or inaccessible.

To address challenges in distinguishing genuine point-of-care implementation from aspirational claims, we applied supplementary screening criteria excluding (1) aspirational point-of-care mentions without actual deployment or validation, (2) public dataset studies without clinical validation, (3) algorithm development without patient-level testing, (4) retrospective analysis of archived images, (5) device development without point-of-care clinical testing, (6) unverified point-of-care workflows, (7) purely technical improvement studies, (8) ambiguous facility types, and (9) retrospective comparisons without prospective validation.

### Ethical Considerations

This systematic review analyzed published data without direct human interaction. Therefore, institutional review board approval was not required per standard guidelines.

### Information Sources

We searched 4 electronic databases on November 24, 2025: PubMed/MEDLINE (via the National Center for Biotechnology Information interface), Scopus (via the Elsevier platform), IEEE Xplore (via the IEEE platform), and Web of Science Core Collection (via the Clarivate platform). Gray literature, trial registers, and preprint servers were not searched. Neither backward citation searching nor forward citation searching was performed.

### Search Strategy

Search strategies combined 4 concept blocks using Boolean AND operators: (1) AI or ML technology terms (eg, “artificial intelligence,” “machine learning,” “deep learning,” “neural networks,” and “computer vision”); (2) clinical decision support terms (eg, “decision support,” “clinical decision support,” “CDSS,” “computer-aided detection,” and “diagnostic aids”); (3) medical imaging modality terms (eg, “ultrasound,” “dermoscopy,” “fundus imaging,” “microscopy,” “x-ray,” and “clinical photography”); and (4) point-of-care context terms (eg, “point-of-care,” “POCUS,” “bedside,” “handheld,” “portable,” “mobile health,” and “telemedicine”). No language restrictions were applied to the database searches, although only English-language reports were eligible for inclusion. Searches were limited to studies published from January 2018 onward to capture the recent era of deep learning applications in medical imaging. The search strategy was adapted to each database’s syntax requirements, with PubMed incorporating MeSH (Medical Subject Headings) terms combined with title or abstract keywords, Scopus using TITLE-ABS-KEY field tags, IEEE Xplore using explicit singular or plural forms, and Web of Science using topic search field tags. Complete search strategies for all databases, including exact queries and filters, are provided in Multimedia Appendix 1.

### Study Selection

Screening and selection followed a 2-stage process. Search results were imported into Zotero (Corporation for Digital Scholarship) for deduplication and reference management. A total of 2 reviewers independently screened titles and abstracts using Rayyan (Rayyan Systems Inc) software with anonymous dual review. Studies meeting the inclusion criteria or with uncertain eligibility proceeded to full-text review. A total of 2 reviewers independently assessed full texts, with disagreements resolved through discussion. No automation tools were used to support title or abstract screening or full-text assessment; human reviewers made all screening decisions. When multiple exclusion criteria were applied to a single study, the primary reason for exclusion was recorded following a prespecified hierarchy: first, study type, language, date, and availability issues; then, population characteristics; and finally, intervention criteria.

A representative sample of excluded studies, along with the reasons for exclusion, is provided in Multimedia Appendix 2.

### Data Collection Process

A total of 2 reviewers independently extracted data from each included study using a standardized form encompassing 18 prespecified sections: (1) study registry, (2) study design, (3) population, (4) point-of-care setting, (5) operator characteristics, (6) AI system, (7) AI output, (8) imaging, (9) reference standard, (10) diagnostic performance, (11) comparator, (12) explainability, (13) clinical outcomes, (14) limitations, (15) task-shifting, (16) integration, (17) QUADAS-2 summary, and (18) derived metrics (Multimedia Appendix 3). Disagreements were resolved through discussion. No automation tools were used in the data extraction process. Study authors were not contacted to obtain or confirm missing or unclear data. Unreported items were coded as “not reported,” and strict anti-inference rules prohibited any imputation or assumption of unreported data.

### Data Items

Data extracted from the 18 prespecified sections of the standardized form were organized for synthesis into thematic domains encompassing study characteristics, point-of-care context, AI system features, diagnostic performance, and clinical impact.

### Study, Clinical, and AI System Characteristics

Extracted variables encompassed study characteristics (author, year, country, setting, design, sample size, condition, and prevalence), point-of-care context (facility type, geographic setting, and World Bank income classification), operator characteristics (profession, experience, and training), and AI system features (architecture, regulatory status, modality, device type, and processing capability).

### Diagnostic Performance Outcomes

Primary outcomes were sensitivity and specificity; secondary outcomes included area under the receiver operating characteristic curve (AUC), positive predictive value (PPV), and negative predictive value (NPV), with 95% CIs where reported. For studies reporting multiple thresholds, we extracted values at the author-identified optimal threshold. No amendments were made to outcome definitions from the a priori protocol.

### Additional Outcomes and Contextual Variables

Additional items included comparator characteristics, explainability assessment (technique, user presentation, understanding, and decision impact), clinical outcomes (workflow, referrals, time, cost, and patient outcomes), task-shifting demonstrations, and training requirements.

### Study Risk of Bias Assessment

Methodological quality was assessed using QUADAS-2 across 4 domains: patient selection, index test, reference standard, and flow and timing. Overall risk of bias (RoB) was synthesized using predefined criteria: low (all domains low), low-moderate (1 unclear), moderate (1 high or multiple unclear), high (2 or more high), or very high (3 or more high or critical flaws). A total of 2 reviewers independently assessed each study, with disagreements resolved through discussion. Study authors were not contacted; no automation tools were used.

### Synthesis Methods

#### Data Preparation and Eligibility for Synthesis

All 20 studies were eligible for narrative synthesis; meta-analysis was precluded by substantial clinical heterogeneity (12 conditions, 6 modalities, and diverse settings and operators) and methodological heterogeneity (varying designs, reference standards, and thresholds). Only explicitly reported values were extracted without imputation or transformation. Studies with multiple subgroups or time points were synthesized using author-identified primary values.

#### Synthesis Approach

Data were synthesized narratively, organized around themes aligned with the review’s secondary objectives: (1) AI system and imaging characteristics, (2) diagnostic performance, (3) task-shifting demonstrations and training requirements, (4) explainability implementation, (5) clinical outcomes beyond diagnostic accuracy, and (6) methodological quality. Studies were grouped for synthesis based on prespecified characteristics extracted during data collection: clinical condition, imaging modality, resource context (World Bank income classification), operator type (specialist vs nonspecialist), and methodological quality strata (QUADAS-2 overall RoB). The decision process for creating synthesis groupings was determined a priori through the standardized data extraction domains, with studies categorized according to their extracted characteristics. Formal statistical tests for heterogeneity were not applicable given the narrative synthesis approach and absence of meta-analysis.

#### Subgroup Analyses

Prespecified subgroup analyses examined patterns across clinical conditions, imaging modalities, resource contexts (high-income country [HIC] vs lower-middle-income country [LMIC] or low-income country [LIC]), operator types, and methodological quality strata. Task-shifting and training requirements were characterized through tabulation. Explainability and clinical impact were categorized using the proposed XAI cascade and clinical impact pyramid frameworks.

#### Sensitivity Analyses

Sensitivity analysis assessed the robustness of diagnostic performance findings by restricting the analysis to studies with a low or low-moderate overall RoB, as assessed by the QUADAS-2. This quality-restricted analysis compared performance ranges (minimum-maximum) and central tendency measures (median) between the complete evidence base and the quality-restricted subset to evaluate the influence of methodological quality on reported diagnostic accuracy estimates. The results were compared descriptively without formal statistical testing.

### Data Presentation Methods

The results are presented in descriptive tables and figures including a PRISMA flow diagram, geographic distribution map, task-shifting illustration, forest plots grouped by condition, XAI cascade and clinical impact pyramid distributions, and QUADAS-2 visualizations.

### Proposed Synthesis Frameworks

A total of 2 frameworks were developed to systematically characterize evidence gaps not captured by standard diagnostic accuracy reporting. The first framework, an XAI implementation cascade (levels 0‐5), categorizes the depth of explainability implementation from nonmention through clinical user exposure to evaluation of whether explanations influenced clinical decisions. The second framework, a clinical impact pyramid (levels 0‐5), categorizes the maturity of clinical evidence from technical validation toward demonstration of health benefit. Diagnostic accuracy (level 0) is positioned at the pyramid’s base because, while foundational to any AI diagnostic system, it represents the least mature form of clinical evidence—demonstrating that a system works technically without establishing whether it improves patient outcomes. Process outcomes such as workflow efficiency and time to diagnosis occupy level 1. Clinical actions documented based on AI recommendations constitute level 2. Patient outcomes measured represent level 3, health system impact represents level 4, and population health outcomes represent level 5. These frameworks enabled systematic identification of evidence gaps and regulatory alignment assessment.

### Reporting Bias Assessment

Reporting bias was assessed qualitatively, given the narrative synthesis approach and substantial heterogeneity that precluded meta-analysis. The assessment examined (1) literature search comprehensiveness, including gray literature coverage; (2) patterns potentially indicating publication bias, including commercial system predominance and small-study effects; and (3) selective outcome reporting completeness for diagnostic accuracy metrics (sensitivity, specificity, AUC, PPV, and NPV) and precision estimates (95% CIs). Formal statistical methods for detecting publication bias (eg, Deeks funnel plot asymmetry testing) were not feasible; however, a qualitative assessment was conducted to characterize potential biases that may have affected the evidence base. A total of 2 reviewers independently assessed the reporting bias, with disagreements resolved through discussion.

### Certainty of Evidence Assessment

The certainty of evidence was assessed using the GRADE (Grading of Recommendations, Assessment, Development, and Evaluation) framework, adapted for diagnostic test accuracy studies. Following GRADE guidance, diagnostic accuracy studies are initiated at a high level of certainty. They are rated down across 5 domains: RoB (informed by QUADAS-2 assessments), indirectness (applicability and heterogeneity), inconsistency (variation in diagnostic performance), imprecision (CI width and sample size), and publication bias (completeness of literature search and selective reporting). Each domain was rated as no serious concern (no downgrade), serious concern (downgrade by 1 level), or grave concern (downgrade by 2 levels). Overall certainty was determined by summing downgrades across domains, with final ratings of high (⊕⊕⊕⊕), moderate (⊕⊕⊕◯), low (⊕⊕◯◯), or very low (⊕◯◯◯). A total of 2 reviewers independently assessed the certainty of evidence for each outcome, with disagreements resolved through discussion.

## Results

### Study Selection

The systematic search yielded 2113 records across 4 databases: Scopus (n=993), Web of Science (n=422), IEEE Xplore (n=408), and PubMed (n=290). After removing 707 duplicates, 1406 unique records underwent title and abstract screening.

Title and abstract screening excluded 1245 records. The most frequent exclusion reasons were wrong study type (n=319, including reviews and editorials), wrong imaging modality (n=224, such as computed tomography or magnetic resonance imaging), lack of clinical validation (n=181), absence of point-of-care setting validation (n=153), and publication before 2018 (n=144). Additional exclusions included studies without an AI or ML component (n=70), retrospective designs (n=59), device development studies only (n=40), studies without clinical decision support output (n=37), nonhuman studies (n=14), and studies with missing abstracts (n=4).

**Figure 1.**
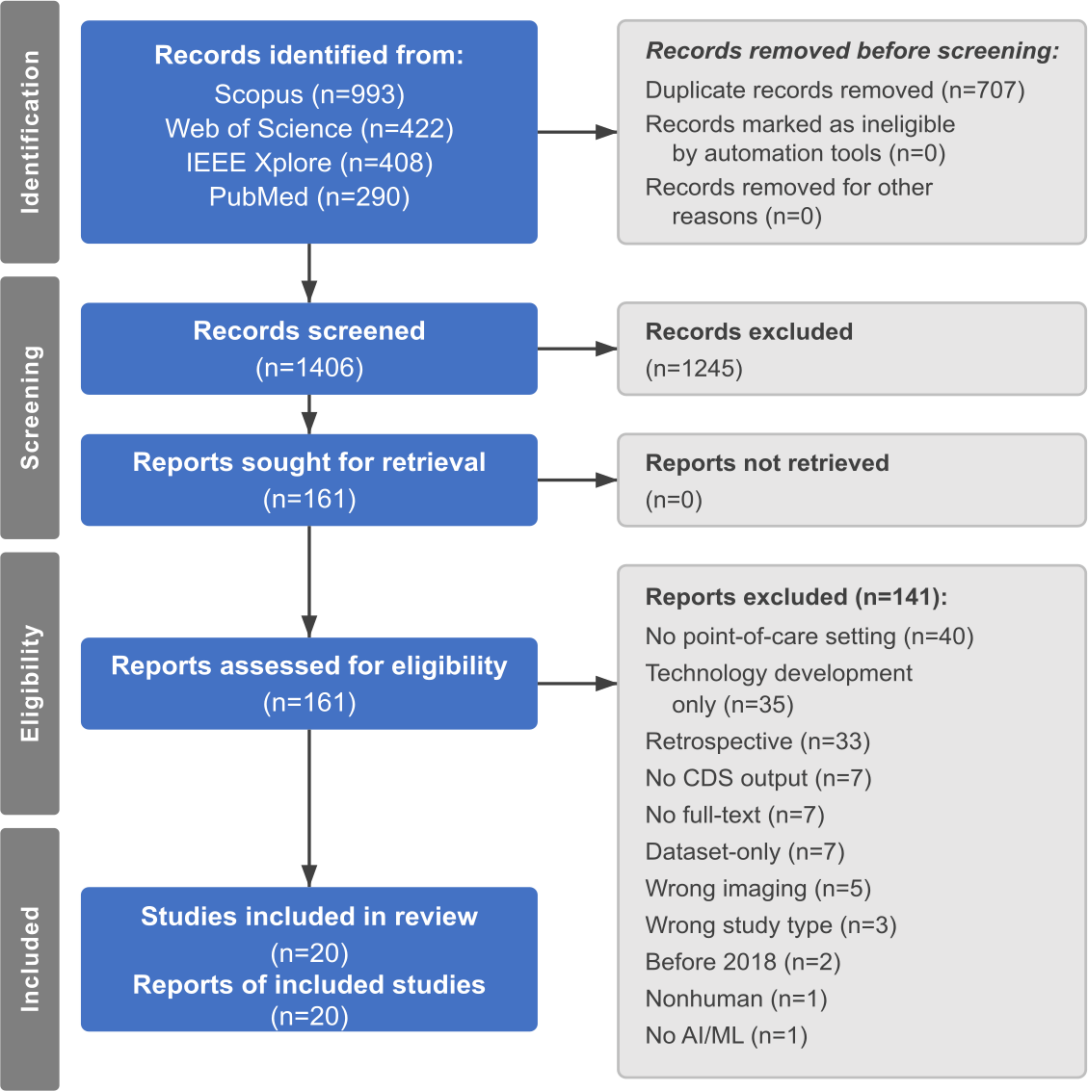
PRISMA 2020 flow diagram showing study selection process. AI: artificial intelligence; CDS: clinical decision support; ML: machine learning; PRISMA: Preferred Reporting Items for Systematic Reviews and Meta-Analyses.

All 161 records identified for full-text review were successfully retrieved; no potentially eligible reports were unretrievable. Full-text assessment excluded 141 studies, primarily due to the lack of point-of-care setting validation (n=40), device development without clinical implementation (n=35), and retrospective designs (n=33). Other exclusions included no clinical decision support output (n=7), unavailable full text (n=7), lack of clinical validation (n=7), wrong imaging modality (n=5), wrong study type (n=3), pre-2018 publication (n=2), nonhuman participants (n=1), and no AI or ML component (n=1). Twenty studies met all inclusion criteria and were included in the final synthesis (Figure 1). Human reviewers made all screening and selection decisions without the use of automation tools.

A representative sample of 30 borderline cases excluded at full-text review is provided in Multimedia Appendix 2. These studies met many criteria but failed to meet specific requirements, most commonly due to a retrospective design with AI applied to historical data (10/30, 33.3%) and the development of algorithms without subsequent clinical implementation (8/30, 26.7%). Other reasons included aspirational point-of-care claims without demonstrated deployment (5/30, 16.7%), specialist settings (3/30, 10%), and validation at a tertiary center only (2/30, 6.7%).

### Study Characteristics

The 20 included studies enrolled approximately 78,000 patients across 15 countries (Table 1). The temporal distribution demonstrated an accelerating research trajectory, with 85% (17/20) of studies published in 2023 or later.

**Table 1.** Characteristics of included studies.

Author	Country	Setting	Design	N	Condition	Prevalence (%)
Avgerinos et al [10]	Greece	Emergency department	NR^a^	53	DVT^b^	11.3
Berg et al [11]	Mexico	Hospital	Diagnostic accuracy	758	Breast cancer	7.4
Cao et al [12]	China	Township health centers	Prospective cohort	3705	Tuberculosis	2.1
Chen et al [13]	China	ED^c^; primary care	Cross-sectional	364	Ophthalmic emergencies	19.1 urgent
Fergus et al [14]	UK	Home care	Clinical trial	216	Pressure ulcers	100
Heydon et al [15]	UK	Screening program	Diagnostic accuracy	30,405	Diabetic retinopathy	7.3
Iacob et al [16]	Romania	Primary care	Diagnostic accuracy	1780	Cardiac pathology	32.9
Jaremko et al [17]	Canada	Primary care	Implementation	306	Hip dysplasia	2
Jayaraman et al [18]	India	Mobile units	NR	25,598	Tuberculosis	4
Kazemzadeh et al [19]	Zambia	Health facilities	Prospective observational	1827	Tuberculosis	10.5
Love et al [20]	Mexico	Government hospital	Pilot study	32	Breast masses	6
Malherbe [21]	South Africa	Primary care	Prospective cohort	203	Breast cancer	NR
Marquez et al [22]	Philippines	Mixed	Retrospective	5740	Tuberculosis	13.1
Nath et al [23]	India	PHC^d^+ tertiary centers	Diagnostic accuracy	4363	Tuberculosis	53.7
Nothnagel and Aslam [24]	Germany	Hospital	Feasibility study	58	DVT	9
Papachristou et al [25]	Sweden	Primary care	Prospective trial	253	Melanoma	8.3
Poli et al [26]	India	Community screening	Exploratory intervention	2052	Cervical cancer	4.3
Yang et al [27]	Madagascar	Rural villages	Diagnostic accuracy	113	Helminthiases	70
Yu et al [28]	Sudan	Primary hospitals	Diagnostic accuracy	85‐189	Malaria	52.6
Zhu et al [29]	United States	Community screening	NR	385	Vision-threatening diseases	20.5

a NR: not reported.

b DVT: deep vein thrombosis.

c ED: emergency department.

d PHC: primary health center.

**Figure 2.**
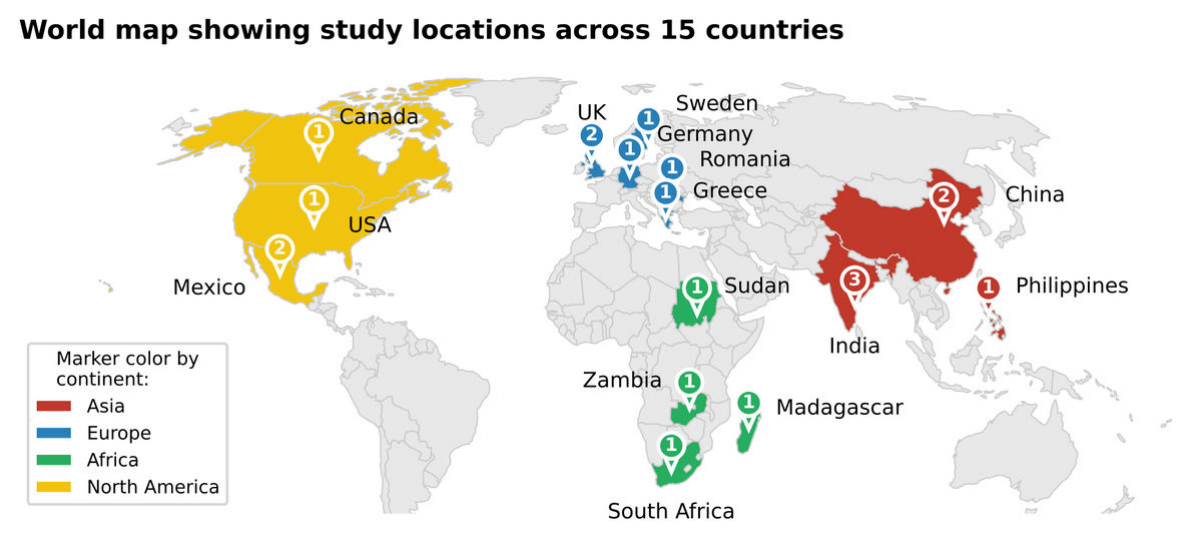
Illustrating the geographic distribution of included studies.

The geographic distribution spanned 4 continents, with Asia and Europe each contributing 30% (6/20) of the studies, followed by Africa and North America, each at 20% (4/20; Figure 2). India 15% (3/20), China 10% (2/20), Mexico 10% (2/20), and the United Kingdom 10% (2/20) contributed multiple studies; single studies originated from 11 additional countries. Notably, no studies were conducted in South America or Oceania.

World Bank income classification (Table 2) revealed substantial research activity in resource-limited settings, with 60% (12/20) of studies conducted outside HICs, including 2 in LICs (Sudan and Madagascar).

Sample sizes ranged from 32 to 30,405 participants (median 375, IQR 158-2879). The most extensive studies by sample size were Heydon et al [15] (n=30,405) evaluating diabetic retinopathy in the United Kingdom screening program, Jayaraman et al [18] (n=25,598) evaluating tuberculosis in Indian mobile units, and Marquez et al [22] (n=5740) evaluating tuberculosis in the Philippines. The clinical domains were diverse, including tuberculosis (5/20, 25%), breast cancer (3/20, 15%), deep vein thrombosis (DVT) (2/20, 10%), and diabetic retinopathy (2/20, 10%). Single studies addressed cardiac pathology, cervical cancer, developmental hip dysplasia, malaria, ophthalmic emergencies, parasitic infections, pressure ulcers, and melanoma. Disease prevalence ranged from 2% (hip dysplasia) to 70% (helminthiases).

**Table 2.** Point-of-care setting and operator characteristics.

Author	Facility type	Geographic	Resource context	Operator	Prior experience	Training duration
Avgerinos et al [10]	ED^a^	NR^b^	HIC^c^	Clinical researcher + vascular residents	None	1 hour
Berg et al [11]	Hospital	NR	UMIC^d^	Radiologist + research coordinators	None	30 min
Cao et al [12]	Township PHC^e^	Rural	UMIC	Local radiologists	NR	NR
Chen et al [13]	ED; PHC	NR	UMIC	Patients; staff	Mixed	NR
Fergus et al [14]	Home care	NR	HIC	District nurses	Varied	NR
Heydon et al [15]	Screening	NR	HIC	Trained graders	Trained	NR
Iacob et al [16]	PHC	NR	HIC	Family physicians	Nonspecialist	6 months
Jaremko et al [17]	PHC	Towns	HIC	Nurses, LPNs^f^, FPs^g^	Nonspecialist	2‐4 d
Jayaraman et al [18]	Mobile vans	Rural	LMIC^h^	Radiologist	15 years	NR
Kazemzadeh et al [19]	Health facilities	NR	LMIC	NR	NR	NR
Love et al [20]	Hospital	NR	UMIC	Medical student, nurse	None	30 min
Malherbe [21]	PHC	Urban	UMIC	GP^i^ with ultrasound experience	Experienced	NR
Marquez et al [22]	Mixed	NR	LMIC	Health workers	NR	NR
Nath et al [23]	PHC + TC^j^	NR	LMIC	X-ray technicians	NR	Simple process
Nothnagel and Aslam [24]	Hospital	NR	HIC	Nonspecialists	None	1 hour
Papachristou et al [25]	PHC	NR	HIC	GPs, residents	Varied	On-site
Poli et al [26]	Community	Rural	LMIC	Nurses	3‐12 years VIA^k^	NR
Yang et al [27]	Rural villages	Rural	LIC^l^	Local HCWs^m^	NR	NR
Yu et al [28]	Primary hospitals	Rural	LIC	Microscopists	NR	Online session
Zhu et al [29]	Community	NR	HIC	Medical students	None	Equipment training

a ED: emergency department.

b NR: not reported.

c HIC: high-income country.

d UMIC: upper-middle-income country.

e PHC: primary health center.

f LPN: licensed practical nurse.

g FP: family physician.

h LMIC: lower-middle-income country.

i GP: general practitioner.

j TC: tertiary center.

k VIA: visual inspection with acetic acid.

l LIC: low-income country.

m HCW: health care worker.

### Point-of-Care Settings and Operator Characteristics

Point-of-care settings varied substantially across the 20 studies, reflecting the versatility of AI-assisted imaging (Table 2). Primary care facilities predominated (6/20, 30%), followed by community screening programs (4/20, 20%), general hospitals (3/20, 15%), emergency departments (2/20, 10%), and mixed settings (2/20, 10%). Deployments also included mobile diagnostic units [18], home care [14], and remote rural villages [27], demonstrating adaptability to decentralized health care delivery. Geographic settings, where reported, revealed that 25% (5/20) of studies were conducted in rural areas, 5% (1/20) in urban settings, and 70% (14/20) did not specify a geographic context.

### Task-Shifting Evidence

**Figure 3.**
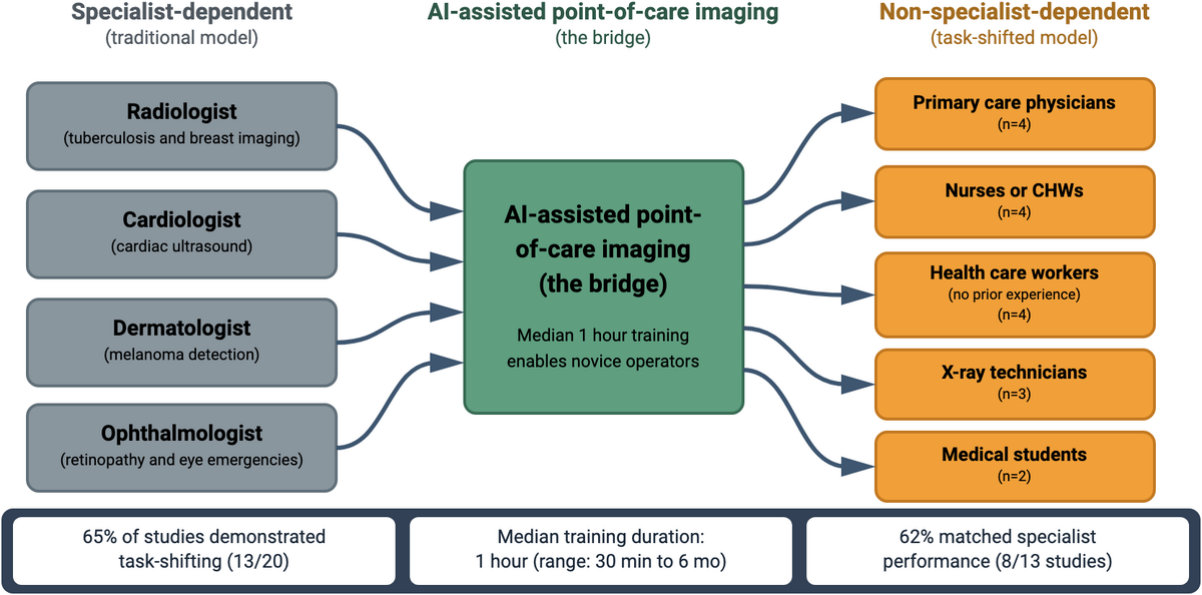
AI-enabled task-shifting in point-of-care imaging. The figure illustrates the transfer of diagnostic tasks from specialist physicians (left) to nonspecialist health care workers (right) enabled by AI-assisted point-of-care imaging systems. AI: artificial intelligence; CHW:.

A central finding of this review is evidence for task-shifting enabled by AI-assisted imaging (Figure 3). A total of 13 (13/20, 65%) studies explicitly demonstrated task-shifting from specialists to nonspecialist health care workers, transferring tasks traditionally performed by radiologists, cardiologists, dermatologists, and ophthalmologists to primary care physicians, nurses, community health workers, and medical students. The operator types included primary care physicians (n=4), nurses or community health workers (n=4), health care workers without prior imaging experience (n=4), x-ray technicians (n=3), and medical students (n=2). Further, 7 studies did not specify the primary operator. Notably, most task-shifted operators had no prior experience with the specific imaging modality before training.

### Training Requirements

Training requirements varied across studies reporting training details (13/20, 65%). Among studies reporting training details, the duration ranged remarkably from 30 minutes to 6 months (median 1 h; n=6 studies reporting specific numeric durations); IQR was not calculated due to the small number of studies and extreme right skew of the distribution. A total of 6 of 13 (46%) studies achieved acceptable diagnostic performance with training programs of 1 hour or less. Training formats included brief PowerPoint (Microsoft Corp) presentations (n=4), app-based training (n=2), in-person demonstrations (n=3), hands-on supervised practice (n=4), and online training sessions (n=2).

No clear dose-response relationship emerged between training duration and diagnostic performance. Studies with 30‐60 minutes of training achieved sensitivities of 100%, comparable to or exceeding those with longer programs. Love et al [20] reported a medical student achieving 100% sensitivity and specificity for breast mass classification after 30 minutes of PowerPoint training. Avgerinos et al [10] demonstrated that nonultrasound-trained providers achieved 100% sensitivity and 95.7% specificity for DVT after 1 hour of app training. Similarly, Nothnagel and Aslam [24] found that health care professionals without ultrasound training achieved 100% sensitivity and 90.6% specificity for DVT after 1 hour. Berg et al [11] reported that research coordinators achieved 86% sensitivity for breast cancer triage after 30 minutes of training, although specificity was lower (33%) with portable devices.

Among studies where task-shifted performance was explicitly compared to specialists or predefined thresholds, 62% (8/13) demonstrated performance comparable to specialists, 31% (4/13) met predefined acceptable thresholds, and only 8% (1/13) showed performance below acceptable levels.

### AI System Characteristics

Having characterized this study’s settings and operators, we next examined the AI systems themselves. The 20 studies evaluated 18 distinct AI systems (Table 3): commercial products (12/20, 60%), research prototypes (7/20, 35%), and 1 open-source system (Kankanet). Commercial systems included ThinkSono Guidance, Koios DS, EyeArt, MEDO-Hip, Genki, Breast AI, qXR, Dermalyser, and SELENA+.

**Table 3.** AI^a^ system and imaging characteristics.

Author	AI system	Architecture	Commercial status	Regulatory	Modality	Device type
Avgerinos et al [10]	ThinkSono Guidance	NR^b^	Commercial	CE^c^ IIb^d^	POCUS^e^	Handheld
Berg et al [11]	Koios DS	NR	Commercial	NR	Breast ultrasound	Portable/Cart
Cao et al [12]	JF CXR-1 v3.0	DL^f^	Commercial	NR	CXR^g^	NR
Chen et al [13]	EE-Explorer	DenseNet + XGBoost	Research	NR	Photography	Mobile
Fergus et al [14]	Faster R-CNN^h^	ResNet101	Research	NR	Photography	Mobile
Heydon et al [15]	EyeArt v2.1.0	DL	Commercial	CE	Fundus	NR
Iacob et al [16]	Wis+	CNN^h^	Commercial	NR	Cardiac US	Cart
Jaremko et al [17]	MEDO-Hip	UNet CNN	Commercial	FDA^i^	Hip US	Handheld
Jayaraman et al [18]	Genki v1	UNet + Xception	Commercial	NR	CXR	Mobile
Kazemzadeh et al [19]	Google tuberculosis AI	NR	Research	NR	CXR	NR
Love et al [20]	Triage-CADx	Inception-v3	Research	NR	Breast ultrasound	Portable
Malherbe [21]	Breast AI	NR	Commercial	SAHPRA^j^	Breast ultrasound	Handheld
Marquez et al [22]	qXR v3	NR	Commercial	NR	CXR	NR
Nath et al [23]	DecXpert v1.4	CNN + attention	Research	NR	CXR	Basic
Nothnagel and Aslam [24]	ThinkSono Guidance	U-Net CNN	Commercial	CE I	POCUS	Handheld
Papachristou et al [25]	Dermalyser	CNN	Commercial	NR	Dermoscopy	Handheld
Poli et al [26]	VIA-AI	EfficientNet	Research	NR	Colposcopy	Mobile
Yang et al [27]	Kankanet	SSD + MobileNet	Open-source	NR	Microscopy	Mobile
Yu et al [28]	Malaria Screener	DL	Research	NR	Microscopy	Mobile
Zhu et al [29]	SELENA+	CNN	Commercial	NR	Fundus	Cart

a AI: artificial intelligence.

b NR: not reported.

c CE: conformité européenne.

d IIb: Class IIb medical device software.

e POCUS: point-of-care ultrasound.

f DL: deep learning.

g CXR: chest x-ray.

h CNN: convolutional neural network.

i FDA: Food and Drug Administration.

j SAHPRA: South African Health Products Regulatory Authority.

### Architectures

Deep learning architectures dominated (18/20, 90%), with 1 hybrid system combining deep learning and gradient boosting (EE-Explorer), and 1 study not reporting its architecture. No studies used traditional ML approaches alone. Specific architectures included convolutional neural networks of unspecified type (n=7), U-Net or UNet-like architectures for segmentation tasks (n=3), Inception-v3 for classification (n=2), and individual studies using DenseNet, EfficientNet, ResNet-101, SSD with MobileNet, and Faster R-CNN.

### Regulatory Status

Regulatory status was largely unreported, representing a critical gap. Only 25% (5/20) of studies reported any regulatory approval: FDA clearance for MEDO-Hip [17], CE (conformité européenne) marking for ThinkSono Guidance (Class IIb [10] and Class I [24]) and EyeArt [15], and South African SAHPRA (South African Health Products Regulatory Authority) approval for Breast AI [21]. The remaining 75% (15/20) either did not report their regulatory status or used unapproved systems.

### Imaging Modalities and Devices

Imaging modalities reflected in point-of-care contexts included ultrasound (7/20, 35%), chest x-ray (5/20, 25%), smartphone photography (3/20, 15%), fundus photography (2/20, 10%), microscopy (2/20, 10%), and dermoscopy (1/20, 5%). Device types emphasized portability, with mobile phone-based devices (6/20, 30%), handheld devices (5/20, 25%), cart-based devices (3/20, 15%), and portable units (2/20, 10%) being the most prevalent; device type was not reported for 20% (4/20) of studies. Real-time processing was reported in 70% (14/20) of studies, and offline capability—critical for settings with unreliable connectivity—was confirmed in 30% (6/20) of the studies.

### Diagnostic Performance

#### Overview

**Figure 4.**
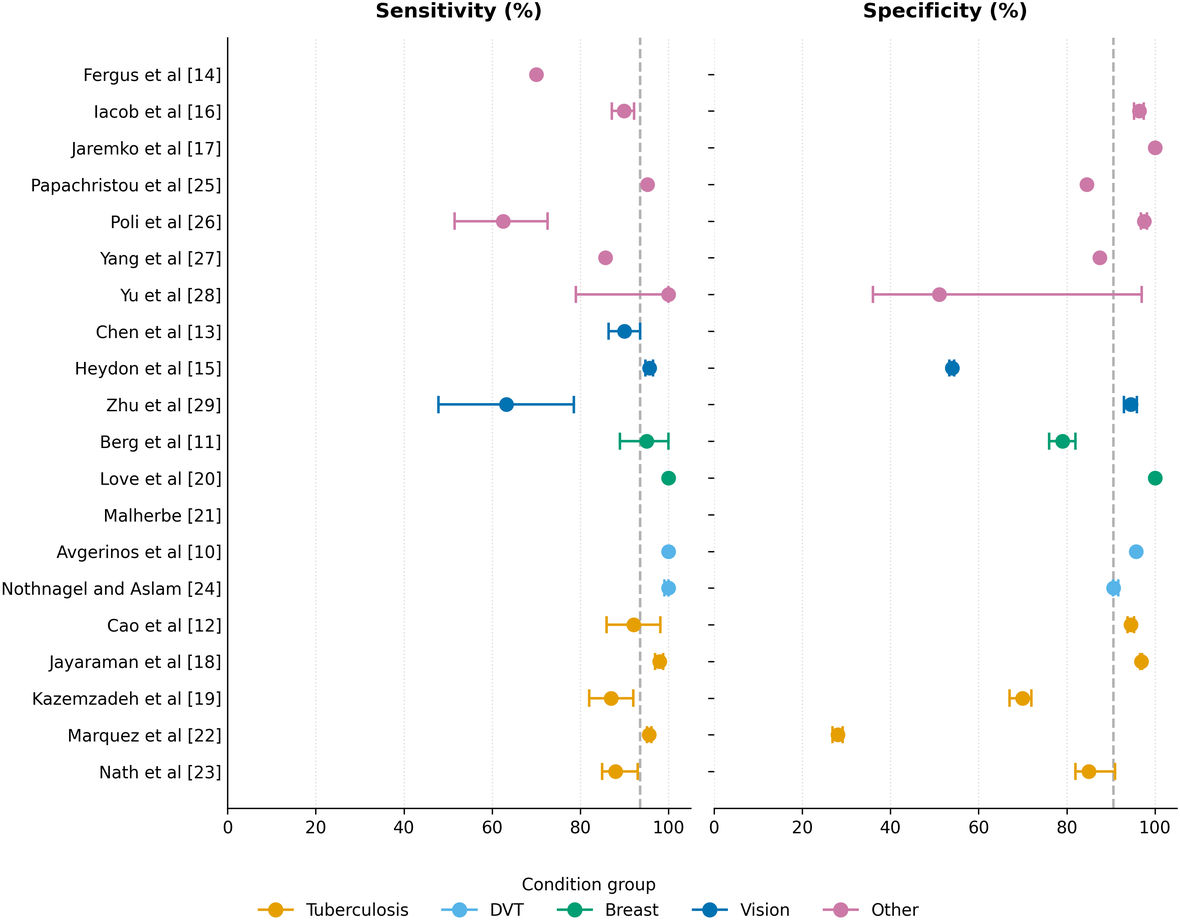
Forest plot of diagnostic accuracy showing sensitivity (left panel) and specificity (right panel) with 95% CIs, grouped by clinical condition. Vertical dashed lines indicate median values (sensitivity 93.6%, specificity 90.6%). Each horizontal dash between panels corresponds to 1 study and serves as a visual guide to help readers track data points across both metrics for the same study. The absence of a data point in the corresponding panel indicates that metrics are missing. DVT: deep vein thrombosis [10-29].

We next assessed the primary outcomes of diagnostic accuracy. Diagnostic performance metrics were reported heterogeneously (Table 4). Sensitivity, reported in 90% (18/20) of studies, ranged from 62.5% to 100% (median 93.6%, IQR 87%-98%). Specificity, reported in 85% (17/20), ranged from 28.1% to 100% (median 90.6%, IQR 74.5%-96.7%), reflecting a broader variation that is context-dependent and likely due to threshold optimization (Figure 4 [10-29]). AUC was reported in 50% (10/20) of studies, with a range of 0.63 to 1.00 (median 0.91, IQR 0.82-0.96). PPV ranged from 14% to 92.6% (median 53.4%, IQR 24.3%-86.8%), reflecting the effects of disease prevalence, while NPV was consistently high (median 98.4%, IQR 95.1%-99%), supporting its use for ruling out disease.

**Table 4.** Diagnostic performance summary.

Author	Condition	Value, N	Sensitivity % (95% CI)	Specificity % (95% CI)	AUC^a^ (95% CI)
Avgerinos et al [10]	DVT^b^	53	100 (NR^c^)	95.7 (NR)	NR
Berg et al [11]	Breast cancer	758	95 (89‐100)	79 (76‐82)	0.95 (0.91‐0.99)
Cao et al [12]	Tuberculosis	3705	92.1 (86.0‐98.2)	94.5 (93.8‐95.3)	NR
Chen et al [13]	Ophthalmic	364	90‐96.2 (86.4‐93.6)	NR	0.98 (0.97‐1.00)
Fergus et al [14]	Pressure ulcer	216	70 (NR)	NR	0.63‐0.93
Heydon et al [15]	Referable DR^d^	30,405	95.7 (94.8‐96.5)	54 (53.4‐54.5)	NR
Iacob et al [16]	Cardiac	1780	89.9 (87.2‐92.2)	96.5 (95.3‐97.5)	0.94 (0.92‐0.96)
Jaremko et al [17]	DDH^e^	306	NR	100 (NR)	NR
Jayaraman et al [18]	Tuberculosis	25,598	98 (97‐98.8)	96.9 (96.6‐97.1)	NR
Kazemzadeh et al [19]	Tuberculosis	1827	87 (82‐92)	70 (67‐72)	0.87 (0.84‐0.90)
Love et al [20]	Breast mass	32	100 (NR)	100 (NR)	1.00
Malherbe [21]	Breast cancer	203	NR	NR	NR
Marquez et al [22]	Tuberculosis	5740	95.6 (95.1‐96.1)	28.1 (26.9‐29.2)	0.82 (0.80‐0.84)
Nath et al [23]	Tuberculosis	4363	88 (85‐93)	85 (82‐91)	0.85 (0.82‐0.87)
Nothnagel and Aslam [24]	DVT	58	100 (99.1‐100)	90.6 (90.5‐91.7)	NR
Papachristou et al [25]	Melanoma	253	95.2 (NR)	84.5 (NR)	0.96 (0.93‐0.98)
Poli et al [26]	Cervical	2052	62.5 (51.5‐72.6)	97.6 (96.8‐98.2)	0.76
Yang et al [27]	Lumbricoides	113	85.7 (NR)	87.5 (NR)	NR
Yu et al [28]	Malaria	85‐189	86.9‐100 (79-100)	51.1‐91.1 (36-97)	NR
Zhu et al [29]	VTDs^f^	385	63.2 (47.8‐78.5)	94.5 (93‐95.9)	NR

a AUC: area under the receiver operating characteristic curve.

b DVT: deep vein thrombosis.

c NR: not reported.

d DR: diabetic retinopathy.

e DDH: developmental dysplasia of the hip.

f VTD: vision-threatening disease.

#### Performance by Clinical Condition

Tuberculosis studies (n=5) demonstrated sensitivities ranging from 87% to 98% (median 92.1%, IQR 87.5%-96.8%), specificities ranging from 28% to 97% (median 85%, IQR 49%-95.7%), and AUCs ranging from 0.82 to 0.87. Lower specificity in some studies (Marquez et al [22]: 28%) reflected operational decisions aimed at maximizing sensitivity in high-prevalence settings. Both DVT studies achieved 100% sensitivity with specificity ranging from 90.6% to 95.7%, demonstrating excellent performance. Breast imaging studies (n=3) achieved a sensitivity of 95%‐100%, although specificity varied (79%‐100%) depending on the operator’s training and the type of device used. Diabetic retinopathy studies (n=2) showed more variable performance (sensitivity: 63%‐96%), reflecting challenges with image quality and the presence of ungradable images.

#### Performance by Resource Context

Analysis by resource context revealed slightly lower performance in LMIC/LIC settings: HIC studies (n=8) achieved a median sensitivity of 95.2% (IQR 70%-100%) and specificity of 94.5% (IQR 84.5%-96.5%), compared with a median sensitivity of 88% (IQR 85.7%-98%) and specificity of 85% (IQR 51.1%-96.9%) in LMIC/LIC studies (n=7). However, these differences may reflect the clinical context rather than inherent limitations of AI. Several LMIC studies achieved excellent performance: Jayaraman et al [18] (India) reported 98% sensitivity and 97% specificity for tuberculosis, Nath et al [23] (India) achieved 88% sensitivity and 85% specificity, and Kazemzadeh et al [19] (Zambia) demonstrated 87% sensitivity and 70% specificity.

#### AI Versus Comparator Performance

All 20 studies included some form of comparator (Table 5). A total of 8 studies compared AI against the reference standard alone, while 5 directly compared AI to specialist physicians, 3 compared it to nonspecialist physicians or nurses, 2 used standard-of-care comparisons, and 2 used expert panels.

**Table 5.** AI versus comparator performance^a^.

Author	Comparator	Metrics	AI, n/N (%)	Comparator, n/N (%)	Statistical test	Result
Avgerinos et al [10]	D-dimer	Sensitivity; specificity	6/6 (100); 45/47 (95.7)	6/6 (100); 17/47 (36.2)	NR	AI superior (specificity)
Berg et al [11]	Radiologist	Sensitivity; specificity	53/56 (95); 554/702 (79)	56/56 (100); 612/702 (87)	DeLong	Noninferior (*P*=.10)
Cao et al [12]	Local radiologists	Sensitivity; specificity	70/76 (92.1); 3431/3629 (94.5)	25/76 (32.9); 3604/3629 (99.3)	McNemar	AI superior (sensitivity; *P*<.05)^b^
Chen et al [13]	Triage nurses	Accuracy	246/261 (94.3)	215/261 (82.4)	McNemar	AI superior (*P*<.001)
Kazemzadeh et al [19]	10 radiologists	Sensitivity; specificity	167/192 (87); 1145/1635 (70)	76^c^; 82^c^	Obuchowski-Rockette-Hillis procedure	AI superior (sens; *P*<.001)
Nath et al [23]	3 radiologists	Sensitivity; specificity	2064/2345 (88); 1715/2018 (85)	1665/2345 (71, PPV^d^); NR^e^ (84, NPV^f^)	NR	AI superior (sensitivity)^g^
Papachristou et al [25]	PCPs^h^	PPV	20/56 (36)	12/51 (24)	Logistic regression	AI superior (OR^i^=26.55, *P*=.002)

a The table includes only studies with direct head-to-head comparison data. All 20 studies had some form of comparator (reference standard or human comparator).

b Exact *P* value not reported in source; *P*<.05 indicates statistical significance threshold met.

c Radiologist performance represents the mean across 10 independent readers; absolute values cannot be expressed as a single numerator/denominator.

d PPV: positive predictive value.

e NR: not reported. Absolute values for comparator negative predictive value could not be reliably derived.

f NPV: negative predictive value.

g Direct metric comparison limited; comparator study reported positive predictive value or negative predictive value rather than sensitivity or specificity.

h PCP: primary care physician.

i OR: odds ratio.

Among studies with head-to-head comparisons, AI demonstrated superior performance in most cases. Cao et al [12] found AI sensitivity (92.1%) significantly exceeded that of local radiologists (32.9%) for tuberculosis detection (McNemar test, *P*<.05; exact value not reported). Kazemzadeh et al [19] reported that AI achieved higher sensitivity (87%) than 10 experienced radiologists (mean 76%) for tuberculosis screening (*P<.*001). Chen et al [13] demonstrated that AI accuracy (94.3%) significantly exceeded that of triage nurses (82.4%) for ophthalmic emergencies (*P*<.001). Papachristou et al [25] showed AI substantially outperformed primary care physicians for melanoma detection (OR 26.55, *P*=.002 vs OR 3.35, *P*=.02). A total of 2 studies demonstrated equivalence: Berg et al [11] found AI performance with standard ultrasound (AUC 0.95) was noninferior to expert radiologists (AUC 0.98, *P*=.10), and Avgerinos et al [10] showed AI-assisted point-of-care ultrasound matched D-dimer sensitivity (100%) while achieving substantially higher specificity (95.7% vs 36.2%).

Notably, Berg et al [11] demonstrated that AI performance decreased when images were acquired by minimally trained operators using portable devices rather than specialists using standard equipment, highlighting the importance of image quality for AI performance.

### Explainability (XAI) Assessment

**Figure 5.**
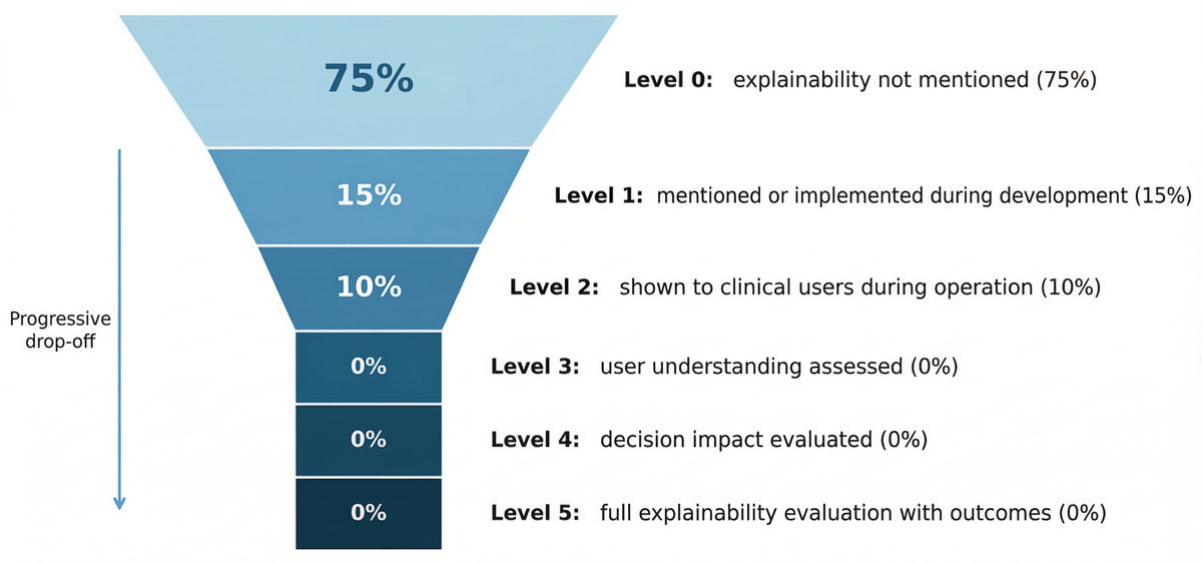
Illustrating the distribution of studies across explainability implementation levels.

Beyond diagnostic accuracy, we examined the implementation of explainability features using a proposed cascade framework (Table 6), assessing progression from no mention through clinical evaluation of decision impact. The cascade revealed a progressive drop-off (Figure 5): 75% (15/20) of studies did not mention explainability at all (level 0). A total of 3 studies (3/20, 15%) implemented XAI during development but did not provide explanations to clinical users (level 1): Chen et al [13] used Grad-CAM (Gradient-weighted Class Activation Mapping) and SHAP (Shapley Additive Explanation) values, Nath et al [23] used heatmaps and attention mechanisms, and Poli et al [26] used Grad-CAM. Only 2 of 10 (10%) studies provided explanations to clinical users (level 2): Jayaraman et al [18] displayed heatmaps to radiologists, and Yang et al [27] showed bounding boxes with confidence scores to health care workers. Critically, no studies assessed whether clinicians understood the explanations (level 3) or evaluated whether XAI influenced clinical decisions (levels 4‐5), representing a complete drop-off above level 2.

Among studies mentioning XAI, the techniques included Grad-CAM activation maps (n=2), heatmaps (n=2), attention mechanisms (n=2), bounding boxes with confidence scores (n=1), and SHAP values (n=1); no studies implemented Local Interpretable Model-Agnostic Explanations.

**Table 6.** XAI^a^ assessment. Cascade level: 0=not mentioned, 1=implemented only, 2=shown to users, 3=understanding assessed, 4=decision impact evaluated, 5=full evaluation.

Author	XAI mentioned	Technique	Shown to users	Understanding assessed	Decision impact	Cascade level
Avgerinos et al [10]	No	—^b^	No	No	No	0
Berg et al [11]	No	—	No	No	No	0
Cao et al [12]	No	—	No	No	No	0
Chen et al [13]	Yes	GC^c^, SHAP^d^	NR^e^	No	No	1
Fergus et al [14]	No	—	No	No	No	0
Heydon et al [15]	No	—	No	No	No	0
Iacob et al [16]	No	—	No	No	No	0
Jaremko et al [17]	No	—	No	No	No	0
Jayaraman et al [18]	Yes	Heatmap, ATT^f^	Yes	No	No	2
Kazemzadeh et al [19]	No	—	No	No	No	0
Love et al [20]	No	—	No	No	No	0
Malherbe [21]	No	—	No	No	No	0
Marquez et al [22]	No	—	No	No	No	0
Nath et al [23]	Yes	Heatmap, ATT	NR	No	No	1
Nothnagel and Aslam [24]	No	—	No	No	No	0
Papachristou et al [25]	No	—	No	No	No	0
Poli et al [26]	Yes	GC	No	No	No	1
Yang et al [27]	Yes	BB^g^, Conf^h^	Yes	No	No	2
Yu et al [28]	No	—	No	No	No	0
Zhu et al [29]	No	—	No	No	No	0

a XAI: explainable artificial intelligence.

b Not available.

c GC: Gradient-weighted Class Activation Mapping.

d SHAP: Shapley Additive Explanations.

e NR: not reported.

f ATT: attention mechanism.

g BB: bounding box.

h Conf: confidence score.

### Clinical Outcomes Beyond Diagnostic Accuracy

**Figure 6.**
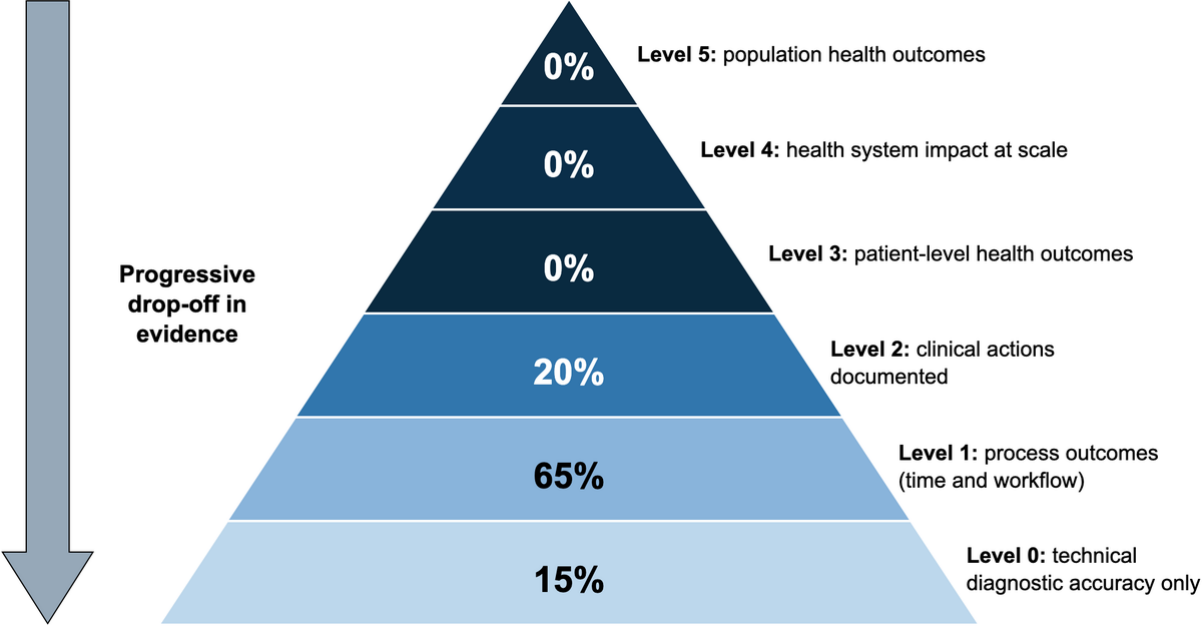
Distribution of evidence across clinical impact levels. The pyramid represents evidence maturity from technical validation (base) toward demonstration of patient benefit (apex).

A second critical finding is the evidence gap between technical validation and clinical impact. Applying the clinical impact pyramid framework (Table 7), the distribution revealed (Figure 6): 15% (3/20) of studies reported technical accuracy only (level 0: Kazemzadeh et al [19], Love et al [20], Yang et al [27]); 65% (13/20) reported process outcomes such as time to diagnosis and workflow efficiency (level 1); and 20% (4/20) documented clinical actions based on AI recommendations (level 2: Chen et al [13], Iacob et al [16], Jaremko et al [17], and Malherbe [21]). Critically, no studies measured patient-level health outcomes (level 3) or health system impact (levels 4‐5). Specific outcomes reported included workflow efficiency (10/20, 50%), time to diagnosis (9/20, 45%; AI processing typically <1 min), referral documentation (6/20, 30%), and cost analysis (5/20, 25%; projection-based rather than measured savings). The absence of patient outcome data is notable, given that several studies enrolled thousands of patients in operational settings (Heydon et al, n=30,405 [15]; Jayaraman et al, n=25,598 [18]; Marquez et al, n=5740 [22]), where outcome tracking was feasible.

**Table 7.** Clinical outcomes beyond diagnostic accuracy. Impact level: 0=technical accuracy only, 1=process outcomes, 2=clinical actions documented, 3=patient outcomes measured, 4=health system impact, and 5=population health outcomes.

Author	Workflow outcomes	Referrals	Time impact	Cost analysis	Patient outcomes	Impact level
Avgerinos et al [10]	Yes	17/53 (32%) discharged	Yes (37 min)	No	No	1
Berg et al [11]	Yes	NR^a^	No	No	No	1
Cao et al [12]	No	Yes	No	No	No	1
Chen et al [13]	Yes	Yes	No	No	No	2
Fergus et al [14]	Yes	NR	Yes (2‐3 s)	No	No	1
Heydon et al [15]	Yes	NR	No	Yes	No	1
Iacob et al [16]	Yes	Yes	No	Yes	No	2
Jaremko et al [17]	Yes	Yes	Yes (<1 min)	No	No	2
Jayaraman et al [18]	Yes	Yes	Yes (<1 min)	No	No	1
Kazemzadeh et al [19]	No	NR	Yes (<1 min)	No	No	0
Love et al [20]	No	NR	No	No	No	0
Malherbe [21]	Yes	NR	Yes	No	No	2
Marquez et al [22]	Yes	NR	No	No	No	1
Nath et al [23]	Yes	NR	Yes (<1 min)	Yes	No	1
Nothnagel and Aslam [24]	Yes	NR	No	No	No	1
Papachristou et al [25]	Yes	NR	No	No	No	1
Poli et al [26]	Yes	NR	No	No	No	1
Yang et al [27]	Yes	NR	No	Yes	No	0
Yu et al [28]	Yes	NR	Yes	Yes	No	1
Zhu et al [29]	Yes	Yes	Yes (35.6 s)	No	No	1

a NR: not reported.

### Methodological Quality

**Figure 7.**
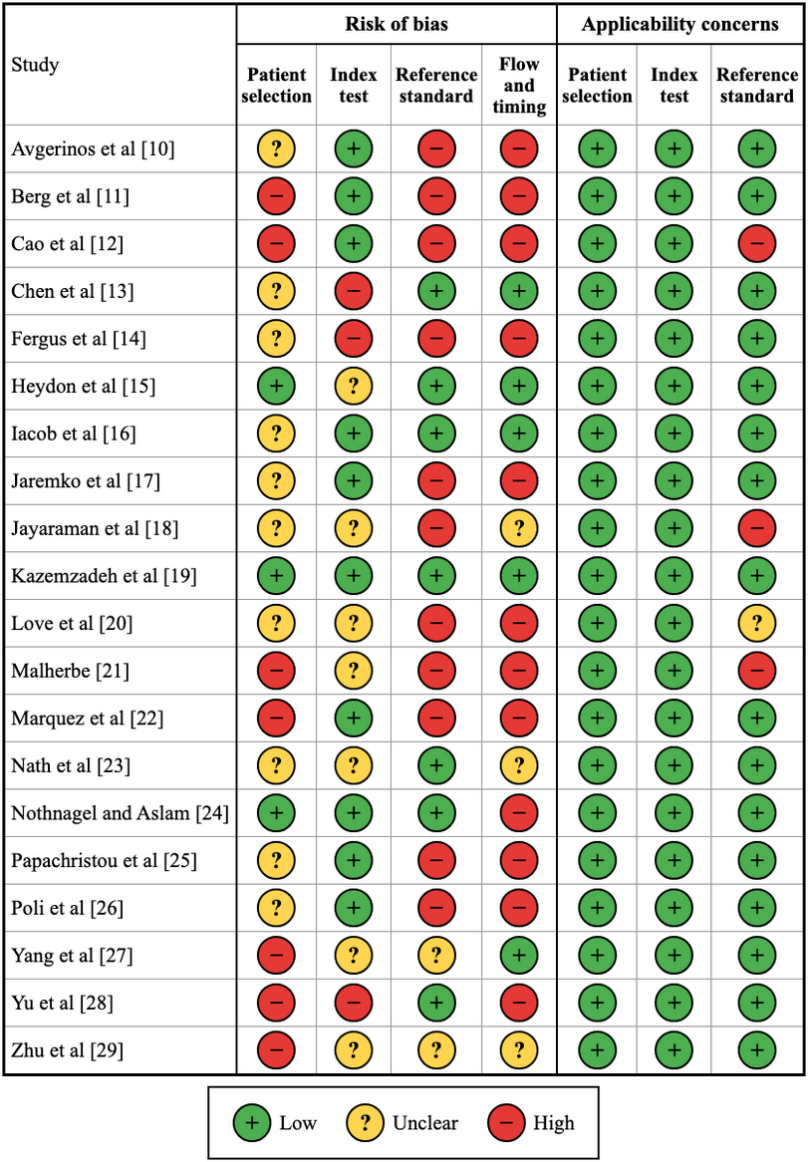
Summarizing the risk of bias and applicability concerns across all QUADAS-2 domains. QUADAS-2: Quality Assessment of Diagnostic Accuracy Studies 2 [10-29].

We assessed methodological quality using the QUADAS-2 tool. Assessment revealed substantial methodological concerns (Table 8; Figures7  8 [10-29]; detailed justifications for each judgment are provided in Multimedia Appendix 4). Thus, 70% (14/20) of the evidence base carries high or very high RoB, substantially limiting confidence in reported performance estimates.

**Table 8.** QUADAS-2^a^ RoB^b^ summary.

Author	D1^c^ RoB	D2^d^ RoB	D3^e^ RoB	D4^f^ RoB	D1 app^g^	D2 app	D3 app	Overall
Avgerinos et al [10]	U^h^	L^i^	H^j^	H	L	L	L	High
Berg et al [11]	H	L	H	H	L	L	L	Very high
Cao et al [12]	H	L	H	H	L	L	H	Very high
Chen et al [13]	U	H	L	L	L	L	L	Moderate
Fergus et al [14]	U	H	H	H	L	L	L	Very high
Heydon et al [15]	L	U	L	L	L	L	L	Low
Iacob et al [16]	U	L	L	L	L	L	L	Low-moderate
Jaremko et al [17]	U	L	H	H	L	L	L	High
Jayaraman et al [18]	U	U	H	U	L	L	H	High
Kazemzadeh et al [19]	L	L	L	L	L	L	L	Low
Love et al [20]	U	U	H	H	L	L	U	High
Malherbe [21]	H	U	H	H	L	L	H	Very high
Marquez et al [22]	H	L	H	H	L	L	L	High
Nath et al [23]	U	U	L	U	L	L	L	Moderate
Nothnagel and Aslam [24]	L	L	L	H	L	L	L	Moderate
Papachristou et al [25]	U	L	H	H	L	L	L	High
Poli et al [26]	U	L	H	H	L	L	L	High
Yang et al [27]	H	U	U	L	L	L	L	High
Yu et al [28]	H	H	L	H	L	L	L	High
Zhu et al [29]	H	U	U	U	L	L	L	High

a QUADAS-2: Quality Assessment of Diagnostic Accuracy Studies 2.

b RoB: risk of bias.

c D1: patient selection.

d D2: index test.

e D3: reference standard.

f D4: flow and timing.

g App: applicability concerns.

h U: unclear risk.

i L: low risk.

j H: high risk.

**Figure 8.**
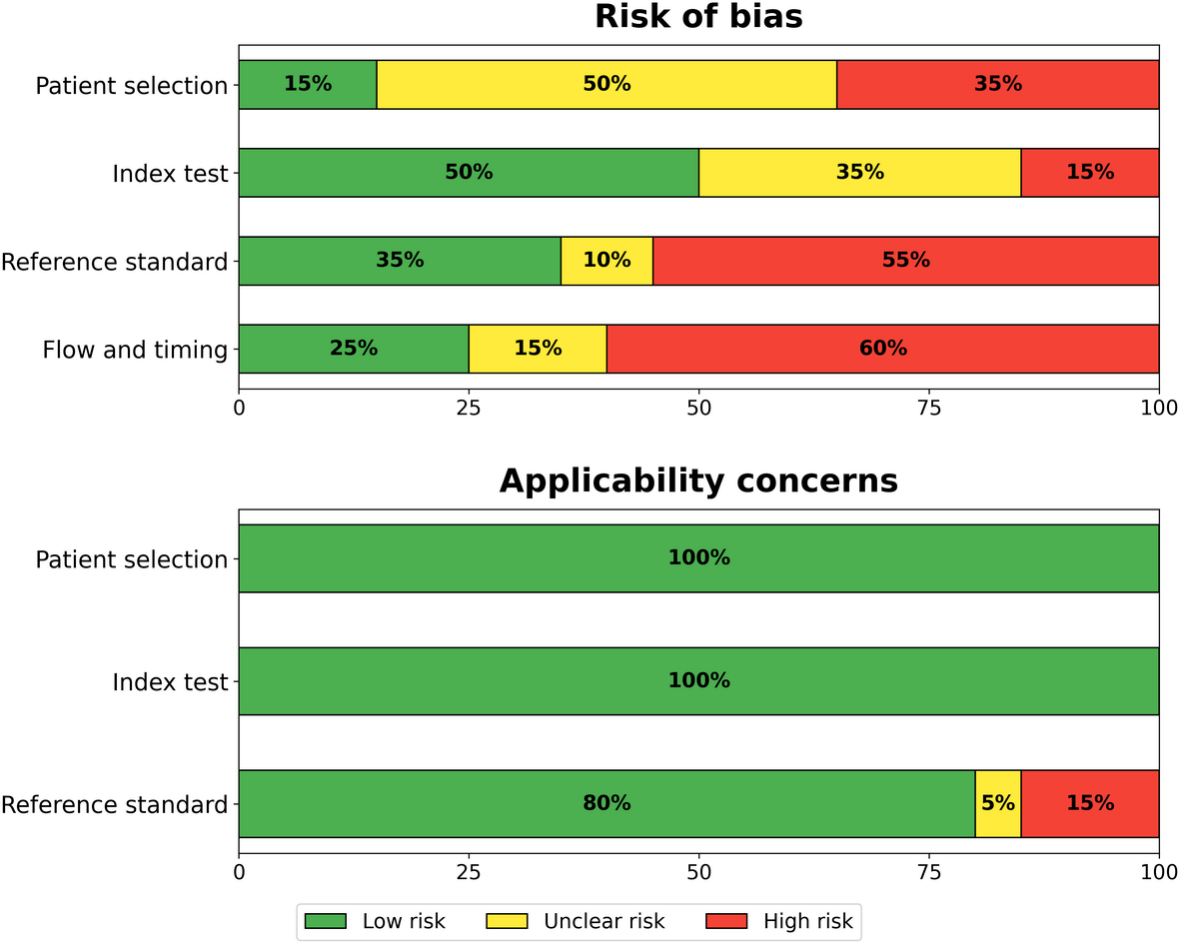
Summarizing the proportion of studies at low, unclear, and high risk of bias across all QUADAS-2 domains. QUADAS-2: Quality Assessment of Diagnostic Accuracy Studies 2.

Domain-level assessment revealed that the index test domain (domain 2) was the strongest, with 50% (10/20) of studies classified as being at low risk, reflecting reduced bias from automated AI interpretation. Patient selection (domain 1) revealed a 50% (10/20) unclear risk due to inadequate enrollment reporting, with high-risk studies relying on convenience sampling or case-control designs. The reference standard (domain 3) indicated a 55% (11/20) high risk, primarily due to differential verification bias; notably, Malherbe [21] did not have a reference standard. Flow and timing (domain 4) showed a 60% (12/20) high-risk rating, reflecting substantial exclusions and inadequate patient accounting.

Applicability concerns were generally low across all domains: patient selection 100% (20/20), index test 100% (20/20), and reference standard 80% (16/20) low concern, suggesting that, despite concerns about RoB, the studied populations and AI systems are relevant to the review question.

The most common methodological issues were verification bias (14/20, 70%) of studies, where only positive cases received the reference standard or substantial proportions were unverified; selection bias (10/20, 50%), involving nonconsecutive or convenience sampling; post hoc threshold optimization (6/20, 30%), potentially inflating performance estimates; and high exclusion rates (6/20, 30%), with some studies excluding over 20% of enrolled patients.

### Sensitivity Analysis

Given that 70% (14/20) of studies were rated as having a high or very high RoB, we conducted a sensitivity analysis restricted to studies with a low-to-moderate RoB (6/20, 30%; detailed in Multimedia Appendix 5). Sensitivity ranges narrowed from 62.5%-100% to 87%-100% (median 93.6%, IQR 87%-98% vs 90%, IQR 88%-95.7%), and specificity ranges narrowed from 28.1%-100% to 54%-96.5% (median 90.6%, IQR 74.5%-96.7% vs 85%, IQR 62%-93.5%). Median AUC remained stable at 0.91 (IQR 0.82-0.96) for the full cohort versus 0.91 (IQR 0.86-0.96) for the quality-restricted subset. These narrower ranges reflected elimination of outliers rather than systematic inflation in lower-quality studies, suggesting primary diagnostic accuracy conclusions are robust to quality concerns.

### Reporting Bias Assessment

A qualitative assessment of reporting bias revealed several limitations (Multimedia Appendix 6). The comprehensiveness of the literature search was moderate: while 4 major databases (PubMed, Scopus, IEEE Xplore, and Web of Science) were systematically searched, gray literature, conference proceedings, preprint servers, and trial registries were not included, potentially missing unpublished studies with null or negative findings. Language restrictions to English-only studies may have excluded relevant evidence from non-English sources.

Publication bias indicators suggested moderate concern. Commercial AI systems predominated (12/20, 60%), potentially introducing bias in favor of favorable results from vendor-sponsored research. Small-study effects were evident: small studies (n<200, 5/20, 25%) demonstrated narrower and higher performance ranges (sensitivity 85.7%‐100%, specificity 71.1%‐100%) compared to extensive studies (n>1000, 8/20, 40%; sensitivity 62.5%‐98%, specificity 28.1%‐97.6%), consistent with preferential publication of favorable results from smaller studies.

Selective outcome reporting was moderate. Core sensitivity and specificity metrics were well reported (18/20, 90%, and 17/20, 85% of studies, respectively), but secondary metrics showed substantial gaps: AUC (10/20, 50%), PPV (10/20, 50%), NPV (10/20, 50%), sensitivity 95% CI (13/20, 65%), and specificity 95% CI (12/20, 60%). A total of 1 study reported no diagnostic performance metrics. However, the narrative synthesis approach and substantial heterogeneity limit the impact of these biases on review conclusions compared to meta-analytic approaches.

### Certainty of Evidence

GRADE assessment revealed very low certainty in diagnostic accuracy estimates (Table 9). Starting from a high certainty level per GRADE guidance, the evidence was downgraded to 6 levels across all domains. The RoB was rated as of grave concern (2-level downgrade), given that 70% (14/20) of studies were at high or very high risk, with verification bias (14/20, 70%), selection bias (10/20, 50%), and post hoc threshold optimization (6/20, 30%) being the predominant concerns. Indirectness was rated as a serious concern (1-level downgrade) due to heterogeneity across 12 conditions, 6 modalities, and 18 AI systems. Inconsistency was rated as a serious concern, given the wide performance variation (sensitivity: 62.5%‐100%, specificity: 28.1%‐100%); even tuberculosis studies showed specificity ranging from 28.1% to 96.9%. Imprecision was rated a serious concern because 30% (6/20) of studies lacked CIs, and 25% (5/20) were small (n<200). Publication bias was rated as a serious concern due to the predominance of commercial systems (12/20, 60%) and small-study effects.

**Table 9.** GRADE^a^ certainty of evidence assessment.

Domain	Concern level	Rationale	Downgrade
Risk of bias	Very serious	70% (14/20) of studies at high or very high risk of bias, predominantly due to verification bias (14/20, 70%), selection bias (10/20, 50%), and post hoc threshold optimization (6/20, 30%). Reference standard (11/20, 55%, high risk) and flow or timing (12/20, 60%, high risk) were the weakest domains.	−2
Indirectness	Serious	Extreme heterogeneity across 12 conditions, 6 imaging modalities, and 18 AI systems limits applicability to specific clinical scenarios despite appropriate point-of-care contexts.	−1
Inconsistency	Serious	Wide performance variation (sensitivity 62.5%‐100%, specificity 28.1%‐100%); even within tuberculosis studies, specificity ranged 28.1%‐96.9%.	−1
Imprecision	Serious	30% of studies lack CIs, 15% report very wide CIs (>20 points), 25% are small studies (n<200).	−1
Publication bias	Serious	Commercial system predominance (12/20, 60%), gray literature not searched, small-study effects evident.	−1
Overall certainty	Very low (⊕◯◯◯)	Total downgrade of 6 levels from a high certainty starting point. True diagnostic performance may be substantially different from reported estimates.	−6

a GRADE: Grading of Recommendations, Assessment, Development, and Evaluation.

The very low certainty rating suggests that the proper diagnostic performance may differ substantially from the reported estimates. While median sensitivity (93.6%, IQR 87%-98%) and specificity (90.6%, IQR 74.5%-96.7%) appear promising, decision-makers should interpret these values cautiously, recognizing that real-world performance is likely to differ from the published results. Local validation remains essential before clinical adoption, and current evidence supports pilot implementation rather than widespread deployment without addressing methodological and heterogeneity concerns.

## Discussion

### Principal Findings

This systematic review of 20 studies evaluating AI in point-of-care imaging for clinical decision support reveals a rapidly evolving field, with 85% (17/20) of studies published since 2023 and approximately 78,000 patients enrolled across 15 countries. Five principal findings emerge with distinct implications for research, practice, and policy: (1) AI enables meaningful task-shifting from specialists to nonspecialist health care workers; (2) training requirements are remarkably minimal; (3) a profound gap exists between regulatory expectations for AI transparency and actual explainability implementation; (4) no studies measured patient-level health outcomes; and (5) the absence of cross-context validation undermines claims of global applicability.

This review provides the first systematic evidence synthesis demonstrating that AI enables meaningful task-shifting from specialists to nonspecialist health care workers across diverse point-of-care imaging applications. A total of 13 (65%) studies, 13/20 explicitly evaluated task-shifting, consistently showing that nonspecialists with AI assistance achieved diagnostic performance comparable to specialists. Key exemplars include Love et al [20] (100% sensitivity or specificity after 30 min of training), Avgerinos et al [10] and Nothnagel and Aslam [24] (100% DVT sensitivity with 1 hour training), and Nath et al [23] (technicians outperforming radiologists for tuberculosis detection). The consistency of this finding across diverse conditions—including deep vein thrombosis, breast masses, tuberculosis, and hip dysplasia—suggests the generalizability of the task-shifting paradigm, with policy implications for workforce planning, revision of training curricula, and regulatory frameworks for nonspecialist AI-assisted diagnostics.

This review also quantifies remarkably minimal training requirements for practical AI-assisted point-of-care imaging. The median training duration was only 1 hour (range 30 minutes to 6 months), with several studies achieving excellent performance after 30 minutes. Critically, no dose-response relationship was observed between training duration and diagnostic performance. This finding challenges traditional assumptions about extensive training requirements for medical imaging interpretation. The AI effectively serves as an “expert in the pocket,” enabling rapid deployment of diagnostic capabilities. The implications for scalability are profound, particularly in LMIC and LIC contexts, where traditional imaging training programs requiring months or years may be unnecessary when AI provides real-time guidance.

A critical finding concerns the gap between regulatory expectations for AI transparency and actual implementation of explainability. The XAI cascade revealed a progressive drop-off from 75% (15/20) nonmentioning (level 0) to only 10% (2/20) user presentation (level 2), with no studies reaching levels 3‐5—a field-wide gap requiring urgent attention. The findings suggest XAI is being treated as a development tool rather than a clinical communication mechanism. Regulatory compliance with the European Union AI Act and FDA guidance, as well as establishing clinical trust and appropriate AI usage, both depend on addressing this gap.

These findings have direct implications for physician autonomy—the capacity of clinicians to exercise independent judgment over diagnosis and treatment. The near absence of clinician-facing explainability (only 10% of studies) limits physicians’ ability to evaluate AI recommendations critically. When AI functions as opaque, physicians cannot meaningfully integrate AI outputs with their clinical reasoning; as Grote and Berens [30] argue, this uncertainty potentially undermines the epistemic authority of clinicians, risking either uncritical deference to algorithmic outputs or dismissal of potentially valuable decision support. Similarly, AI-enabled task-shifting, while extending diagnostic capacity, reshapes traditional boundaries of professional responsibility; physicians retain accountability for patient outcomes even when nonspecialists perform AI-assisted imaging. Future research should explicitly assess how AI implementation affects physician autonomy, including whether XAI restores clinician control and how task-shifting protocols maintain appropriate oversight structures.

The clinical impact pyramid revealed a substantial disconnect between technical validation and clinical benefit. While 15% (3/20) of studies reported technical accuracy alone, 65% (13/20) of studies progressed to process outcomes such as time to diagnosis and workflow efficiency. Although 20% (4/20) of studies documented clinical actions based on AI recommendations, no studies measured patient-level health outcomes or assessed the impact on the health system. Decision-makers cannot currently determine whether AI-assisted point-of-care imaging improves patient outcomes or represents a sound investment in health care. This gap is particularly critical in LMIC contexts, where resource allocation decisions require evidence of outcomes. The focus on diagnostic accuracy metrics reflects methodological traditions of diagnostic test evaluation, but leaves the fundamental question unanswered: Does AI-assisted point-of-care imaging help patients?

While 35% (7/20) of studies were conducted in LMIC or LIC settings, no studies validated AI systems across different resource contexts. Performance in LMIC and LIC settings was slightly lower (median sensitivity 88% versus 95.2% in HICs), but the absence of cross-context validation prevents conclusions about true generalizability. Before scaling AI systems to underserved populations, multicountry validation is essential to address differences in disease prevalence, imaging equipment, image quality, and patient populations.

These promising findings must be interpreted in light of the substantial methodological concerns detailed in the “Limitations of the Included Evidence” section below. With 70% (14/20) of studies at high or very high RoB, real-world performance may be lower than reported, and the GRADE assessment of very low certainty reflects appropriate caution. These findings must be contextualized within the existing literature to assess their novelty and significance.

### Comparison With Prior Work

This review addresses gaps not covered by existing systematic reviews in this domain. Kossoff et al [1] examined automated lung ultrasound analysis for pneumothorax detection; yet, their review was limited to a single modality and condition, without requiring point-of-care validation. Cold et al [2] focused on AI in bronchoscopy within specialist procedural settings rather than point-of-care contexts. Rambabu et al [3] evaluated AI for papilloedema detection using fundus photography; however, the included studies were predominantly retrospective and lacked clinical outcome assessment. Sunny et al [4] reviewed biomarker-integrated cytopathology for oral lesion detection, a laboratory-based methodology fundamentally distinct from point-of-care imaging.

Our review extends beyond these prior works in several critical dimensions. First, we provide the first cross-modality synthesis encompassing ultrasound, chest radiography, photography, fundus imaging, microscopy, and dermoscopy within point-of-care settings. Second, we developed and applied proposed frameworks—the XAI implementation cascade and clinical impact pyramid—that enable systematic identification of evidence gaps not previously characterized. Third, we provide the first quantitative characterization of task-shifting success and training requirements across point-of-care imaging applications.

The XAI and patient outcome gaps we identified are consistent with broader patterns in medical AI literature. Previous reviews of AI in radiology and pathology have similarly noted the predominance of diagnostic accuracy studies without clinical outcome assessment, suggesting this represents a field-wide methodological pattern requiring systematic attention rather than an isolated finding within point-of-care imaging.

### Implications

These findings carry important implications for clinical practice, research priorities, and health policy.

Clinicians considering AI-assisted point-of-care imaging should recognize both promise and limitations. Diagnostic accuracy data support cautious adoption for specific applications, particularly tuberculosis screening, for which multiple studies demonstrate consistent performance. However, local validation remains essential before deploying systems that have been validated in different contexts. Training programs should be competency-based rather than duration-based; evidence suggests that brief training can be sufficient when AI provides real-time guidance, but competency assessment remains essential. The AI serves as decision support rather than autonomous diagnostics, and clinical judgment remains critical, particularly when AI recommendations conflict with clinical findings. Health care organizations implementing AI-assisted imaging should document clinical actions and patient outcomes; real-world performance data will build the evidence base currently lacking and enable quality improvement.

Based on the identified evidence gaps, we make the following explicit recommendations for future research. XAI evaluation studies should assess not only implementation but also clinician understanding and the impact on decision-making, using mixed methods that include cognitive task analysis and decision quality metrics. Implementation research with patient outcomes as primary end points, using cluster-randomized trials to compare AI-assisted vs standard care pathways, is crucial for determining whether these systems enhance patient health. Multicountry and multicontext validation is needed before claims of global applicability can be substantiated, including sites across HIC, upper-middle-income country, LMIC, and LIC settings. Methodological improvements, including consecutive enrollment, complete verification, prespecified thresholds, and reporting per STARD-AI (Standards for Reporting of Diagnostic Accuracy Studies–Artificial Intelligence) guidelines, would substantially strengthen the evidence base. Additional priorities include cost-effectiveness analyses using standardized methods and health system perspectives, longitudinal monitoring frameworks for deployed systems that track performance drift and recalibration needs, and equity-focused analyses that explicitly address whether AI reduces or exacerbates health disparities.

Policy implications are equally substantial, with 5 key recommendations. First, regulatory frameworks should evolve beyond requiring XAI implementation to requiring evidence of XAI evaluation and effectiveness; approval pathways should assess whether explanations are understood and valuable rather than merely present. Second, funding agencies should shift priorities from additional diagnostic accuracy studies toward implementation research with patient outcome end points for conditions where technical performance is established. Third, health technology assessment bodies should require cross-context validation evidence before recommending the adoption of systems in settings different from those in which they were initially validated. Fourth, workforce policy should anticipate task-shifting implications, as evidence suggests nonspecialists can achieve specialist-level performance with minimal training, with implications for training curricula, scope of practice regulations, and workforce planning. Fifth, global health initiatives should invest in validation infrastructure for LMIC and LIC settings, where the current evidence base is insufficient to support confident deployment despite these settings having the greatest need.

### Strengths

This review has several methodological strengths. A comprehensive search across 4 databases (PubMed, Scopus, IEEE Xplore, and Web of Science) with rigorous dual-reviewer screening addressed the challenges inherent in identifying genuine point-of-care implementations amid aspirational claims. The strict requirement for actual point-of-care deployment or validation, rather than theoretical potential, ensures the included evidence reflects real-world implementation. Thorough QUADAS-2 quality assessment with prespecified synthesis rules, sensitivity analysis restricted to studies with low-to-moderate RoB (6/20), and GRADE certainty evaluation ensured transparent and reproducible evidence appraisal.

### Limitations of the Included Evidence

The evidence base has substantial limitations that temper interpretation of the generally favorable diagnostic accuracy findings. First, 70% (14/20) of studies carried a high or very high RoB, predominantly due to verification bias (14/20, 70%), where only screen-positive cases received reference standard confirmation, and selection bias (10/20, 50%), which involved convenience sampling rather than consecutive sampling. These methodological weaknesses likely inflate reported sensitivity and specificity estimates. Second, post hoc threshold optimization in 30% (6/20) of studies may have overfitted performance to specific datasets, limiting generalizability. Third, substantial clinical heterogeneity across 12 conditions, 6 imaging modalities, and 18 distinct AI systems limits the applicability of aggregate findings to any specific clinical scenario. Fourth, incomplete outcome reporting—with 30% (6/20) of studies lacking CIs and secondary metrics (AUC, PPV, and NPV) reported in only 50% (10/20) of the studies—impairs the precision of effect estimates. Fifth, the predominance of commercial systems (12/20, 60%) raises concerns about the selective publication of favorable results. Collectively, these evidence limitations suggest that actual diagnostic performance in routine clinical practice may be lower than reported estimates, and the GRADE assessment of very low certainty (⊕◯◯◯) reflects this uncertainty.

### Limitations of the Review Process

Several limitations of this review warrant consideration. First, restricting our search to English-language publications may have excluded relevant studies from non-English contexts; given that 35% (7/20) of included studies originated from LMIC/LIC settings where non-English publication is common, this restriction may have introduced selection bias that underrepresents the global evidence base. Second, excluding gray literature—including conference proceedings, preprints, and technical reports—may have introduced publication bias by omitting negative or null findings, though this decision prioritized peer-reviewed evidence quality for clinical decision-making. Third, encountering substantial heterogeneity across conditions, modalities, and AI systems precluded meta-analysis, limiting our ability to generate pooled effect estimates that would enable more precise conclusions. Fourth, concluding our search in November 2025 means that subsequently published studies are not captured, potentially missing recent methodological advances given the rapid evolution of this field.

### Conclusions

AI-assisted point-of-care imaging demonstrates promising diagnostic accuracy (median sensitivity 93.6%, IQR 87%-98%; median specificity 90.6%, IQR 74.5%-96.7%) and enables meaningful task shifting to nonspecialist operators with minimal training. These findings support cautious optimism regarding AI’s potential to democratize diagnostic capabilities in specialist-scarce settings.

However, 4 critical evidence gaps preclude confident recommendations for widespread adoption. The explainability gap is profound, with 75% (15/20) of studies not mentioning XAI and none evaluating whether explanations influenced clinical decisions, creating misalignment with regulatory requirements. The patient outcome gap is equally concerning, as no studies measured whether AI-assisted imaging actually improves patient health. The cross-context validation gap prevents conclusions about global applicability, and methodological quality concerns—with 70% (14/20) of studies at high or very high RoB—suggest reported performance may be optimistic.

The field requires urgent reorientation toward implementation research with patient outcome end points, rigorous XAI evaluation demonstrating clinical utility, and multicontext validation. Regulatory frameworks should require evidence of explainability effectiveness, not merely its presence. Only by systematically addressing these gaps can the field transition from promising technology demonstration to evidence-based clinical adoption that demonstrably improves patient outcomes.

## Supplementary material

10.2196/80928 Multimedia Appendix 1 Complete search strategies for all databases.

10.2196/80928 Multimedia Appendix 2 Studies excluded at full-text review with reasons.

10.2196/80928 Multimedia Appendix 3 Blank data extraction form template.

10.2196/80928 Multimedia Appendix 4 Detailed QUADAS-2 quality assessments. QUADAS-2: Quality Assessment of Diagnostic Accuracy Studies 2.

10.2196/80928 Multimedia Appendix 5 Sensitivity analysis data and results.

10.2196/80928 Multimedia Appendix 6 Reporting bias assessment details.

10.2196/80928 Multimedia Appendix 7 Complete extracted data for all 20 included studies.

10.2196/80928 Multimedia Appendix 8 GRADE certainty of evidence assessment. GRADE: Grading of Recommendations, Assessment, Development, and Evaluation.

10.2196/80928 Checklist 1 PRISMA 2020 checklist.

## Abbreviations

AI: artificial intelligence
AUC: area under the receiver operating characteristic curve
CDSS: clinical decision support systems
CE: conformité européenne
DVT: deep vein thrombosis
DVT: deep vein thrombosis
FDA: Food and Drug Administration
Grad-CAM: Gradient-weighted Class Activation Mapping
GRADE: Grading of Recommendations, Assessment, Development and Evaluation
HIC: high-income country
LIC: low-income country
LMIC: lower-middle-income country
MeSH: Medical Subject Headings
ML: machine learning
NPV: negative predictive value
PCC: Population, Concept, Context
PPV: positive predictive value
PRISMA: Preferred Reporting Items for Systematic Reviews and Meta-Analyses
QUADAS-2: Quality Assessment of Diagnostic Accuracy Studies 2
RoB: risk of bias
SAHPRA: South African Health Products Regulatory Authority
SHAP: Shapley Additive Explanations
STARD-AI: Standards for Reporting of Diagnostic Accuracy Studies–Artificial Intelligence
XAI: explainable artificial intelligence
