# Supplementary material for: AI in Point-of-Care Imaging for Clinical Decision Support: Systematic Review of Diagnostic Accuracy, Task-Shifting, and Explainability
Source: JMIR AI. 2026 Apr 27;5:e80928. doi: 10.2196/80928 (PMC13119389; doi:10.2196/80928)
Supplement: Multimedia Appendix 1 — Complete search strategies for all databases. [file ai-v5-e80928-s001.docx]

**Search Strategy**

Artificial Intelligence in Point-of-Care Imaging for Clinical Decision Support: Systematic Review of Diagnostic Accuracy, Task-Shifting, and Explainability

**Search Date:** November 24, 2025

**Summary**

| **Database** | **Results** |
| --- | --- |
| PubMed | 290 |
| Scopus | 993 |
| IEEE Xplore | 408 |
| Web of Science Core Collection | 422 |
| **Total** | **2113** |

**1. PubMed (290 results)**

("artificial intelligence"[MeSH Terms] OR "machine learning"[MeSH Terms] OR "deep learning"[MeSH Terms] OR "artificial intelligence"[Title/Abstract] OR "machine learning"[Title/Abstract] OR "deep learning"[Title/Abstract] OR "neural network*"[Title/Abstract] OR "convolutional neural network*"[Title/Abstract] OR ("CNN"[Title/Abstract] AND "convolutional"[Title/Abstract]) OR "computer vision"[Title/Abstract] OR ("transformer"[Title/Abstract] AND ("neural"[Title/Abstract] OR "attention"[Title/Abstract] OR "model"[Title/Abstract])) OR "generative ai"[Title/Abstract] OR "generative artificial intelligence"[Title/Abstract] OR "foundation model*"[Title/Abstract])

AND

("decision support techniques"[MeSH Terms] OR "decision support"[Title/Abstract] OR "clinical decision support"[Title/Abstract] OR "CDSS"[Title/Abstract] OR "computer-aided"[Title/Abstract] OR "computer-aided"[Title/Abstract] OR "CADe"[Title/Abstract] OR "CADx"[Title/Abstract] OR "diagnostic aid*"[Title/Abstract] OR "risk stratification"[Title/Abstract] OR "triage"[Title/Abstract])

AND

("diagnostic imaging"[MeSH Terms] OR "radiography"[MeSH Terms] OR "magnetic resonance imaging"[MeSH Terms] OR "ultrasonography"[MeSH Terms] OR "microscopy"[MeSH Terms] OR "medical imag*"[Title/Abstract] OR "clinical imag*"[Title/Abstract] OR "diagnostic imag*"[Title/Abstract] OR "ultrasound"[Title/Abstract] OR "ultrasonography"[Title/Abstract] OR "dermoscopy"[Title/Abstract] OR "dermatoscopy"[Title/Abstract] OR "clinical photograph*"[Title/Abstract] OR "fundus"[Title/Abstract] OR "retinal imag*"[Title/Abstract] OR "microscopy"[Title/Abstract] OR "microscopic imag*"[Title/Abstract] OR "histopathology"[Title/Abstract] OR "histopathologic*"[Title/Abstract] OR "cytology"[Title/Abstract] OR "pathology imag*"[Title/Abstract] OR "X-ray"[Title/Abstract] OR "radiograph*"[Title/Abstract] OR "ct scan"[Title/Abstract] OR "computed tomography"[Title/Abstract] OR "MRI"[Title/Abstract] OR "magnetic resonance"[Title/Abstract] OR "radiology"[Title/Abstract])

AND

("point of care systems"[MeSH Terms] OR "point of care testing"[MeSH Terms] OR "point-of-care"[Title/Abstract] OR "POC"[Title/Abstract] OR "POCUS"[Title/Abstract] OR "bedside"[Title/Abstract] OR ("handheld"[Title/Abstract] AND ("device*"[Title/Abstract] OR "ultrasound"[Title/Abstract] OR "imaging"[Title/Abstract])) OR ("portable"[Title/Abstract] AND ("device*"[Title/Abstract] OR "ultrasound"[Title/Abstract] OR "imaging"[Title/Abstract] OR "diagnostic*"[Title/Abstract])) OR "portable ultrasound"[Title/Abstract] OR ("mobile device*"[Title/Abstract] AND ("health"[Title/Abstract] OR "medical"[Title/Abstract] OR "diagnostic*"[Title/Abstract])) OR ("smartphone"[Title/Abstract] AND ("diagnostic*"[Title/Abstract] OR "imaging"[Title/Abstract] OR "medical"[Title/Abstract])) OR "mobile health"[Title/Abstract] OR "mHealth"[Title/Abstract] OR "low resource setting*"[Title/Abstract] OR "resource-limited"[Title/Abstract] OR "resource-constrained"[Title/Abstract] OR "on-site"[Title/Abstract] OR "decentralized"[Title/Abstract] OR "field diagnostic*"[Title/Abstract] OR "telemedicine"[Title/Abstract])

**2. Scopus (993 results)**

TITLE-ABS-KEY(("artificial intelligence" OR "machine learning" OR "deep learning" OR "neural network*" OR "convolutional neural network*" OR ("CNN" AND "convolutional") OR "computer vision" OR ("transformer" AND ("neural" OR "attention" OR "model")) OR "generative ai" OR "generative artificial intelligence" OR "foundation model*") AND ("decision support" OR "clinical decision support" OR "CDSS" OR "computer-aided" OR "computer aided" OR "CADe" OR "CADx" OR "diagnostic aid*" OR "risk stratification" OR "triage") AND ("medical imag*" OR "clinical imag*" OR "diagnostic imaging" OR "ultrasound" OR "ultrasonography" OR "dermoscopy" OR "dermatoscopy" OR "clinical photograph*" OR "fundus" OR "retinal imag*" OR "microscopy" OR "microscopic imag*" OR "histopathology" OR "histopathologic*" OR "cytology" OR "pathology imag*" OR "X-ray" OR "radiograph*" OR "ct scan" OR "computed tomography" OR "MRI" OR "magnetic resonance" OR "radiology") AND ("point-of-care" OR "POC" OR "POCUS" OR "bedside" OR (("handheld" OR "portable") AND ("device*" OR "ultrasound" OR "imaging" OR "diagnostic*")) OR "portable ultrasound" OR (("mobile device*" OR "smartphone") AND ("health" OR "medical" OR "diagnostic*" OR "imaging")) OR "mobile health" OR "mHealth" OR "low-resource setting*" OR "resource-limited" OR "resource-constrained" OR "on-site" OR "decentralized" OR "field diagnostic*" OR "telemedicine"))

**3. IEEE Xplore (408 results)**

((("artificial intelligence" OR "machine learning" OR "deep learning" OR "neural network" OR "neural networks" OR "convolutional neural network" OR "convolutional neural networks" OR ("CNN" AND "convolutional") OR "computer vision" OR ("transformer" AND ("neural" OR "attention" OR "model")) OR "generative ai" OR "generative artificial intelligence" OR "foundation model" OR "foundation models") AND ("decision support" OR "clinical decision support" OR "CDSS" OR "computer-aided" OR "computer aided" OR "CADe" OR "CADx" OR "diagnostic aid" OR "diagnostic aids" OR "risk stratification" OR "triage") AND ("medical imag*" OR "clinical imag*" OR "diagnostic imaging" OR "ultrasound" OR "ultrasonography" OR "dermoscopy" OR "dermatoscopy" OR "clinical photograph*" OR "fundus" OR "retinal imag*" OR "microscopy" OR "microscopic imaging" OR "histopathology" OR "histopathologic" OR "cytology" OR "pathology imaging" OR "X-ray" OR "radiograph" OR "radiography" OR "ct scan" OR "computed tomography" OR "MRI" OR "magnetic resonance" OR "radiology") AND ("point-of-care" OR "POC" OR "POCUS" OR "bedside" OR (("handheld" OR "portable") AND ("device" OR "devices" OR "ultrasound" OR "imaging" OR "diagnostic" OR "diagnostics")) OR "portable ultrasound" OR (("mobile device" OR "mobile devices" OR "smartphone" OR "smartphones") AND ("health" OR "medical" OR "diagnostic" OR "diagnostics" OR "imaging")) OR "mobile health" OR "mHealth" OR "low-resource" OR "resource-limited" OR "resource-constrained" OR "on-site" OR "decentralized" OR "field diagnostic" OR "field diagnostics" OR "telemedicine")))

**4. Web of Science Core Collection (422 results)**

TS=(("artificial intelligence" OR "machine learning" OR "deep learning" OR "neural network*" OR "convolutional neural network*" OR ("CNN" AND "convolutional") OR "computer vision" OR ("transformer*" AND ("neural" OR "attention" OR "model")) OR "generative ai" OR "generative artificial intelligence" OR "foundation model*") AND ("decision support" OR "clinical decision support" OR "CDSS" OR "computer-aided" OR "computer aided" OR "CADe" OR "CADx" OR "diagnostic aid*" OR "risk stratification" OR "triage") AND ("medical imag*" OR "clinical imag*" OR "diagnostic imaging" OR "ultrasound" OR "ultrasonography" OR "dermoscopy" OR "dermatoscopy" OR "clinical photograph*" OR "fundus" OR "retinal imag*" OR "microscopy" OR "microscopic imag*" OR "histopathology" OR "histopathologic*" OR "cytology" OR "pathology imag*" OR "X-ray" OR "radiograph*" OR "ct scan" OR "computed tomography" OR "MRI" OR "magnetic resonance" OR "radiology") AND ("point-of-care" OR "POC" OR "POCUS" OR "bedside" OR (("handheld" OR "portable") AND ("device*" OR "ultrasound" OR "imaging" OR "diagnostic*")) OR "portable ultrasound" OR (("mobile device*" OR "smartphone*") AND ("health" OR "medical" OR "diagnostic*" OR "imaging")) OR "mobile health" OR "mHealth" OR "low-resource setting*" OR "resource-limited" OR "resource-constrained" OR "on-site" OR "decentralized" OR "field diagnostic*" OR "telemedicine"))

**Search Concepts**

The search strategy covers four main concept groups combined with AND:

**1. AI/ML Technology:** artificial intelligence, machine learning, deep learning, neural networks, CNNs, computer vision, transformers, generative AI, foundation models

**2. Clinical Decision Support:** decision support systems, CDSS, computer-aided detection/diagnosis (CADe/CADx), diagnostic aids, risk stratification, triage

**3. Medical Imaging Modalities:** ultrasound, dermoscopy, fundus/retinal imaging, microscopy, histopathology, cytology, X-ray, CT, MRI, radiology

**4. Point-of-Care Context:** point-of-care, bedside, handheld/portable devices, smartphone diagnostics, mHealth, low-resource settings, telemedicine
