# Supplementary material for: AI in Point-of-Care Imaging for Clinical Decision Support: Systematic Review of Diagnostic Accuracy, Task-Shifting, and Explainability
Source: JMIR AI. 2026 Apr 27;5:e80928. doi: 10.2196/80928 (PMC13119389; doi:10.2196/80928)
Supplement: Multimedia Appendix 2 — Studies excluded at full-text review with reasons. [file ai-v5-e80928-s002.docx]

**Studies Excluded at Full-Text Review**

Artificial Intelligence in Point-of-Care Imaging for Clinical Decision Support: Systematic Review of Diagnostic Accuracy, Task-Shifting, and Explainability

**Overview**

This supplementary material provides a representative sample of studies that progressed to full-text review but were ultimately excluded, with specific reasons for exclusion, in accordance with PRISMA 2020 Item 16b. These represent borderline studies that met many inclusion criteria but failed on specific requirements related to point-of-care (POC) deployment, study design, or clinical decision support output.

**Exclusion Reason Codes**

| **Code** | **Description** |
| --- | --- |
| E5 | Wrong imaging modality (not POC-capable medical imaging) |
| E6 | Specialist setting or operators (not primary care/community) |
| E7 | Technical output only (image quality, not clinical diagnosis) |
| E11 | Device/algorithm development without clinical deployment |
| E14 | Tertiary/specialist center (not primary care or community setting) |
| E15 | Retrospective design (AI applied to historical data, not prospective validation) |
| RE1 | Aspirational POC (POC mentioned as goal but not demonstrated) |

**Summary of Exclusions**

| **Reason** | **N** | **%** |
| --- | --- | --- |
| Retrospective design (E15) | 10 | 33.3% |
| Device/algorithm development only (E11) | 8 | 26.7% |
| Aspirational POC (RE1) | 5 | 16.7% |
| Specialist setting/operators (E6) | 3 | 10.0% |
| Tertiary/specialist center (E14) | 2 | 6.7% |
| Technical output only (E7) | 1 | 3.3% |
| Wrong imaging modality (E5) | 1 | 3.3% |
| Total | 30 | 100% |

**Table S5.1: Representative Sample of Studies Excluded at Full-Text Review**

| **#** | **Study** | **Year** | **Exclusion Code** | **Reason for Exclusion** |
| --- | --- | --- | --- | --- |
| 1 | Bai Y et al., Cancer Cytopathol | 2025 | E11 | Algorithm development phase; clinical deployment planned as future work |
| 2 | Boyle JA et al., Diabetes Technol Ther | 2024 | E15 | Retrospective accuracy comparison despite POC-capable device |
| 3 | Brenes D et al., Gynecol Oncol | 2024 | E11 | Device development phase; deployment described as future work |
| 4 | Chen G et al., Intensive Care Med | 2021 | E11 | Algorithm development with cross-validation; not clinical deployment |
| 5 | Crockett BG et al., J Am Soc Echocardiogr | 2022 | E15 | Retrospective model validation despite genuine POC setting |
| 6 | Dadon Z et al., Eur Heart J Digit Health | 2023 | E14 | Tertiary center cardiology service with specialist operators |
| 7 | Dadon Z et al., JACC Cardiovasc Imaging | 2024 | E6 | Specialist cardiology department at tertiary center |
| 8 | Duan H et al., EBioMedicine | 2021 | E11 | Algorithm development; mobile app is aspirational future work |
| 9 | Faierstein K et al., Intensive Care Med | 2024 | E15 | Retrospective secondary analysis of prospective cohort |
| 10 | Felgueiras J et al., Procedia Comput Sci | 2018 | E11 | Device prototype development without clinical deployment |
| 11 | Hartline C et al., Mil Med | 2025 | E11 | Device development and training assessment focus |
| 12 | Hu Z et al., Sci Rep | 2021 | E11 | Algorithm development and device validation focus |
| 13 | James BL et al., Biomed Opt Express | 2021 | E11 | Device validation rather than clinical deployment |
| 14 | Liao H et al., Eye | 2022 | E15 | Retrospective AI analysis of existing clinical images |
| 15 | Malherbe K, Ultrasound Med Biol | 2025 | E14 | Tertiary breast clinic with specialist operators |
| 16 | Marsden PJ et al., Br J Dermatol | 2023 | E6 | Secondary care dermatology with specialist operators |
| 17 | Marwaha JS et al., Genet Med | 2021 | E6 | Specialist genetics clinic with clinical geneticists |
| 18 | Moore CL et al., Ultrasound J | 2022 | E15 | Retrospective algorithm validation against expert sonologists |
| 19 | Motie-Shirazi M et al., Nat Med | 2025 | E7 | AI output is signal quality, not clinical diagnosis |
| 20 | Okada K et al., Int J Tuberc Lung Dis | 2024 | E15 | AI retrospectively applied to evaluate applicability |
| 21 | Rhee SY et al., Diabetes Care | 2025 | E15 | Retrospective secondary analysis of clinical trial data |
| 22 | Ridhi et al., BMC Infect Dis | 2024 | E15 | Retrospective comparison of AI on different input formats |
| 23 | Schilpzand MG et al., BJOG | 2022 | RE1 | Health care worker deployment is aspirational future work |
| 24 | Shamsunder S et al., JCO Glob Oncol | 2023 | RE1 | Tertiary hospital with specialist supervision |
| 25 | Sunny S et al., Oral Oncol | 2019 | RE1 | Field health worker deployment described as future work |
| 26 | Talathi S et al., J Cancer Res Clin Oncol | 2023 | RE1 | Hospital department setting, not community/primary care |
| 27 | Udrea A et al., J Eur Acad Dermatol Venereol | 2020 | E15 | Retrospective algorithm validation on user database |
| 28 | Uthoff RD et al., Sci Rep | 2018 | RE1 | Conducted at specialist institution despite POC device |
| 29 | Wang T et al., Otolaryngol Head Neck Surg | 2022 | E15 | Retrospective accuracy comparison via online survey |
| 30 | Yu S et al., Ann Neurol | 2020 | E5 | Facial video/speech is not medical imaging |

**Notes**

These 30 studies represent a representative sample of borderline exclusions that came closest to meeting inclusion criteria for this systematic review. All passed initial eligibility screening (appropriate study type, language, year, AI/ML component, imaging modality) and demonstrated POC-relevant elements such as POC-capable devices, prospective data collection, non-specialist operators, or resource-limited settings. However, each failed on specific POC deployment criteria, typically because (1) AI was validated retrospectively rather than deployed prospectively, (2) POC-capable devices were used in specialist rather than primary care/community settings, or (3) the study focused on device/algorithm development rather than clinical implementation.
