# Supplementary material for: AI in Point-of-Care Imaging for Clinical Decision Support: Systematic Review of Diagnostic Accuracy, Task-Shifting, and Explainability
Source: JMIR AI. 2026 Apr 27;5:e80928. doi: 10.2196/80928 (PMC13119389; doi:10.2196/80928)
Supplement: Multimedia Appendix 3 — Blank data extraction form template. [file ai-v5-e80928-s003.docx]

**Data Extraction Form**

Artificial Intelligence in Point-of-Care Imaging for Clinical Decision Support: Systematic Review of Diagnostic Accuracy, Task-Shifting, and Explainability

**Section 1: Study Registry**

| **Field** | **Definition** | **Value** |
| --- | --- | --- |
| Study_ID | Unique identifier (FirstAuthor_Year format) |  |
| First_Author | Last name of first author |  |
| Year | Publication year |  |
| Country | Country where study was conducted |  |
| Continent | Geographic continent (Africa / Asia / Europe / North America / South America / Oceania) |  |
| Income_Level | World Bank classification (HIC / UMIC / LMIC / LIC / NR) |  |
| Condition | Target condition being diagnosed |  |
| Setting | Clinical setting description |  |
| N_Analyzed | Number of participants/samples in final analysis |  |

**Section 2: Study Design**

| **Field** | **Definition** | **Value** |
| --- | --- | --- |
| Study_ID | Study identifier |  |
| Study_Design | Study design as described by authors |  |
| Prospective_Retro | Temporal direction (Prospective / Retrospective / Case-control / NR) |  |
| Single_Multi | Number of sites (Single / Multi) |  |
| N_Sites | Number of study sites |  |
| Enrollment_Period | Time period of patient enrollment |  |
| Enrollment_Method | How participants were selected (Consecutive / Convenience / Random / NR) |  |
| Sample_Size | Total number enrolled or analyzed |  |
| Sample_Unit | Unit of analysis (Patients / Images / Lesions / Eyes / Samples / Other) |  |

**Section 3: Population**

| **Field** | **Definition** | **Value** |
| --- | --- | --- |
| Study_ID | Study identifier |  |
| Target_Condition | Primary condition being diagnosed/screened |  |
| Presentation | How patients presented (Symptomatic / Screening / Mixed / NR) |  |
| Age_Mean | Mean or median age of participants (years) |  |
| Age_Range | Age range of participants |  |
| Male_Percent | Percentage male participants (%) |  |
| N_Analyzed | Final number in analysis |  |
| Prevalence | Disease prevalence in study population (%) |  |

**Section 4: POC Setting**

| **Field** | **Definition** | **Value** |
| --- | --- | --- |
| Study_ID | Study identifier |  |
| Facility_Type | Type of health care facility (Primary care / ED / Hospital / Community / Mobile / Home / Other) |  |
| Geographic | Urban/rural classification (Urban / Rural / Mixed / NR) |  |
| Resource_Context | Resource availability description |  |
| Healthcare_System | Public/private system (Public / Private / Mixed / NR) |  |
| Income_Level | Country income classification (HIC / UMIC / LMIC / LIC) |  |
| POC_Justification | Authors' rationale for POC need (quote or paraphrase) |  |
| Specialist_Avail | Specialist availability described |  |

**Section 5: Operator Characteristics**

| **Field** | **Definition** | **Value** |
| --- | --- | --- |
| Study_ID | Study identifier |  |
| Operator_Primary | Primary operator type (Specialist / PCP / Nurse / CHW / Technician / Student / Other) |  |
| Operator_All | All operator types listed |  |
| N_Operators | Number of operators |  |
| Prior_Experience | Prior imaging/diagnostic experience (Yes / No / Mixed / NR) |  |
| Training_Provided | Was training provided for study? (Yes / No / NR) |  |
| Training_Duration | Duration of training (text description) |  |
| Training_Hours | Training duration in hours (number) |  |
| Training_Format | Format of training (Didactic / Hands-on / App-based / Video / Mixed / NR) |  |
| Competency_Assessed | Was operator competency assessed? (Yes / No / NR) |  |

**Section 6: AI System**

| **Field** | **Definition** | **Value** |
| --- | --- | --- |
| Study_ID | Study identifier |  |
| AI_Name | Name of AI system |  |
| AI_Developer | Developer/manufacturer |  |
| Architecture_Category | General AI architecture type (Deep learning / Machine learning / Hybrid / NR) |  |
| Architecture_Specific | Specific architecture (eg, CNN, ResNet, VGG) |  |
| Framework | Software framework (TensorFlow / PyTorch / Other / NR) |  |
| Training_Data_Size | Size of AI training dataset |  |
| Validation_Type | Type of validation (Internal / External / Both / NR) |  |
| Commercial_Status | Development status (Commercial / Research / Open source) |  |
| Regulatory_Status | Regulatory approval (FDA / CE / Other / None / NR) |  |
| Availability | How system can be accessed (Commercial / Research only / Open source / NR) |  |

**Section 7: AI Output**

| **Field** | **Definition** | **Value** |
| --- | --- | --- |
| Study_ID | Study identifier |  |
| Output_Type | Type of AI output (Binary / Multiclass / Continuous / Localization / Other) |  |
| Output_Classes | Classification categories |  |
| Threshold | Decision threshold used |  |
| Threshold_Derivation | How threshold was determined (Pre-specified / Optimized / Manufacturer / NR) |  |
| Clinical_Action | Recommended clinical action |  |
| CDSS_Integration | How AI integrates with clinical workflow |  |
| Processing_Time | Time to generate result |  |
| Real_Time | Real-time processing capability (Yes / No / NR) |  |
| Offline_Capable | Works without internet (Yes / No / NR) |  |
| Hardware | Hardware requirements |  |

**Section 8: Imaging**

| **Field** | **Definition** | **Value** |
| --- | --- | --- |
| Study_ID | Study identifier |  |
| Modality_Primary | Primary imaging modality (Ultrasound / X-ray / Photography / Fundus / Microscopy / Other) |  |
| Modality_Subtype | Specific imaging technique |  |
| Manufacturer | Device manufacturer |  |
| Model | Device model name |  |
| Device_Type | Portability (Handheld / Portable / Cart-based / Smartphone / Fixed / NR) |  |
| Cost_USD | Device cost in USD |  |
| Image_Acquisition_By | Who acquired images |  |
| Protocol_Standardized | Was imaging protocol standardized? (Yes / No / NR) |  |

**Section 9: Reference Standard**

| **Field** | **Definition** | **Value** |
| --- | --- | --- |
| Study_ID | Study identifier |  |
| Reference_Type | Type of reference standard |  |
| Reference_Details | Description of reference standard |  |
| Reference_Expert | Who performed/interpreted reference standard |  |
| Ref_Blinding | Was reference standard blinded to index test? (Yes / No / Unclear / NR) |  |
| Ref_Timing | Timing between index test and reference standard |  |
| Index_Blinding | Was index test blinded to reference standard? (Yes / No / Unclear / NR) |  |
| Verification_Bias | Evidence of verification bias? (Yes / No / Unclear) |  |
| Verification_Percent | Percentage receiving reference standard (%) |  |

**Section 10: Diagnostic Performance**

| **Field** | **Definition** | **Value** |
| --- | --- | --- |
| Study_ID | Study identifier |  |
| Sensitivity | True positive rate (%) |  |
| Sens_CI | 95% confidence interval for sensitivity |  |
| Specificity | True negative rate (%) |  |
| Spec_CI | 95% confidence interval for specificity |  |
| AUC | Area under ROC curve (0-1) |  |
| AUC_CI | 95% confidence interval for AUC |  |
| PPV | Positive predictive value (%) |  |
| NPV | Negative predictive value (%) |  |
| Accuracy | Overall accuracy (%) |  |
| TP | True positives (count) |  |
| FP | False positives (count) |  |
| TN | True negatives (count) |  |
| FN | False negatives (count) |  |

**Section 11: Comparator**

| **Field** | **Definition** | **Value** |
| --- | --- | --- |
| Study_ID | Study identifier |  |
| Comparator_Included | Was a comparator included? (Yes / No) |  |
| Comparator_Type | Type of comparator (Specialist / Non-specialist / Standard care / Other AI / None) |  |
| Comparator_Desc | Description of comparator |  |
| Comp_Sensitivity | Comparator sensitivity (%) |  |
| Comp_Specificity | Comparator specificity (%) |  |
| Comp_AUC | Comparator AUC |  |
| Statistical_Test | Statistical test used for comparison |  |
| AI_vs_Comparator | Result of comparison (AI superior / AI equivalent / AI inferior / Mixed / NR) |  |

**Section 12: Explainability (XAI)**

| **Field** | **Definition** | **Value** |
| --- | --- | --- |
| Study_ID | Study identifier |  |
| XAI_Mentioned | Is explainability mentioned? (Yes / No) |  |
| XAI_Terms | Terms used to describe XAI |  |
| Heatmap | Heatmap visualization used? (Yes / No / NR) |  |
| Attention | Attention mechanism described? (Yes / No / NR) |  |
| GradCAM | Grad-CAM used? (Yes / No / NR) |  |
| SHAP | SHAP used? (Yes / No / NR) |  |
| LIME | LIME used? (Yes / No / NR) |  |
| Other_XAI | Other XAI technique |  |
| XAI_Output_Type | Type of XAI output |  |
| Shown_Clinicians | Were explanations shown to clinical users? (Yes / No / NR) |  |
| Understanding_Assessed | Was user understanding assessed? (Yes / No / NR) |  |
| Decision_Impact | Was decision impact evaluated? (Yes / No / NR) |  |
| XAI_Cascade_Level | XAI implementation level (0-5, see definitions below) |  |

**XAI Cascade Level Definitions**

| **Level** | **Definition** |
| --- | --- |
| 0 | Not mentioned — No mention of explainability in paper |
| 1 | Mentioned/Implemented — XAI mentioned or implemented during development but not shown to users |
| 2 | Shown to users — XAI explanations displayed to clinical users during operation |
| 3 | Understanding assessed — Study evaluated whether users understood the explanations |
| 4 | Decision impact evaluated — Study measured whether XAI influenced clinical decisions |
| 5 | Full evaluation — Complete XAI evaluation including impact on patient outcomes |

**Section 13: Clinical Outcomes**

| **Field** | **Definition** | **Value** |
| --- | --- | --- |
| Study_ID | Study identifier |  |
| Outcomes_Beyond_Accuracy | Were outcomes beyond diagnostic accuracy reported? (Yes / No) |  |
| Referrals_Doc | Were referral patterns documented? (Yes / No / NR) |  |
| N_Referred | Number of patients referred |  |
| Time_to_Diagnosis | Was time to diagnosis reported? (Yes / No / NR) |  |
| Time_Value | Time to diagnosis value |  |
| Cost_Analysis | Was cost analysis performed? (Yes / No / NR) |  |
| Cost_Savings | Cost savings reported |  |
| Patient_Outcomes | Were patient health outcomes measured? (Yes / No / NR) |  |
| Workflow_Impact | Workflow impact described |  |
| User_Satisfaction | Was user satisfaction assessed? (Yes / No / NR) |  |
| Barriers | Implementation barriers identified |  |
| Facilitators | Implementation facilitators identified |  |
| Clinical_Impact_Level | Clinical impact level (0-5, see definitions below) |  |

**Clinical Impact Pyramid Level Definitions**

| **Level** | **Definition** |
| --- | --- |
| 0 | Technical accuracy only — Sensitivity, specificity, AUC reported; no workflow data |
| 1 | Process outcomes — Time to diagnosis, workflow efficiency, referral rates |
| 2 | Clinical actions — Treatment decisions, referral completion, clinical interventions |
| 3 | Patient outcomes — Mortality, morbidity, quality of life, patient-reported outcomes |
| 4 | Health system impact — Cost-effectiveness, resource utilization, system efficiency |
| 5 | Population health — Disease incidence/prevalence changes, population screening outcomes |

**Section 14: Limitations**

| **Field** | **Definition** | **Value** |
| --- | --- | --- |
| Study_ID | Study identifier |  |
| Author_Limitations | Limitations stated by authors |  |
| Sample_Size_Limit | Sample size limitation noted? (Yes / No) |  |
| Selection_Bias_Limit | Selection bias limitation noted? (Yes / No) |  |
| Single_Site_Limit | Single site limitation noted? (Yes / No) |  |
| Generalizability_Limit | Generalizability limitation noted? (Yes / No) |  |
| Ref_Standard_Limit | Reference standard limitation noted? (Yes / No) |  |
| Reviewer_Limitations | Additional limitations identified by reviewer |  |

**Section 15: Task-Shifting**

| **Field** | **Definition** | **Value** |
| --- | --- | --- |
| Study_ID | Study identifier |  |
| Task_From | Original task performer (eg, Specialist) |  |
| Task_To | New task performer with AI (eg, Nurse) |  |
| AI_Role | Role of AI in task-shifting (Autonomous / Decision support / Triage / NR) |  |
| Training_Required | Training required for task-shifted operators |  |
| Performance_Achieved | Performance achieved by task-shifted operators |  |
| Task_Shift_Quote | Quote describing task-shifting |  |

**Section 16: Integration**

| **Field** | **Definition** | **Value** |
| --- | --- | --- |
| Study_ID | Study identifier |  |
| AI_Imaging_Match | Does AI match imaging modality requirements? (Yes / No / Partial / NR) |  |
| AI_POC_Suitability | Is AI suitable for POC use? (Yes / No / Partial / NR) |  |
| AI_Hardware_Compatible | Is AI compatible with available hardware? (Yes / No / NR) |  |
| Integration_Model | Model of clinical integration |  |
| Integration_Success | Was integration successful? (Yes / No / Partial / NR) |  |
| Pathway_Change | Changes to clinical pathway described |  |

**Section 17: QUADAS-2 Summary**

| **Field** | **Definition** | **Value** |
| --- | --- | --- |
| Study_ID | Study identifier |  |
| D1_RoB | Domain 1 (Patient Selection) - Risk of Bias (Low / High / Unclear) |  |
| D1_App | Domain 1 - Applicability Concern (Low / High / Unclear) |  |
| D2_RoB | Domain 2 (Index Test) - Risk of Bias (Low / High / Unclear) |  |
| D2_App | Domain 2 - Applicability Concern (Low / High / Unclear) |  |
| D3_RoB | Domain 3 (Reference Standard) - Risk of Bias (Low / High / Unclear) |  |
| D3_App | Domain 3 - Applicability Concern (Low / High / Unclear) |  |
| D4_RoB | Domain 4 (Flow and Timing) - Risk of Bias (Low / High / Unclear) |  |
| Overall_Risk | Overall risk of bias (Low / Low-Moderate / Moderate / High / Very High) |  |
| Key_Bias_Issues | Summary of key bias concerns |  |

**QUADAS-2 Domain Definitions**

Domain 1 - Patient Selection: Was a consecutive or random sample enrolled? Was a case-control design avoided? Did the study avoid inappropriate exclusions?

Domain 2 - Index Test: Were index test results interpreted without knowledge of reference standard? Was the threshold pre-specified?

Domain 3 - Reference Standard: Is the reference standard likely to correctly classify the condition? Were reference standard results interpreted without knowledge of index test?

Domain 4 - Flow and Timing: Was there appropriate interval between index test and reference standard? Did all patients receive the same reference standard? Were all patients included in analysis?

**Overall Risk of Bias Classification**

| **Rating** | **Criteria** |
| --- | --- |
| Low | All domains rated low risk |
| Low-Moderate | One domain unclear, all others low |
| Moderate | One domain high risk OR multiple domains unclear |
| High | Two or more domains high risk |
| Very High | Three or more domains high risk OR critical methodological flaw |

**Section 18: Derived Metrics**

| **Field** | **Definition** | **Value** |
| --- | --- | --- |
| Study_ID | Study identifier |  |
| XAI_Cascade_Level | Assigned XAI cascade level (0-5) |  |
| Clinical_Impact_Level | Assigned clinical impact level (0-5) |  |
| Task_Shifting_Present | Evidence of task-shifting to non-specialists (Yes / No) |  |
| External_Validation | Was external validation performed? (Yes / No) |  |
| LMIC_Study | Study conducted in LMIC or LIC? (Yes / No) |  |

*END OF DATA EXTRACTION FORM*

*This form should be completed for each included study. Completed forms constitute the raw extracted data for the systematic review.*
