# Supplementary material for: AI in Point-of-Care Imaging for Clinical Decision Support: Systematic Review of Diagnostic Accuracy, Task-Shifting, and Explainability
Source: JMIR AI. 2026 Apr 27;5:e80928. doi: 10.2196/80928 (PMC13119389; doi:10.2196/80928)
Supplement: Multimedia Appendix 4 — Detailed QUADAS-2 quality assessments. QUADAS-2: Quality Assessment of Diagnostic Accuracy Studies 2. [file ai-v5-e80928-s004.docx]

QUADAS-2

**Avgerinos et al. 2025**Novel Artificial Intelligence Guided Non-expert Compression Ultrasound Deep Vein Thrombosis Diagnostic Pathway May Reduce Vascular Laboratory Venous Testing
*Study ID: Avgerinos_2025 | Assessment Date: 2025-11-28*

____________________________________________________________________________________________________

# Phase 1: State the Review Question

*Patients (setting, intended use of index test, presentation, prior testing):*

| Patients presenting to emergency department with suspected deep vein thrombosis (DVT) requiring diagnostic evaluation. Setting: tertiary hospital ED. Intended use: AI-guided point-of-care compression ultrasound as a triage/screening tool for proximal DVT performed by non-specialists with remote radiologist interpretation. Presentation: clinical suspicion of DVT (e.g., leg swelling, pain). Prior testing: D-dimer testing where available. |
| --- |

*Index test(s):*

| ThinkSono Guidance system (CE Class IIb medical device) paired with Clarius HD3 L7 handheld ultrasound. AI-guided two-point compression ultrasound protocol performed by non-expert operators (1-hour training) with remote radiologist interpretation via cloud platform. Binary classification: compressible vs. incompressible vessels. |
| --- |

*Reference standard and target condition:*

| Reference standard: Full leg duplex ultrasound (DUS) performed by institutional radiologist, including compression ultrasound throughout upper and lower leg plus Doppler ultrasound. For patients with negative index test AND normal D-dimers: 2-month clinical follow-up via medical record review and phone contact. Target condition: Proximal deep vein thrombosis (DVT) in common femoral, femoral, profunda femoral, or popliteal veins. |
| --- |

# Phase 2: Draw a Flow Diagram for the Primary Study

| PATIENT FLOW DIAGRAM  Patients with suspected DVT presenting to ED (n = 59 consented)  ↓ Excluded: 2 withdrew consent, 4 incomplete scans (n = 6)  ↓ ThinkSono AI-guided scan performed (n = 53 analyzed)  ↓  ├─→ COMPRESSIBLE (n = 45)  │ ↓  │ D-dimer testing  │ ↓  │ ├─→ Normal D-dimer (n = 17)  │ │ ↓  │ │ No DUS - Clinical follow-up at 2 months  │ │ (15/17 contacted; 5 unreachable, 2 died)  │ │  │ └─→ Elevated/unavailable D-dimer (n = 28)  │ ↓  │ DUS performed same day → 2 false positives  │  └─→ INCOMPRESSIBLE (n = 8)  ↓  DUS performed same day → All 8 confirmed DVT (6 true positives)  FINAL ANALYSIS (n = 53) • Total DUS performed: 36/53 (68%) • Total DVT cases: 6/53 (11.3% prevalence) • Sensitivity: 100% (6/6) • Specificity: 95.7% (45/47) |
| --- |

# Phase 3: Risk of Bias and Applicability Judgments

*QUADAS-2 is structured so that 4 key domains are each rated in terms of the risk of bias and the concern regarding applicability to the research question (as defined above). Each key domain has a set of signalling questions to help reach the judgments regarding bias and applicability.*

# DOMAIN 1: PATIENT SELECTION

## A. Risk of Bias

**Describe methods of patient selection:**This single-center prospective study enrolled patients presenting to the Emergency Department (ED) of Attikon University Hospital (Athens, Greece) with suspected deep vein thrombosis (DVT) over a 12-month period. Patients presenting with suspected DVT indicated for duplex ultrasound (DUS) were prospectively enrolled. Initially, 59 patients were consented; 2 withdrew and 4 scans could not be completed due to logistical issues (operator availability or hardware battery levels), leaving 53 patients with complete ThinkSono scans included in the analysis (p.520, Figure 2). Ethics approval was granted by the institutional review board (approval C24/07-04-2021), and all patients gave written informed consent (p.518).

Page reference: (p.518, p.520)

| Signaling Question | Answer |
| --- | --- |
| ❖ Was a consecutive or random sample of patients enrolled? | UNCLEAR |
| ❖ Was a case-control design avoided? | YES |
| ❖ Did the study avoid inappropriate exclusions? | YES |

**Could the selection of patients have introduced bias?: RISK: UNCLEAR**

**Justification:** While the study used a prospective design and enrolled patients presenting with suspected DVT to a real-world ED setting, the enrollment method was not explicitly stated as consecutive or random. The authors explicitly acknowledge that "recruitment and scanning of patients was limited by the scheduling availability of the non-expert operators, which may have led to unintentional selection bias" (p.521-522). This suggests convenience sampling based on when trained operators were available rather than consecutive or random enrollment. The study also lacks demographic data on the broader ED population, preventing assessment of whether enrolled patients were representative. This introduces potential selection bias, as patients enrolled may systematically differ from those who presented when operators were unavailable.

## B. Concerns regarding applicability

**Describe included patients (prior testing, presentation, intended use of index test and setting):**The study population matches the review scope well: patients presenting to an ED with suspected DVT requiring evaluation—a typical point-of-care scenario. The setting (tertiary hospital ED in Athens, Greece), population characteristics (mean age 55.6 ± 18.3 years, 45% female, mean BMI 32.9 ± 16.2 kg/m²), and clinical context (suspected DVT indicated for ultrasound evaluation) are representative of real-world POC imaging applications (p.518, p.520). The prevalence of DVT (6/53 = 11.3%) is consistent with clinical populations evaluated for suspected DVT.

**Is there concern that the included patients do not match the review question?: CONCERN: LOW**

**Justification:** The included patients appropriately match the review question.

# DOMAIN 2: INDEX TEST(S)

*If more than one index test was used, please complete for each test.*

## A. Risk of Bias

**Describe the index test and how it was conducted and interpreted:**The index test was AI-guided two-region proximal DVT compression ultrasound using the ThinkSono Guidance system (ThinkSono GmbH, Potsdam, Germany), a CE Class IIb medical device software paired with a Clarius HD3 L7 handheld ultrasound device (p.518). Three non-specialists (one clinical researcher, two vascular surgery residents) with no prior ultrasound training performed examinations after a 1-hour training session covering app use and scanning techniques. After submitting two scans for review and technique approval, data collection began (p.518). The software guided users through a two-point compression ultrasound (without duplex), instructing compressions in the groin areas (common femoral vein, femoral and profunda femoral veins) and popliteal fossa (popliteal vein and proximal trifurcation). Four 10-second cine loops were recorded per examination and uploaded to a cloud platform for remote review by on-call radiologists (p.518). Images were rated using the ACEP image quality scale (1-5, with ≥3 constituting adequate quality) and assessed as "Compressible," "Incompressible," or "Indeterminate" (p.518-519, Table 1).

Page reference: (p.518-519)

| Signaling Question | Answer |
| --- | --- |
| ❖ Were the index test results interpreted without knowledge of the results of the reference standard? | YES |
| ❖ If a threshold was used, was it pre-specified? | YES |

**Could the conduct or interpretation of the index test have introduced bias?: RISK: LOW**

**Justification:** The index test was conducted prospectively before the reference standard, ensuring blinding of test interpretation. The AI-guided scan was performed first, and the radiologist review of uploaded images occurred remotely via cloud dashboard before any decision about DUS was made (p.518). The ThinkSono Guidance system used established vessel compressibility criteria—a standard diagnostic approach for DVT—rather than a novel threshold that could have been optimized on this dataset. The system is CE Class IIb certified with documented training and validation (p.518). The ACEP image quality scale used for rating is a pre-existing standardized tool (Table 1, p.519).

## B. Concerns regarding applicability

**Is there concern that the index test, its conduct, or interpretation differ from the review question?: CONCERN: LOW**

**Justification:** The index test represents AI-guided POC ultrasound as intended for clinical use—non-expert operators using a handheld device with real-time AI guidance and remote expert review. This matches the review scope of AI-based clinical decision support using point-of-care imaging. The workflow (ED setting, minimally trained operators, remote radiologist interpretation) reflects realistic POC deployment scenarios (p.518). The ThinkSono Guidance system is a commercially available CE-certified device used as designed.

# DOMAIN 3: REFERENCE STANDARD

## A. Risk of Bias

**Describe the reference standard and how it was conducted and interpreted:**The reference standard pathway was dependent on the index test and D-dimer results, creating a differential verification design. For patients with positive AI-guided scans OR elevated D-dimers: Full leg duplex ultrasound (DUS) was performed by the institution's on-call radiologist, including compression throughout upper and lower leg plus Doppler ultrasound, with results recorded in a signed written report (p.519). For patients with negative AI-guided scans AND normal D-dimers: Patients were discharged without DUS (17/53 patients, 32%) (p.520). These patients had medical records checked two months after study conclusion, and 15 were contacted by phone to confirm no subsequent DVT; 5 could not be reached, 1 died of heart failure at 2 months, and 1 died of liver failure at 4 months (p.520). Two consultant radiologists performed image quality review and interpretation of the ThinkSono scans (51 reviews by radiologist 1, 2 reviews by radiologist 2) (p.520).

Page reference: (p.519-520)

| Signaling Question | Answer |
| --- | --- |
| ❖ Is the reference standard likely to correctly classify the target condition? | YES |
| ❖ Were the reference standard results interpreted without knowledge of the results of the index test? | NO |

**Could the reference standard, its conduct, or its interpretation have introduced bias?: RISK: HIGH**

**Justification:** This domain has HIGH risk of bias due to incorporation and review bias. First, the same radiologists who interpreted the ThinkSono images (index test) also performed the confirmatory DUS (reference standard) on the same day (p.519-520), knowing these patients were flagged as having suspicious findings. This creates incorporation bias and review bias—the radiologists were not blinded to the index test result when conducting DUS. Second, the reference standard decision was directly influenced by the index test result: only patients with incompressible findings or elevated D-dimers received DUS, while those with compressible findings and normal D-dimers were discharged without DUS (differential verification). This design systematically biases results toward high NPV because false negatives can only be detected if patients become symptomatic during the 2-month follow-up period.

## B. Concerns regarding applicability

**Is there concern that the target condition as defined by the reference standard does not match the review question?: CONCERN: LOW**

**Justification:** The target condition (proximal DVT) and reference standard (duplex ultrasound) are appropriate and align with the review question. DUS is the gold standard for DVT diagnosis. However, the differential verification design (using clinical follow-up instead of DUS for 32% of patients) introduces methodological concerns about bias rather than applicability.

# DOMAIN 4: FLOW AND TIMING

## A. Risk of Bias

**Describe any patients who did not receive the index test(s) and/or reference standard or who were excluded from the 2x2 table (refer to flow diagram):**Six patients were excluded after enrollment: 2 withdrew consent and 4 had incomplete scans due to logistical issues (operator availability or hardware battery levels) (p.520, Figure 2). These 6 patients did not receive complete index test evaluation and were excluded before analysis. Of the 53 patients with complete ThinkSono scans, 17 (32%) did not receive the reference standard DUS. These patients had negative ThinkSono scans AND normal D-dimers, and were discharged without DUS, verified instead by 2-month clinical follow-up (p.520).

**Describe the time interval and any interventions between index test(s) and reference standard:**For patients who received DUS (36/53, 68%), the interval between index test and reference standard was the same day. The ThinkSono scan was performed first, followed by D-dimer testing if available, then DUS on the same day for those with positive findings (p.518-519). For patients who did not receive DUS (17/53, 32%), follow-up occurred at 2 months post-enrollment via medical record review and phone contact (p.520). The study design created two different verification pathways based on index test results: immediate DUS for positive cases versus delayed clinical follow-up for negative cases.

| Signaling Question | Answer |
| --- | --- |
| ❖ Was there an appropriate interval between index test(s) and reference standard? | YES |
| ❖ Did all patients receive a reference standard? | NO |
| ❖ Did patients receive the same reference standard? | NO |
| ❖ Were all patients included in the analysis? | YES |

**Could the patient flow have introduced bias?: RISK: HIGH**

**Justification:** This domain has HIGH risk of bias due to partial and differential verification. First, 17/53 patients (32%) did not receive the gold standard reference (DUS) but were instead verified by clinical follow-up at 2 months (p.520). This creates verification bias because any subclinical or subsequently symptomatic DVT in this group could have been missed, particularly given that 5/15 patients could not be reached by phone, and 2/17 died of other causes before the 2-month follow-up window (p.520). Second, the reference standard was determined by the index test result itself (differential verification): patients negative on ThinkSono received a weaker reference standard (clinical follow-up), while those positive or with elevated D-dimers received DUS. This design systematically favors high NPV because false negatives can only be detected if patients become symptomatic during follow-up. The 2-month follow-up window may be insufficient to capture all missed DVTs.

QUADAS-2

**Berg et al. 2023**Toward AI-supported US Triage of Women with Palpable Breast Lumps in a Low-Resource Setting *Study ID: Berg_2023 | Assessment Date: 2025-11-28*

____________________________________________________________________________________________________

# Phase 1: State the Review Question

*Patients (setting, intended use of index test, presentation, prior testing):*

| Women aged >18 years with palpable breast lumps presenting to government hospitals in low-resource settings in Mexico. Setting: Hospital Valentin Gomez Farias in Zapopan, Jalisco, Mexico (primary site) and Hospital General de Tijuana (9 patients). Intended use: AI-assisted triage using portable point-of-care ultrasound to identify women requiring biopsy/specialist referral vs those who can safely be monitored. Presentation: Women with at least one palpable breast lump awaiting diagnostic assessment. Prior testing: None specified. Population: 300 Hispanic women (mean age 50.0 ± 12.5 years, range 18-92) with 758 breast masses, 56 malignancies (7.4% prevalence at lesion level). |
| --- |

*Index test(s):*

| Koios DS version 3.x AI software (Koios Medical) applied to breast ultrasound images. Two US systems used: (1) Portable low-cost Vscan Extend (GE HealthCare) with 2.9-cm, 8.0-3.3 MHz linear transducer—images obtained by either specialist radiologist (first 376 women) or minimally trained non-physician research coordinators (subsequent 102 women with 30-minute training); (2) Standard-of-care Hi-Vision Avius (Hitachi Medical) with 5-cm, 13-5 MHz linear transducer—images by specialist radiologist. Deidentified paired images processed automatically by Koios DS without human intervention. AI outputs: benign, probably benign, suspicious, malignant (corresponding to BI-RADS 1/2, 3, 4A/4B, 4C/5) plus quantitative malignancy risk score (0-1). AI trained on >700,000 images from 40 clinical sites. |
| --- |

*Reference standard and target condition:*

| Reference standard: Differential verification design. For suspicious lesions (BI-RADS 4 or 5 by radiologist on standard-of-care US): Histopathologic examination via core needle biopsy or surgical excision. For benign-appearing lesions (BI-RADS 1, 2, or 3): Radiologist assessment alone without histopathologic confirmation. This creates verification bias as only radiologist-suspected malignancies received gold standard verification. Note: 116 BI-RADS 3 masses without follow-up were excluded from analysis. Target condition: Breast malignancy (invasive ductal carcinoma or ductal carcinoma in situ). Final cohort: 56 malignancies among 758 masses (7.4%). |
| --- |

# Phase 2: Draw a Flow Diagram for the Primary Study

| PATIENT FLOW DIAGRAM  Initial cohort: 1216 breast masses in 478 women (Enrolled Dec 2017 - May 2021, suspended May 2019 - Dec 2020)  ↓ EXCLUSIONS: ├─→ 9 masses from Tijuana site (incomplete data) ├─→ 50 lesions unsuitable for Koios DS (36 lymph nodes, 11 skin, 2 normal variant, 1 scar) ├─→ 4 data mismatches ├─→ 44 lesions >29mm (exceeded portable US field of view) ├─→ 116 BI-RADS 3 masses without follow-up ├─→ 3 redundant lesions ├─→ 1 missing pathology ├─→ 1 non-breast malignancy (sarcoma) └─→ 230 missing Hitachi images Total excluded: 458 masses (38% of initial cohort)  ↓ FINAL LESION-LEVEL ANALYSIS 758 masses in 300 women  ↓ Both index tests performed: ├─→ Portable US (Vscan Extend) + Koios DS AI │ • 376 women: Images by radiologist │ • 102 women: Images by minimally trained coordinators │ └─→ Standard US (Hitachi Avius) + Koios DS AI  • All images by radiologist  ↓ Reference Standard (Differential Verification): ├─→ BI-RADS 4/5 (Suspicious) → Histopathology │ Result: 56 malignancies confirmed │ (50 invasive ductal carcinoma, 6 DCIS) │ └─→ BI-RADS 1/2/3 (Benign/Probably benign) → Radiologist assessment only  Result: 702 masses classified as benign  (No histopathologic verification - verification bias)  PARTICIPANT-LEVEL ANALYSIS 300 index masses in 300 women 49 malignancies (16.3% prevalence) (Additional 458 multiple lesions excluded from this analysis)  KEY PERFORMANCE (Lesion-level): • AI on radiologist-obtained portable images: AUC 0.98, Sensitivity 97%, Specificity 52% • AI on minimally trained images: AUC 0.78, Sensitivity 86%, Specificity 33% • Standard US + AI: AUC 0.95-0.98, Sensitivity 95-100%, Specificity 52-80% |
| --- |

# Phase 3: Risk of Bias and Applicability Judgments

*QUADAS-2 is structured so that 4 key domains are each rated in terms of the risk of bias and the concern regarding applicability to the research question (as defined above). Each key domain has a set of signalling questions to help reach the judgments regarding bias and applicability.*

# DOMAIN 1: PATIENT SELECTION

## A. Risk of Bias

**Describe methods of patient selection:**This prospective multicenter study sought to enroll 500 women older than 18 years, each with at least one palpable breast lump (p.2). Women were recruited from December 11, 2017, through May 21, 2021, at two centers in Mexico: Hospital Valentin Gomez Farias in Zapopan, Jalisco (primary site), and Hospital General de Tijuana (closed after enrolling 9 patients due to inability to provide supporting data) (p.2). Enrollment was suspended from May 14, 2019, through December 12, 2020, first to amend the protocol to allow minimally trained personnel to obtain images, then due to COVID-19 (p.2). Initially, 1216 breast masses in 478 Hispanic women were documented, with 126 malignant (10.4%). After exclusions detailed in Figure 1 (p.3), the final lesion-level analysis included 758 masses from 300 women, with 56 malignancies (7.4%). For participant-level analysis, 300 index masses in 300 women were analyzed, including 49 malignancies (16.3%) (p.3-4).

Page reference: (p.2-4, Figure 1)

| Signaling Question | Answer |
| --- | --- |
| ❖ Was a consecutive or random sample of patients enrolled? | UNCLEAR |
| ❖ Was a case-control design avoided? | YES |
| ❖ Did the study avoid inappropriate exclusions? | NO |

**Could the selection of patients have introduced bias?: RISK: HIGH**

**Justification:** Of 1216 initially documented lesions in 478 women, only 758 lesions in 300 women (62% of lesions, 63% of patients) were included in final analysis. The exclusion of 116 BI-RADS 3 masses without follow-up (p.3) is particularly concerning because these represent indeterminate lesions whose final diagnosis is unknown—excluding them may artificially improve apparent diagnostic performance by removing ambiguous cases. Additionally, the 230 missing Hitachi images represent data loss that could be non-random (p.3, Figure 1). The exclusion of lesions >29mm due to transducer field-of-view limitations (N=44) represents spectrum bias, as larger masses may have different characteristics. While enrollment appears prospective, the lack of explicit statement about consecutive enrollment and substantial post-hoc exclusions create selection bias risk.

## B. Concerns regarding applicability

**Describe included patients (prior testing, presentation, intended use of index test and setting):**The final analysis included 300 Hispanic women (mean age 50.0 years ± 12.5, range 18-92 years) with 758 breast masses presenting with palpable lumps to a government hospital in Jalisco, Mexico (p.3). Of 758 masses, 360 (47.5%) were palpable and 56 (7.4%) were malignant. The mean largest lesion diameter was 13 mm ± 8 (range 2-54 mm) (p.3). The setting was a low-resource context in Mexico, specifically intended to evaluate AI for triage in low- and middle-income countries (LMIC) where women with lumps may wait months for diagnostic assessment (p.1).

**Is there concern that the included patients do not match the review question?: CONCERN: LOW**

**Justification:** The study population closely matches the review question for AI-enabled clinical decision support using point-of-care imaging. Patients presented with palpable breast lumps at point of care in a low-resource setting. The study specifically evaluated portable US with AI for triage at point of care by minimally trained observers. The clinical context (LMIC, long wait times for specialist evaluation) represents an ideal application for POC AI-CDS. The Hispanic population in Mexico may differ somewhat from other global LMIC populations, but the clinical presentation and resource constraints are generalizable.

# DOMAIN 2: INDEX TEST(S)

*If more than one index test was used, please complete for each test.*

## A. Risk of Bias

**Describe the index test and how it was conducted and interpreted:**The index test was Koios DS version 3.x AI software (Koios Medical) applied to breast US images (p.2). Two US systems were used: (1) Portable low-cost Vscan Extend (GE HealthCare) with a 2.9-cm, 8.0-3.3 MHz linear-array transducer—orthogonal images with and without calipers obtained by either a specialist breast imaging radiologist (J.C.L.P., 5 years experience, first 376 women) or minimally trained non-physician research coordinators (subsequent 102 women trained with a validated 30-minute PowerPoint presentation) (p.2); (2) Standard-of-care (SOC) Hi-Vision Avius (Hitachi Medical) with a 5-cm, 13-5 MHz linear-array transducer—orthogonal images obtained by the specialist radiologist (p.2). Deidentified paired images from both systems were processed by Koios DS without human intervention (p.2). AI outputs were benign, probably benign, suspicious, and malignant (corresponding to BI-RADS 1/2, 3, 4A/4B, and 4C/5, respectively), plus a numeric quantitative score from 0-1 approximating relative malignancy risk (p.2-3). The AI software was trained on >700,000 images from 40 clinical sites representing 17 different US models (p.2-3).

Page reference: (p.2-3)

| Signaling Question | Answer |
| --- | --- |
| ❖ Were the index test results interpreted without knowledge of the results of the reference standard? | YES |
| ❖ If a threshold was used, was it pre-specified? | YES |

**Could the conduct or interpretation of the index test have introduced bias?: RISK: LOW**

**Justification:** The AI software (Koios DS) automatically processed deidentified US images "without human intervention" (p.2). The AI algorithm has no knowledge of reference standard results—it processes images independently based on image features. The AI classification thresholds (benign, probably benign, suspicious, malignant corresponding to BI-RADS categories) were inherent to the validated Koios DS software, which was trained on >700,000 images from 40 clinical sites prior to this study (p.2-3). The thresholds were not optimized on this study's data. The automated nature of AI interpretation eliminates review bias from knowledge of diagnosis.

## B. Concerns regarding applicability

**Is there concern that the index test, its conduct, or interpretation differ from the review question?: CONCERN: LOW**

**Justification:** The index test directly matches the review scope: AI-enabled clinical decision support using portable point-of-care ultrasound. The Koios DS AI is a commercially available, validated system used as intended. The study evaluated both expert-obtained and minimally trained observer-obtained images, reflecting realistic POC deployment scenarios. However, the authors note the AI was NOT trained on images from the Vscan Extend portable device used in this study (p.7), which may limit generalizability. Despite this limitation, the application (portable US with AI for breast mass triage in low-resource settings) is highly applicable to the review question.

# DOMAIN 3: REFERENCE STANDARD

## A. Risk of Bias

**Describe the reference standard and how it was conducted and interpreted:**Differential verification design was used. For lesions classified as BI-RADS 4 or 5 (suspicious/malignant) by the radiologist on standard-of-care US: histopathologic examination via core needle biopsy or surgical excision was performed (p.2). For lesions classified as BI-RADS 1, 2, or 3 (benign or probably benign): radiologist assessment alone served as the reference standard without histopathologic verification (p.2). This means the vast majority of the 702 benign-classified masses did NOT receive gold standard histopathologic confirmation. The radiologist's BI-RADS assessment was made on standard-of-care Hitachi US images before AI analysis of portable images, ensuring blinding of reference standard from index test. However, 116 BI-RADS 3 masses "without follow-up" were excluded from analysis entirely (p.3, Figure 1).

Page reference: (p.2-3)

| Signaling Question | Answer |
| --- | --- |
| ❖ Is the reference standard likely to correctly classify the target condition? | NO |
| ❖ Were the reference standard results interpreted without knowledge of the results of the index test? | YES |

**Could the reference standard, its conduct, or its interpretation have introduced bias?: RISK: HIGH**

**Justification:** Differential verification bias is present. Only radiologist-suspected malignancies (BI-RADS 4/5) received histopathologic confirmation; the majority of lesions classified as benign received radiologist assessment only without gold standard verification (p.2). This creates verification bias because false negatives cannot be detected in the benign-classified group. If even 1-2% of radiologist-assessed benign lesions were actually malignant (consistent with BI-RADS 3 category risk of 0-2%), this would add 7-14 missed cancers, substantially reducing true sensitivity from reported values. The authors themselves acknowledge: "The radiologist's performance in this study appears artificially high...in part because of lack of follow-up and exclusion of many masses assessed as negative, benign, or probably benign" (p.7). While the reference standard (histopathology) is appropriate when performed, its differential application based on radiologist suspicion introduces systematic bias.

## B. Concerns regarding applicability

**Is there concern that the target condition as defined by the reference standard does not match the review question?: CONCERN: LOW**

**Justification:** The target condition (breast malignancy) and reference standard (histopathology when performed) are appropriate and match the review question. Histopathologic examination is the gold standard for breast cancer diagnosis. The differential verification design introduces bias concerns but does not affect applicability of the target condition definition itself.

# DOMAIN 4: FLOW AND TIMING

## A. Risk of Bias

**Describe any patients who did not receive the index test(s) and/or reference standard or who were excluded from the 2x2 table (refer to flow diagram):**Substantial exclusions occurred. From 1216 initially documented lesions in 478 women, only 758 masses in 300 women were included in final analysis (p.3, Figure 1). Exclusions: 9 lesions from Tijuana (incomplete data), 50 lesions unsuitable for Koios DS (lymph nodes, skin lesions, etc.), 4 data mismatches, 44 lesions >29mm (field-of-view limitation), 116 BI-RADS 3 masses without follow-up, 3 redundant lesions, 1 missing pathology, 1 non-breast malignancy, and 230 missing Hitachi images. Additionally, 458 multiple lesions per participant (in 153 women including 7 malignancies) were excluded from participant-level analysis (p.3). The exclusion of 116 BI-RADS 3 masses is particularly problematic as these indeterminate cases were removed from analysis.

**Describe the time interval and any interventions between index test(s) and reference standard:**Both US systems (portable Vscan and standard Hitachi) were used during the same study visit to image breast masses (p.2). For suspicious lesions (BI-RADS 4/5), histopathologic confirmation followed within a reasonable timeframe via core needle biopsy or surgical excision. For benign-classified lesions (BI-RADS 1/2/3), no histopathologic follow-up was performed—radiologist assessment at the time of imaging served as the reference standard. This differential timing/verification creates two reference standard pathways: immediate pathology for suspicious lesions vs. no pathology for benign-classified lesions.

| Signaling Question | Answer |
| --- | --- |
| ❖ Was there an appropriate interval between index test(s) and reference standard? | YES |
| ❖ Did all patients receive a reference standard? | YES* |
| ❖ Did patients receive the same reference standard? | NO |
| ❖ Were all patients included in the analysis? | NO |

*All analyzed patients received *a* reference standard, but different types (histopathology vs. radiologist assessment).

**Could the patient flow have introduced bias?: RISK: HIGH**

**Justification:** High risk of bias from both differential verification and substantial exclusions. First, differential verification bias: patients with benign-appearing lesions received radiologist assessment only (not histopathology), while suspicious lesions received gold standard pathology (p.2). This systematically prevents detection of false negatives in the benign-classified group. Second, exclusion bias: 38% of initially documented lesions were excluded (458/1216), including 116 BI-RADS 3 indeterminate masses "without follow-up"—precisely the cases where diagnostic accuracy is most uncertain (p.3). These exclusions may artificially inflate reported performance. The authors acknowledge this limitation, noting the radiologist's "artificially high" performance due to "lack of follow-up and exclusion of many masses assessed as negative, benign, or probably benign" (p.7). These methodological issues substantially undermine the reliability of reported sensitivity (95-100%) and AUC (0.78-0.98) values.

# OVERALL ASSESSMENT SUMMARY

**Note: This study has been rated as VERY HIGH overall risk of bias due to multiple serious methodological limitations across 3 of 4 domains.**

| Domain | Signaling Questions | Risk of Bias | Applicability |
| --- | --- | --- | --- |
| D1: Patient Selection | 1.1 Unclear 1.2 Yes 1.3 No | HIGH | LOW |
| D2: Index Test | 2.1 Yes 2.2 Yes | LOW | LOW |
| D3: Reference Standard | 3.1 No 3.2 Yes | HIGH | LOW |
| D4: Flow and Timing | 4.1 Yes 4.2 Yes* 4.3 No 4.4 No | HIGH | N/A |

**Overall Risk of Bias: VERY HIGH**

**Justification:** This study has 3 domains rated HIGH for Risk of Bias (D1: Patient Selection, D3: Reference Standard, D4: Flow and Timing). The primary methodological issue is differential verification bias affecting both D3 and D4: only radiologist-suspected suspicious lesions received histopathologic confirmation, while the majority of masses were classified as benign based on radiologist assessment alone without gold standard verification. This is compounded by exclusion bias in D1 and D4: 38% of initially documented lesions were excluded, including 116 BI-RADS 3 masses "without follow-up"—precisely the indeterminate cases where diagnostic accuracy is most uncertain. The authors themselves acknowledge that "The radiologist's performance in this study appears artificially high...in part because of lack of follow-up and exclusion of many masses assessed as negative, benign, or probably benign" (p.7). These limitations mean reported sensitivity (95-100%) and AUC values (0.78-0.98) should be interpreted with extreme caution and may not reflect real-world performance.

**Overall Applicability Concern: LOW**

**Justification:** Despite severe methodological limitations, the study is highly applicable to the review question. The setting (low-resource government hospitals in Mexico), population (women with palpable breast lumps awaiting diagnostic assessment), index test (portable AI-enabled ultrasound performed by minimally trained observers), and target condition (breast malignancy requiring triage) directly align with POC AI-CDS applications. The clinical context—LMIC settings with long wait times for specialist evaluation—represents an ideal use case for AI-assisted triage. The evaluation of both expert and minimally trained operators reflects realistic POC deployment scenarios.

## Key Methodological Limitations

**1. Differential verification bias (HIGH impact):** Only 56/758 masses (7.4%) received histopathologic verification; 702 benign-classified masses had no gold standard confirmation, preventing detection of false negatives.
**2. Substantial exclusion bias (HIGH impact):** 458/1216 initially documented lesions (38%) excluded, including 116 BI-RADS 3 indeterminate masses and 230 missing images.
**3. Spectrum bias:** Exclusion of lesions >29mm (N=44), lymph nodes, and skin lesions limits generalizability.
**4. Operator-dependent performance:** AI performance on minimally trained observer images significantly worse (AUC 0.78, sensitivity 86%, specificity 33%) vs. radiologist images (AUC 0.98, sensitivity 97%, specificity 52%).
**5. AI training mismatch:** Koios DS was NOT trained on images from the Vscan Extend portable device used in this study, potentially explaining reduced specificity.
**6. Small malignancy sample:** Only 56 malignancies at lesion level (21 in minimally trained subgroup), limiting precision of sensitivity estimates.

QUADAS-2

**Cao et al. 2025**Performance of Computer-Aided Detection Software in Tuberculosis Case Finding in Township Health Centers in China
*Study ID: Cao_2025 | Assessment Date: 2025-11-28*

____________________________________________________________________________________________________

# Phase 1: State the Review Question

*Patients (setting, intended use of index test, presentation, prior testing):*

| Outpatients aged ≥15 years presenting for chest X-rays at 13 township health centers in Zhongmu County, Henan Province, China (June-December 2020). Setting: Primary care/township health centers (point-of-care). Intended use: TB screening and triage using AI-assisted chest radiography to identify patients requiring further investigation. Presentation: General outpatient population seeking chest X-ray evaluation. Population characteristics: 3,705 participants; 54.84% male, 28.72% aged ≥65 years, 27.96% with cough symptoms, 1.73% with prior TB history. Prior testing: None specified. TB prevalence: 76 active TB cases identified radiologically (2.05%). |
| --- |

*Index test(s):*

| JF CXR-1 v3.0 computer-aided detection (CAD) software developed by JF Healthcare (Nanchang, China) using deep learning technology. Trained on approximately 300,000 chest X-ray images from township-level hospitals across China. Digital chest X-rays uploaded to software, which calculated continuous abnormality scores from 0 (normal) to 1 (highly abnormal) for multiple conditions including active TB, prior TB, pneumonia, and lung nodule/mass. Developer-recommended threshold score of 0.5 used to classify images as positive or negative. AI interpretation performed independently before local radiologists gave final diagnosis after seeing AI results. |
| --- |

*Reference standard and target condition:*

| Reference standard: Expert panel consisting of one radiologist and two clinical experts with >10 years experience in chest X-ray reading and TB diagnosis. CRITICAL LIMITATION: Expert panel ONLY reviewed chest X-rays with discordant results between local radiologists and JF CXR-1 v3.0 (235/3,705 cases = 6.35%). Concordant results (3,470 cases = 93.65%) were assumed correct without expert verification. Positive criterion required unanimous agreement among all three panel members. NO microbiological confirmation (culture or molecular testing) was performed due to logistical/budgetary constraints. Target condition: Active tuberculosis as identified by radiological abnormalities suggestive of TB on chest X-ray (not microbiologically confirmed). Note: Reference standard is expert radiological interpretation, NOT WHO-recommended culture-based confirmation. |
| --- |

# Phase 2: Draw a Flow Diagram for the Primary Study

| PATIENT FLOW DIAGRAM  Prospectively collected chest X-rays (June - December 2020, 13 township health centers) (n = 4,222)  ↓ EXCLUSIONS (n = 517): ├─→ 212 patients aged <15 years └─→ 305 patients with incomplete JF CXR-1 v3.0 reading (7.6% of age-eligible)  Reason for incomplete readings NOT explained - potential selection bias  ↓ FINAL ANALYSIS COHORT (n = 3,705 participants)  ↓ ALL PATIENTS RECEIVED: ├─→ JF CXR-1 v3.0 CAD analysis (INDEX TEST) │ Threshold: 0.5 (developer-recommended) │ Output: Abnormality score 0-1 │ └─→ Local radiologist interpretation  ↓ REFERENCE STANDARD APPLICATION (Differential Verification):  ↓ ├─→ CONCORDANT RESULTS (n = 3,470 = 93.65%) │ Both CAD and local radiologist agree (both positive OR both negative) │ → Accepted as TRUE without expert panel review │ → NO VERIFICATION BY REFERENCE STANDARD │ └─→ DISCORDANT RESULTS (n = 235 = 6.35%)  CAD and local radiologist disagree  → Expert panel review (1 radiologist + 2 clinical experts)  → Unanimous decision required for positive diagnosis  → THIS IS THE ONLY GROUP VERIFIED BY REFERENCE STANDARD  CRITICAL ISSUE: Partial & Differential Verification Bias • Only 235/3,705 (6.35%) received expert panel review • 3,470/3,705 (93.65%) assumed correct based on concordance • Concordant errors (both CAD and radiologist wrong) cannot be detected • NO microbiological confirmation for any cases  REPORTED PERFORMANCE (Active TB): • JF CXR-1 v3.0: Sensitivity 92.11% (95% CI: 86.04-98.17%)  Specificity 94.54% (95% CI: 93.81-95.28%) • Local radiologists: Sensitivity 32.89% (95% CI: 22.33-43.46%)  Specificity 99.28% (95% CI: 99.01-99.56%) • 76 "active TB" cases identified (radiological, not microbiologically confirmed)  Note: Performance estimates likely inflated due to verification bias |
| --- |

# Phase 3: Risk of Bias and Applicability Judgments

*QUADAS-2 is structured so that 4 key domains are each rated in terms of the risk of bias and the concern regarding applicability to the research question (as defined above). Each key domain has a set of signalling questions to help reach the judgments regarding bias and applicability.*

# DOMAIN 1: PATIENT SELECTION

## A. Risk of Bias

**Describe methods of patient selection:**This prospective study conducted from June to December 2020 enrolled outpatients who visited radiology departments for chest X-rays at 13 township health centers in Zhongmu County, Henan Province, China (p.141). Inclusion criteria were: outpatients presenting for chest X-ray, aged 15 years or older, and voluntary participation (p.141). From 4,222 prospectively collected chest X-rays, 517 were excluded: 212 patients aged <15 years and 305 patients whose JF CXR-1 v3.0 reading was not completed (p.142-143, Figure 1). The final analysis included 3,705 participants. This represents a cohort design with consecutive enrollment of outpatients presenting for chest radiography.

Page reference: (p.141, p.142-143, Figure 1)

| Signaling Question | Answer |
| --- | --- |
| ❖ Was a consecutive or random sample of patients enrolled? | UNCLEAR |
| ❖ Was a case-control design avoided? | YES |
| ❖ Did the study avoid inappropriate exclusions? | NO |

**Could the selection of patients have introduced bias?: RISK: HIGH**

**Justification:** The exclusion of 305 participants (7.6% of age-eligible patients) due to incomplete JF CXR-1 v3.0 readings represents a significant methodological concern. The authors acknowledge this limitation, stating that "approximately 7.6% (305/4,010) of participants' chest X-rays were not successfully imported into the CAD system for reading, which might also introduce bias to the results" (p.146). Without understanding why these images failed to process, there is potential for systematic exclusion of certain image characteristics (e.g., poor quality images, unusual positioning, or atypical presentations) that could bias performance estimates upward. This represents potential selection bias that may inflate diagnostic accuracy.

## B. Concerns regarding applicability

**Describe included patients (prior testing, presentation, intended use of index test and setting):**The study population is highly relevant to the review question. Participants were outpatients at township health centers (primary care level) in rural China, which represents a true point-of-care setting. The population characteristics (54.84% male, 28.72% aged ≥65 years, 27.96% with cough symptoms, 1.73% with prior TB history) reflect a real-world primary care population in a TB-endemic region (p.144, Table 2). This closely matches the intended use case for AI-based TB screening at point of care in resource-limited settings.

**Is there concern that the included patients do not match the review question?: CONCERN: LOW**

**Justification:** The patient population is highly applicable - outpatients at primary care township health centers in a TB-endemic region seeking chest radiography, which is the exact target population for POC AI-assisted TB screening.

# DOMAIN 2: INDEX TEST(S)

*If more than one index test was used, please complete for each test.*

## A. Risk of Bias

**Describe the index test and how it was conducted and interpreted:**The index test was JF CXR-1 v3.0, a computer-aided detection (CAD) software developed by JF Healthcare (Nanchang, China) using deep learning technology trained on approximately 300,000 chest X-ray images from township-level hospitals across China (p.141). Digital chest X-rays were uploaded to the software, which calculated continuous abnormality scores ranging from 0 (normal) to 1 (highly abnormal) for multiple conditions including active TB, prior TB, pneumonia, and lung nodule/mass (p.141). A developer-recommended threshold score of 0.5 was used to classify images as positive or negative for each condition (p.141). The AI interpreted images independently before local radiologists gave their final diagnosis after seeing AI results, ensuring the AI assessment was not influenced by the reference standard determination (p.141, Figure 1).

Page reference: (p.141, Figure 1)

| Signaling Question | Answer |
| --- | --- |
| ❖ Were the index test results interpreted without knowledge of the results of the reference standard? | YES |
| ❖ If a threshold was used, was it pre-specified? | YES |

**Could the conduct or interpretation of the index test have introduced bias?: RISK: LOW**

**Justification:** The index test was conducted appropriately with adequate safeguards against bias. The AI software processed images automatically without knowledge of the reference standard result, eliminating review bias. The threshold of 0.5 was pre-specified by the developer and applied consistently, not optimized on the study data (p.141). The prospective design ensured the AI interpretation occurred before reference standard determination. While the authors note that "variation exists in AI performance across different contexts, and the threshold abnormality scores probably need to vary depending on the test population and needs" (p.146), the use of a manufacturer-recommended threshold rather than a study-optimized threshold reduces risk of overfitting.

## B. Concerns regarding applicability

**Is there concern that the index test, its conduct, or interpretation differ from the review question?: CONCERN: LOW**

**Justification:** The AI system (JF CXR-1 v3.0) was evaluated in its intended use setting - township health centers in China, the same environment the software was trained on (images from township-level hospitals). The workflow mimicked real-world implementation where the CAD system would assist local radiologists (p.141). The system was designed for TB screening/triage at primary care level, which aligns well with point-of-care clinical decision support. The use of standard threshold values without post-hoc optimization supports applicability to similar settings.

# DOMAIN 3: REFERENCE STANDARD

## A. Risk of Bias

**Describe the reference standard and how it was conducted and interpreted:**The reference standard was reading by a panel of experts composed of one radiologist and two clinical experts with more than 10 years of experience in chest X-ray reading and TB diagnosis (p.141). CRITICALLY, the expert panel only reviewed chest X-rays with inconsistent reading results between local radiologists' initial reading and JF CXR-1 v3.0 (p.141). A positive criterion required every panel member to give a positive diagnosis (p.141). The study explicitly did NOT use microbiological confirmation (culture or molecular testing) as the reference standard. The authors acknowledge: "due to logistical and budgetary constraints, we did not collect sputum samples from each participant for pathogen detection... Therefore, we used a panel of expert readings as the reference standard instead of using culture-based methods, which remain recommended by the WHO, as a reference standard for detecting TB" (p.146).

Page reference: (p.141, p.146)

| Signaling Question | Answer |
| --- | --- |
| ❖ Is the reference standard likely to correctly classify the target condition? | NO |
| ❖ Were the reference standard results interpreted without knowledge of the results of the index test? | NO |

**Could the reference standard, its conduct, or its interpretation have introduced bias?: RISK: HIGH**

**Justification:** This domain has multiple serious limitations. First, the reference standard was expert radiological interpretation, NOT microbiological confirmation. WHO recommends culture-based methods as the reference standard for TB detection (p.146). Chest X-ray interpretation alone, even by experts, cannot definitively diagnose active TB. Second, the expert panel only reviewed discordant cases (235/3,705 = 6.35%), meaning concordant cases were assumed correct without expert verification (p.141). This creates differential verification bias. Third, the expert panel explicitly knew they were reviewing "chest X-rays with inconsistent reading results between local radiologists' initial reading and JF CXR-1 v3.0" (p.141), introducing incorporation bias. The study measures agreement between AI and expert radiological interpretation, not AI's ability to detect true microbiologically confirmed TB.

## B. Concerns regarding applicability

**Is there concern that the target condition as defined by the reference standard does not match the review question?: CONCERN: HIGH**

**Justification:** The target condition as defined (radiological abnormalities suggestive of TB by expert interpretation) does NOT match the true clinical target condition (microbiologically confirmed active TB disease). The study measures AI performance against an imperfect radiological reference standard rather than against bacteriologically confirmed disease. This fundamental limitation means findings demonstrate AI-radiologist concordance rather than AI's ability to detect true TB. The 76 "active TB" cases represent radiological diagnoses, not confirmed disease.

# DOMAIN 4: FLOW AND TIMING

## A. Risk of Bias

**Describe any patients who did not receive the index test(s) and/or reference standard or who were excluded from the 2x2 table (refer to flow diagram):**Of 3,705 participants in final analysis, only 235 (6.35%) received expert panel review (the reference standard). The remaining 3,470 participants (93.65%) with concordant results between local radiologists and JF CXR-1 were assumed correct and did NOT receive expert panel verification (p.141, p.143). This represents massive partial verification bias where >93% of the cohort did not receive the reference standard. Concordant results (both positive or both negative by local radiologists and AI) were accepted without independent verification.

**Describe the time interval and any interventions between index test(s) and reference standard:**The AI analysis and local radiologist reading occurred during the same clinical encounter. For the 235 discordant cases, expert panel review followed within a reasonable timeframe. The timing interval was appropriate. However, the differential application of the reference standard (expert panel review only for discordant cases) creates two verification pathways: expert review for 6.35% vs. no expert review for 93.65%.

| Signaling Question | Answer |
| --- | --- |
| ❖ Was there an appropriate interval between index test(s) and reference standard? | YES |
| ❖ Did all patients receive a reference standard? | YES* |
| ❖ Did patients receive the same reference standard? | NO |
| ❖ Were all patients included in the analysis? | YES |

*Technically all received *a* reference, but 93.65% received assumed concordance (not expert panel).

**Could the patient flow have introduced bias?: RISK: HIGH**

**Justification:** Severe partial and differential verification bias is present. Only 235/3,705 participants (6.35%) received the reference standard (expert panel review); the remaining 3,470 (93.65%) with concordant results were assumed correct without expert verification (p.141, p.143). This systematically prevents detection of concordant errors—if both local radiologists and AI missed a TB case or both falsely identified TB in a non-TB case, this would be counted as correctly classified. The study design inherently favors concordance and cannot detect systematic errors occurring in the same direction by both the AI and local radiologists. This creates verification bias that likely inflates reported accuracy estimates.

# OVERALL ASSESSMENT SUMMARY

**Note: This study has been rated as VERY HIGH overall risk of bias with MODERATE applicability concerns due to fundamental methodological limitations.**

| Domain | Signaling Questions | Risk of Bias | Applicability |
| --- | --- | --- | --- |
| D1: Patient Selection | 1.1 Unclear 1.2 Yes 1.3 No | HIGH | LOW |
| D2: Index Test | 2.1 Yes 2.2 Yes | LOW | LOW |
| D3: Reference Standard | 3.1 No 3.2 No | HIGH | HIGH |
| D4: Flow and Timing | 4.1 Yes 4.2 Yes* 4.3 No 4.4 Yes | HIGH | N/A |

**Overall Risk of Bias: VERY HIGH**

**Justification:** This study has 3 domains rated HIGH for Risk of Bias (D1: Patient Selection, D3: Reference Standard, D4: Flow and Timing). The fundamental methodological flaw is differential verification bias: only 6.35% of participants (235/3,705) received expert panel review, while 93.65% with concordant results were assumed correct without verification. This design systematically prevents detection of concordant errors where both AI and local radiologists make the same mistake. Additionally, the reference standard is expert radiological interpretation rather than WHO-recommended microbiological confirmation, meaning the study measures AI-radiologist agreement rather than AI's ability to detect true TB disease. The exclusion of 7.6% of patients due to incomplete CAD readings without explanation further compounds selection bias concerns.

**Overall Applicability Concern: MODERATE**

**Justification:** The study has HIGH applicability concern for the reference standard domain because it measures AI performance against expert radiological interpretation rather than microbiologically confirmed TB—the true target condition of clinical interest. The 76 "active TB" cases represent radiological diagnoses, not bacteriologically confirmed disease. However, the patient population (primary care outpatients at township health centers in TB-endemic region) and index test implementation (CAD software used as intended for TB screening/triage) are highly applicable to point-of-care settings. The overall MODERATE rating reflects this tension between poor reference standard applicability and excellent patient/test applicability.

## Key Methodological Limitations & Findings

**1. Differential verification bias (CRITICAL):** Only 235/3,705 (6.35%) received expert panel review; 3,470 (93.65%) assumed correct based on concordance without verification. Concordant errors undetectable.
**2. Imperfect reference standard (CRITICAL):** Expert radiological interpretation used instead of WHO-recommended microbiological confirmation. Cannot definitively identify true active TB disease.
**3. Incorporation/review bias:** Expert panel knew they were reviewing discordant cases, potentially influencing interpretation.
**4. Selection bias:** 305/4,010 (7.6%) excluded due to incomplete CAD readings; reason not explained, suggesting potential systematic exclusion.
**5. Reported Performance (should be interpreted with extreme caution):** • JF CXR-1 v3.0: Sensitivity 92.11% (95% CI: 86.04-98.17%), Specificity 94.54% (95% CI: 93.81-95.28%)
 • Local radiologists: Sensitivity 32.89% (95% CI: 22.33-43.46%), Specificity 99.28% (95% CI: 99.01-99.56%)
 • Based on 76 radiologically identified "active TB" cases (not microbiologically confirmed)
**6. Limited generalizability:** Another study using same JF CXR-1 version in Vietnam required different threshold (0.83 vs 0.5) for similar sensitivity, suggesting population-dependent performance.

QUADAS-2

**Chen et al. 2023**EE-Explorer: A Multimodal Artificial Intelligence System for Eye Emergency Triage and Primary Diagnosis *Study ID: Chen_2023 | Assessment Date: 2025-11-28*

____________________________________________________________________________________________________

# Phase 1: State the Review Question

*Patients (setting, intended use of index test, presentation, prior testing):*

| Patients presenting with acute ophthalmic symptoms (within 1 month) to emergency departments at 5 hospitals in China (January 2021 - September 2022). Setting: Ocular emergency departments at Zhongshan Ophthalmic Center (ZOC, development/internal testing) and 4 external hospitals (TMUGH, HEH, FAHKMU, GAEH). Intended use: AI-assisted triage to classify emergency severity (urgent/semiurgent/nonurgent) and primary diagnosis for 18 ophthalmic conditions. Presentation: First-time visits for acute ophthalmic symptoms. Eligibility: Able to complete triage form and cooperate with smartphone photography or slit-lamp examination. Prior testing: None specified. Population: 2,038 patients for triage model (1,777 training/validation + 261 internal testing + 103 external testing); 2,405 patients for diagnostic model (1,932 training/validation + 473 internal testing + 103 external testing). Disease prevalence: 26.4% urgent, 41.9% semiurgent, 31.7% nonurgent. |
| --- |

*Index test(s):*

| EE-Explorer multimodal AI system with two components: (1) TRIAGE MODEL: DenseNet201 for smartphone image feature extraction (6 sign features: normal, conjunctival congestion/hemorrhage/edema, corneal ulcer, ocular rupture) + XGBoost classifier combining image features with metadata from Triage Form for Ophthalmic Emergency (TFOE: 12 category features including symptoms, duration, ocular history). Outputs 3-level emergency classification (urgent/semiurgent/nonurgent). (2) DIAGNOSTIC MODEL: InceptionV3 for slit-lamp image sign recognition (10 ocular signs) + XGBoost classifier integrating predicted signs with TFOE metadata, visual acuity, and intraocular pressure. Outputs 18 diagnostic labels using one-versus-rest strategy. Threshold determination: Hyperparameters optimized via grid search on training data (NOT pre-specified). Explainability: Grad-CAM visualization + SHAP feature importance charts. |
| --- |

*Reference standard and target condition:*

| Reference standard: Independent assessment by 2 experienced ophthalmologists (>8 years experience) based on comprehensive ophthalmic examination including slit-lamp biomicroscopy, fundoscopy, and ancillary tests as needed. For disagreements, senior ophthalmologist consultation was sought until consensus reached. Same reference standard applied uniformly across all sites and time periods. Target conditions: (1) Triage classification: Urgent (requires immediate treatment to prevent vision loss/ocular damage), semiurgent (treatment within hours to days), nonurgent (routine care sufficient). (2) Primary diagnosis: 18 specific ophthalmic conditions including acute angle closure glaucoma, corneal ulcer, keratitis, uveitis, conjunctivitis, trauma-related conditions, and others. Reference standard determination occurred during the same emergency visit as index test data collection (no time delay). |
| --- |

# Phase 2: Draw a Flow Diagram for the Primary Study

| PATIENT FLOW DIAGRAM  ═══════════════════════════════════════════════════════════════════════ TRIAGE MODEL PATHWAY ═══════════════════════════════════════════════════════════════════════  INTERNAL (ZOC): January 1 - October 31, 2021  Initial screening: 2,450 patients  ↓  EXCLUSIONS:  ├─→ 49 patients: No qualified images  └─→ 363 patients: Lacking paired smartphone images  ↓  Final cohort: 2,038 patients  ├─→ Training/Validation: 1,777 patients  └─→ Internal Testing: 261 patients  EXTERNAL (4 hospitals): February 10 - September 10, 2022  Initial screening: 110 patients  ↓  EXCLUSIONS: Lacking qualified/paired images (number not specified)  ↓  External Testing: 103 patients  TOTAL TRIAGE MODEL ANALYSIS: 2,038 patients  • Training/Validation: 1,777 (26.4% urgent, 41.9% semiurgent, 31.7% nonurgent)  • Internal Testing: 261  • External Testing: 103  ═══════════════════════════════════════════════════════════════════════ DIAGNOSTIC MODEL PATHWAY ═══════════════════════════════════════════════════════════════════════  INTERNAL (ZOC): Same time period  Initial available: 2,508 patients  ↓  EXCLUSIONS:  ├─→ 10 patients: Lacking paired slit-lamp images  └─→ 29 patients: Not meeting study diagnosis criteria  ↓  Final cohort: 2,469 patients  ├─→ Training/Validation: 1,932 patients  └─→ Internal Testing: 473 patients (corrected to 537 per figure)  EXTERNAL: Same 103 patients as triage model after exclusions  TOTAL DIAGNOSTIC MODEL ANALYSIS: 2,405 patients  • Training/Validation: 1,932 (18 diagnostic categories)  • Internal Testing: 473  • External Testing: 103  ═══════════════════════════════════════════════════════════════════════ REFERENCE STANDARD & INDEX TEST ═══════════════════════════════════════════════════════════════════════  ALL PATIENTS RECEIVED: ├─→ INDEX TEST (same visit): │ • Triage model: Smartphone photos + TFOE metadata │ • Diagnostic model: Slit-lamp images + TFOE + VA + IOP │ └─→ REFERENCE STANDARD (same visit):  • Independent assessment by 2 experienced ophthalmologists  • Comprehensive examination (slit-lamp, fundoscopy, ancillary tests)  • Consensus by senior ophthalmologist if disagreement  • No time delay between index and reference  KEY PERFORMANCE (External Testing, n=103): TRIAGE MODEL:  • Accuracy: 94.3% (AI) vs 82.4% (triage nurses), p<.001  • Sensitivity for urgent: 100%  • Specificity: 94.7%  DIAGNOSTIC MODEL:  • Top-3 accuracy: 84.5%  • Macro-average AUROC: 0.93  • Some categories had very small samples (e.g., uveitis N=2, perforation N=1)  PILOT STUDY: Real-world testing with 100 participants in hierarchical referral |
| --- |

# Phase 3: Risk of Bias and Applicability Judgments

*QUADAS-2 is structured so that 4 key domains are each rated in terms of the risk of bias and the concern regarding applicability to the research question (as defined above). Each key domain has a set of signalling questions to help reach the judgments regarding bias and applicability.*

# DOMAIN 1: PATIENT SELECTION

## A. Risk of Bias

**Describe methods of patient selection:**This diagnostic cross-sectional validity and reliability study enrolled patients from Zhongshan Ophthalmic Center (ZOC) between January 1, 2021, and October 31, 2021, for model development and internal testing. External testing data were collected from four additional hospitals (TMUGH, HEH, FAHKMU, GAEH) between February 10, 2022, and September 10, 2022 (p.254). Eligibility criteria included: (1) patients who suffered acute ophthalmic symptoms within 1 month, (2) visited the ocular emergency department for the first time, (3) were able to complete the triage form for ophthalmic emergency (TFOE), and (4) could cooperate either by submitting smartphone photographs or receiving slit-lamp examination (p.254). Images were filtered for quality control by integrity, illumination, and clarity before inclusion. For the triage model: 2,450 patients were initially screened from ZOC, with 49 excluded for images without qualified images and 363 excluded for lacking paired smartphone images, yielding 2,038 qualified patients (1,777 training/validation + 261 internal testing). For external testing, 110 patients were screened from 4 hospitals, with exclusions for lacking qualified/paired images, yielding 103 patients (p.255, Figure 1B). The study does not explicitly state whether enrollment was consecutive or based on convenience sampling within the specified time periods.

Page reference: p.254-255, Figure 1B

| Signaling Question | Answer |
| --- | --- |
| ❖ Was a consecutive or random sample of patients enrolled? | UNCLEAR |
| ❖ Was a case-control design avoided? | YES |
| ❖ Did the study avoid inappropriate exclusions? | YES |

**Could the selection of patients have introduced bias?: RISK: UNCLEAR**

**Justification:** While the study provides clear time periods and eligibility criteria, it fails to explicitly state whether patient enrollment was consecutive or random. The lack of clarity on enrollment methodology creates uncertainty about potential selection bias. The study design (cross-sectional) is appropriate, and exclusions were reasonable (quality-based image exclusions rather than diagnostic difficulty exclusions). However, without confirmation of consecutive/random enrollment, we cannot rule out selection bias that could affect the representativeness of the enrolled population.

## B. Concerns regarding applicability

**Describe included patients (prior testing, presentation, intended use of index test and setting):**The study population matches the review question well: patients presenting to emergency departments with acute ophthalmic symptoms within 1 month of onset, which is representative of the point-of-care emergency setting. The multi-center external validation across 5 hospitals in China enhances generalizability within that healthcare context. The triage model uses smartphone-captured images (accessible technology for POC settings) and the diagnostic model uses slit-lamp images (standard equipment in primary/emergency ophthalmology settings). The spectrum of conditions (urgent, semiurgent, nonurgent) reflects real-world emergency presentations with appropriate disease prevalence distribution (26.4% urgent, 41.9% semiurgent, 31.7% nonurgent).

**Is there concern that the included patients do not match the review question?: CONCERN: LOW**

**Justification:** The patient population is highly applicable - emergency department patients with acute ophthalmic symptoms requiring triage and diagnosis, which is the exact target for POC AI-assisted emergency ophthalmology decision support.

# DOMAIN 2: INDEX TEST(S)

*If more than one index test was used, please complete for each test.*

## A. Risk of Bias

**Describe the index test and how it was conducted and interpreted:**EE-Explorer consists of two models: (1) a triage model combining DenseNet201 for smartphone image feature extraction with XGBoost for final 3-level emergency classification (urgent, semiurgent, nonurgent), and (2) a primary diagnostic model using InceptionV3 for slit-lamp image sign recognition combined with XGBoost for 18-label diagnosis classification (p.255-256, Figure 1C). Triage model inputs: Metadata from TFOE (12 category features: event, symptom duration, blurred vision, eye pain, visual field defect, flashes, floats, photophobia, tearing, excessively swollen eyelid, ocular disease history, ocular surgery history) + smartphone photographs (p.254, 256). DenseNet201 extracted 6 sign features from images (normal, conjunctival congestion, conjunctival hemorrhage, conjunctival edema, corneal ulcer, ocular rupture), with the most urgent sign added to metadata for XGBoost classification. Diagnostic model inputs: Metadata from TFOE + paired visual acuity (VA) and intraocular pressure (IOP) + slit-lamp images (p.254). InceptionV3 identified 10 ocular signs. XGBoost then integrated predicted signs with metadata using one-versus-rest strategy for 18 diagnostic labels. Threshold specification: The paper does not explicitly state pre-specified thresholds. Hyperparameters were determined through grid search (p.256), suggesting optimization on training data. Explainability: Grad-CAM was used to visualize regions influencing CNN predictions; SHAP charts showed feature contributions (p.256).

Page reference: p.254-256, Figure 1C

| Signaling Question | Answer |
| --- | --- |
| ❖ Were the index test results interpreted without knowledge of the results of the reference standard? | YES |
| ❖ If a threshold was used, was it pre-specified? | NO |

**Could the conduct or interpretation of the index test have introduced bias?: RISK: HIGH**

**Justification:** Question 2.2 is answered "No" because thresholds/hyperparameters were optimized via grid search on the same dataset used for development, without pre-specification from external sources or prior validation studies. While the AI models were appropriately blinded to reference standard results during inference (Question 2.1 = Yes), the threshold optimization creates risk of overfitting and potentially inflated performance metrics. The study did include separate internal testing (261 triage, 473 diagnostic) and external testing (103 patients) sets, which partially mitigates this concern, but the fundamental issue of data-driven threshold selection without pre-specification introduces high risk of bias for the index test domain.

## B. Concerns regarding applicability

**Is there concern that the index test, its conduct, or interpretation differ from the review question?: CONCERN: LOW**

**Justification:** The index tests are highly applicable to point-of-care settings. The triage model uses smartphone photographs (accessible, portable technology ideal for remote/POC triage) combined with structured symptom questionnaires. The diagnostic model uses slit-lamp imaging (standard equipment in ophthalmology clinics/EDs). The multimodal approach (images + metadata + clinical measurements) reflects realistic clinical workflows. The pilot study (N=100) demonstrated feasibility in a hierarchical referral pattern. Explainability features (Grad-CAM, SHAP) enhance clinical acceptability. The system was designed for and tested in its intended use context.

# DOMAIN 3: REFERENCE STANDARD

## A. Risk of Bias

**Describe the reference standard and how it was conducted and interpreted:**The reference standard was independent assessment by 2 experienced ophthalmologists (>8 years experience) based on comprehensive ophthalmic examination including slit-lamp biomicroscopy, fundoscopy, and ancillary tests as needed (p.254, 256). For disagreements between the two ophthalmologists, senior ophthalmologist consultation was sought until consensus was reached. The same reference standard methodology was applied uniformly across all study sites and time periods. Reference standard assessment occurred during the same emergency visit as index test data collection, with no time delay. The ophthalmologists' assessments were independent of the AI system predictions.

Page reference: p.254, 256

| Signaling Question | Answer |
| --- | --- |
| ❖ Is the reference standard likely to correctly classify the target condition? | YES |
| ❖ Were the reference standard results interpreted without knowledge of the results of the index test? | YES |

**Could the reference standard, its conduct, or its interpretation have introduced bias?: RISK: LOW**

**Justification:** The reference standard is appropriate for both triage classification and primary diagnosis. Expert ophthalmologist assessment based on comprehensive examination is the gold standard for ophthalmic diagnosis. The consensus methodology (2 independent ophthalmologists with senior consultation for disagreements) enhances reliability. The same reference standard was applied uniformly across all sites and time periods, preventing differential verification bias. The ophthalmologists were independent of the AI system and performed their assessments based on clinical examination, ensuring blinding from index test results. No indication of incorporation bias or review bias.

## B. Concerns regarding applicability

**Is there concern that the target condition as defined by the reference standard does not match the review question?: CONCERN: LOW**

**Justification:** The target conditions (triage classification into urgent/semiurgent/nonurgent and specific diagnoses for 18 ophthalmic conditions) are clinically relevant and match the review question for AI-assisted emergency ophthalmology decision support. Expert ophthalmologist assessment is the appropriate reference standard for both triage urgency and diagnostic accuracy. The study acknowledges that the diagnostic model does not yet cover all emergent conditions (e.g., central retinal artery occlusion, retinal detachment were excluded), which is a limitation of scope rather than applicability of the reference standard itself.

# DOMAIN 4: FLOW AND TIMING

## A. Risk of Bias

**Describe any patients who did not receive the index test(s) and/or reference standard or who were excluded from the 2x2 table (refer to flow diagram):**Figure 1B (p.255) shows complete patient flow. All qualified patients were included in analysis. Exclusions occurred only at enrollment stage based on image quality criteria (49 without qualified images, 363 without paired smartphone images for triage model; 10 without paired slit-lamp images, 29 not meeting study diagnosis criteria for diagnostic model). No patients were excluded after receiving the reference standard. Internal testing: 261/261 triage patients and 473/473 diagnostic patients analyzed. External testing: 103/103 analyzed for both models.

**Describe the time interval and any interventions between index test(s) and reference standard:**There was no time delay between index test data collection and reference standard assessment - both occurred during the same emergency visit. Index test data (images, metadata, clinical measurements) were collected first, followed by independent ophthalmologist assessment. The same-visit timing ensures that patient condition remained stable between index test and reference standard, preventing interval changes that could affect diagnostic concordance.

| Signaling Question | Answer |
| --- | --- |
| ❖ Was there an appropriate interval between index test(s) and reference standard? | YES |
| ❖ Did all patients receive a reference standard? | YES |
| ❖ Did patients receive the same reference standard? | YES |
| ❖ Were all patients included in the analysis? | YES |

**Could the patient flow have introduced bias?: RISK: LOW**

**Justification:** All four signaling questions are answered "Yes," indicating low risk of bias for flow and timing. There was no time delay between index test data collection and reference standard assessment (same emergency visit). All patients received the same reference standard (expert ophthalmologist consensus). Complete patient flow is documented in Figure 1B with clear accounting of exclusions at appropriate stages. All enrolled and qualified patients were included in the final analysis with no post-reference-standard dropouts.

# OVERALL ASSESSMENT SUMMARY

**Note: This study has been rated as MODERATE-HIGH overall risk of bias with LOW applicability concerns.**

| Domain | Signaling Questions | Risk of Bias | Applicability |
| --- | --- | --- | --- |
| D1: Patient Selection | 1.1 Unclear 1.2 Yes 1.3 Yes | UNCLEAR | LOW |
| D2: Index Test | 2.1 Yes 2.2 No | HIGH | LOW |
| D3: Reference Standard | 3.1 Yes 3.2 Yes | LOW | LOW |
| D4: Flow and Timing | 4.1 Yes 4.2 Yes 4.3 Yes 4.4 Yes | LOW | N/A |

**Overall Risk of Bias: MODERATE-HIGH**

**Justification:** The study has one domain rated HIGH risk (Domain 2: Index Test due to non-pre-specified thresholds determined by grid search) and one domain rated UNCLEAR (Domain 1: Patient Selection due to lack of explicit confirmation of consecutive/random enrollment). The high risk of bias in Domain 2 is the primary concern, as threshold optimization on the study dataset may inflate performance estimates. However, this is partially mitigated by separate internal and external testing sets. Domains 3 and 4 show low risk of bias with appropriate reference standard methodology and complete patient flow documentation.

**Overall Applicability Concern: LOW**

**Justification:** All assessed domains (1, 2, 3) show low applicability concerns. The study population (emergency department patients with acute ophthalmic symptoms), index tests (smartphone images for remote triage, slit-lamp images for diagnosis), and reference standard (expert ophthalmologist assessment) are highly relevant to AI-based clinical decision support using point-of-care imaging. The multi-center design, pilot testing in hierarchical referral settings, and use of accessible imaging modalities enhance applicability to real-world POC settings.

## Key Strengths & Limitations

**STRENGTHS:
1. Multi-center external validation:** Testing on 103 independent patients from 4 external hospitals beyond development site
**2. Prospective pilot study:** Real-world application testing with N=100 participants in hierarchical referral pattern
**3. Multimodal design:** Integration of metadata, symptoms, and imaging provides comprehensive clinical assessment
**4. Complete documentation:** Clear CONSORT-style flow diagram with explicit exclusion counts
**5. Human comparator:** Direct comparison with triage nurses showing significant improvement (94.3% vs 82.4%, p<.001)
**6. Explainability:** Grad-CAM and SHAP visualizations for model interpretability

**LIMITATIONS:
1. Threshold optimization (HIGH impact):** Grid search hyperparameter tuning without pre-specification risks overfitting
**2. Enrollment method unclear:** No explicit statement of consecutive vs. convenience sampling
**3. Geographic limitation:** All data from Chinese hospitals may limit generalizability to other populations/healthcare systems
**4. No long-term follow-up:** Reference standard based on single visit without outcome verification
**5. Limited disease spectrum:** Diagnostic model focused on anterior segment; excluded some emergent conditions (central retinal artery occlusion, retinal detachment)
**6. Small external testing subgroups:** Some diagnostic categories had very limited representation in external testing (acute uveitis N=2, perforation N=1, orbital cellulitis N=1)

## Sample Size Considerations

• Triage model: 2,038 patients total (1,777 training/validation; 261 internal testing; 103 external testing)
• Diagnostic model: 2,405 patients total (1,932 training/validation; 473 internal testing; 103 external testing)
• Disease prevalence in training/validation: 26.4% urgent, 41.9% semiurgent, 31.7% nonurgent
• External testing adequate for overall performance but small for class-specific estimates in rare conditions

QUADAS-2

**Fergus et al. 2023**Pressure Ulcer Categorization and Reporting in Domiciliary Settings Using Deep Learning and Mobile Devices: A Clinical Trial to Evaluate End-to-End Performance *Study ID: Fergus_2023 | Assessment Date: 2025-11-28*

____________________________________________________________________________________________________

# Phase 1: State the Review Question

*Patients (setting, intended use of index test, presentation, prior testing):*

| 50 patients seen by district nurses during routine home visits in domiciliary (home) settings, Mersey Care NHS Foundation Trust, UK (March 15 - December 21, 2021). Setting: Point-of-care - patients' homes with district nursing care. Intended use: AI-assisted pressure ulcer classification to support clinical decision-making by non-specialist district nurses with minimal pressure ulcer categorization experience. Presentation: Patients with pressure ulcers requiring assessment during routine district nursing visits. Prior testing: None specified. Population characteristics: Not detailed in paper. Final analysis: 216 images across 6 categories - Category I: 5 images (2.3%), Category II: 93 images (43.1%), Category III: 11 images (5.1%), Category IV: 0 images (0%), Deep Tissue Injury (DTI): 30 images (13.9%), Unstageable: 77 images (35.6%). NOTE: Severe class imbalance and complete absence of Category IV cases (most severe category). |
| --- |

*Index test(s):*

| Faster Region-based Convolutional Neural Network (Faster R-CNN) using ResNet101 base network, pre-trained on COCO dataset and fine-tuned on 4,290 pressure ulcer images (from Medetec dataset + Google Images) containing 5,084 tagged objects across 6 classes. District nurses used iOS and Android mobile phones to photograph pressure ulcers in patients' homes. Images transmitted over 4/5G communications to TensorFlow Serving inferencing server. Server processed images and returned classifications viewable in WordPress gallery 2-3 seconds after photograph taken. Model classifies six categories: Category I, II, III, IV, Deep Tissue Injuries, and Unstageable pressure ulcers. Classification automated without human interpretation required. CRITICAL LIMITATION: Confidence score thresholds evaluated retrospectively at @.30, @.50, @.75, @.90, with optimal threshold (@.75 for cropped images) determined by evaluating performance on the clinical trial data - NOT pre-specified. Training data consisted of controlled/pre-processed images (Medetec, Google), contrasting with ad-hoc quality images from domiciliary settings, contributing to ~30% performance drop in real-world deployment. |
| --- |

*Reference standard and target condition:*

| Reference standard: Assessment by specialist nurses and clinical staff from Mersey Care NHS Foundation Trust. CRITICAL LIMITATION - REVIEW BIAS: Specialist nurses "reviewed the classifications made [by AI] and either confirmed the category(s) was correct or reported what the correct category should be" (p.7). This means reference standard assessors saw AI classifications BEFORE making their assessments, creating circularity/incorporation bias where assessors may have been influenced toward confirming AI output, artificially inflating agreement metrics. Assessor qualifications not specified (unclear if tissue viability nurses with formal certification). Inter-rater reliability not assessed. Target condition: Pressure ulcer category classification according to 6-level National Pressure Ulcer Advisory Panel (NPUAP) system: Category I (non-blanchable erythema), Category II (partial thickness skin loss), Category III (full thickness skin loss), Category IV (full thickness tissue loss), Deep Tissue Injury, and Unstageable. Reference standard assessments made after AI classification during or shortly after image collection. |
| --- |

# Phase 2: Draw a Flow Diagram for the Primary Study

| PATIENT FLOW DIAGRAM  Clinical Trial Period: March 15 - December 21, 2021 (8 months) Setting: Domiciliary (home) settings, Mersey Care NHS Foundation Trust, UK  ↓ ENROLLED PATIENTS (n = 50 patients) District nurses photographed pressure ulcers during routine patient visits  ↓ INITIAL IMAGE COLLECTION (n = 1,016 images)  ↓ FIRST QUALITY CHECKING EXCLUSIONS (1,016 → 624): Excluded 392 images (38.6%): ├─→ Blurry images ├─→ Images containing identifiable patient/staff information  └─→ Images that did not contain pressure ulcers  ↓ SECOND REVIEW EXCLUSIONS (624 → 216): Excluded 408 images (65.4%): └─→ "Images that looked similar" (same pressure ulcer repeatedly with little variance)  Vaguely defined criterion - potential for bias  Specific numbers at each exclusion step NOT provided  ↓ FINAL TEST SET FOR ANALYSIS (n = 216 images from 50 patients)  TOTAL EXCLUSIONS: 800/1,016 images (78.7%)  ↓ DISTRIBUTION IN FINAL TEST SET (Severe Class Imbalance): ├─→ Category I: 5 images (2.3%) [SEVERELY UNDER-REPRESENTED] ├─→ Category II: 93 images (43.1%) [DOMINANT CLASS] ├─→ Category III: 11 images (5.1%) [SEVERELY UNDER-REPRESENTED] ├─→ Category IV: 0 images (0%) [MISSING - MOST SEVERE CATEGORY] ├─→ Deep Tissue Injury: 30 images (13.9%) └─→ Unstageable: 77 images (35.6%) [SECOND DOMINANT CLASS]  ═══════════════════════════════════════════════════════════════════════ INDEX TEST & REFERENCE STANDARD APPLICATION ═══════════════════════════════════════════════════════════════════════  ALL 216 IMAGES RECEIVED:  ↓ INDEX TEST (AI Classification): ├─→ District nurses captured images on mobile phones (iOS/Android) ├─→ Transmitted via 4/5G to TensorFlow Serving server ├─→ Faster R-CNN with ResNet101 processed images ├─→ Classifications returned in 2-3 seconds └─→ Viewed in WordPress gallery  ↓ REFERENCE STANDARD (Specialist Nurse Assessment): ** CRITICAL BIAS: REVIEW/INCORPORATION BIAS ** └─→ Specialist nurses REVIEWED AI CLASSIFICATIONS FIRST └─→ Then "confirmed the category(s) was correct OR reported what   the correct category should be" └─→ Reference standard assessors saw index test results BEFORE making  their own assessment └─→ Creates circularity - may inflate agreement artificially  PERFORMANCE EVALUATION: • Confidence thresholds tested: @.30, @.50, @.75, @.90 • Optimal threshold (@.75) selected AFTER evaluating test data performance • POST-HOC THRESHOLD OPTIMIZATION on test set (not pre-specified)  REPORTED PERFORMANCE (at @.75 for cropped images): • mAP (mean Average Precision): 0.6796 • mAR (mean Average Recall): 0.6997  • F1-score: 0.6786 • ~30% performance drop from training/validation to clinical deployment  due to domain shift (controlled training images vs. ad-hoc clinical images)  CRITICAL METHODOLOGICAL ISSUES: 1. 78.7% image exclusion with vague criteria ("similar images") 2. Review bias - reference standard saw AI before assessment 3. Threshold optimization on test data (not pre-specified) 4. Spectrum bias - no Category IV, severe class imbalance 5. Training-testing domain mismatch 6. Small sample (50 patients, 216 images) 7. No confidence intervals reported |
| --- |

# Phase 3: Risk of Bias and Applicability Judgments

*QUADAS-2 is structured so that 4 key domains are each rated in terms of the risk of bias and the concern regarding applicability to the research question (as defined above). Each key domain has a set of signalling questions to help reach the judgments regarding bias and applicability.*

# DOMAIN 1: PATIENT SELECTION

## A. Risk of Bias

**Describe methods of patient selection:**The study was a clinical trial conducted by Mersey Care NHS Foundation Trust evaluating a deep learning system for pressure ulcer classification. The trial enrolled 50 patients seen by district nurses during routine patient visits in domiciliary (home) settings (p.12). The study ran between March 15, 2021 and December 21, 2021 (p.7). During this eight-month trial, 1016 images were collected initially (p.9, p.12). Following quality checking, this number was reduced to 624 by removing blurry images, images that contained identifiable patient/staff information, and images that did not contain pressure ulcers (p.9). A second review removed images that were similar (same pressure ulcer taken repeatedly with little variance). The final test set contained 216 images: 5 Category I, 93 Category II, 11 Category III, 0 Category IV (none seen during trial), 30 Deep Tissue Injury (DTI), and 77 Unstageable (p.9). The paper does not explicitly state whether patient enrollment was consecutive, random, or convenience-based, though it appears images were collected opportunistically during routine visits.

Page reference: p.7, p.9, p.12

| Signaling Question | Answer |
| --- | --- |
| ❖ Was a consecutive or random sample of patients enrolled? | UNCLEAR |
| ❖ Was a case-control design avoided? | YES |
| ❖ Did the study avoid inappropriate exclusions? | UNCLEAR |

**Could the selection of patients have introduced bias?: RISK: UNCLEAR**

**Justification:** The enrollment method (consecutive vs. convenience) is not explicitly stated, making it impossible to determine if selection bias was introduced. The large number of image exclusions (800/1016 = 79% excluded) raises concern about selection bias, though some exclusions were appropriate (blurry images, identifiable information). The vaguely defined exclusion of "similar" images could have systematically excluded certain types of pressure ulcers. The absence of Category IV cases and the severe class imbalance (5 Category I vs. 93 Category II) suggests the sample may not be representative of the full spectrum of pressure ulcer presentations. The small sample size (50 patients, 216 images) limits generalizability.

## B. Concerns regarding applicability

**Describe included patients (prior testing, presentation, intended use of index test and setting):**50 patients seen by district nurses during routine home visits in the Mersey Care NHS Foundation Trust catchment area, UK (p.12). Final analysis included 216 images across 6 categories: 5 Category I (2.3%), 93 Category II (43.1%), 11 Category III (5.1%), 0 Category IV (0%), 30 DTI (13.9%), and 77 Unstageable (35.6%) (p.9). The target population is patients with pressure ulcers requiring assessment in domiciliary settings, representing real-world community nursing practice.

**Is there concern that the included patients do not match the review question?: CONCERN: LOW**

**Justification:** The study evaluates AI for pressure ulcer classification in point-of-care settings (domiciliary/home care), which closely matches the review question about AI-enabled clinical decision support using point-of-care imaging. District nurses using mobile phones in patients' homes represents authentic point-of-care imaging. The population (community-dwelling patients with pressure ulcers) is appropriate for the review scope. However, the absence of Category IV cases means the full disease spectrum was not evaluated.

# DOMAIN 2: INDEX TEST(S)

*If more than one index test was used, please complete for each test.*

## A. Risk of Bias

**Describe the index test and how it was conducted and interpreted:**The index test was a Faster Region-based Convolutional Neural Network (Faster R-CNN) using ResNet101 as the base network, pre-trained on COCO dataset and fine-tuned on 4,290 pressure ulcer images (from Medetec dataset and Google Images) containing 5,084 tagged objects across 6 classes (p.4, p.6). District nurses used iOS and Android mobile phones to photograph pressure ulcers in patients' homes and transmit them over 4/5G communications to a TensorFlow Serving inferencing server (p.7). The server processed images and returned classifications viewable in a WordPress gallery 2-3 seconds after the photograph was taken (p.7). The model classifies six categories: Category I, II, III, IV, Deep Tissue Injuries, and Unstageable pressure ulcers (p.1). Classification was automated without human interpretation of AI outputs required. The authors used confidence score thresholds at @.30, @.50, @.75, and @.90 for evaluation, with optimal performance at @.75 (p.8-11).

Page reference: p.1, p.4, p.6-7, p.8-11

| Signaling Question | Answer |
| --- | --- |
| ❖ Were the index test results interpreted without knowledge of the results of the reference standard? | YES |
| ❖ If a threshold was used, was it pre-specified? | NO |

**Could the conduct or interpretation of the index test have introduced bias?: RISK: HIGH**

**Justification:** While the AI interpretation was appropriately blinded to the reference standard (automated classification), the threshold was not pre-specified. The study evaluated multiple confidence score thresholds (@.30, @.50, @.75, @.90) on the clinical trial data and selected the optimal threshold based on observed performance (p.8-11). This post-hoc threshold optimization on the test set leads to optimistic performance estimates and reduced generalizability. Additionally, the training data consisted of low-quality images from Medetec dataset (175 images) and Google Images (675 images), which may not match the ad-hoc quality of images obtained in domiciliary settings (p.4). The significant difference between training data (controlled images) and clinical trial data (ad-hoc images) contributed to the 30% performance drop in real-world deployment (p.3).

## B. Concerns regarding applicability

**Is there concern that the index test, its conduct, or interpretation differ from the review question?: CONCERN: LOW**

**Justification:** The index test closely matches the intended point-of-care use case: district nurses using standard mobile phones (iOS/Android) to photograph pressure ulcers in patients' homes, with images transmitted via 4/5G networks for AI classification (p.7). This represents authentic point-of-care imaging workflow. The system was designed for non-specialist users (district nurses with "minimal experience" in pressure ulcer categorization) providing clinical decision support (p.1-2). The quick turnaround (2-3 seconds for results) is appropriate for point-of-care applications.

# DOMAIN 3: REFERENCE STANDARD

## A. Risk of Bias

**Describe the reference standard and how it was conducted and interpreted:**The reference standard was assessment by specialist nurses and clinical staff from Mersey Care NHS Foundation Trust (p.7). CRITICAL LIMITATION: The specialist nurses "reviewed the classifications made [by the AI] and either confirmed the category(s) was correct or reported what the correct category should be" (p.7). This means the reference standard assessors saw the AI classifications BEFORE making their assessments, creating review/incorporation bias. The qualifications of "specialist nurses" and "clinical staff" are not specified - it is unclear if they were tissue viability nurses with formal certification. Inter-rater reliability was not assessed; the paper does not report whether multiple specialist nurses reviewed images or what inter-rater agreement was achieved.

Page reference: p.7

| Signaling Question | Answer |
| --- | --- |
| ❖ Is the reference standard likely to correctly classify the target condition? | UNCLEAR |
| ❖ Were the reference standard results interpreted without knowledge of the results of the index test? | NO |

**Could the reference standard, its conduct, or its interpretation have introduced bias?: RISK: HIGH**

**Justification:** Severe review/incorporation bias is present. The specialist nurses explicitly "reviewed the classifications made [by AI] and either confirmed the category(s) was correct or reported what the correct category should be" (p.7), meaning they saw the index test results before making reference standard assessments. This creates circularity where assessors may have been biased toward confirming AI output, artificially inflating agreement/accuracy metrics. Additionally, the qualifications of reference standard assessors are unclear - "specialist nurses" and "clinical staff" are not defined, and it is uncertain if they were certified tissue viability nurses. Inter-rater reliability was not assessed, creating further uncertainty about reference standard quality.

## B. Concerns regarding applicability

**Is there concern that the target condition as defined by the reference standard does not match the review question?: CONCERN: LOW**

**Justification:** The target condition (pressure ulcer category classification according to NPUAP 6-level system) is appropriate and clinically relevant. Specialist nurse assessment is an acceptable reference standard for pressure ulcer classification in clinical practice. The categories (I, II, III, IV, DTI, Unstageable) are standard clinical classifications. However, the absence of formal verification (e.g., by certified wound care specialists or tissue viability nurses) introduces some uncertainty.

# DOMAIN 4: FLOW AND TIMING

## A. Risk of Bias

**Describe any patients who did not receive the index test(s) and/or reference standard or who were excluded from the 2x2 table (refer to flow diagram):**MAJOR EXCLUSION BIAS: Of 1,016 initially collected images, 800 (78.7%) were excluded before final analysis. First round excluded 392 images (blurry images, identifiable information, no pressure ulcers) - these exclusions appear appropriate. Second round excluded 408 images described as "images that looked similar" (same pressure ulcer repeatedly with little variance) (p.9). This "similar images" criterion is vaguely defined and could introduce systematic bias. The specific numbers excluded at each step after the first 392 are not provided. All 216 final images received both index test and reference standard.

**Describe the time interval and any interventions between index test(s) and reference standard:**The time interval between AI classification and specialist nurse review is not explicitly stated but appears to be minimal (likely same day or shortly after image capture). The AI provided classifications within 2-3 seconds of image upload (p.7). Specialist nurses then reviewed these AI classifications and made their assessments. No interventions between index test and reference standard are described.

| Signaling Question | Answer |
| --- | --- |
| ❖ Was there an appropriate interval between index test(s) and reference standard? | UNCLEAR |
| ❖ Did all patients receive a reference standard? | YES |
| ❖ Did patients receive the same reference standard? | YES |
| ❖ Were all patients included in the analysis? | NO |

**Could the patient flow have introduced bias?: RISK: HIGH**

**Justification:** Significant exclusion bias and spectrum bias are present. First, 800/1,016 images (78.7%) were excluded, with 408 removed based on vaguely defined "similar images" criterion that could systematically exclude certain pressure ulcer presentations. Second, severe spectrum bias exists: no Category IV pressure ulcers (most severe category) were observed during the entire 8-month trial, and severe class imbalance affects reliability (only 5 Category I images vs. 93 Category II). The small final sample (50 patients, 216 images) with highly unbalanced category distribution limits the study's ability to accurately assess AI performance across the full spectrum of pressure ulcer severity. The 79% exclusion rate without clear justification for all exclusions raises serious concerns about selection bias.

# OVERALL ASSESSMENT SUMMARY

**CRITICAL WARNING: This study has been rated as VERY HIGH overall risk of bias with multiple severe methodological limitations.**

| Domain | Signaling Questions | Risk of Bias | Applicability |
| --- | --- | --- | --- |
| D1: Patient Selection | 1.1 Unclear 1.2 Yes 1.3 Unclear | UNCLEAR | LOW |
| D2: Index Test | 2.1 Yes 2.2 No | HIGH | LOW |
| D3: Reference Standard | 3.1 Unclear 3.2 No | HIGH | LOW |
| D4: Flow and Timing | 4.1 Unclear 4.2 Yes 4.3 Yes 4.4 No | HIGH | N/A |

**Overall Risk of Bias: VERY HIGH**

**Justification:** This study has 3 domains rated HIGH risk (D2: Index Test, D3: Reference Standard, D4: Flow and Timing) and 1 domain rated UNCLEAR (D1: Patient Selection). Key biases include: (1) Threshold optimization bias - confidence score threshold selected post-hoc based on observed performance on test data (D2); (2) Review/incorporation bias - specialist nurses saw AI classifications BEFORE making reference standard assessments, creating circularity that may artificially inflate agreement (D3); (3) Exclusion bias - 79% of collected images excluded with vaguely defined "similar images" criterion (D4); (4) Spectrum bias - no Category IV cases evaluated and severe class imbalance (only 5 Category I vs 93 Category II) (D4); (5) Training-testing domain mismatch contributing to 30% performance drop (D2). The combination of these severe methodological limitations renders the reported performance metrics highly unreliable.

**Overall Applicability Concern: LOW**

**Justification:** Despite the very high risk of bias, applicability concerns are LOW. The study setting (district nurses using mobile phones in patients' homes), patient population (community-dwelling individuals with pressure ulcers), index test (AI classification via mobile imaging), and reference standard (specialist nurse assessment) all match the review question for AI-assisted POC clinical decision support. The 2-3 second turnaround time and use of standard consumer mobile devices (iOS/Android) represent realistic POC implementation. However, the absence of Category IV cases means applicability to the full spectrum of pressure ulcer severity is limited.

## Critical Methodological Limitations

**1. REVIEW/INCORPORATION BIAS (SEVERE):** Reference standard assessors saw AI classifications before making their assessments, creating circularity. Specialist nurses "reviewed the classifications made and either confirmed the category(s) was correct or reported what the correct category should be" (p.7). This may artificially inflate agreement metrics.

**2. THRESHOLD OPTIMIZATION ON TEST DATA (SEVERE):** Confidence thresholds (@.30, @.50, @.75, @.90) were evaluated on clinical trial data and optimal threshold selected post-hoc, not pre-specified. This risks overfitting and optimistic performance estimates.

**3. MASSIVE EXCLUSION BIAS (SEVERE):** 800/1,016 images (78.7%) excluded, with 408 removed based on vaguely defined "similar images" criterion. Could systematically exclude certain pressure ulcer types.

**4. SPECTRUM BIAS (SEVERE):** No Category IV cases (most severe) observed during entire 8-month trial. Severe class imbalance: Category I (N=5, 2.3%), Category II (N=93, 43.1%), Category III (N=11, 5.1%), Category IV (N=0, 0%), DTI (N=30, 13.9%), Unstageable (N=77, 35.6%).

**5. TRAINING-TESTING DOMAIN MISMATCH:** Model trained on controlled/pre-processed images (Medetec, Google) but tested on ad-hoc quality images from domiciliary settings, contributing to ~30% performance drop (p.3).

**6. SMALL SAMPLE SIZE:** Only 50 patients and 216 images analyzed. Inadequate for rare categories (5 Category I, 11 Category III). No confidence intervals reported.

**7. UNCLEAR REFERENCE STANDARD QUALITY:** Assessor qualifications not specified. No inter-rater reliability assessment.

**8. CLASS IMBALANCE IN TRAINING DATA:** Training dataset also had imbalance (432 Category III vs 1,401 Category II tags) likely contributing to poor performance on under-represented classes.

## Reported Performance (Interpret with Extreme Caution)

**At confidence threshold @.75 for cropped images:**• mAP (mean Average Precision): 0.6796
• mAR (mean Average Recall): 0.6997
• F1-score: 0.6786
• ~30% performance drop from training/validation to real-world deployment

**WARNING:** These metrics are likely inflated due to (1) threshold optimization on test data, (2) review bias, and (3) exclusion of 79% of images. True performance in unselected real-world populations is likely substantially lower.

QUADAS-2

**Heydon et al. 2021**Prospective Evaluation of an Artificial Intelligence-Enabled Algorithm for Automated
Diabetic Retinopathy Screening of 30,000 Patients *Study ID: Heydon_2021 | Assessment Date: 2025-11-28*

____________________________________________________________________________________________________

# Phase 1: State the Review Question

*Patients (setting, intended use of index test, presentation, prior testing):*

| Consecutive screening episodes from three English NHS Diabetic Eye Screening Programmes (DESPs) during 2017: North East London (NEL), South East London (SEL), and Gloucestershire (GS). Setting: Population-based diabetic retinopathy screening in community settings (point-of-care screening). Intended use: AI-assisted automated triage to identify patients requiring human grader review. Presentation: Routine screening participants with Type 1 and Type 2 diabetes attending scheduled screening appointments. Prior testing: Annual or biennial retinopathy screening as per NHS protocol. Population: 30,405 consecutive screening episodes - 10,137 from NEL, 10,091 from GS, 10,177 from SEL. Demographics: Mean ages 60-66 years, 54-57% male, ethnically diverse (35.8-66.9% White, 2.5-43.0% Asian, 1-26.3% Black depending on centre). Uptake rates: 77-83%. Disease prevalence: 70.1% no retinopathy (R0M0), 18.1% mild-moderate NPDR without maculopathy (R1M0), 7.2% mild-moderate NPDR with maculopathy (R1M1), 1.8% moderate-severe NPDR (R2), 0.6% proliferative DR (R3), 2.4% ungradable (U). |
| --- |

*Index test(s):*

| EyeArt v2.1.0 (Eyenuk, Woodland Hills, CA), a CE-marked machine learning-enabled automated retinal image analysis system. Software installed on servers at Homerton Hospital on 3 January 2017. Pseudo-anonymised images processed on encrypted disks: NEL on 14 January 2017, SEL on 6 February 2017, GS on 25 April 2017. Binary classification output: test-positive or test-negative for each screening episode. Episodes with images classified as un-assessable (technical failure) by software allocated as test-positive for safety. At least two digital image fields captured per eye (one disc-centred, one macula-centred) per NHS DESP protocol. All images captured per episode included without editing or selection, including partial retinal images, poor quality images, and non-retinal images. AI processing occurred in parallel with usual care; patient clinical pathway not affected. Human grade for each image not available while images being processed by EyeArt software. NOTE: Study used CE-marked commercial software (v2.1.0), an updated version from prior studies with "differing software thresholding cut-offs being implemented," but exact classification thresholds not reported and pre-specification not explicitly confirmed. |
| --- |

*Reference standard and target condition:*

| Reference standard: Human grading performed according to NHS Diabetic Eye Screening Programme (DESP) guidelines. Multi-tier grading pathway: (1) All images reviewed by primary grader, (2) Patients with mild or worse retinopathy/maculopathy (plus 10% of "no retinopathy") reviewed by secondary grader, (3) Discrepancies resolved by tertiary arbitration grader. Final human grade in worst eye used as reference standard. Grading classifications: R0M0 (no retinopathy), R1M0 (mild-to-moderate NPDR without referable maculopathy), R1M1 (mild-to-moderate NPDR with referable maculopathy), R2 (moderate-to-severe NPDR), R3 (proliferative DR), U (ungradable). Human graders were trained and underwent regular quality assurance. Authors note that "the final human grade is a stable reference standard since measures of screening performance and accuracy of performance metrics were not materially altered when the reference standard was further refined by arbitration by an expert reading centre" in prior validation work. Target condition: Referable diabetic retinopathy requiring clinical review or treatment (R1M1, R2, R3) versus non-referable (R0M0, R1M0). ETDRS grade equivalencies provided. |
| --- |

# Phase 2: Draw a Flow Diagram for the Primary Study

| PATIENT FLOW DIAGRAM - MULTI-CENTRE PROSPECTIVE STUDY  ═══════════════════════════════════════════════════════════════════════ RECRUITMENT STRATEGY: Consecutive Enrollment ═══════════════════════════════════════════════════════════════════════  Target: ~10,000 consecutive screening episodes per centre during 2017  CENTRE 1: NORTH EAST LONDON (NEL)  Images processed: 14 January 2017  Final episodes: 10,137  CENTRE 2: GLOUCESTERSHIRE (GS)   Images processed: 25 April 2017  Final episodes: 10,091  CENTRE 3: SOUTH EAST LONDON (SEL)  Images processed: 6 February 2017  Final episodes: 10,177  ═══════════════════════════════════════════════════════════════════════ TOTAL COHORT: 30,405 CONSECUTIVE SCREENING EPISODES ═══════════════════════════════════════════════════════════════════════  INCLUSION: ALL images captured per episode ├─→ NO editing or selection prior to AI processing ├─→ Included partial retinal images ├─→ Included poor quality images ├─→ Included non-retinal images (e.g., cataracts) └─→ Included ungradable images (2.4% overall; range 1.9-3.0% by centre)  EXCLUSIONS: NONE └─→ All 30,405 episodes included in final analysis  DISTRIBUTION BY RETINOPATHY GRADE (n=30,405): ├─→ R0M0 (no retinopathy): 21,322 (70.1%) ├─→ R1M0 (mild-moderate NPDR, no maculopathy): 5,495 (18.1%) ├─→ R1M1 (mild-moderate NPDR with maculopathy): 2,183 (7.2%) ├─→ R2 (moderate-severe NPDR): 553 (1.8%) ├─→ R3 (proliferative DR): 187 (0.6%) └─→ U (ungradable): 721 (2.4%)  REFERABLE vs NON-REFERABLE: • Non-referable (R0M0 + R1M0): 26,817 (88.2%) • Referable (R1M1 + R2 + R3): 2,923 (9.6%) • Ungradable (U): 721 (2.4%)  ═══════════════════════════════════════════════════════════════════════ INDEX TEST & REFERENCE STANDARD - PARALLEL PROCESSING ═══════════════════════════════════════════════════════════════════════  ALL 30,405 EPISODES RECEIVED BOTH:  INDEX TEST (EyeArt v2.1.0): ├─→ Software installed at Homerton Hospital (3 Jan 2017) ├─→ Pseudo-anonymised images on encrypted disks ├─→ Automated processing without human intervention ├─→ Binary output: Test-positive OR Test-negative ├─→ Technical failures → classified as test-positive (safety measure) └─→ BLINDED: Human grades NOT available during AI processing  REFERENCE STANDARD (NHS DESP Human Grading): ├─→ Multi-tier pathway applied to ALL episodes: │ 1. Primary grader reviews all images │ 2. Secondary grader reviews DR-positive + 10% of R0 │ 3. Tertiary arbitration grader resolves discrepancies ├─→ Final grade in worst eye = reference standard ├─→ Standardised NHS DESP protocol across all centres └─→ BLINDED: Graders performed assessments as routine care,  independent of AI results  TIMING: Same digital images analysed by both AI and humans  (minimal interval, both from 2017 screening)  ═══════════════════════════════════════════════════════════════════════ ANALYSIS - COMPLETE DATA FOR ALL 30,405 EPISODES ═══════════════════════════════════════════════════════════════════════  NO MISSING DATA - NO EXCLUSIONS POST-ENROLLMENT  REPORTED PERFORMANCE (Overall): • Sensitivity for referable DR: 94.7% (95% CI: 93.8-95.5%) • Specificity for R0M0: 67.9% (95% CI: 67.3-68.5%) • Technical failure rate: 3.0% (range 1.7-5.7% by centre)  CENTRE-SPECIFIC PERFORMANCE: • NEL: Sensitivity 94.6%, Specificity for R0M0 71.0% • GS: Sensitivity 94.2%, Specificity for R0M0 65.2% • SEL: Sensitivity 95.2%, Specificity for R0M0 67.3%  KEY STRENGTHS: 1. Consecutive enrollment - no selection bias 2. All episodes included - no verification bias 3. Independent blinding both directions 4. Real-world diverse populations 5. Complete transparent reporting 6. Multi-centre validation |
| --- |

# Phase 3: Risk of Bias and Applicability Judgments

*QUADAS-2 is structured so that 4 key domains are each rated in terms of the risk of bias and the concern regarding applicability to the research question (as defined above). Each key domain has a set of signalling questions to help reach the judgments regarding bias and applicability.*

# DOMAIN 1: PATIENT SELECTION

## A. Risk of Bias

**Describe methods of patient selection:**This prospective study enrolled consecutive screening episodes from three English Diabetic Eye Screening Programmes (DESPs): North East London (NEL), South East London (SEL), and Gloucestershire (GS) during 2017. The target was approximately 10,000 consecutive screening episodes per centre (p.724). The final dataset comprised 30,405 consecutive screening episodes with complete human grading: 10,137 from NEL, 10,091 from GS, and 10,177 from SEL (p.725, Table 2). All images captured for each screening episode were included without editing or selection, including partial retinal images, poor quality images, and non-retinal images such as cataracts (p.725). Uptake rates ranged from 77%-83% across centres, with demographic variations reflecting local populations (35.8-66.9% White, 2.5-43.0% Asian, 1-26.3% Black) (p.724, Table 1).

Page reference: p.724-725

| Signaling Question | Answer |
| --- | --- |
| ❖ Was a consecutive or random sample of patients enrolled? | YES |
| ❖ Was a case-control design avoided? | YES |
| ❖ Did the study avoid inappropriate exclusions? | YES |

**Could the selection of patients have introduced bias?: RISK: LOW**

**Justification:** The study enrolled consecutive screening episodes from three real-world NHS diabetic eye screening programmes, representing routine clinical practice rather than a selected population. All 30,405 episodes were processed without exclusions based on image quality or other factors that could bias results. The inclusion of ungradable images (2.4% overall, ranging 1.9-3.0% across centres; p.725, Table 2) and poor-quality images reflects the actual screening population and challenges the AI system would face in clinical deployment. The multi-centre design with diverse ethnic populations (varying from predominantly White in Gloucestershire to high Asian/Black representation in London centres) enhances generalizability.

## B. Concerns regarding applicability

**Describe included patients (prior testing, presentation, intended use of index test and setting):**The study population is highly applicable to the review question regarding AI for point-of-care clinical decision support in screening contexts. Diabetic retinopathy screening is conducted in community settings at or near the point of care. The population represents real-world screening participants with Type 1 and Type 2 diabetes across diverse demographics (mean ages 60-66 years, 54-57% male, ethnically diverse populations) (p.724, Table 1). This matches the intended use case for AI-assisted triage in population screening.

**Is there concern that the included patients do not match the review question?: CONCERN: LOW**

**Justification:** The patient population is highly applicable - routine diabetic retinopathy screening participants across three diverse NHS programmes, which is the exact target for AI-assisted POC screening and triage.

# DOMAIN 2: INDEX TEST(S)

*If more than one index test was used, please complete for each test.*

## A. Risk of Bias

**Describe the index test and how it was conducted and interpreted:**The index test was EyeArt v2.1.0 (Eyenuk, Woodland Hills, CA), a CE-marked machine learning-enabled automated retinal image analysis system (p.725). The software was installed on servers at Homerton Hospital on 3 January 2017, and pseudo-anonymised images were processed on encrypted disks: NEL on 14 January 2017, SEL 6 February 2017, and GS 25 April 2017 (p.725). The AI provided a binary classification of test-positive or test-negative for each screening episode. Episodes with images classified as un-assessable (technical failure) by the software were allocated as test-positive (p.725). The patient clinical pathway was not affected by AI processing, which was undertaken in parallel with usual care. The human grade for each image was not available while images were being processed by the EyeArt software (p.725).

Page reference: p.725

| Signaling Question | Answer |
| --- | --- |
| ❖ Were the index test results interpreted without knowledge of the results of the reference standard? | YES |
| ❖ If a threshold was used, was it pre-specified? | UNCLEAR |

**Could the conduct or interpretation of the index test have introduced bias?: RISK: UNCLEAR**

**Justification:** Blinding of the AI to human reference grades was appropriately maintained, with images processed in parallel with usual care and human grades unavailable during AI processing. However, the threshold specification is unclear. While the study used CE-marked commercial software (v2.1.0), which implies regulatory-approved thresholds, the paper acknowledges that "differing software thresholding cut-offs being implemented" can affect performance comparisons across studies (p.727). The exact classification thresholds used by EyeArt are not reported, nor is it explicitly stated whether these were pre-specified prior to this evaluation or if any threshold optimization occurred. The software version was an update from prior studies (v1), suggesting potential modifications.

## B. Concerns regarding applicability

**Is there concern that the index test, its conduct, or interpretation differ from the review question?: CONCERN: LOW**

**Justification:** EyeArt v2.1.0 is a CE-marked commercial software designed specifically for automated diabetic retinopathy screening. The test was applied as intended in a real-world screening workflow, providing binary test-positive/test-negative classifications suitable for triaging patients requiring human grader review. Technical failures were appropriately classified as test-positive to ensure safety. This represents the intended use case for AI-assisted DR screening.

# DOMAIN 3: REFERENCE STANDARD

## A. Risk of Bias

**Describe the reference standard and how it was conducted and interpreted:**The reference standard was human grading performed according to NHS Diabetic Eye Screening Programme (DESP) guidelines (p.725). At least two digital image fields were captured per eye (one disc-centred, one macula-centred) per NHS DESP protocol (p.724-725). In the existing screening pathway, all images are reviewed by a primary grader, then patients with mild or worse retinopathy/maculopathy (plus 10% of "no retinopathy") are reviewed by a secondary grader, with discrepancies resolved by a tertiary arbitration grader (p.724). The final human grade in the worst eye was used as the reference standard. Grading classifications followed NHS DESP categories: R0M0 (no retinopathy), R1M0 (mild-to-moderate NPDR without referable maculopathy), R1M1 (mild-to-moderate NPDR with referable maculopathy), R2 (moderate-to-severe NPDR), R3 (proliferative DR), and U (ungradable) (p.725). The authors note that "the final human grade is a stable reference standard since measures of screening performance and accuracy of performance metrics were not materially altered when the reference standard was further refined by arbitration by an expert reading centre" in prior work (p.725).

Page reference: p.724-725

| Signaling Question | Answer |
| --- | --- |
| ❖ Is the reference standard likely to correctly classify the target condition? | YES |
| ❖ Were the reference standard results interpreted without knowledge of the results of the index test? | YES |

**Could the reference standard, its conduct, or its interpretation have introduced bias?: RISK: LOW**

**Justification:** The NHS DESP human grading protocol is a well-validated reference standard with trained graders undergoing regular quality assurance. The multi-tier grading pathway (primary, secondary, tertiary arbitration) with quality checks provides robust classification. The authors validated this approach in prior work, showing results were "not materially altered when the reference standard was further refined by arbitration by an expert reading centre" (p.725). Human graders performed assessments as part of routine care, independent of AI results, preventing review bias. The reference standard was applied uniformly across all three centres using the same NHS DESP protocol.

## B. Concerns regarding applicability

**Is there concern that the target condition as defined by the reference standard does not match the review question?: CONCERN: LOW**

**Justification:** The target condition (referable diabetic retinopathy requiring clinical review or treatment) is highly clinically relevant and directly maps to management decisions in DR screening programmes. The NHS DESP grading system with ETDRS equivalencies is a standard, validated classification system. The reference standard aligns perfectly with the intended use of AI for screening triage - identifying patients who need specialist ophthalmology review versus those who can return to routine screening.

# DOMAIN 4: FLOW AND TIMING

## A. Risk of Bias

**Describe any patients who did not receive the index test(s) and/or reference standard or who were excluded from the 2x2 table (refer to flow diagram):**All 30,405 consecutive screening episodes received both the AI index test and human grading reference standard with complete inclusion in analysis. Tables 2, 3, and 4 provide complete, transparent accounting of all episodes by retinopathy grade and classification outcomes across all three centres. No episodes were excluded from analysis after enrollment. No missing data or unexplained exclusions.

**Describe the time interval and any interventions between index test(s) and reference standard:**AI processing and human grading were performed concurrently on the same captured images. Images processed by EyeArt in January-April 2017 (p.725); human grading occurred as part of standard care during the 2017-2018 screening period (p.724, Table 1). The time interval is minimal as both tests analysed the same digital images captured during routine screening. No interventions occurred between index test and reference standard.

| Signaling Question | Answer |
| --- | --- |
| ❖ Was there an appropriate interval between index test(s) and reference standard? | YES |
| ❖ Did all patients receive a reference standard? | YES |
| ❖ Did patients receive the same reference standard? | YES |
| ❖ Were all patients included in the analysis? | YES |

**Could the patient flow have introduced bias?: RISK: LOW**

**Justification:** The study demonstrates excellent patient flow with all 30,405 consecutive screening episodes receiving both the AI index test and human grading reference standard with complete inclusion in analysis. There was no differential verification—all patients received the same standardised NHS DESP human grading pathway. The timing was appropriate as both assessments were performed on identical digital images captured during routine screening. Tables 2, 3, and 4 provide complete, transparent accounting of all episodes by retinopathy grade and classification outcomes across all three centres, with no missing data or unexplained exclusions.

# OVERALL ASSESSMENT SUMMARY

**✓ METHODOLOGICALLY STRONG STUDY: This study has been rated as LOW TO UNCLEAR overall risk of bias with LOW applicability concerns. ✓**

| Domain | Signaling Questions | Risk of Bias | Applicability |
| --- | --- | --- | --- |
| D1: Patient Selection | 1.1 Yes 1.2 Yes 1.3 Yes | LOW | LOW |
| D2: Index Test | 2.1 Yes 2.2 Unclear | UNCLEAR | LOW |
| D3: Reference Standard | 3.1 Yes 3.2 Yes | LOW | LOW |
| D4: Flow and Timing | 4.1 Yes 4.2 Yes 4.3 Yes 4.4 Yes | LOW | N/A |

**Overall Risk of Bias: LOW TO UNCLEAR**

**Justification:** This is a methodologically strong prospective validation study with low risk of bias across most domains. The consecutive enrollment of 30,405 real-world screening episodes from three diverse NHS centres, complete reference standard application to all patients, and excellent transparency in patient flow represent key strengths. The only uncertainty relates to Index Test Domain 2.2 (threshold pre-specification)—while the study used CE-marked commercial software which implies validated thresholds, the paper does not explicitly confirm the exact thresholds or whether they were pre-specified for this evaluation versus potentially optimized. This is a minor concern given the regulatory approval context.

**Overall Applicability Concern: LOW**

**Justification:** The study is highly applicable to AI-assisted diabetic retinopathy screening in point-of-care contexts. The population represents routine screening participants with diabetes across diverse UK communities. The AI system (EyeArt v2.1.0) is a commercially available, CE-marked product used as intended for triaging screening episodes. The reference standard (NHS DESP grading) directly maps to clinical management decisions. The real-world implementation across three different programmes with varying demographics, camera systems, and grading teams enhances external validity.

## Key Strengths & Limitations

**STRENGTHS (This is an exemplary study):
1. Large prospective sample:** 30,405 consecutive screening episodes from real-world programmes
**2. Multi-centre validation:** Three NHS DESPs with ethnically diverse populations (35.8-66.9% White, 2.5-43.0% Asian, 1-26.3% Black)
**3. Independent evaluation:** Not conducted by commercial partner (p.727)
**4. Zero exclusions:** All episodes included without selection - no verification bias
**5. Real-world image quality:** Included ungradable (2.4%) and poor-quality images reflecting clinical reality
**6. Transparent reporting:** Detailed tables showing complete patient flow across all centres
**7. Appropriate blinding:** Bidirectional - AI blind to human grades, graders blind to AI results
**8. Consecutive enrollment:** Eliminates selection bias

**LIMITATIONS (Minor):
1. Threshold specification unclear:** Exact AI classification thresholds not reported; pre-specification not explicitly confirmed (though CE-marked software suggests regulatory validation)
**2. Reference standard is human grading:** Not clinical outcomes or expert consensus panel; however, authors validated this approach in prior work (p.725)
**3. Centre variation:** Some performance variation across centres (specificity 65-71% for R0M0), though this reflects real-world variation
**4. Single software version:** Performance may differ with future updates

## Reported Performance (Reliable Estimates)

**Overall Performance:**• Sensitivity for referable DR: 94.7% (95% CI: 93.8-95.5%)
• Specificity for R0M0 (no retinopathy): 67.9% (95% CI: 67.3-68.5%)
• Technical failure rate: 3.0% (range 1.7-5.7% by centre)

**Centre-Specific Performance:**• North East London: Sensitivity 94.6%, Specificity 71.0%
• Gloucestershire: Sensitivity 94.2%, Specificity 65.2%
• South East London: Sensitivity 95.2%, Specificity 67.3%

**NOTE:** These performance estimates are highly reliable due to: (1) consecutive enrollment without selection bias, (2) complete verification of all episodes, (3) large sample size with narrow confidence intervals, (4) multi-centre validation, and (5) transparent reporting. The minor threshold specification uncertainty does not substantially undermine these findings.

QUADAS-2

**Iacob et al. 2025**Diagnostic Accuracy of AI-Assisted Focused Cardiac Ultrasound (FOCUS) in Primary Care *Study ID: Iacob_2025 | Assessment Date: 2025-11-28*

____________________________________________________________________________________________________

# Phase 1: State the Review Question

*Patients (setting, intended use of index test, presentation, prior testing):*

| 1780 patients aged 40-75 years from community-based primary care practices across Timis County, Romania. Setting: Primary care offices (point-of-care). Intended use: AI-assisted FOCUS for cardiac pathology screening/triage in primary care to identify patients requiring specialist referral. Presentation: Patients meeting dual inclusion criteria - either high estimated cardiovascular risk (SCORE2-OP ≥10%) OR clinical symptoms suggestive of cardiac pathology (most commonly dyspnea or chest discomfort). Prior testing: None specified. Exclusions: Known prior cardiovascular disease (to avoid confounding), morbid obesity (BMI ≥40 kg/m²), severe pulmonary disease, or other causes of inadequate acoustic windows. Population characteristics: Age 40-75 years, high CV risk region (Romania has one of highest CV mortalities in Europe), enriched population with elevated risk scores or symptoms. Disease prevalence: 32.9% had at least one target cardiac pathology (585/1780: LVEF <50%, valvular abnormalities, or pericardial effusion). NOTE: Enrollment method (consecutive vs. random) NOT explicitly stated. |
| --- |

*Index test(s):*

| AI-assisted Focused Cardiac Ultrasound (AI-FOCUS) performed by family physicians using Sonoscape-P60 ultrasound system equipped with Wis+ AI platform. Operators: Family physicians after standardized 6-month training curriculum including didactic seminars, supervised scanning with >50 documented patient examinations, and competency assessment covering image acquisition and interpretation. Protocol: Five standardized acoustic views - subxiphoid (pericardial effusion/IVC), parasternal long-axis, parasternal short-axis, apical four-chamber (AI-assisted LVEF via Simpson's biplane), and subcostal IVC. Three prespecified target conditions: (1) LVEF <50% (AI-computed Simpson's biplane, binary abnormal/normal), (2) Valvular abnormalities (≥mild regurgitation or suspected stenosis on 2D/Doppler, present/absent), (3) Pericardial effusion (any echo-free space, present/absent). AI processing: Wis+ AI module automatically processed cine loops using convolutional neural network for endocardial border segmentation and computed LVEF, flagging abnormal when LVEF <50%. Also provided speckle-tracking wall-motion support and Doppler-aided valvular screening. Family physician reviewed and recorded AI output without alteration. Proprietary AI model NOT modified or retrained. Thresholds: Pre-specified based on clinical guidelines (LVEF <50% per ASE/EACVI standards). |
| --- |

*Reference standard and target condition:*

| Reference standard: Comprehensive transthoracic echocardiography (TTE) performed by a cardiologist with ≥10 years of experience and active ASE/EACVI certification. Cardiologist was BLINDED to FOCUS results and patient clinical history. Reference TTE performed within 24 hours of AI-FOCUS exam (mean interval 12 hours, range 2-23 hours). Protocol followed ASE/EACVI guidelines with complete standard views including parasternal long/short-axis, apical four-chamber/two-chamber/three-chamber, and subcostal windows. LVEF quantified using Simpson's biplane method. Valvular assessment included 2D, color Doppler, and spectral Doppler per guidelines. Same diagnostic thresholds applied: LVEF <50%, valvular abnormalities (≥mild regurgitation or any stenosis), pericardial effusion (any measurable). Target condition: Binary patient-level classification - "any target cardiac pathology present" (LVEF <50% OR valvular abnormality OR pericardial effusion) vs. "absent" (none of the three). All 1780 patients received identical reference standard by same blinded cardiologist. |
| --- |

# Phase 2: Draw a Flow Diagram for the Primary Study

| PATIENT FLOW DIAGRAM  PROSPECTIVE CROSS-SECTIONAL STUDY Setting: Community-based primary care practices, Timis County, Romania Study Period: Not explicitly stated in paper  ↓ ENROLLED PATIENTS (n = 1,780) Age 40-75 years from primary care practices  INCLUSION CRITERIA (DUAL): ├─→ High estimated cardiovascular risk (SCORE2-OP ≥ 10%) OR └─→ Clinical symptoms suggestive of cardiac pathology (dyspnea, chest discomfort)  EXCLUSIONS (clinically appropriate): ├─→ Known prior cardiovascular disease (avoid confounding) ├─→ Morbid obesity (BMI ≥ 40 kg/m²) (acoustic window limitation) ├─→ Severe pulmonary disease (acoustic window limitation) └─→ Other causes of inadequate acoustic windows  NOTE: Enrollment method (consecutive vs. random) NOT explicitly stated  ↓ ═══════════════════════════════════════════════════════════════════════ ALL 1,780 PATIENTS RECEIVED BOTH INDEX TEST & REFERENCE STANDARD ═══════════════════════════════════════════════════════════════════════  INDEX TEST (AI-FOCUS): Performed by family physicians (after 6-month training) Equipment: Sonoscape-P60 with Wis+ AI platform Protocol: ├─→ 5 standardized views (subxiphoid, parasternal LA/SA, apical 4C, subcostal IVC) ├─→ AI-computed LVEF (Simpson's biplane) - threshold <50% ├─→ Valvular assessment (≥mild regurgitation or stenosis) └─→ Pericardial effusion (any echo-free space) Output: Binary for each condition + overall "any pathology present/absent" Family physician recorded AI output WITHOUT alteration  ↓ REFERENCE STANDARD (Cardiologist TTE): Within 24 hours (mean 12h, range 2-23h) Performed by: Cardiologist with ≥10 years experience, ASE/EACVI certified BLINDED: Unaware of FOCUS results AND patient clinical history Protocol: ASE/EACVI guideline-compliant comprehensive TTE ├─→ Complete standard views (parasternal LA/SA, apical 4C/2C/3C, subcostal) ├─→ LVEF quantified via Simpson's biplane (<50% = abnormal) ├─→ Valvular assessment (2D + color + spectral Doppler) └─→ Pericardial effusion assessment Same diagnostic thresholds as index test  ↓ ═══════════════════════════════════════════════════════════════════════ COMPLETE 2×2 ANALYSIS (n = 1,780) - NO EXCLUSIONS ═══════════════════════════════════════════════════════════════════════  PATIENT-LEVEL BINARY CLASSIFICATION: "Any target cardiac pathology" = LVEF <50% OR valvular abnormality OR   pericardial effusion  RESULTS: ├─→ True Positives (TP): 526 patients ├─→ False Positives (FP): 42 patients ├─→ True Negatives (TN): 1,153 patients └─→ False Negatives (FN): 59 patients  TOTAL: 526 + 42 + 1,153 + 59 = 1,780 ✓  DISEASE PREVALENCE: • Any target pathology: 585/1,780 = 32.9% • No pathology: 1,195/1,780 = 67.1%  REPORTED PERFORMANCE: • Sensitivity: 89.9% (95% CI: 87.3-92.5%)  [526/(526+59) = 526/585] • Specificity: 96.5% (95% CI: 95.4-97.6%)  [1,153/(1,153+42) = 1,153/1,195] • PPV: 92.6% • NPV: 95.1%  CONDITION-SPECIFIC PERFORMANCE: • LVEF <50%: Sensitivity 88%, Specificity 97% • Valvular abnormalities: Sensitivity 85%, Specificity 94% • Pericardial effusion: Sensitivity 100%, Specificity 99%  MISCLASSIFICATIONS: • Concentrated in borderline cases: LVEF 45-50%, mild valvular regurgitation • Authors acknowledge AI performance may be worse at clinical decision thresholds  KEY METHODOLOGICAL STRENGTHS: 1. All 1,780 patients received both tests 2. Uniform reference standard (same blinded cardiologist) 3. Short interval (≤24 hours, mean 12h) 4. Complete verification - no partial/differential verification 5. Pre-specified thresholds based on guidelines 6. Appropriate blinding (cardiologist blind to FOCUS + clinical history)  KEY LIMITATION: • Enrollment method (consecutive vs. random) NOT explicitly stated |
| --- |

# Phase 3: Risk of Bias and Applicability Judgments

*QUADAS-2 is structured so that 4 key domains are each rated in terms of the risk of bias and the concern regarding applicability to the research question (as defined above). Each key domain has a set of signalling questions to help reach the judgments regarding bias and applicability.*

# DOMAIN 1: PATIENT SELECTION

## A. Risk of Bias

**Describe methods of patient selection:**This prospective, observational cross-sectional study enrolled 1780 patients aged 40-75 years from community-based primary care practices across Timis County, Romania (p.5). All participants met dual inclusion criteria: either high estimated cardiovascular risk (SCORE2-OP ≥10%) OR clinical symptoms suggestive of cardiac pathology, most commonly dyspnea or chest discomfort (p.5). The study explicitly excluded patients with known prior cardiovascular disease, morbid obesity (BMI ≥40 kg/m²), severe pulmonary disease, or other causes of potentially inadequate acoustic windows to avoid confounding from pre-established diagnoses and ensure technical feasibility of image acquisition (p.3). The study does not state whether enrollment was consecutive or random, though it refers to "community-based primary care practices" and describes the population as representing "real-world diagnostic dilemmas" (p.3). The study was conducted in Romania, which has one of the highest overall cardiovascular mortalities in Europe (p.3).

Page reference: p.3, p.5

| Signaling Question | Answer |
| --- | --- |
| ❖ Was a consecutive or random sample of patients enrolled? | UNCLEAR |
| ❖ Was a case-control design avoided? | YES |
| ❖ Did the study avoid inappropriate exclusions? | YES |

**Could the selection of patients have introduced bias?: RISK: UNCLEAR**

**Justification:** While the study used a prospective cohort design (avoiding case-control) and had appropriate exclusion criteria, there is insufficient information to determine whether enrollment was consecutive or random. The paper states patients were enrolled from "community-based primary care practices" meeting specific risk/symptom criteria, but the actual enrollment mechanism (consecutive vs. convenience sampling) is not specified (p.3, p.5). This is a critical gap because non-consecutive/non-random enrollment could introduce selection bias, potentially over-representing patients with more obvious symptoms or excluding patients seen at inconvenient times. The large sample size (n=1780) and single-region recruitment raise questions about whether all eligible patients were approached systematically.

## B. Concerns regarding applicability

**Describe included patients (prior testing, presentation, intended use of index test and setting):**The study population is highly applicable to the review question concerning AI in point-of-care imaging for clinical decision support. The study was conducted in primary care settings with family physicians (p.3), evaluating patients with high cardiovascular risk scores or cardiac symptoms—exactly the population where point-of-care cardiac imaging would be clinically relevant. The Romanian primary care context, while potentially limiting generalizability to other healthcare systems, represents a real-world implementation scenario for AI-FOCUS triage. The age range (40-75 years) and cardiovascular risk profile (SCORE2-OP ≥10%) are appropriate for cardiac screening applications.

**Is there concern that the included patients do not match the review question?: CONCERN: LOW**

**Justification:** The patient population is highly applicable - primary care patients with elevated CV risk or cardiac symptoms requiring triage for potential specialist referral, which is the exact target for AI-assisted POC cardiac imaging.

# DOMAIN 2: INDEX TEST(S)

*If more than one index test was used, please complete for each test.*

## A. Risk of Bias

**Describe the index test and how it was conducted and interpreted:**The index test was AI-assisted Focused Cardiac Ultrasound (AI-FOCUS) performed by family physicians using a Sonoscape-P60 ultrasound system equipped with the Wis+ AI platform (p.3). All family physicians underwent a standardized 6-month training curriculum that included didactic seminars, supervised scanning with >50 documented patient examinations, and a competency assessment covering image acquisition (standard views) and interpretation (LVEF, basic valvular assessment, pericardial effusion) (p.3). The FOCUS protocol included five standardized acoustic views: subxiphoid, parasternal long and short axis, apical four-chamber (AI-assisted LVEF quantification using Simpson's biplane method), and subcostal IVC assessment (p.3-4). Three prespecified target conditions were assessed: (1) LVEF <50% (AI-computed Simpson's biplane; binary abnormal/normal), (2) valvular abnormalities (≥mild regurgitation or suspected stenosis on 2D/Doppler, recorded as present/absent), and (3) pericardial effusion (any echo-free space, present/absent) (p.3). The Wis+ AI module automatically processed cine loops using a convolutional neural network for endocardial border segmentation and computed LVEF (Simpson's biplane), flagging abnormal results when LVEF <50% (p.4). The family physician reviewed and recorded the AI output but did not alter it (p.4). The authors did not modify or retrain the proprietary AI model (p.4).

Page reference: p.3-4

| Signaling Question | Answer |
| --- | --- |
| ❖ Were the index test results interpreted without knowledge of the results of the reference standard? | YES |
| ❖ If a threshold was used, was it pre-specified? | YES |

**Could the conduct or interpretation of the index test have introduced bias?: RISK: LOW**

**Justification:** The index test was conducted with appropriate blinding—family physicians recorded AI-FOCUS results before the blinded cardiologist performed reference echocardiography (p.4, Figure 1). The AI algorithm itself is a fixed proprietary model (Wis+) that was not modified or retrained on the study data, eliminating concerns about optimization bias at the algorithm level (p.4). Thresholds were clinically pre-specified based on established guidelines (LVEF <50% per ASE/EACVI standards), not optimized on the study population (p.3-4). The standardized 6-month training protocol with competency assessment ensured consistent image acquisition across family physicians (p.3). This represents appropriate methodology for evaluating a diagnostic AI system.

## B. Concerns regarding applicability

**Is there concern that the index test, its conduct, or interpretation differ from the review question?: CONCERN: LOW**

**Justification:** The index test is highly applicable to the review question. AI-FOCUS was performed by non-specialist family physicians after structured training, representing the intended real-world use case for point-of-care cardiac imaging with AI assistance (p.3). The Sonoscape-P60 with Wis+ AI platform is a commercially available system, not a research prototype (p.3). The five-view FOCUS protocol and target conditions (LVEF, valvular abnormalities, pericardial effusion) are standard clinical applications for cardiac point-of-care ultrasound. The primary care setting in a high-cardiovascular-risk region (Romania) is appropriate for evaluating triage utility.

# DOMAIN 3: REFERENCE STANDARD

## A. Risk of Bias

**Describe the reference standard and how it was conducted and interpreted:**The reference standard was comprehensive transthoracic echocardiography (TTE) performed by a cardiologist with ≥10 years of experience and active ASE/EACVI certification (p.4). Critically, the cardiologist was BLINDED to both the FOCUS results and patient clinical history (p.4). Reference TTE was performed within 24 hours of the AI-FOCUS examination (mean interval 12 hours, range 2-23 hours) (p.3). The protocol followed ASE/EACVI guidelines with complete standard views including parasternal long-axis, parasternal short-axis, apical four-chamber, apical two-chamber, apical three-chamber, and subcostal windows (p.4). LVEF was quantified using Simpson's biplane method, valvular assessment included 2D, color Doppler, and spectral Doppler per guidelines, and the same diagnostic thresholds were applied: LVEF <50%, valvular abnormalities (≥mild regurgitation or any stenosis), and pericardial effusion (any measurable) (p.3-4). All 1780 patients received the identical reference standard performed by the same blinded cardiologist.

Page reference: p.3-4

| Signaling Question | Answer |
| --- | --- |
| ❖ Is the reference standard likely to correctly classify the target condition? | YES |
| ❖ Were the reference standard results interpreted without knowledge of the results of the index test? | YES |

**Could the reference standard, its conduct, or its interpretation have introduced bias?: RISK: LOW**

**Justification:** The reference standard is appropriate and was conducted with excellent methodological rigor. Comprehensive TTE performed by an experienced, credentialed cardiologist following ASE/EACVI guidelines is the gold standard for cardiac structural and functional assessment (p.4). The cardiologist was blinded to both FOCUS results AND patient clinical history, preventing both review bias and clinical context bias (p.4). The uniform application of the reference standard to all 1780 patients by the same cardiologist eliminates inter-observer variability and differential verification. The short time interval (mean 12 hours, maximum 24 hours) ensures clinical stability between tests. The use of identical diagnostic thresholds for both index and reference tests (LVEF <50%, ≥mild valvular regurgitation, any pericardial effusion) is methodologically sound.

## B. Concerns regarding applicability

**Is there concern that the target condition as defined by the reference standard does not match the review question?: CONCERN: LOW**

**Justification:** The target conditions (LVEF <50%, valvular abnormalities, pericardial effusion) assessed by comprehensive TTE are clinically relevant and directly applicable to the review question. These are standard cardiac pathologies requiring specialist evaluation and management. The binary patient-level classification ("any target cardiac pathology present vs. absent") is appropriate for a triage/screening application. Comprehensive TTE by a credentialed cardiologist represents the accepted clinical standard for diagnosing these conditions. The thresholds used (LVEF <50% per ASE/EACVI guidelines) align with established clinical decision points.

# DOMAIN 4: FLOW AND TIMING

## A. Risk of Bias

**Describe any patients who did not receive the index test(s) and/or reference standard or who were excluded from the 2x2 table (refer to flow diagram):**All 1780 enrolled patients received both AI-FOCUS (index test) and cardiologist TTE (reference standard) with complete inclusion in the 2×2 analysis (p.3-4). The 2×2 table arithmetic verifies complete data: TP=526 + FP=42 + TN=1,153 + FN=59 = 1,780 patients (p.6). No patients were excluded from analysis after enrollment. The paper does not provide a STARD/CONSORT flow diagram, which limits verification of any technically inadequate scans, though image adequacy was recorded as part of the protocol (p.3).

**Describe the time interval and any interventions between index test(s) and reference standard:**The reference TTE was performed within 24 hours of each AI-FOCUS examination (mean interval 12 hours, range 2-23 hours) (p.3). This short time interval ensures clinical stability and prevents interval changes in cardiac status that could affect diagnostic concordance. No interventions or treatments were initiated between the index test and reference standard. Both tests analyzed the same clinical state.

| Signaling Question | Answer |
| --- | --- |
| ❖ Was there an appropriate interval between index test(s) and reference standard? | YES |
| ❖ Did all patients receive a reference standard? | YES |
| ❖ Did patients receive the same reference standard? | YES |
| ❖ Were all patients included in the analysis? | YES |

**Could the patient flow have introduced bias?: RISK: LOW**

**Justification:** The study demonstrates excellent patient flow. All 1780 patients received both the index test and reference standard within an appropriate time interval (≤24 hours, mean 12 hours), preventing interval changes in clinical status. There was no differential verification—all patients received the identical reference standard performed by the same blinded cardiologist following ASE/EACVI guidelines. Complete inclusion in analysis is verified by the 2×2 table arithmetic (TP+FP+TN+FN=1,780). The absence of a flow diagram is a documentation limitation but does not indicate actual exclusions, as the complete dataset accounting is provided.

# OVERALL ASSESSMENT SUMMARY

**✓ WELL-DESIGNED STUDY: This study has been rated as LOW-MODERATE overall risk of bias with LOW applicability concerns. ✓**

| Domain | Signaling Questions | Risk of Bias | Applicability |
| --- | --- | --- | --- |
| D1: Patient Selection | 1.1 Unclear 1.2 Yes 1.3 Yes | UNCLEAR | LOW |
| D2: Index Test | 2.1 Yes 2.2 Yes | LOW | LOW |
| D3: Reference Standard | 3.1 Yes 3.2 Yes | LOW | LOW |
| D4: Flow and Timing | 4.1 Yes 4.2 Yes 4.3 Yes 4.4 Yes | LOW | N/A |

**Overall Risk of Bias: LOW-MODERATE**

**Justification:** This is a methodologically strong diagnostic accuracy study with low risk of bias in three of four domains (Index Test, Reference Standard, Flow and Timing). The only uncertain domain is Patient Selection, where the absence of explicit documentation of consecutive or random enrollment creates uncertainty about potential selection bias. The study strengths are substantial: prospective design with 1780 patients, pre-specified thresholds based on clinical guidelines, blinded cardiologist reference standard, uniform verification of all patients with the same reference standard within 24 hours, and complete inclusion of all patients in analysis. The single "Unclear" in Domain 1 results in an overall classification between Low and Moderate risk. Per QUADAS-2 guidance (1 Unclear = Low-Moderate; the study has 0 High ratings), this study represents above-average methodological quality for a diagnostic accuracy study, though the enrollment method should have been explicitly documented.

**Overall Applicability Concern: LOW**

**Justification:** The study is highly applicable to AI-assisted point-of-care cardiac imaging in primary care settings. The population (primary care patients with elevated CV risk or symptoms), setting (family physician offices), index test (AI-FOCUS performed by trained non-specialists), and reference standard (comprehensive TTE) all match the intended use case for POC cardiac screening and triage. The commercially available Wis+ AI platform on Sonoscape-P60 represents a realistic implementation scenario. The target conditions (LVEF <50%, valvular abnormalities, pericardial effusion) are clinically relevant and directly inform management decisions. External validation in diverse populations is needed given the single-region Romanian cohort.

## Key Strengths & Limitations

**STRENGTHS:
1. Large prospective cohort:** n=1780 in target population (primary care patients with high CV risk or cardiac symptoms)
**2. Uniform reference standard:** All patients received identical cardiologist TTE per ASE/EACVI guidelines
**3. Appropriate blinding:** Cardiologist blinded to FOCUS results AND clinical history; AI algorithm fixed/not retrained
**4. Pre-specified clinical thresholds:** Based on established guidelines (LVEF <50% per ASE/EACVI)
**5. Short interval:** ≤24 hours (mean 12h) between index test and reference standard
**6. Complete data:** All 1780 patients included in 2×2 analysis with no unexplained exclusions
**7. Standardized operator training:** 6-month curriculum with >50 supervised scans and competency assessment

**LIMITATIONS:
1. Enrollment method unclear:** Paper does not explicitly state consecutive vs. random enrollment (selection bias cannot be ruled out)
**2. No STARD/CONSORT flow diagram:** Limits verification of patient flow and any technically inadequate scans
**3. Single geographic region:** Timis County, Romania - may limit generalizability
**4. Predominantly Caucasian population:** Authors acknowledge algorithm bias cannot be excluded; external validation needed across diverse populations
**5. Single AI platform:** Wis+ on Sonoscape-P60 - results may not generalize to other AI systems
**6. Borderline case limitations:** Misclassifications concentrated in borderline LVEF (45-50%) and mild valvular regurgitation - AI may perform worse at clinical decision thresholds

## Reported Performance (Reliable Estimates)

**Patient-Level Binary Classification ("Any Target Pathology"):**• Sensitivity: 89.9% (95% CI: 87.3-92.5%)
• Specificity: 96.5% (95% CI: 95.4-97.6%)
• PPV: 92.6%
• NPV: 95.1%
• Disease prevalence: 32.9% (585/1,780)

**Condition-Specific Performance:**• LVEF <50%: Sensitivity 88%, Specificity 97%
• Valvular abnormalities: Sensitivity 85%, Specificity 94%
• Pericardial effusion: Sensitivity 100%, Specificity 99%

**NOTE:** Performance estimates are likely reliable due to: (1) large sample size with narrow CIs, (2) complete verification without bias, (3) pre-specified thresholds, (4) appropriate blinding, and (5) uniform reference standard. The enrollment method uncertainty is a documentation gap but does not fundamentally undermine the validity of these findings within the enrolled cohort.

QUADAS-2

**Jaremko et al. 2023**AI aided workflow for hip dysplasia screening using ultrasound in primary care clinics *Study ID: Jaremko_2023 | Assessment Date: 2025-11-28*

____________________________________________________________________________________________________

# Phase 1: State the Review Question

*Patients (setting, intended use of index test, presentation, prior testing):*

| Infants presenting for routine wellness-check visits at 3 fee-for-service primary care clinics in towns within 150 km of a referral city in Canada, February 1, 2021 to March 31, 2022. Setting: Point-of-care - primary care clinics serving populations of ~80,000-100,000. Intended use: AI-assisted screening for developmental dysplasia of the hip (DDH) during routine wellness visits to identify infants requiring specialist referral. Presentation: Healthy infants at routine wellness checks. Enrollment: Following ACR guidelines, study sought to scan all infants presenting to clinics at least once between 4-16 weeks of age (preference for younger infants as scans technically easier). Prior testing: None. Population: 306 unique infants (369 total scans, 63 repeat scans). Mean age 45 days (range 3-193 days), 51% female, 9% breech deliveries, 9% positive family history of DDH, 10% Indigenous. Exclusions: 18/306 infants (5.9%) could not be scanned (typically large or uncooperative infants near upper age limits). Disease prevalence: 6 confirmed DDH cases requiring treatment (2.0% of scanned population). NOTE: Enrollment method (consecutive vs. random) not explicitly stated. |
| --- |

*Index test(s):*

| MEDO-Hip AI application (MEDO.ai, Edmonton, Canada, 2021) - FDA-cleared commercial product. Equipment: Philips Lumify handheld 12 MHz linear probes connected to Samsung S7 tablets. AI methodology: U-Net-like CNN model for segmentation of anatomic landmarks (acetabulum, femoral head), followed by automated geometric analysis measuring alpha angle and femoral head coverage. Classification: Combined with heuristics based on Graf methodology to classify hips as Healthy, FU Recommended, or Inconclusive/Repeat Scan. Training data: CNN previously trained on 10,572 femoral head and 16,657 acetabulum labeled images from 4 centers, with 80/20 training/validation split disjoint at patient level. Operators: Non-expert clinic staff - 2 RNs (158 infants), 4 LPNs (49 infants), 4 physicians (65 infants), 3 sonographers (24 infants) - after training via video, PowerPoint slides, and brief in-person demonstration with 2-4 supervised scanning days. Results: Returned to tablet typically in <1 minute. Thresholds: Pre-specified as part of FDA-cleared product developed prior to this implementation study; based on Graf methodology. No threshold optimization performed on study population. |
| --- |

*Reference standard and target condition:*

| CRITICAL LIMITATION - DIFFERENTIAL VERIFICATION: The reference standard pathway was tiered and only 6/306 infants (2.0%) received gold standard assessment. Pathway: (1) When AI recommended follow-up or indicated suboptimal scans, internal follow-up scan performed by study sonographer using SAME AI app (when available). (2) Cases remaining abnormal after internal follow-up referred to pediatric orthopedic clinic. (3) Final reference standard for positive cases (N=6, 2.0%): Pediatric orthopedic specialist assessment leading to DDH treatment decisions (5 Pavlik harness, 1 closed reduction). SEVERE BIAS: Infants classified as Normal by AI (N=244, 80%) received NO further imaging or orthopedic evaluation. Infants initially flagged but cleared at internal sonographer follow-up (N=26, 8.2%) also received NO orthopedic referral. Gold standard orthopedic assessment only applied to 6 infants (2%) who remained positive through all screening levels. Target condition: Developmental dysplasia of the hip (DDH) requiring treatment. FUNDAMENTAL FLAW: This creates tautological reference for negatives - the AI negative classification itself becomes the reference standard. False negatives cannot be detected because normal AI results did not receive independent expert verification. Authors acknowledge: Diagnostic performance could only be assessed on a limited basis, because it was not feasible to also perform gold-standard conventional ultrasound on all babies. |
| --- |

# Phase 2: Draw a Flow Diagram for the Primary Study

| PATIENT FLOW DIAGRAM - IMPLEMENTATION STUDY  ENROLLMENT: February 1, 2021 - March 31, 2022 Setting: 3 primary care clinics, Canada  ↓ ELIGIBLE INFANTS: Routine wellness visits, 4-16 weeks age  ↓ ENROLLED: 306 unique infants (369 total scans)  ↓ EXCLUSIONS: 18 (5.9%) could not be scanned  ↓ SUCCESSFULLY SCANNED: 288 infants  INDEX TEST (All 288): MEDO-Hip AI app Operators: Non-expert staff (RNs, LPNs, physicians) Results: <1 min turnaround  DIFFERENTIAL VERIFICATION PATHWAY (SEVERE BIAS):  PATHWAY 1: AI "Normal" (n=244, 80%)  → NO REFERENCE STANDARD  → NO orthopedic assessment  → NO expert ultrasound  → FALSE NEGATIVES UNDETECTABLE  PATHWAY 2: AI Flagged (n=44)  → Internal FU with SAME AI (INCORPORATION BIAS)  → Cleared at FU (n=38)  → NO orthopedic referral  → NO GOLD STANDARD  PATHWAY 3: Orthopedic Referral (n=6, 2%)  → GOLD STANDARD (pediatric orthopedist)  → 6/6 (100%) required treatment  VERIFICATION SUMMARY: • Gold standard: 6/306 (2%) • No verification: 300/306 (98%)  CRITICAL FLAW: Reference determined BY index test • AI negative → No verification → Counted as true negative • False negatives UNDETECTABLE  Authors acknowledge: "Diagnostic performance could only be  assessed on a limited basis"  IMPLEMENTATION FINDINGS (Valuable despite bias): • Real-world feasibility demonstrated • Follow-up rate declined: 40% → 14% with experience • 4/6 DDH cases had NO risk factors • PPV for referral: 100% (6/6 treated) |
| --- |

# Phase 3: Risk of Bias and Applicability Judgments

# DOMAIN 1: PATIENT SELECTION

## A. Risk of Bias

**Describe methods of patient selection:**This implementation study enrolled infants presenting for routine wellness-check visits at 3 fee-for-service primary care clinics in Canada, February 1, 2021 to March 31, 2022. Following ACR guidelines, the study sought to scan all infants presenting to clinics at least once between 4-16 weeks of age. A total of 369 scans were performed in 306 unique patients. Of these, 18/306 infants (5.9%) could not be scanned. The enrollment appeared consecutive based on wellness visits, though not explicitly stated. Population had mean age 45 days, 51% female, 9% breech, 9% family history DDH, 10% Indigenous. Page reference: p.3, p.4

| Signaling Question | Answer |
| --- | --- |
| ❖ Was a consecutive or random sample enrolled? | UNCLEAR |
| ❖ Was a case-control design avoided? | YES |
| ❖ Did study avoid inappropriate exclusions? | YES |

**Could the selection of patients have introduced bias?: RISK: UNCLEAR**

**Justification:** While the study enrolled infants from routine wellness visits across 3 primary care clinics, which appears to be a consecutive or near-consecutive sample, the enrollment method is not explicitly described. The study aimed to scan all infants presenting to the clinic within the target age range, but details on whether all eligible infants were actually offered enrollment are not provided. The 18 excluded patients (5.9%) had appropriate technical exclusions. The lack of explicit confirmation of consecutive enrollment prevents a Low Risk judgment, but there is no evidence of cherry-picking that would warrant High Risk.

## B. Concerns regarding applicability

**Is there concern that the included patients do not match the review question?: CONCERN: LOW**

**Justification:** This population matches the review question exceptionally well. The study enrolled infants at routine primary care wellness visits in fee-for-service clinics serving populations of ~80,000-100,000. This represents a real-world point-of-care screening population with typical prevalence of risk factors.

# DOMAIN 2: INDEX TEST(S)

## A. Risk of Bias

**Describe the index test:**FDA-cleared MEDO-Hip AI application using Philips Lumify handheld ultrasound connected to Samsung S7 tablets. AI uses U-Net-like CNN for segmentation of anatomic landmarks, followed by automated geometric analysis based on Graf methodology. Performed by non-expert clinic staff after minimal training. Results returned in <1 minute. Thresholds pre-specified as part of FDA-cleared product. Page reference: p.1-3

| Signaling Question | Answer |
| --- | --- |
| ❖ Interpreted without knowledge of reference? | YES |
| ❖ If threshold used, was it pre-specified? | YES |

**Could the conduct of the index test have introduced bias?: RISK: LOW**

**Justification:** The AI classification was performed in real-time at point of initial scanning, clearly before any reference standard was obtained. The automated algorithm cannot incorporate knowledge of later diagnostic findings. The thresholds were pre-specified as part of the FDA-cleared product. No threshold optimization was performed on this study population.

## B. Concerns regarding applicability

**Is there concern about the index test applicability?: CONCERN: LOW**

**Justification:** The index test is highly applicable. This is an FDA-cleared AI decision support system used at point-of-care in primary care clinics by non-expert users with minimal training. The handheld portable ultrasound and tablet-based workflow represent exactly the type of AI-assisted point-of-care imaging system relevant to this review.

# DOMAIN 3: REFERENCE STANDARD

## A. Risk of Bias

**Describe the reference standard:**SEVERE DIFFERENTIAL VERIFICATION: Only 6/306 infants (2.0%) received gold standard pediatric orthopedic specialist assessment. The 244 infants (80%) classified as Normal by AI received NO further imaging or orthopedic evaluation. Additional 38 infants initially flagged but cleared at internal follow-up (using same AI) also received NO orthopedic referral. Internal follow-up used same MEDO-Hip AI app (incorporation bias). Gold standard only applied to 6 infants who remained positive through all screening levels. Page reference: p.3, p.4

| Signaling Question | Answer |
| --- | --- |
| ❖ Is reference standard likely to correctly classify? | NO |
| ❖ Interpreted without knowledge of index test? | NO |

**Could the reference standard have introduced bias?: RISK: HIGH**

**Justification:** SEVERE DIFFERENTIAL VERIFICATION BIAS is present. Only 6/306 infants (2.0%) received gold standard reference. The remaining 300/306 infants (98%) were classified based on AI alone or AI-assisted sonographer follow-up. This creates a tautological reference for negative cases: the AI negative classification itself becomes the reference standard. False negatives cannot be detected. Additionally, INCORPORATION BIAS exists: internal follow-up used the same AI system. The study design fundamentally cannot assess diagnostic accuracy.

## B. Concerns regarding applicability

**Is there concern about reference standard applicability?: CONCERN: LOW**

**Justification:** For the 6 infants who received it, the reference standard (pediatric orthopedic specialist assessment with treatment decisions) is appropriate and clinically relevant for DDH diagnosis. However, this appropriate reference was only applied to 2% of the study population, creating severe verification bias rather than an applicability concern.

# DOMAIN 4: FLOW AND TIMING

## A. Risk of Bias

**Patient flow:**SEVERE PARTIAL VERIFICATION BIAS: All 306 infants received index test, but only 6/306 (2.0%) received gold standard reference. The 244 infants classified as Normal by AI received NO reference standard verification - no orthopedic exam, no expert ultrasound, no independent imaging. Additional 38 initially flagged were cleared at internal follow-up and received NO independent verification. Thus 300/306 infants (98%) had NO gold standard reference applied. Page reference: p.4

| Signaling Question | Answer |
| --- | --- |
| ❖ Appropriate interval between tests? | UNCLEAR |
| ❖ Did all patients receive reference standard? | NO |
| ❖ Did patients receive same reference standard? | NO |
| ❖ Were all patients included in analysis? | NO |

**Could the patient flow have introduced bias?: RISK: HIGH**

**Justification:** SEVERE PARTIAL AND DIFFERENTIAL VERIFICATION BIAS dominates. Only 6/306 infants (2.0%) received gold standard reference, while 300/306 (98%) received no independent verification. Reference standard pathway was determined by index test result. This creates three different verification pathways, fundamentally preventing valid diagnostic accuracy assessment. Authors acknowledge: Diagnostic performance could only be assessed on a limited basis. False negatives are completely undetectable in this design.

# OVERALL ASSESSMENT SUMMARY

**HIGH RISK OF BIAS - IMPLEMENTATION STUDY WITH SEVERE VERIFICATION BIAS**

| Domain | Signaling Questions | Risk of Bias | Applicability |
| --- | --- | --- | --- |
| D1: Patient Selection | 1.1 Unclear 1.2 Yes 1.3 Yes | UNCLEAR | LOW |
| D2: Index Test | 2.1 Yes 2.2 Yes | LOW | LOW |
| D3: Reference Standard | 3.1 No 3.2 No | HIGH | LOW |
| D4: Flow and Timing | 4.1 Unclear 4.2 No 4.3 No 4.4 No | HIGH | N/A |

**Overall Risk of Bias: HIGH**

**Justification:** This implementation study has HIGH risk of bias due to severe differential verification bias. Only 6/306 infants (2.0%) received gold standard pediatric orthopedic assessment. The 244 infants (80%) classified as Normal by AI received no reference standard verification. False negatives are undetectable. Authors acknowledge: Diagnostic performance could only be assessed on a limited basis. The internal follow-up process used the same AI system, creating incorporation bias. While the study provides useful implementation data (100% treatment rate among referrals, declining follow-up rates with experience), it cannot provide valid estimates of sensitivity, specificity, or diagnostic accuracy.

**Overall Applicability Concern: LOW**

**Justification:** Despite high risk of bias, this study has excellent applicability. Conducted in real-world primary care clinics (not academic centers), used non-expert operators with minimal training, employed FDA-cleared handheld ultrasound with automated AI interpretation, and integrated screening into routine wellness visits. The population, setting, index test implementation, and clinical workflow are all highly representative of how AI-assisted point-of-care screening would be deployed in practice.

## Critical Biases & Study Value

**SEVERE BIASES (Prevent valid diagnostic accuracy assessment):**1. VERIFICATION BIAS: Only 6/306 (2%) received gold standard
2. DIFFERENTIAL VERIFICATION: Three pathways based on index test
3. INCORPORATION BIAS: Follow-up used same AI
4. FALSE NEGATIVES UNDETECTABLE: 244 Normal patients unverified

**IMPLEMENTATION VALUE (Despite methodological limitations):**• Real-world feasibility demonstrated
• PPV for referral: 100% (6/6 treated)
• Follow-up rate declined 40%→14% with experience
• 4/6 DDH cases had NO traditional risk factors
• Workflow integration successful (scan time ~7-11 min total)

QUADAS-2

**Jayaraman et al. 2025**Artificial intelligence as a proficient tool in detecting pulmonary tuberculosis in massive population screening programs: a case study in Chennai, India *Study ID: Jayaraman_2025 | Assessment Date: 2025-11-28*

____________________________________________________________________________________________________

# Phase 1: State the Review Question

*Patients (setting, intended use of index test, presentation, prior testing):*

| Real-world population screening in Chennai, India. Total 25,598 patients screened January-December 2022 using four mobile diagnostic units (MDUs) deployed in remote/low-resource areas. Setting: Point-of-care - mobile diagnostic vans bringing TB screening to remote populations in high TB burden area. Intended use: Genki v1 AI-powered CADe software for automated chest X-ray analysis to detect tuberculosis. Presentation: Patients from remote areas visited mobile vans, completed registration, underwent chest radiography. Population: Mass chest radiography screening program including symptomatic and asymptomatic individuals. Demographics: 49.98% male (12,805), 50.02% female (12,793), mean age 48±16 years. Age distribution: 22.9% (5,887) aged 14-35 years, 54.9% (14,077) aged 36-60 years, 22% (5,634) aged ≥61 years. Exclusion: Patients <14 years (consistent with WHO CADe recommendations for ≥15 years). TB prevalence by radiologist: 1,029/25,598 (4.02%). UNCLEAR - ENROLLMENT METHOD: Paper states patients 'visited van and completed registration' but does NOT specify whether enrollment consecutive, random, or convenience-based. Described as 'mass chest radiography' screening but exact enrollment method not explicitly stated. No STARD/CONSORT flow diagram provided. Cannot assess if enrolled population representative of target screening population. No explicit exclusion criteria beyond age. Number screened versus excluded not reported. |
| --- |

*Index test(s):*

| Genki v1 (DeepTek Medical Imaging Pvt. Ltd., Pune, India) - AI-powered Computer-Aided Detection (CADe) software for chest X-ray TB screening. Architecture: UNET-based convolutional neural network with Xception backbone and attention mechanism. Training: Trained on >1 million CXRs. CRITICAL: None of study participants' CXR scans used to train software (independent test population). Deployment: Connected to X-ray machines via DICOM protocol in four mobile diagnostic vans. Processing: Provided results within one minute. Output: Binary classification per scan - 'TB suggestive' or 'TB not suggestive'. Visual output: Generated heatmaps and outlines around suspicious regions of interest. Threshold: Performance assessed 'at default operating point of Genki' (manufacturer-specified). Hardware: Operated on laptop ('Genki Edge Device') for offline/portable use without reliable internet. Workflow: AI triaging occurred immediately after image acquisition, before radiologist review and sputum/swab collection. STRENGTH: Automated AI processed CXR images before any clinical interpretation or confirmatory testing - inherently blinded to reference. UNCLEAR - THRESHOLD PRE-SPECIFICATION: Study used 'default operating point' suggesting manufacturer-specified threshold, but paper does NOT explicitly state this was pre-specified before study began, validated on independent external data, or provide actual threshold value. No evidence of data-driven threshold optimization on study data. |
| --- |

*Reference standard and target condition:*

| CRITICAL METHODOLOGICAL FLAW - INADEQUATE REFERENCE STANDARD  This study used RADIOLOGIST INTERPRETATION as reference standard for evaluating AI performance, NOT true gold standard (sputum microscopy/culture/NAAT). Paper explicitly states: 'Hence, only AI annotations were considered to be true for evaluating AI performance' (p.15, appears to be typo - context indicates 'radiologist annotations' were reference). Performance metrics calculated by comparing Genki predictions against classifications of 'expert radiologist with 15 years experience in reading chest radiographs' who classified scans as 'TB suggestive' or 'TB not suggestive'. Confusion matrix (Table 2) shows comparisons between 'Reported by radiologist' and 'Predicted by Genki'. CONSEQUENCE: Reported sensitivity (98%), specificity (96.9%), accuracy (96.9%) reflect AI-RADIOLOGIST AGREEMENT, NOT true diagnostic accuracy for tuberculosis. Study measures CONCORDANCE between two CXR interpretation methods rather than actual diagnostic performance against confirmed TB cases. ADDITIONAL CRITICAL FLAW - REVIEW/INCORPORATION BIAS: Radiologist serving as reference had ACCESS TO AI OUTPUTS (heatmaps, predictions) when classifying images. Paper states radiologist 'reviewed the output generated by the AI software to identify if the AI-flagged cases were indeed true TB' (p.15). Could bias radiologist classifications toward agreement with AI, artificially inflating apparent concordance. Authors acknowledge limitation: 'performance of CADe determined against annotations made by human reader' and 'preferred reference standard involves biological confirmation using sputum smears or NAATs' (p.18). Sputum/NAAT used for patient management but NOT as study reference. Target condition: Tuberculosis (pulmonary TB detectable on chest X-ray). |
| --- |

# Phase 2: Draw a Flow Diagram for the Primary Study

| PATIENT FLOW DIAGRAM - POPULATION SCREENING STUDY  STUDY PERIOD: January - December 2022 (12 months) STUDY DESIGN: Real-world population-based TB screening SETTING: Four mobile diagnostic units (MDUs) LOCATION: Remote/low-resource areas of Chennai, India • High TB burden setting • Mobile vans bringing screening to underserved populations • Point-of-care deployment in field conditions  ↓ ═══════════════════════════════════════════════════════════════════════ UNCLEAR: ENROLLMENT METHODOLOGY NOT EXPLICITLY STATED ═══════════════════════════════════════════════════════════════════════  RECRUITMENT DESCRIPTION: • "Patients from remote areas visited the van" • "Completed registration process" • "Underwent chest radiography" • Described as "mass chest radiography" screening program  ⚠️ ENROLLMENT METHOD UNCLEAR: • NOT explicitly stated if consecutive enrollment • NOT explicitly stated if random selection • Could be convenience sampling • Cannot assess representativeness • No STARD/CONSORT flow diagram provided • Number screened vs enrolled NOT reported  POPULATION TYPE: • Both symptomatic AND asymptomatic individuals • Appropriate for screening study  ↓ ELIGIBLE: Patients from remote areas visiting mobile diagnostic units Age: ≥14 years (consistent with WHO CADe recommendations for ≥15 years) (Number approached/screened: NOT REPORTED)  ↓ ENROLLED & ANALYZED: 25,598 patients (No exclusions reported from enrolled population)  DEMOGRAPHICS: • Sex: 49.98% male (12,805), 50.02% female (12,793) • Age: Mean 48±16 years  - 14-35 years: 5,887 (22.9%)  - 36-60 years: 14,077 (54.9%)  - ≥61 years: 5,634 (22%)  ✓ LARGE SAMPLE SIZE (N=25,598) ✓ BALANCED SEX DISTRIBUTION ✓ BROAD AGE RANGE ✓ NO MISSING DATA - all enrolled analyzed ✓ COMPLETE DATA OVER 12 MONTHS  ⚠️ No flow diagram showing screening cascade ⚠️ Exclusion criteria not explicitly stated beyond age  ↓ ═══════════════════════════════════════════════════════════════════════ ALL 25,598 PATIENTS RECEIVED BOTH INDEX TEST AND REFERENCE STANDARD ═══════════════════════════════════════════════════════════════════════  ✓ NO DIFFERENTIAL VERIFICATION ✓ NO PARTIAL VERIFICATION ✓ NO MISSING DATA ✓ COMPLETE 12-MONTH DATASET  INDEX TEST: GENKI v1 AI-POWERED CADe SOFTWARE  Technology Specifications: • Developer: DeepTek Medical Imaging Pvt. Ltd., Pune, India • Architecture: UNET-based convolutional neural network  Xception backbone  Attention mechanism • Training: >1 million chest X-rays  ⚠️ CRITICAL: None from study participants (independent test set)  Deployment: • Four mobile diagnostic vans • Connected to X-ray machines via DICOM protocol • Laptop-based ("Genki Edge Device") • Offline/portable operation (no reliable internet required) • Field-deployable for remote areas  Workflow Timing (Figure 1): Step 1: Patient registration Step 2: CXR image acquisition Step 3: AI triaging (Genki processing) ← INDEX TEST Step 4: Sputum/swab collection Step 5: Radiologist review ← REFERENCE STANDARD Step 6: Treatment/referral decisions  Processing: • Real-time analysis • Results provided within ONE MINUTE • Immediate after image acquisition • Before radiologist review • Before sputum collection  Output Classification (Binary): • "TB suggestive" • "TB not suggestive"  Visual Output: • Heatmaps showing suspicious regions • Outlines around regions of interest • Support for radiologist review  Threshold: ⚠️ UNCLEAR PRE-SPECIFICATION • Evaluated "at default operating point of Genki" • Suggests manufacturer-specified threshold • BUT: NOT explicitly stated as pre-specified before study • NOT validated on independent external data (per paper) • Actual threshold value NOT provided • No evidence of data-driven optimization on study data  Blinding: ✓ YES • Automated AI system • Processed images immediately after acquisition • Before any clinical interpretation • Before confirmatory testing • Cannot have foreknowledge of reference result  ↓ ═══════════════════════════════════════════════════════════════════════ ⚠️⚠️⚠️ CRITICAL FLAW: INADEQUATE REFERENCE STANDARD ⚠️⚠️⚠️ ═══════════════════════════════════════════════════════════════════════  REFERENCE STANDARD USED IN STUDY: Expert radiologist interpretation of chest X-rays • Single radiologist with 15 years experience • Classified each scan as "TB suggestive" or "TB not suggestive" • Based on CXR appearance only  ⚠️⚠️⚠️ THIS IS NOT THE TRUE GOLD STANDARD FOR TB ⚠️⚠️⚠️  TRUE GOLD STANDARD FOR TB DIAGNOSIS (NOT USED AS REFERENCE): • Sputum smear microscopy • Sputum culture • Nucleic acid amplification tests (NAAT) • Microbiological confirmation  WHAT WAS ACTUALLY DONE: • Sputum/NAAT collected (Step 4 in workflow) • Used for PATIENT MANAGEMENT decisions • NOT used as study reference standard for AI evaluation • AI performance evaluated against radiologist only  FUNDAMENTAL METHODOLOGICAL FLAW: Study measures AI-RADIOLOGIST AGREEMENT NOT AI ability to detect microbiologically-confirmed TB  CONSEQUENCE FOR PERFORMANCE METRICS: • Reported sensitivity (98%) = AI agreement with radiologist • Reported specificity (96.9%) = AI agreement with radiologist • Reported accuracy (96.9%) = AI-radiologist concordance • THESE ARE NOT TRUE DIAGNOSTIC ACCURACY METRICS  ADDITIONAL CRITICAL FLAW - REVIEW/INCORPORATION BIAS: ⚠️ Radiologist had ACCESS to AI outputs during interpretation Paper states: "reviewed the output generated by AI software to identify if AI-flagged cases were indeed true TB"  BIAS CONSEQUENCE: • Radiologist saw AI heatmaps and predictions • Could influence radiologist classification • Bias toward agreement with AI • Artificially inflates apparent concordance • Circular reasoning: AI evaluated against reference that saw AI  AUTHORS ACKNOWLEDGE LIMITATION (p.18): "Performance of CADe determined against annotations made by human reader" "Preferred reference standard involves biological confirmation using sputum smears or NAATs" "In future, AI threshold may be tuned using NAAT results to attain more reliable sensitivity and specificity values"  ↓ DISEASE STATUS BY RADIOLOGIST INTERPRETATION (IMPERFECT REFERENCE):  TB SUGGESTIVE BY RADIOLOGIST: 1,029/25,598 (4.02%) • This is radiologist classification, NOT confirmed TB • Unknown how many had microbiological confirmation  TB NOT SUGGESTIVE BY RADIOLOGIST: 24,569/25,598 (95.98%) • This is radiologist classification, NOT confirmed TB-negative • Unknown how many were microbiologically tested  TB PREVALENCE BY RADIOLOGIST: 4.02% • Reflects radiologist interpretation only • May not reflect true TB prevalence  ↓ AI CLASSIFICATION BY GENKI:  TB SUGGESTIVE BY AI: 1,802/25,598 (7.04%) • Higher rate than radiologist (4.02%) • 773 more cases flagged by AI than radiologist  TB NOT SUGGESTIVE BY AI: 23,796/25,598 (92.96%)  DISAGREEMENT CASES: • AI positive, Radiologist negative: 773 cases (3.02%)  → Could be AI false positives OR radiologist false negatives  → Cannot determine without microbiological confirmation • AI negative, Radiologist positive: 21 cases (0.08%)  → Could be AI false negatives OR radiologist false positives  → Cannot determine without microbiological confirmation  ↓ ═══════════════════════════════════════════════════════════════════════ CONFUSION MATRIX (Table 2) - AI vs RADIOLOGIST AGREEMENT ═══════════════════════════════════════════════════════════════════════   RADIOLOGIST  TB+ TB- TOTAL AI TB+ 1,008 773 1,802  TB- 21 23,775 23,796  TOTAL 1,029 24,569 25,598  TRUE POSITIVES: 1,008 (AI+ and Radiologist+) FALSE POSITIVES: 773 (AI+ but Radiologist-) FALSE NEGATIVES: 21 (AI- but Radiologist+) TRUE NEGATIVES: 23,775 (AI- and Radiologist-)  ARITHMETIC CHECK: 1,008 + 773 + 21 + 23,775 = 25,598 ✓  AGREEMENT METRICS (NOT TRUE DIAGNOSTIC ACCURACY): • Sensitivity: 1,008/1,029 = 98.0% • Specificity: 23,775/24,569 = 96.9% • PPV: 1,008/1,802 = 55.9% • NPV: 23,775/23,796 = 99.9% • Overall Accuracy: (1,008+23,775)/25,598 = 96.9%  ⚠️ INTERPRETATION WARNING: These metrics reflect AI-RADIOLOGIST AGREEMENT NOT ability to detect microbiologically-confirmed TB Cannot interpret as true sensitivity/specificity for TB detection  ↓ SUBGROUP ANALYSES (Table 3) - AGREEMENT BY DEMOGRAPHICS:  SEX: • Male (n=12,805): Sensitivity 98.3%, Specificity 96.8% • Female (n=12,793): Sensitivity 97.8%, Specificity 97.0%  AGE: • 14-35 years (n=5,887): Sensitivity 95.1%, Specificity 94.2% • 36-60 years (n=14,077): Sensitivity 98.6%, Specificity 97.1% • ≥61 years (n=5,634): Sensitivity 98.7%, Specificity 97.3%  Consistent >95% sensitivity and >94% specificity across subgroups BUT: Still represents agreement with radiologist, not true accuracy  MONTHLY PERFORMANCE (Table 1) - TEMPORAL CONSISTENCY: All 12 months showed: • Sensitivity range: 95.3% - 100% • Specificity range: 95.7% - 98.5% • Accuracy range: 95.8% - 98.4%  Stable performance throughout study period BUT: Still measures agreement with radiologist interpretation  ═══════════════════════════════════════════════════════════════════════ SUMMARY OF CRITICAL METHODOLOGICAL ISSUES ═══════════════════════════════════════════════════════════════════════  1. INADEQUATE REFERENCE STANDARD (HIGH RISK - FATAL FLAW):  • Radiologist CXR interpretation used as reference  • NOT microbiological confirmation (true gold standard)  • Metrics reflect AI-radiologist agreement only  • Cannot assess true TB diagnostic accuracy  • Fundamental methodological flaw  2. REVIEW/INCORPORATION BIAS (HIGH RISK):  • Radiologist had access to AI outputs (heatmaps, predictions)  • Could bias classifications toward AI agreement  • Artificially inflates apparent concordance  • Circular evaluation  3. ENROLLMENT METHOD UNCLEAR:  • Not specified if consecutive, random, or convenience  • Cannot assess selection bias risk  • No flow diagram  • Representativeness uncertain  4. THRESHOLD PRE-SPECIFICATION UNCLEAR:  • "Default operating point" used  • But NOT explicitly stated as pre-specified before study  • Threshold value not provided  • External validation not documented  5. AUTHORS ACKNOWLEDGE LIMITATIONS:  • Explicitly state radiologist interpretation inadequate  • Recommend future validation against NAAT  • Recognize need for biological confirmation |
| --- |

# Phase 3: Risk of Bias and Applicability Judgments

# DOMAIN 1: PATIENT SELECTION

## A. Risk of Bias

**Describe methods of patient selection:**Real-world population screening in Chennai, India. Total 25,598 patients screened January-December 2022 using four mobile diagnostic units in remote/low-resource areas. Population: Mass chest radiography screening including symptomatic and asymptomatic individuals. Demographics: 49.98% male (12,805), 50.02% female (12,793), mean age 48±16 years. Age distribution: 22.9% (5,887) aged 14-35, 54.9% (14,077) aged 36-60, 22% (5,634) aged ≥61. Exclusion: Age <14 years consistent with WHO CADe recommendations. TB prevalence by radiologist: 4.02% (1,029/25,598). UNCLEAR - ENROLLMENT METHOD: Paper states patients 'visited van and completed registration' but does NOT specify whether enrollment consecutive, random, or convenience-based. Described as 'mass chest radiography' screening but exact method not explicit. No STARD/CONSORT flow diagram. No explicit exclusion criteria beyond age. Number screened versus enrolled NOT reported. Cannot assess if enrolled population representative of target screening population. Large sample (N=25,598) with balanced demographics is strength. Page reference: p.14-16

| Signaling Question | Answer |
| --- | --- |
| ❖ Was a consecutive or random sample enrolled? | UNCLEAR |
| ❖ Was a case-control design avoided? | YES |
| ❖ Did study avoid inappropriate exclusions? | UNCLEAR |

**Could the selection of patients have introduced bias?: RISK: UNCLEAR**

**Justification:** While this study employed a real-world population screening approach with a large sample (N=25,598) from mobile diagnostic units, the absence of explicit enrollment methodology creates uncertainty about selection bias. The paper does not specify whether patients presented consecutively, were randomly selected from those visiting the vans, or represented convenience sampling of available individuals. Additionally, no flow diagram or exclusion criteria were provided, making it impossible to assess whether the enrolled population is representative of the target screening population. The study includes both symptomatic and asymptomatic patients, which is appropriate for a screening study, but lack of methodological transparency prevents a 'Low Risk' judgment.

## B. Concerns regarding applicability

**Is there concern that the included patients do not match the review question?: CONCERN: LOW**

**Justification:** This study directly aligns with the review scope of AI-based clinical decision support in point-of-care imaging settings. The population consists of individuals from remote/low-resource areas of Chennai, India - a high TB burden setting - presenting to mobile diagnostic units for TB screening. This represents a true point-of-care, real-world screening scenario. The demographic distribution (mean age 48±16 years, balanced sex distribution, TB prevalence 4.02%) reflects the intended target population for AI-based TB screening in resource-limited settings. The setting is appropriate for the review question regarding POC imaging applications.

# DOMAIN 2: INDEX TEST(S)

## A. Risk of Bias

**Describe the index test:**Genki v1 (DeepTek Medical Imaging Pvt. Ltd., Pune, India) - AI-powered CADe software for CXR TB screening. Architecture: UNET-based CNN with Xception backbone and attention mechanism. Training: >1 million CXRs. CRITICAL: None of study participants' CXR used to train software (independent test population). Deployment: Connected to X-ray machines via DICOM in four mobile vans. Processing: Results within one minute. Output: Binary classification 'TB suggestive' or 'TB not suggestive'. Visual: Heatmaps and outlines around suspicious regions. Threshold: Performance assessed 'at default operating point of Genki' (manufacturer-specified). Hardware: Laptop ('Genki Edge Device') for offline/portable use. Workflow: AI triaging after image acquisition, before radiologist review and sputum collection. STRENGTH: Automated AI processed CXR before any clinical interpretation or confirmatory testing - inherently blinded to reference (Figure 1). None of study CXRs in training data reduces training-test contamination. UNCLEAR - THRESHOLD: Used 'default operating point' suggesting manufacturer-specified threshold, but paper does NOT explicitly state this pre-specified before study, validated on independent external data, or provide actual threshold value. No evidence of data-driven optimization on study data. Page reference: p.14-15, p.17-18

| Signaling Question | Answer |
| --- | --- |
| ❖ Interpreted without knowledge of reference? | YES |
| ❖ If threshold used, was it pre-specified? | UNCLEAR |

**Could the conduct of the index test have introduced bias?: RISK: UNCLEAR**

**Justification:** The index test was appropriately blinded to reference standard results, as Genki is an automated AI system that processed images before any clinical interpretation or confirmatory testing. The study explicitly states that none of the study participants' CXR scans were used to train the software, reducing concern for training-test contamination. However, while the study used the 'default operating point' suggesting a manufacturer-specified threshold, the paper does not explicitly document that this threshold was pre-specified before the study began, validated on independent external data, or provide the actual threshold value. This lack of transparency about threshold specification prevents a 'Low Risk' judgment, though there is no evidence of data-driven threshold optimization.

## B. Concerns regarding applicability

**Is there concern about the index test applicability?: CONCERN: LOW**

**Justification:** Genki is a commercially available CADe software designed specifically for TB screening using chest X-rays in resource-limited settings. The system was deployed in its intended clinical context - mobile diagnostic vans in remote areas without reliable internet access. The workflow represents real-world deployment conditions: the software operates on a laptop ('Genki Edge Device') for offline/portable use and provides results within one minute. This directly aligns with the review's focus on AI-based clinical decision support in point-of-care imaging. The index test implementation matches how such systems would be used in practice for TB screening.

# DOMAIN 3: REFERENCE STANDARD

## A. Risk of Bias

**Describe the reference standard:**CRITICAL METHODOLOGICAL FLAW - INADEQUATE REFERENCE STANDARD:
Study used RADIOLOGIST INTERPRETATION as reference for evaluating AI performance, NOT true gold standard (sputum microscopy/culture/NAAT). Paper states: 'Hence, only AI annotations were considered true for evaluating AI performance' (p.15, appears typo - context indicates 'radiologist annotations' were reference). Performance calculated by comparing Genki versus 'expert radiologist with 15 years experience' who classified scans 'TB suggestive' or 'TB not suggestive'. Confusion matrix (Table 2) shows 'Reported by radiologist' versus 'Predicted by Genki'. CONSEQUENCE: Reported sensitivity (98%), specificity (96.9%), accuracy (96.9%) reflect AI-RADIOLOGIST AGREEMENT, NOT true TB diagnostic accuracy. Study measures CONCORDANCE between two CXR interpretation methods, not actual diagnostic performance against confirmed TB. ADDITIONAL CRITICAL FLAW - REVIEW/INCORPORATION BIAS: Radiologist serving as reference had ACCESS TO AI OUTPUTS (heatmaps, predictions) when classifying. Paper: radiologist 'reviewed output generated by AI software to identify if AI-flagged cases were indeed true TB' (p.15). Could bias radiologist toward AI agreement, artificially inflating concordance. Circular evaluation. Authors acknowledge: 'performance determined against annotations by human reader' and 'preferred reference involves biological confirmation using sputum/NAATs' (p.18). Sputum/NAAT used for patient management but NOT study reference. Page reference: p.15, p.16, p.18

| Signaling Question | Answer |
| --- | --- |
| ❖ Is reference standard likely to correctly classify? | NO |
| ❖ Interpreted without knowledge of index test? | NO |

**Could the reference standard have introduced bias?: RISK: HIGH**

**Justification:** HIGH risk due to two critical flaws. First, INADEQUATE REFERENCE STANDARD: The study used radiologist CXR interpretation, not microbiological confirmation (sputum smear/culture/NAAT), as the reference standard for evaluating AI performance. This is fundamentally inappropriate because radiologist interpretation of chest X-rays is not the true gold standard for TB diagnosis. Consequently, the reported performance metrics (98% sensitivity, 96.9% specificity) reflect AI-radiologist agreement rather than ability to detect microbiologically-confirmed TB. Second, REVIEW/INCORPORATION BIAS: The radiologist serving as the reference standard had access to AI outputs (heatmaps and predictions) when making classifications. This creates circular reasoning where the AI is evaluated against a reference that was influenced by the AI itself, artificially inflating apparent concordance. Authors explicitly acknowledge these limitations.

## B. Concerns regarding applicability

**Is there concern about reference standard applicability?: CONCERN: LOW**

**Justification:** Despite being an imperfect reference standard for the study, radiologist interpretation of chest X-rays is a relevant and appropriate clinical comparator for AI systems in TB screening programs. In real-world practice, AI CADe systems are intended to assist or replace radiologist interpretation in resource-limited settings. The target condition (pulmonary TB detectable on chest X-ray) is clearly defined and clinically relevant. The reference standard, while inadequate for rigorous diagnostic accuracy assessment, reflects actual deployment conditions where microbiological confirmation may not be available for all screened individuals.

# DOMAIN 4: FLOW AND TIMING

## A. Risk of Bias

**Patient flow:**Excellent patient flow. All 25,598 enrolled patients received both index test (Genki AI) and reference standard (radiologist CXR interpretation). NO differential verification - all received same reference regardless of AI results. NO partial verification - all received complete reference. NO missing data - entire enrolled population included in analysis. Time interval appropriate - AI processing occurred immediately after image acquisition (within 1 minute) followed by radiologist review as part of same screening visit. Workflow shows AI at step 3 and radiologist at step 5 during same clinical encounter (Figure 1). Complete 12-month dataset with stable performance throughout study period (Table 1). No post-enrollment exclusions. This domain demonstrates excellent methodological quality for flow and timing. STRENGTH: Complete verification without verification bias, no missing data, appropriate temporal sequence. Page reference: p.15-16, Figure 1

| Signaling Question | Answer |
| --- | --- |
| ❖ Appropriate interval between tests? | YES |
| ❖ Did all patients receive reference standard? | YES |
| ❖ Did patients receive same reference standard? | YES |
| ❖ Were all patients included in analysis? | YES |

**Could the patient flow have introduced bias?: RISK: LOW**

**Justification:** LOW risk - this domain demonstrates excellent methodological quality. All 25,598 enrolled patients received both the index test (Genki AI) and reference standard (radiologist interpretation) with no differential or partial verification. There was no missing data, with 100% of enrolled patients included in the analysis. The time interval was appropriate, with AI processing occurring immediately after image acquisition (within 1 minute) followed by radiologist review during the same screening visit. Performance remained stable throughout the 12-month study period with no post-enrollment exclusions. This represents one of the study's major methodological strengths.

# OVERALL ASSESSMENT SUMMARY

**HIGH RISK OF BIAS - INADEQUATE REFERENCE STANDARD**

| Domain | Signaling Questions | Risk of Bias | Applicability |
| --- | --- | --- | --- |
| D1: Patient Selection | 1.1 Unclear 1.2 Yes 1.3 Unclear | UNCLEAR | LOW |
| D2: Index Test | 2.1 Yes 2.2 Unclear | UNCLEAR | LOW |
| D3: Reference Standard | 3.1 No 3.2 No | HIGH | LOW |
| D4: Flow and Timing | 4.1 Yes 4.2 Yes 4.3 Yes 4.4 Yes | LOW | N/A |

**Overall Risk of Bias: HIGH**

**Justification:** This study has HIGH overall risk of bias (1 HIGH + 2 UNCLEAR + 1 LOW) due to a fundamental methodological flaw in Domain 3 (Reference Standard). PRIMARY CONCERN: The study used radiologist CXR interpretation as the reference standard rather than microbiological confirmation (sputum smear/culture/NAAT). This means the reported performance metrics (98% sensitivity, 96.9% specificity, 96.9% accuracy) reflect AI-radiologist agreement, NOT true diagnostic accuracy for tuberculosis. The study measures concordance between two CXR interpretation methods rather than ability to detect microbiologically-confirmed TB. SECONDARY CONCERN: Review/incorporation bias - the radiologist had access to AI outputs (heatmaps, predictions) when classifying images, creating circular reasoning that could inflate apparent concordance. Domain 1 (Patient Selection): UNCLEAR - enrollment method not explicitly stated. Domain 2 (Index Test): UNCLEAR - threshold pre-specification not explicitly documented. Domain 4 (Flow & Timing): LOW - excellent with complete verification and no missing data. Authors explicitly acknowledge the reference standard limitation.

**Overall Applicability Concern: LOW**

**Justification:** Despite methodological limitations, this study has high applicability to the review question. The study evaluated a commercially available AI CADe system (Genki) in its intended deployment context - mobile diagnostic units screening for TB in remote, resource-limited areas of Chennai, India (high TB burden setting). The population from remote areas seeking TB screening, the point-of-care setting with portable offline equipment, the real-world operational conditions over 12 months, and the large sample size (N=25,598) all represent authentic conditions where such AI systems would be deployed. This directly aligns with the review's focus on AI-based clinical decision support in point-of-care imaging for resource-limited settings.

## Critical Biases and Limitations

**1. INADEQUATE REFERENCE STANDARD (SEVERITY: HIGH - FATAL FLAW):**Study used radiologist CXR interpretation as reference standard, NOT microbiological confirmation (sputum smear/culture/NAAT) which is the true gold standard for TB diagnosis. CONSEQUENCE: Reported sensitivity (98%), specificity (96.9%), and accuracy (96.9%) reflect AI-RADIOLOGIST AGREEMENT, not true diagnostic accuracy for detecting microbiologically-confirmed tuberculosis. Study fundamentally measures CONCORDANCE between two CXR interpretation methods rather than actual diagnostic performance against confirmed TB cases. This is a fundamental methodological flaw that prevents interpretation of results as true diagnostic accuracy. Authors explicitly acknowledge limitation.

**2. REVIEW BIAS / INCORPORATION BIAS (SEVERITY: HIGH):**Radiologist serving as reference standard had ACCESS TO AI OUTPUTS (heatmaps and predictions) when classifying images. Paper states radiologist "reviewed output generated by AI software to identify if AI-flagged cases were indeed true TB." Creates CIRCULAR REASONING where AI evaluated against reference that was influenced by AI itself. Could bias radiologist classifications toward agreement with AI, artificially inflating apparent concordance. Violates principle of independent reference standard.

**3. ENROLLMENT METHOD UNCLEAR (SEVERITY: UNCLEAR):**Paper does not specify whether patients were consecutively enrolled, randomly selected, or represented convenience sampling from those visiting mobile units. Described as "mass chest radiography" screening but exact enrollment methodology not explicit. No STARD/CONSORT flow diagram. Number screened versus enrolled not reported. Cannot assess selection bias risk or whether enrolled population representative of target screening population. Large sample (N=25,598) partially mitigates concern but transparency lacking.

**4. THRESHOLD PRE-SPECIFICATION UNCLEAR (SEVERITY: UNCLEAR):**Study evaluated performance "at default operating point of Genki" suggesting manufacturer-specified threshold (not data-derived optimization on study data). However, paper does NOT explicitly state threshold was pre-specified before study began, validated on independent external data, or provide actual threshold value. Lack of documentation creates uncertainty about potential optimistic bias from post-hoc threshold selection, though no evidence of optimization on study data.

## Significant Methodological Strengths

**Despite the fatal flaw in reference standard, this study has important strengths:

✓ Large sample size:** 25,598 patients provides substantial statistical power and narrow confidence intervals
**✓ Real-world deployment:** Evaluated AI in actual operational conditions (mobile vans in remote areas) over 12 months
**✓ Independent test population:** None of study participants' CXRs used to train Genki, reduces training-test contamination
**✓ Complete verification:** All 25,598 patients received both index test and reference (no differential/partial verification)
**✓ No missing data:** 100% of enrolled analyzed, complete 12-month dataset
**✓ Subgroup analyses:** Performance evaluated across sex and age strata, showing consistency (>95% sensitivity, >94% specificity)
**✓ Temporal consistency:** Monthly performance stable throughout 12 months (Table 1)
**✓ Appropriate timing:** AI processing within 1 minute, radiologist review same visit, no interval issues
**✓ Point-of-care deployment:** Offline laptop-based system for remote areas without internet
**✓ Authors transparent:** Explicitly acknowledge reference standard limitation and need for biological confirmation

## Reported Performance (AI-Radiologist Agreement)

**CRITICAL: These metrics reflect AI-RADIOLOGIST AGREEMENT, NOT true TB diagnostic accuracy

Population: 25,598 patients screened

Disease Status by Radiologist (Imperfect Reference):**• TB suggestive: 1,029/25,598 (4.02%)
• TB not suggestive: 24,569/25,598 (95.98%)

**AI Classification by Genki:**• TB suggestive: 1,802/25,598 (7.04%)
• TB not suggestive: 23,796/25,598 (92.96%)
• AI flagged 773 MORE cases than radiologist (3.02%)

**Confusion Matrix (AI vs Radiologist):**• True Positives: 1,008 (both AI+ and Rad+)
• False Positives: 773 (AI+ but Rad-)
• False Negatives: 21 (AI- but Rad+)
• True Negatives: 23,775 (both AI- and Rad-)

**Agreement Metrics (NOT True Diagnostic Accuracy):**• Sensitivity: 1,008/1,029 = 98.0%
• Specificity: 23,775/24,569 = 96.9%
• PPV: 1,008/1,802 = 55.9%
• NPV: 23,775/23,796 = 99.9%
• Overall Accuracy: 96.9%

**Subgroup Performance (Agreement by Demographics):**Sex:
• Male (n=12,805): Sens 98.3%, Spec 96.8%
• Female (n=12,793): Sens 97.8%, Spec 97.0%

Age:
• 14-35 years (n=5,887): Sens 95.1%, Spec 94.2%
• 36-60 years (n=14,077): Sens 98.6%, Spec 97.1%
• ≥61 years (n=5,634): Sens 98.7%, Spec 97.3%

**Monthly Performance (Temporal Consistency):**All 12 months:
• Sensitivity range: 95.3%-100%
• Specificity range: 95.7%-98.5%
• Accuracy range: 95.8%-98.4%

**INTERPRETATION WARNING:** These metrics measure AI-radiologist agreement, NOT ability to detect microbiologically-confirmed TB. Without biological confirmation as reference, cannot assess true sensitivity/specificity for TB diagnosis. Disagreement cases (773 AI+/Rad- and 21 AI-/Rad+) could represent either AI errors OR radiologist errors - cannot determine without gold standard.

## Study Value and Context

**What this study demonstrates:

✓ AI-radiologist concordance:** High agreement (96.9%) between Genki AI and expert radiologist in CXR TB screening
**✓ Real-world feasibility:** Demonstrated deployment in mobile vans, remote areas, 12 months operational
**✓ Large-scale validation:** 25,598 patients, substantial statistical power
**✓ Demographic consistency:** Performance stable across sex and age groups
**✓ Temporal stability:** Monthly performance consistent throughout year
**✓ Point-of-care deployment:** Offline portable system suitable for resource-limited settings

**What this study does NOT demonstrate:

✗ True TB diagnostic accuracy:** Cannot assess sensitivity/specificity for microbiologically-confirmed TB
**✗ Clinical decision validity:** Unknown if AI correctly identifies actual TB cases versus radiologist errors
**✗ Independent evaluation:** Reference standard contaminated by AI outputs (review bias)

**Authors' recommendations:**Authors explicitly acknowledge limitations and recommend:
"In future, AI threshold may be tuned using NAAT results to attain
more reliable sensitivity and specificity values"

**Value for systematic review:

• Supports:** Feasibility of AI CADe deployment in mobile screening, technical consistency
**• Does NOT support:** Claims about true sensitivity/specificity for TB detection
**• Requires:** Cautious interpretation as AI-radiologist agreement study, not diagnostic accuracy

QUADAS-2

**Kazemzadeh et al. 2024**Prospective Multi-Site Validation of AI to Detect Tuberculosis and Chest X-Ray Abnormalities
*Study ID: Kazemzadeh_2024 | Assessment Date: 2025-11-28*

____________________________________________________________________________________________________

# Phase 1: State the Review Question

*Patients (setting, intended use of index test, presentation, prior testing):*

| Adults aged ≥18 years enrolled prospectively from November 2021 to February 2023 at three health facilities in Lusaka district, Zambia (Chawama, Kanyama, Chainda) - all providing TB diagnostic and treatment services. Setting: Primary care-level health facilities in high TB/HIV burden region (TB incidence 307/100,000). Intended use: Cloud-based AI for TB screening and CXR abnormality triage to guide diagnostic workup. Presentation: Eligible patients met any of three conditions - (1) Presumptive TB patient with cardinal symptoms (cough, fever, weight loss, night sweats) regardless of HIV status, (2) Household contact with TB patient, or (3) Newly diagnosed HIV patient. Prior testing: None specified. Exclusions: Patients on current TB treatment or within 1 year of treatment (to avoid diagnostic confusion). Sample: 1978 screened, 1932 enrolled. Post-enrollment exclusions: 20 missing digital CXR (1.0%), 2 withdrew (0.1%), 83 excluded from TB AI analysis due to indeterminate reference standard (24 contaminated samples, 52 trace Xpert without culture positivity, 7 invalid Xpert). Final analysis: 1827 for TB AI (192 TB-positive = 10.5% prevalence), 1910 for abnormality AI (554 abnormalities = 29%). Population characteristics: 35.5% HIV-positive (649), 16.5% previous TB treatment (301). |
| --- |

*Index test(s):*

| Two cloud-based AI systems from Google: (1) TB-detecting AI (TB AI) and (2) Abnormality-detecting AI (abnormality AI). Both models accepted single frontal CXR DICOM image as input and produced score 0-1. Digital CXRs were de-identified and uploaded to Google Cloud DICOM store; both AI systems ran daily on uploaded images through Apache Beam pipelines. AI results written into new DICOM images within 24 hours of upload. AI results NOT available to study team during visit and did NOT influence patient care. Thresholds: PRE-SPECIFIED based on pilot phase of 493 patients preceding main study (pilot data excluded from statistical analysis). TB AI thresholds: (a) High-sensitivity 0.305 designed to favor sensitivity given WHO targets, (b) Balanced exploratory 0.465 designed to approximate radiologist performance. Abnormality AI thresholds: (a) High-sensitivity 0.54, (b) Balanced 0.67. Study preregistered on clinicaltrials.gov (NCT05139940). Automated inference with no human interpretation eliminated review bias. |
| --- |

*Reference standard and target condition:*

| Two reference standards: (1) TB status: Mycobacterium tuberculosis isolated on culture OR detected at very low to high levels through Xpert Ultra Mtb testing. Xpert Ultra trace results included ONLY if culture was positive. Sputum samples sent to CIDRZ central laboratory for Xpert Ultra testing, smear, and Mycobacterium culture using standard operating procedures. Samples processed using NALC-NaOH method; remaining pellet used for smear, inoculation onto solid Lowenstein-Jensen media and liquid MGIT media, and Xpert Ultra testing. Positive culture samples identified using Ziehl-Neelsen staining followed by BD MGIT TBc identification test for AFB-positive cultures to confirm M. tuberculosis presence. Extra sample also sent to local laboratory for Xpert Ultra testing. (2) CXR abnormality: Majority vote of 3 India-based radiologists with 8, 12, and 14 years experience reviewing CXRs for suspected TB (selected from pool of 19 based on availability and experience). Radiologists underwent onboarding, reviewed each image independently, provided binary yes/no for presence of potentially actionable findings. Target conditions: Active TB (microbiologically confirmed) and CXR abnormalities requiring clinical action. UNIFORM REFERENCE STANDARD: All patients received same reference standard protocol - NO differential verification bias. |
| --- |

# Phase 2: Draw a Flow Diagram for the Primary Study

| PATIENT FLOW DIAGRAM - PROSPECTIVE VALIDATION STUDY  STUDY PERIOD: November 2021 - February 2023 (15 months) SETTING: Three health facilities, Lusaka district, Zambia (Chawama, Kanyama, Chainda - all provide TB diagnostic/treatment services)  ↓ SCREENED FOR ELIGIBILITY (n = 1,978)  ↓ EXCLUSIONS: 46 (2.3%) • Patients on current TB treatment or within 1 year of treatment  ↓ ENROLLED (n = 1,932)  ELIGIBILITY CRITERIA (any of three): 1. Presumptive TB patient with cardinal symptoms (cough, fever, weight loss,   night sweats), regardless of HIV status 2. Household contact with TB patient 3. Newly diagnosed HIV patient  POPULATION CHARACTERISTICS: • Adults ≥18 years • 35.5% HIV-positive (649) • 16.5% previous TB treatment (301) • High TB/HIV burden setting (TB incidence 307/100,000)  ↓ ═══════════════════════════════════════════════════════════════════════ ALL PATIENTS RECEIVED BOTH INDEX TEST & REFERENCE STANDARD ═══════════════════════════════════════════════════════════════════════  INDEX TEST: Cloud-based AI Systems (Google) Digital CXR performed at enrollment De-identified and uploaded to Google Cloud DICOM store  ↓ TWO AI SYSTEMS (automated inference via Apache Beam pipelines): 1. TB-detecting AI (TB AI)  • Input: Single frontal CXR DICOM image  • Output: Score 0-1  • Thresholds PRE-SPECIFIED from separate pilot cohort (n=493):  - High-sensitivity: 0.305  - Balanced: 0.465   2. Abnormality-detecting AI (abnormality AI)  • Input: Single frontal CXR DICOM image  • Output: Score 0-1  • Thresholds PRE-SPECIFIED from pilot:  - High-sensitivity: 0.54  - Balanced: 0.67  Results: Written into new DICOM images within 24 hours CRITICAL: AI results NOT available to study team during visit  AI did NOT influence patient care (blinding preserved)  ↓ REFERENCE STANDARD (same protocol for ALL patients):  FOR TB STATUS: Sputum samples → CIDRZ central laboratory ├─→ Xpert Ultra testing ├─→ Smear microscopy └─→ M. tuberculosis culture (Lowenstein-Jensen + MGIT media)  DEFINITION OF TB-POSITIVE: • M. tuberculosis on culture OR • Xpert Ultra detection (very low to high levels) • Xpert Ultra "trace" ONLY if culture positive  FOR CXR ABNORMALITY: Majority vote of 3 experienced India-based radiologists • 8, 12, and 14 years experience (from pool of 19) • Independent review with onboarding • Binary yes/no for potentially actionable findings  ↓ POST-ENROLLMENT EXCLUSIONS (n = 105, 5.4%): ├─→ Missing digital CXR: 20 (1.0%) ├─→ Withdrew: 2 (0.1%) └─→ Indeterminate reference standard for TB: 83 (4.3%)  • Contaminated samples: 24  • Trace Xpert without culture positivity: 52  • Invalid Xpert results: 7  ↓ ═══════════════════════════════════════════════════════════════════════ FINAL ANALYSIS - COMPLETE VERIFICATION ═══════════════════════════════════════════════════════════════════════  TB AI ANALYSIS (n = 1,827): • TB-positive: 192 (10.5% prevalence) • TB-negative: 1,635 (89.5%) • ALL received microbiological reference standard  ABNORMALITY AI ANALYSIS (n = 1,910): • Abnormality present: 554 (29.0%) • No abnormality: 1,356 (71.0%) • ALL received radiologist majority vote reference  KEY RESULTS (TB AI at high-sensitivity threshold 0.305): • Sensitivity: 87% (95% CI: 82-92%) • Specificity: 70% (95% CI: 68-72%) • AUC: 0.87 (95% CI: 0.84-0.90) • WHO targets: 90% sensitivity, 70% specificity  → Met specificity target, narrowly missed sensitivity  RESULTS (Abnormality AI at high-sensitivity threshold 0.54): • Sensitivity: 97% (95% CI: 95-98%) • Specificity: 79% (95% CI: 77-81%) • AUC: 0.97 (95% CI: 0.96-0.98) • Targets: 90% sensitivity, 50% specificity → Exceeded both  COMPARISON TO RADIOLOGISTS: • Mean radiologist sensitivity for TB: 76% • Mean radiologist specificity for TB: 82% • AI performed comparably to experienced radiologists  METHODOLOGICAL STRENGTHS: 1. Prospective design with preregistration (NCT05139940) 2. Pre-specified thresholds from separate pilot cohort 3. Uniform reference standard - NO differential verification 4. Automated AI inference - NO interpretation bias 5. Complete patient flow documentation (STARD-compliant) 6. Multi-site validation (3 facilities) 7. High-burden real-world setting 8. Subgroup analyses (HIV status, prior TB, age, sex)  LIMITATIONS: 1. Enrollment method not explicitly stated as consecutive 2. 5.4% exclusion rate (technical issues, indeterminate results) 3. Grid-like artifacts from one CXR machine at two sites 4. India-based radiologists (may not reflect local expertise) 5. Neither AI nor radiologists met WHO 90% sensitivity target |
| --- |

# Phase 3: Risk of Bias and Applicability Judgments

# DOMAIN 1: PATIENT SELECTION

## A. Risk of Bias

**Describe methods of patient selection:**This prospective study enrolled adults aged ≥18 years between November 2021 and February 2023 at three health facilities in Lusaka district of Zambia (Chawama, Kanyama, Chainda), all providing TB diagnostic and treatment services. Total 1978 adults screened, 1932 enrolled. Eligible patients met any of three conditions: (1) presumptive TB patient with cardinal symptoms (cough, fever, weight loss, night sweats) regardless of HIV status, (2) household contact with TB patient, or (3) newly diagnosed HIV patient. Exclusions: Patients on current TB treatment or within 1 year of treatment. Post-enrollment: 20 missing CXR (1.0%), 2 withdrew (0.1%), 83 excluded from TB AI analysis due to indeterminate reference standard (24 contaminated, 52 trace Xpert without culture, 7 invalid Xpert). Page reference: p.3, p.5, p.12

| Signaling Question | Answer |
| --- | --- |
| ❖ Was a consecutive or random sample enrolled? | UNCLEAR |
| ❖ Was a case-control design avoided? | YES |
| ❖ Did study avoid inappropriate exclusions? | YES |

**Could the selection of patients have introduced bias?: RISK: LOW**

**Justification:** This prospective, multi-site study enrolled 1932 participants from three health facilities in Zambia using clearly defined eligibility criteria based on clinical presentation. The cohort design avoided artificial inflation of diagnostic accuracy that would occur with case-control sampling. Exclusion criteria were clinically appropriate (recent TB treatment) and post-enrollment exclusions (105/1932 = 5.4%) were well-documented with clear reasons in a STARD flow diagram. While the enrollment method was not explicitly described as consecutive, the prospective design with specific eligibility windows and clinical triggers suggests systematic enrollment rather than convenience sampling.

## B. Concerns regarding applicability

**Is there concern that the included patients do not match the review question?: CONCERN: LOW**

**Justification:** This study population is highly applicable to AI-based clinical decision support in point-of-care settings. The study was conducted at primary care-level health facilities providing TB diagnostic services in a high TB/HIV burden setting (Zambia, TB incidence 307/100,000). The population included 35.5% HIV-positive patients (649) and 16.5% with previous TB treatment (301), representing the real-world spectrum of patients presenting for TB screening in resource-constrained settings. The use of digital CXR interpreted by cloud-based AI aligns well with the POC imaging concept for screening and triage.

# DOMAIN 2: INDEX TEST(S)

## A. Risk of Bias

**Describe the index test:**Two cloud-based AI systems (Google): TB-detecting AI and abnormality-detecting AI. Both accepted single frontal CXR DICOM image as input, produced score 0-1. Digital CXRs de-identified and uploaded to Google Cloud DICOM store; AI systems ran daily via Apache Beam pipelines. Thresholds PRE-SPECIFIED based on pilot phase of 493 patients preceding main study (pilot data excluded from analysis). TB AI thresholds: 0.305 (high-sensitivity), 0.465 (balanced). Abnormality AI thresholds: 0.54 (high-sensitivity), 0.67 (balanced). AI results NOT available to study team during visit and did NOT influence patient care. Study preregistered (NCT05139940). Page reference: p.3, p.5

| Signaling Question | Answer |
| --- | --- |
| ❖ Interpreted without knowledge of reference? | YES |
| ❖ If threshold used, was it pre-specified? | YES |

**Could the conduct of the index test have introduced bias?: RISK: LOW**

**Justification:** The AI systems operated autonomously via cloud-based pipelines with no human interpretation, eliminating the possibility of review bias. Thresholds were pre-specified using a separate pilot cohort of 493 patients that was excluded from the main analysis, avoiding optimistic performance estimates from threshold optimization on test data. The study was prospectively registered on clinicaltrials.gov (NCT05139940), providing external verification of pre-specification. Both AI models and their thresholds were described in sufficient detail for reproducibility, including exact threshold values.

## B. Concerns regarding applicability

**Is there concern about the index test applicability?: CONCERN: LOW**

**Justification:** The AI systems were used as intended for TB screening triage in a resource-constrained setting with limited radiologist access. The cloud-based deployment model with automated inference within 24 hours is directly applicable to POC settings where immediate radiologist interpretation is unavailable. The primary comparison was against WHO target thresholds for TB screening (90% sensitivity, 70% specificity), which are the established benchmarks for CAD software in TB screening programs. The abnormality AI targets (90% sensitivity, 50% specificity) were selected based on published literature for triage applications.

# DOMAIN 3: REFERENCE STANDARD

## A. Risk of Bias

**Describe the reference standard:**Two reference standards: (1) TB status: M. tuberculosis isolated on culture OR detected at very low to high levels through Xpert Ultra Mtb testing; Xpert Ultra trace results included ONLY if culture was positive. Sputum samples sent to CIDRZ central laboratory for Xpert Ultra, smear, and culture using standard procedures. Samples processed using NALC-NaOH method; remaining pellet for smear, inoculation onto Lowenstein-Jensen and MGIT media, and Xpert Ultra testing. Positive cultures identified using Ziehl-Neelsen staining followed by BD MGIT TBc identification test to confirm M. tuberculosis. (2) CXR abnormality: Majority vote of 3 India-based radiologists with 8, 12, and 14 years experience reviewing TB CXRs (selected from pool of 19). Radiologists underwent onboarding, reviewed each image independently, provided binary yes/no for potentially actionable findings. Page reference: p.3-4

| Signaling Question | Answer |
| --- | --- |
| ❖ Is reference standard likely to correctly classify? | YES |
| ❖ Interpreted without knowledge of index test? | YES |

**Could the reference standard have introduced bias?: RISK: LOW**

**Justification:** For TB, the reference standard used microbiological confirmation: M. tuberculosis culture positivity OR Xpert Ultra detection (very low to high levels), with trace results requiring culture confirmation. Culture and Xpert Ultra are WHO-recommended diagnostic tests for TB and represent the accepted gold standard. For abnormality, the reference was majority vote of 3 experienced radiologists (8-14 years experience), which is a reasonable approach for subjective CXR findings. The microbiological testing was performed at a separate central laboratory without knowledge of AI results. Radiologists reviewed de-identified images independently. No differential verification bias was present - all patients received the same reference standard protocol.

## B. Concerns regarding applicability

**Is there concern about reference standard applicability?: CONCERN: LOW**

**Justification:** The reference standards are appropriate and clinically relevant. Microbiological confirmation (culture/Xpert Ultra) is the WHO-recommended gold standard for TB diagnosis. The radiologist majority vote for abnormality provides an appropriate clinical benchmark for CXR interpretation. The target conditions (active TB requiring treatment, actionable CXR abnormalities) align with the intended use case for AI-assisted TB screening and triage in primary care settings.

# DOMAIN 4: FLOW AND TIMING

## A. Risk of Bias

**Patient flow:**All patients received both index test (CXR with AI analysis) and reference standard (microbiological testing and/or radiologist review) during the same visit. CXRs were performed at enrollment, and sputum samples were collected at the same visit for microbiological testing. Post-enrollment exclusions were well-documented: 20 missing CXR (1.0%), 2 withdrew (0.1%), 83 indeterminate reference for TB (24 contaminated, 52 trace Xpert without culture, 7 invalid Xpert). All exclusions had clear technical/procedural reasons. STARD flow diagram provided complete patient accounting. NO differential verification - all patients received same reference standard protocol. Page reference: p.5, p.12

| Signaling Question | Answer |
| --- | --- |
| ❖ Appropriate interval between tests? | YES |
| ❖ Did all patients receive reference standard? | YES |
| ❖ Did patients receive same reference standard? | YES |
| ❖ Were all patients included in analysis? | YES |

**Could the patient flow have introduced bias?: RISK: LOW**

**Justification:** All enrolled patients received both the index test and reference standard during the same clinical visit, eliminating interval bias. There was NO differential verification - all 1827 patients in the TB AI analysis received the identical microbiological reference standard (culture and Xpert Ultra), and all 1910 in the abnormality AI analysis received radiologist majority vote. Post-enrollment exclusions (105/1932 = 5.4%) were clearly documented with specific reasons (missing CXR, withdrawal, contaminated/invalid samples) in a STARD-compliant flow diagram. The uniform application of reference standards to all patients is a major methodological strength that prevents verification bias.

# OVERALL ASSESSMENT SUMMARY

**✓ GOLD STANDARD STUDY - EXCELLENT METHODOLOGICAL QUALITY ✓**

| Domain | Signaling Questions | Risk of Bias | Applicability |
| --- | --- | --- | --- |
| D1: Patient Selection | 1.1 Unclear 1.2 Yes 1.3 Yes | LOW | LOW |
| D2: Index Test | 2.1 Yes 2.2 Yes | LOW | LOW |
| D3: Reference Standard | 3.1 Yes 3.2 Yes | LOW | LOW |
| D4: Flow and Timing | 4.1 Yes 4.2 Yes 4.3 Yes 4.4 Yes | LOW | N/A |

**Overall Risk of Bias: LOW**

**Justification:** This is a methodologically exemplary diagnostic accuracy study with LOW risk of bias across all four QUADAS-2 domains. The study employed prospective enrollment with clearly defined eligibility criteria, avoided case-control design, used pre-specified AI thresholds from a separate pilot cohort, applied uniform microbiological and radiological reference standards to all patients without differential verification, and provided complete STARD-compliant documentation of patient flow. The study was preregistered (NCT05139940), further supporting pre-specification of methods. The only minor uncertainty is the enrollment method not being explicitly labeled as consecutive, but the prospective multi-site design with clinical triggers suggests systematic rather than convenience sampling. This study represents a gold standard for AI diagnostic accuracy evaluation in point-of-care settings.

**Overall Applicability Concern: LOW**

**Justification:** This study is highly applicable to the review question of AI-based clinical decision support for point-of-care imaging. The population (adults with TB symptoms, TB contacts, or new HIV diagnosis in a high-burden setting), intervention (cloud-based CXR AI for TB detection and abnormality triage), and clinical context (primary care facilities in resource-constrained Zambia) align well with the target use case for AI-assisted TB screening. The comparison against WHO performance targets (90% sensitivity, 70% specificity) and experienced radiologists provides clinically meaningful benchmarks. The cloud-based deployment with 24-hour turnaround represents a realistic POC implementation model.

## Major Methodological Strengths

**1. Prospective design with preregistration:** Registered on clinicaltrials.gov (NCT05139940) prior to enrollment
**2. Pre-specified thresholds:** Derived from separate pilot cohort (n=493) excluded from main analysis - prevents optimization bias
**3. NO differential verification:** All 1827 TB AI patients received identical microbiological reference (culture + Xpert Ultra); all 1910 abnormality AI patients received radiologist majority vote
**4. Appropriate blinding:** AI ran automated without knowledge of reference; microbiological testing performed at separate laboratory; radiologists reviewed de-identified images
**5. STARD-compliant reporting:** Complete flow diagram with all exclusions documented
**6. Multi-site validation:** Three health facilities in Zambia
**7. Real-world high-burden setting:** TB incidence 307/100,000; 35.5% HIV-positive; 16.5% prior TB
**8. Comprehensive subgroup analyses:** Stratified by HIV status, prior TB, age, sex

## Study Limitations

**1. Enrollment method unclear:** Not explicitly stated as consecutive (minor limitation given prospective design)
**2. 5.4% exclusion rate:** Post-enrollment technical issues (contaminated samples, invalid Xpert, missing CXR)
**3. Grid artifacts:** Grid-like artifacts from one CXR machine affected two sites - may have impacted AI performance
**4. India-based radiologists:** May not fully reflect local Zambian radiologist expertise
**5. Performance below WHO target:** Neither AI nor radiologists met WHO 90% sensitivity target (AI achieved 87% at high-sensitivity threshold)

## Reported Performance (Highly Reliable)

**TB AI (n=1,827; 192 TB-positive = 10.5% prevalence):**• AUC: 0.87 (95% CI: 0.84-0.90)
• High-sensitivity threshold (0.305): Sens 87%, Spec 70%
• Balanced threshold (0.465): Sens 78%, Spec 82%
• WHO targets: 90% sensitivity, 70% specificity
 → Met specificity, narrowly missed sensitivity (87% vs 90%)

**Abnormality AI (n=1,910; 554 abnormalities = 29.0%):**• AUC: 0.97 (95% CI: 0.96-0.98)
• High-sensitivity threshold (0.54): Sens 97%, Spec 79%
• Balanced threshold (0.67): Sens 92%, Spec 91%
• Targets: 90% sensitivity, 50% specificity → Exceeded both

**Comparison to Radiologists:**• Mean radiologist sensitivity for TB: 76%
• Mean radiologist specificity for TB: 82%
• AI performed comparably to experienced radiologists

**CONFIDENCE IN RESULTS:** Very high - uniform reference standard, pre-specified thresholds, no verification bias, large sample with narrow confidence intervals, multi-site validation, STARD-compliant reporting. Performance estimates are reliable and generalizable to similar high-burden TB screening settings.

QUADAS-2

**Love et al. 2018**Palpable Breast Lump Triage by Minimally Trained Operators in Mexico Using Computer-Assisted Diagnosis and Low-Cost Ultrasound *Study ID: Love_2018 | Assessment Date: 2025-11-28*

____________________________________________________________________________________________________

# Phase 1: State the Review Question

*Patients (setting, intended use of index test, presentation, prior testing):*

| 32 women with 32 palpable breast masses presenting to a government hospital in Jalisco, Mexico (Instituto de Seguridad y Servicios Sociales de los Trabajadores del Estado Hospital Regional Valentin Gomez Farias) in March 2016. Setting: Point-of-care - government hospital in LMIC with 9-month wait time for diagnostic ultrasound. Intended use: AI-based triage of palpable breast lumps using low-cost portable ultrasound operated by minimally trained healthcare workers to determine need for biopsy. Presentation: Women with at least one self- or physician-identified palpable breast mass, age ≥18 years. Prior testing: None specified. Exclusions: History of breast surgery at lesion site (breast implant, reduction, cancer surgery) that would interfere with field of view; existing mastitis or other signs of breast inflammation. Population: Median age 46 years (range 18-67). Disease prevalence: 2 cancers (6.3% malignancy rate). BI-RADS distribution: 2=13, 3=5, 4a=11, 4b=1, 5=1, 6=1. NOTE: Pilot study - enrollment method (consecutive vs convenience) NOT explicitly stated. Number screened not reported. |
| --- |

*Index test(s):*

| Triage-CADx (computer-assisted diagnosis) system developed by AI Strategy (Warren, NJ) using deep-learning convolutional neural network based on Inception-v3 architecture with TensorFlow. System designed to classify breast lesions as suspicious (biopsy recommended, displayed in red) or benign (no further action, displayed in green), producing score 0-1 where higher scores indicate greater suspicion. Operators: Three minimally trained healthcare workers (first-year medical student, surgical nurse, gynecologic intern) with NO prior ultrasound experience, after approximately 30 minutes of training. Equipment: Low-cost portable ultrasound device (GE Vscan Dual Probe, 8-MHz linear array transducer). Protocol: Operators acquired orthogonal images of palpable breast masses. CADx model was trained/validated on external data (ACRIN 6666, Magee-Womens Hospital, USC/UCLA phase I images) and tested on Mexico images - represents external validation on new population with different equipment. System evaluated each image from orthogonal pair separately and used maximum score from all views for final classification. CRITICAL LIMITATION: Threshold for binary classification (suspicious vs benign) NOT explicitly reported or stated to be pre-specified. Paper does not clarify whether red/green classification threshold was established during training or optimized on test data. |
| --- |

*Reference standard and target condition:*

| CRITICAL LIMITATION - DIFFERENTIAL VERIFICATION BIAS: Reference standard was NOT uniform across all patients. For patients with masses assessed by radiologist as BI-RADS ≥4a (N=14, 44%), reference standard was histopathology obtained via US-guided core biopsy per hospital standard of care. For patients with masses assessed as BI-RADS 2 (N=13) or BI-RADS 3 (N=5), totaling 18/32 patients (56%), there was NO histopathologic confirmation - these lesions classified as benign based SOLELY on radiologist BI-RADS assessment. SEVERE CIRCULARITY: Decision of which reference standard to apply was determined by initial radiologist assessment. Hospital radiologist (A.P.G.M.) had 3 years experience, performed targeted US using in-house Philips HD11XE machine, provided BI-RADS assessments. Breakdown: BI-RADS 6 (1 patient) = histopathology (invasive ductal carcinoma); BI-RADS 5 (1 patient) = histopathology (invasive ductal carcinoma); BI-RADS 4b (1 patient) = histopathology (benign); BI-RADS 4a (11 patients) = histopathology (all benign: 7 fibroadenomas, 2 simple cysts, 1 fibrocystic disease, 1 intraductal papilloma); BI-RADS 3 (5 patients) = NO histopathology, radiologist assessment only; BI-RADS 2 (13 patients) = NO histopathology, radiologist assessment only. Target condition: Malignant breast lesions requiring biopsy. FUNDAMENTAL FLAW: False negatives among unbiopsied BI-RADS 2-3 patients (18/32 = 56%) cannot be detected. BI-RADS 3 lesions have up to 2% malignancy risk per ACR guidelines. |
| --- |

# Phase 2: Draw a Flow Diagram for the Primary Study

| PATIENT FLOW DIAGRAM - PILOT STUDY  STUDY PERIOD: March 2016 (single month) SETTING: Government hospital, Jalisco, Mexico (Instituto de Seguridad y Servicios Sociales de los Trabajadores del Estado) Clinical context: 9-month wait time for diagnostic ultrasound  ↓ SCREENED FOR ELIGIBILITY: Number NOT reported  ↓ EXCLUSIONS: Number NOT reported • History of breast surgery at lesion site (implant, reduction, cancer surgery) • Mastitis or other signs of breast inflammation  ↓ ENROLLED: 32 women with 32 palpable breast masses  ELIGIBILITY: • At least one self- or physician-identified palpable breast mass • Age ≥18 years • NOTE: Enrollment method (consecutive vs convenience) NOT stated  POPULATION CHARACTERISTICS: • Median age: 46 years (range 18-67) • Disease prevalence: 2 cancers (6.3%) • BI-RADS distribution by radiologist:  - BI-RADS 2: 13 (40.6%)  - BI-RADS 3: 5 (15.6%)  - BI-RADS 4a: 11 (34.4%)  - BI-RADS 4b: 1 (3.1%)  - BI-RADS 5: 1 (3.1%)  - BI-RADS 6: 1 (3.1%)  ↓ ═══════════════════════════════════════════════════════════════════════ ALL PATIENTS RECEIVED INDEX TEST ═══════════════════════════════════════════════════════════════════════  INDEX TEST: AI-Based Portable Ultrasound (CADx System)  Equipment: GE Vscan Dual Probe (8-MHz linear array) Operators: 3 minimally trained healthcare workers: ├─→ First-year medical student ├─→ Surgical nurse └─→ Gynecologic intern Training: ~30 minutes (NO prior ultrasound experience)  Protocol: 1. Operators acquired orthogonal images of palpable masses 2. CADx system (AI Strategy, Inception-v3 CNN) processed images 3. Binary output: Suspicious (red) vs Benign (green) 4. Score 0-1, higher = more suspicious 5. Maximum score from all views used for final classification  AI Model Development: • Trained on external data (ACRIN 6666, Magee-Womens, USC/UCLA phase I) • Tested on Mexico images (external validation)  THRESHOLD ISSUE: Binary classification threshold NOT explicitly reported  or stated to be pre-specified  ↓ ═══════════════════════════════════════════════════════════════════════ DIFFERENTIAL VERIFICATION BIAS (SEVERE) ═══════════════════════════════════════════════════════════════════════  REFERENCE STANDARD PATHWAY 1: Histopathology (n=14, 44%) Applied to: BI-RADS ≥4a masses Method: US-guided core biopsy (hospital standard of care) Radiologist: A.P.G.M. (3 years experience, Philips HD11XE)  ↓ HISTOPATHOLOGY RESULTS (n=14): ├─→ BI-RADS 6 (1): Invasive ductal carcinoma ├─→ BI-RADS 5 (1): Invasive ductal carcinoma ├─→ BI-RADS 4b (1): Benign └─→ BI-RADS 4a (11): Benign  • 7 fibroadenomas  • 2 simple cysts  • 1 fibrocystic disease  • 1 intraductal papilloma  TOTAL: 2 malignant, 12 benign  REFERENCE STANDARD PATHWAY 2: Radiologist Assessment Only (n=18, 56%) Applied to: BI-RADS 2-3 masses Method: NO histopathology - assumed benign based on BI-RADS alone  ↓ RADIOLOGIST ASSESSMENT ONLY (n=18): ├─→ BI-RADS 3 (5): Assumed benign (NO biopsy) │ • ACR guidelines: Up to 2% malignancy risk │ • FALSE NEGATIVES CANNOT BE DETECTED │ └─→ BI-RADS 2 (13): Assumed benign (NO biopsy)  • FALSE NEGATIVES CANNOT BE DETECTED  ═══════════════════════════════════════════════════════════════════════ CIRCULARITY PROBLEM ═══════════════════════════════════════════════════════════════════════  Radiologist assessment both: 1. DETERMINES which reference standard to use (biopsy vs no biopsy) 2. SERVES AS the reference standard for 56% of patients  If radiologist missed a cancer in BI-RADS 2-3 group: → No biopsy performed → Cancer counted as "true negative" → Artificially inflates specificity  If CADx missed same cancer: → No biopsy to detect the miss → False negative undetectable → Artificially inflates sensitivity  ═══════════════════════════════════════════════════════════════════════ REPORTED PERFORMANCE (UNRELIABLE DUE TO VERIFICATION BIAS) ═══════════════════════════════════════════════════════════════════════  CADx System Performance: • Sensitivity: 100% (2/2 cancers detected) • Specificity: 100% (30/30 benign correctly classified) • AUC: 1.0 (perfect discrimination)  CRITICAL INTERPRETATION ISSUES: 1. Only 2 cancers total (VERY small sample) 2. 56% of patients had NO gold standard verification 3. Perfect accuracy likely ARTIFACTUALLY INFLATED 4. Confidence intervals would be EXTREMELY WIDE 5. Authors acknowledge: "In practice and with a larger number of   patient cases, we expect these numbers to change"  VERIFICATION SUMMARY: • Gold standard (histopathology): 14/32 (44%) • No gold standard: 18/32 (56%) • Decision criterion: Radiologist BI-RADS (creates circularity)  STUDY VALUE: Despite severe verification bias preventing valid diagnostic accuracy  assessment, study demonstrates: • Feasibility of minimally trained operators using portable ultrasound • AI system can process images from low-cost POC devices • Workflow applicable to resource-limited settings • Proof-of-concept for POC breast mass triage |
| --- |

# Phase 3: Risk of Bias and Applicability Judgments

# DOMAIN 1: PATIENT SELECTION

## A. Risk of Bias

**Describe methods of patient selection:**This prospective pilot study enrolled 32 women with 32 palpable breast masses presenting to a government hospital in Jalisco, Mexico in March 2016. Women were recruited if they had at least one self- or physician-identified palpable breast mass and were age ≥18 years. Exclusion criteria: history of breast surgery at lesion site (breast implant, reduction, cancer surgery) that would interfere with field of view, and existing mastitis or other signs of breast inflammation. Paper describes this as pilot study but does NOT explicitly state whether enrollment was consecutive or convenience-based during March 2016. Exact number screened or excluded not reported; only final 32 participants described. Page reference: p.2, p.4

| Signaling Question | Answer |
| --- | --- |
| ❖ Was a consecutive or random sample enrolled? | UNCLEAR |
| ❖ Was a case-control design avoided? | YES |
| ❖ Did study avoid inappropriate exclusions? | YES |

**Could the selection of patients have introduced bias?: RISK: UNCLEAR**

**Justification:** While the study appropriately avoided case-control design and used clinically reasonable exclusion criteria, the enrollment method is not explicitly stated. The paper does not confirm consecutive enrollment - it states patients were recruited who were presenting to the hospital during March 2016, but whether this was consecutive, random, or convenience sampling is unclear. Selection bias cannot be ruled out because it is unknown whether all eligible patients were enrolled or if certain patients were preferentially selected. This is a common limitation in pilot studies but introduces potential selection bias.

## B. Concerns regarding applicability

**Is there concern that the included patients do not match the review question?: CONCERN: LOW**

**Justification:** The study population directly matches the intended point-of-care use case: women with palpable breast masses presenting to a resource-limited government hospital in a low- to middle-income country setting (Mexico). The clinical scenario - triaging palpable breast lumps using minimally trained operators with low-cost portable ultrasound - is highly relevant to point-of-care clinical decision support. Patient demographics (median age 46 years, range 18-67) and the spectrum of lesions (6% malignancy rate, mix of BI-RADS 2-6 lesions) reflect real-world presentations in LMIC settings. The 9-month wait time for diagnostic ultrasound at this site underscores the clinical need and relevance.

# DOMAIN 2: INDEX TEST(S)

## A. Risk of Bias

**Describe the index test:**Triage-CADx system (AI Strategy, Warren NJ) using deep-learning CNN based on Inception-v3 architecture with TensorFlow. System classifies breast lesions as suspicious (biopsy recommended, red) or benign (no action, green), producing score 0-1. Three minimally trained healthcare workers (first-year medical student, surgical nurse, gynecologic intern) with NO prior ultrasound experience acquired orthogonal images using GE Vscan Dual Probe (8-MHz linear) after ~30 minutes training. CADx model trained/validated on external data (ACRIN 6666, Magee-Womens, USC/UCLA phase I) and tested on Mexico images - external validation. System evaluated each orthogonal image separately, used maximum score for final classification. CRITICAL: Threshold for binary classification (suspicious vs benign) NOT explicitly reported or stated to be pre-specified. Paper does not clarify whether red/green threshold was established during training or optimized on test data. Page reference: p.2, p.3, p.4

| Signaling Question | Answer |
| --- | --- |
| ❖ Interpreted without knowledge of reference? | YES |
| ❖ If threshold used, was it pre-specified? | UNCLEAR |

**Could the conduct of the index test have introduced bias?: RISK: UNCLEAR**

**Justification:** While the automated nature of the CADx system ensures blinding to reference standard results (the algorithm has no mechanism to know pathology outcomes), the threshold for classifying lesions as suspicious vs benign is not explicitly reported or stated to be pre-specified. The paper does not clarify whether the red/green classification threshold was established during the training phase on external data or was determined/optimized using the test set. This is important because threshold optimization on test data artificially inflates diagnostic accuracy. Given this is described as a prospective external validation study, the threshold was likely pre-specified during development, but this cannot be confirmed from the paper alone.

## B. Concerns regarding applicability

**Is there concern about the index test applicability?: CONCERN: LOW**

**Justification:** The index test represents a true point-of-care AI application: a portable, low-cost ultrasound device (GE Vscan Dual Probe) operated by minimally trained healthcare workers (30 minutes of training) with automated CADx interpretation. This matches the intended use case of AI-based clinical decision support for POC imaging in resource-limited settings. The system was specifically designed for triage of palpable breast lumps in LMICs where radiologist expertise is scarce. The workflow (patient identifies lump, operator captures orthogonal images, CADx provides automated classification) is highly applicable to real-world POC implementation.

# DOMAIN 3: REFERENCE STANDARD

## A. Risk of Bias

**Describe the reference standard:**SEVERE DIFFERENTIAL VERIFICATION BIAS: Reference standard was NOT uniform. For BI-RADS ≥4a masses (N=14, 44%), reference was histopathology via US-guided core biopsy per hospital standard. For BI-RADS 2 (N=13) or BI-RADS 3 (N=5), totaling 18/32 (56%), NO histopathologic confirmation - classified as benign based SOLELY on radiologist BI-RADS assessment. CIRCULARITY: Decision of which reference to apply determined by initial radiologist assessment. Hospital radiologist (A.P.G.M., 3 years experience) performed targeted US using Philips HD11XE, provided BI-RADS. Breakdown: BI-RADS 6 (1) + BI-RADS 5 (1) + BI-RADS 4b (1) + BI-RADS 4a (11) = 14 with histopathology (2 cancers: invasive ductal carcinoma; 12 benign: 7 fibroadenomas, 2 simple cysts, 1 fibrocystic disease, 1 intraductal papilloma). BI-RADS 3 (5) + BI-RADS 2 (13) = 18 with NO histopathology, assumed benign. FALSE NEGATIVES among unbiopsied patients UNDETECTABLE. BI-RADS 3 has up to 2% malignancy risk per ACR guidelines. Page reference: p.4-5

| Signaling Question | Answer |
| --- | --- |
| ❖ Is reference standard likely to correctly classify? | NO |
| ❖ Interpreted without knowledge of index test? | NO |

**Could the reference standard have introduced bias?: RISK: HIGH**

**Justification:** SEVERE DIFFERENTIAL VERIFICATION BIAS is present. Only 14/32 patients (44%) received histopathology (gold standard), while 18/32 (56%) were classified as benign based solely on radiologist BI-RADS assessment without histopathologic confirmation. The decision of which reference standard to apply was determined by the initial radiologist assessment, creating circularity: patients deemed low suspicion by radiologist (BI-RADS 2-3) did not receive biopsy and were assumed benign. If the radiologist or CADx missed a cancer in this group, it would not be detected. This fundamentally compromises validity of the reported perfect diagnostic accuracy (100% sensitivity, 100% specificity, AUC 1.0). BI-RADS 3 lesions have up to 2% malignancy risk per ACR guidelines.

## B. Concerns regarding applicability

**Is there concern about reference standard applicability?: CONCERN: UNCLEAR**

**Justification:** For the 14 patients who received it, histopathology (US-guided core biopsy) is the appropriate gold standard for breast lesion diagnosis. However, for 56% of patients, the reference was radiologist BI-RADS assessment alone - a subjective interpretation rather than definitive diagnosis. The radiologist had only 3 years of experience. This creates applicability uncertainty because the reference standard for most patients was not the accepted gold standard (tissue diagnosis) but rather an intermediate clinical assessment that itself requires validation.

# DOMAIN 4: FLOW AND TIMING

## A. Risk of Bias

**Patient flow:**SEVERE PARTIAL VERIFICATION BIAS: All 32 patients received index test (CADx analysis of portable ultrasound images), but only 14/32 (44%) received gold standard reference (histopathology). The 18/32 patients (56%) with BI-RADS 2-3 masses received NO histopathologic verification - classified as benign based solely on radiologist assessment. The decision of which reference standard to apply (histopathology vs radiologist assessment alone) was determined by the radiologist BI-RADS score, creating differential verification. No information provided on time interval between index test and reference standard. All 32 patients appear included in reported 2×2 table, but the reference standard was not uniform. Page reference: p.4-5

| Signaling Question | Answer |
| --- | --- |
| ❖ Appropriate interval between tests? | UNCLEAR |
| ❖ Did all patients receive reference standard? | NO |
| ❖ Did patients receive same reference standard? | NO |
| ❖ Were all patients included in analysis? | YES |

**Could the patient flow have introduced bias?: RISK: HIGH**

**Justification:** SEVERE PARTIAL AND DIFFERENTIAL VERIFICATION BIAS present. Only 14/32 patients (44%) received gold standard histopathology, while 18/32 (56%) received only radiologist BI-RADS assessment without tissue confirmation. The reference standard pathway was determined by the radiologist assessment, creating circularity and preventing detection of false negatives among unbiopsied patients. False negatives in the BI-RADS 2-3 group are completely undetectable in this design. This fundamentally invalidates the reported perfect diagnostic accuracy (100% sensitivity, 100% specificity). All 32 patients appear included in analysis, but non-uniform reference standard creates severe bias.

# OVERALL ASSESSMENT SUMMARY

**HIGH RISK OF BIAS - SEVERE DIFFERENTIAL VERIFICATION**

| Domain | Signaling Questions | Risk of Bias | Applicability |
| --- | --- | --- | --- |
| D1: Patient Selection | 1.1 Unclear 1.2 Yes 1.3 Yes | UNCLEAR | LOW |
| D2: Index Test | 2.1 Yes 2.2 Unclear | UNCLEAR | LOW |
| D3: Reference Standard | 3.1 No 3.2 No | HIGH | UNCLEAR |
| D4: Flow and Timing | 4.1 Unclear 4.2 No 4.3 No 4.4 Yes | HIGH | N/A |

**Overall Risk of Bias: HIGH**

**Justification:** This study has HIGH overall risk of bias primarily due to SEVERE DIFFERENTIAL VERIFICATION BIAS affecting both Domain 3 (Reference Standard) and Domain 4 (Flow and Timing). Only 14/32 patients (44%) received histopathology (the gold standard), while 18/32 patients (56%) were classified as benign based solely on radiologist BI-RADS assessment without histopathologic confirmation. The decision of which reference standard to apply was determined by the initial radiologist assessment, creating circularity: patients deemed low suspicion by the radiologist (BI-RADS 2-3) did not receive biopsy and were assumed benign - but if the radiologist or CADx missed a cancer in this group, it would not be detected. This fundamentally compromises the validity of the reported perfect diagnostic accuracy (100% sensitivity, 100% specificity, AUC 1.0). Additionally, the enrollment method is unclear (Domain 1) and the index test threshold specification is unclear (Domain 2), though these are lesser concerns.

**Overall Applicability Concern: LOW**

**Justification:** Despite the methodological limitations, this study has excellent applicability to POC AI imaging. The study directly addresses the intended use case: AI-based triage of palpable breast masses using low-cost portable ultrasound operated by minimally trained healthcare workers in a resource-limited LMIC setting. The GE Vscan Dual Probe device, 30-minute training protocol, and automated CADx classification represent a realistic POC workflow. The patient population (women presenting with palpable breast masses to a government hospital with 9-month diagnostic wait times) is highly representative of the target clinical scenario.

## Critical Biases Identified

**1. DIFFERENTIAL VERIFICATION BIAS (SEVERITY: SEVERE):**Different patients received different reference standards based on initial test results. 14/32 (44%) with BI-RADS ≥4a received histopathology; 18/32 (56%) with BI-RADS 2-3 received only radiologist assessment. Reported perfect accuracy (100% sensitivity, 100% specificity, AUC 1.0) is artificially inflated because false negatives among unbiopsied BI-RADS 2-3 patients cannot be detected. BI-RADS 3 lesions have up to 2% malignancy risk per ACR guidelines. Circularity exists: radiologist assessment both determines and serves as reference standard for 56% of patients.

**2. PARTIAL VERIFICATION BIAS (SEVERITY: SEVERE):**Only 44% of patients received definitive reference standard (histopathology); 56% had no tissue diagnosis.

**3. SAMPLE SIZE LIMITATION (SEVERITY: MODERATE):**Very small pilot study with only 32 patients and 2 cancers (6% prevalence). Perfect diagnostic accuracy (2/2 cancers detected, 30/30 benign correctly classified) should be interpreted with extreme caution. Confidence intervals would be extremely wide - detecting 2/2 cancers could occur by chance even with a less accurate system. Authors acknowledge: In practice and with a larger number of patient cases, we expect these numbers to change.

## Study Value Despite Methodological Limitations

**Despite severe verification bias preventing valid diagnostic accuracy assessment, this pilot study demonstrates:

• Feasibility:** Minimally trained operators (30 min training) can successfully acquire breast ultrasound images
**• Technical proof-of-concept:** AI system can process images from low-cost portable devices (GE Vscan)
**• Workflow applicability:** POC triage workflow is implementable in resource-limited settings
**• External validation:** CADx trained on US data tested successfully on Mexico data with different equipment
**• Clinical need:** 9-month wait time for diagnostic ultrasound demonstrates urgency of POC solutions

## Reported Performance (UNRELIABLE)

**WARNING: Performance estimates are UNRELIABLE due to verification bias

Reported CADx Performance:**• Sensitivity: 100% (2/2 cancers)
• Specificity: 100% (30/30 benign)
• AUC: 1.0 (perfect discrimination)

**CRITICAL INTERPRETATION ISSUES:**1. Only 2 cancers total - VERY small sample
2. 56% of patients had NO gold standard verification
3. Perfect accuracy likely ARTIFACTUALLY INFLATED
4. Confidence intervals would be EXTREMELY WIDE
5. False negatives among 18 unbiopsied patients UNDETECTABLE

**CONFIDENCE IN RESULTS:** Very low - differential verification bias, partial verification bias, and very small sample size make these performance estimates unreliable for clinical decision-making. Larger studies with uniform histopathologic reference standard are needed.

QUADAS-2

**Malherbe et al. 2025**Revolutionizing Breast Cancer Screening: Integrating Artificial Intelligence With Clinical Examination for Targeted Care in South Africa *Study ID: Malherbe_2025 | Assessment Date: 2025-11-28*

____________________________________________________________________________________________________

# Phase 1: State the Review Question

*Patients (setting, intended use of index test, presentation, prior testing):*

| Women aged 25-85 years presenting to Daspoort PoliClinic in Gauteng Province, South Africa, over 6 months. Setting: Point-of-care - primary care polyclinic in LMIC (South Africa). Intended use: AI-enabled clinical decision support using POC ultrasound for breast cancer screening to determine need for further workup. Presentation: Women presenting for general health-care services who were educated about and invited to participate in breast screening. Inclusion: Age 25-85 years with or without clinical history of breast cancer/symptoms. Exclusions: Age <25 or >85 years, unwilling to provide consent. CRITICAL DISCREPANCY: Abstract states 1,617 women screened; Discussion mentions 530 with clinical signs; Final analysis (Table 2) reports only 203 patients. Massive unexplained attrition (87% excluded) with no flow diagram or explanation. Final cohort: 203 patients (202 female, 1 male), age 25-85 years, clustering in 30-63 age groups. Predominantly urban setting (Gauteng Province). Disease prevalence UNKNOWN - no histopathology performed. Breast AI classified 5/203 (2.46%) as positive; CBE classified 2/203 (0.99%) as positive. NOTE: This is a COMPARISON study between two screening tests (CBE and Breast AI), NOT a diagnostic accuracy study against gold standard. |
| --- |

*Index test(s):*

| Breast AI application (Malherbe, 2021) - Android-based AI software registered with South African Health Products Regulatory Authority (SAHPRA) as Type A Medical Device for diagnostic use. AI trained and validated on over 40,000 histologically confirmed breast cancer masses visualized through breast ultrasound over 3 years, achieving 97.6% accuracy in prior validation. Equipment: Wireless FDA-approved Clarius POCUS probes. Operator: Qualified general practitioner registered with HPCSA Medical and Dental Council, experienced in sonographic imaging. AI provided risk stratification outputs and BI-RADS grading. Risk prediction ranged 0-25% in this cohort. BI-RADS classifications: BIRADS 2, 3, 4, 5. LIMITATIONS: (1) Blinding protocol not explicitly described - unclear if operator knew clinical findings when acquiring images. (2) Specific threshold values for BI-RADS classifications and risk percentages NOT explicitly stated as pre-specified for this study. (3) AI originally trained/validated on 40,000+ images but may not have been from portable Clarius POCUS devices - equipment compatibility not addressed. NOTE: This was used as index test being evaluated, but study lacks reference standard for comparison. |
| --- |

*Reference standard and target condition:*

| CRITICAL FLAW - COMPLETE ABSENCE OF REFERENCE STANDARD: NO reference standard was applied to ANY patient in this study. NO histopathological confirmation was performed. NO gold standard verification of ANY kind. The study compared two index tests (Clinical Breast Examination by nurses vs Breast AI ultrasound by GP) but did NOT verify results against tissue diagnosis, imaging follow-up, or any accepted reference standard for breast cancer. This is NOT differential or partial verification bias - it is COMPLETE ABSENCE of verification. Without a reference standard, TRUE disease status is unknown, making it impossible to calculate sensitivity, specificity, positive predictive value, negative predictive value, or any diagnostic accuracy metric. The study can only assess AGREEMENT between CBE and Breast AI (using McNemar's test, p.5), NOT accuracy of either test. Target condition (breast cancer) cannot be confirmed in any patient. Study essentially demonstrates that Breast AI detected 4 additional suspicious cases compared to CBE (5 vs 2 positives), but whether these were true positives or false positives is UNKNOWN. This is fundamentally a comparison/agreement study, NOT a diagnostic accuracy study. |
| --- |

# Phase 2: Draw a Flow Diagram for the Primary Study

| PATIENT FLOW DIAGRAM - COMPARISON STUDY (NOT DIAGNOSTIC ACCURACY)  STUDY PERIOD: 6 months (dates not specified) SETTING: Daspoort PoliClinic, Gauteng Province, South Africa (Primary care polyclinic in urban LMIC setting)  ↓ COMMUNITY EDUCATION: 1,730 patients educated at local screening events in rural villages  ↓ SCREENED: 1,617 women aged 25-85 years (per Abstract - using CBE and Breast AI)  ↓ WITH CLINICAL SIGNS: 530 patients (palpable lumps, mastalgia, risk factors per Discussion p.6)  ↓ ⚠️ MASSIVE UNEXPLAINED ATTRITION ⚠️  ↓ ANALYZED: 203 patients (per Table 2) • 1,414 patients (87%) EXCLUDED with NO explanation • NO flow diagram provided • NO justification for exclusions  ↓ FINAL COHORT CHARACTERISTICS: • 202 females (99.51%) • 1 male (0.49%) • Age: 25-85 years (clustering 30-63) • Predominantly urban population  ═══════════════════════════════════════════════════════════════════════ TWO INDEX TESTS COMPARED (NO REFERENCE STANDARD) ═══════════════════════════════════════════════════════════════════════  INDEX TEST 1: Clinical Breast Examination (CBE) Performed by: Nurses Method: Physical palpation  INDEX TEST 2: Breast AI Ultrasound Equipment: Clarius wireless POCUS probes (FDA-approved) Operator: GP experienced in sonographic imaging (HPCSA registered) AI: Breast AI app (SAHPRA Type A Medical Device)  • Trained on 40,000+ histologically confirmed cases  • Prior validation: 97.6% accuracy Outputs: Risk stratification (0-25% in this cohort)  BI-RADS classifications (2, 3, 4, 5)  COMPARISON RESULTS (n=203):  CBE Results: • Positive: 2 (0.99%) • Negative: 201 (99.01%)  Breast AI Results: • Positive: 5 (2.46%) • Negative: 198 (97.04%)  BI-RADS Distribution (Breast AI): • No BI-RADS 5 cases (immediate intervention needed) • Risk range: 0-25% • Predominantly negative classifications (97.04%)  AGREEMENT ANALYSIS: • McNemar's test performed (p.5) • Breast AI detected 4 ADDITIONAL cases vs CBE • Agreement between tests assessed  ═══════════════════════════════════════════════════════════════════════ CRITICAL FLAW: COMPLETE ABSENCE OF REFERENCE STANDARD ═══════════════════════════════════════════════════════════════════════  NO VERIFICATION OF ANY KIND: ├─→ NO histopathology (tissue diagnosis) ├─→ NO follow-up imaging ├─→ NO clinical outcomes tracking ├─→ NO gold standard applied to ANY patient └─→ TRUE disease status UNKNOWN for ALL 203 patients  CONSEQUENCES: • Cannot calculate sensitivity (unknown true positives) • Cannot calculate specificity (unknown true negatives) • Cannot calculate PPV or NPV • Cannot assess diagnostic accuracy of EITHER test • Can only assess AGREEMENT between two tests  FUNDAMENTAL PROBLEM: Breast AI found 4 additional suspicious cases vs CBE (5 vs 2) → Are these TRUE POSITIVES (missed cancers) or FALSE POSITIVES? → IMPOSSIBLE TO DETERMINE without reference standard  STUDY TYPE: This is a COMPARISON/AGREEMENT study, NOT diagnostic accuracy study • Compares two screening tests against each other • Does NOT validate either test against gold standard • Cannot inform clinical decision-making re: accuracy  ═══════════════════════════════════════════════════════════════════════ ADDITIONAL METHODOLOGICAL ISSUES ═══════════════════════════════════════════════════════════════════════  1. MASSIVE UNEXPLAINED EXCLUSIONS:  • 1,617 screened → 203 analyzed  • 87% excluded with NO explanation  • NO flow diagram  • Selection bias likely  2. SPECTRUM BIAS (Cannot assess):  • Screening population (mostly asymptomatic)  • 97% classified as negative by Breast AI  • True disease spectrum unknown without reference  3. OPERATOR BIAS:  • Same clinical setting for both tests  • Not explicitly blinded to each other's findings  4. POTENTIAL CONFLICTS:  • Funded by AstraZeneca  • Corresponding author email: kathryn@medsol.ai  • Possible connection to Breast AI technology  STUDY VALUE (Limited): • Demonstrates POC workflow feasibility • Shows AI can process Clarius POCUS images • Indicates AI detects more suspicious cases than CBE • BUT: Cannot determine if additional detections are beneficial  (true positives) or harmful (false positives) |
| --- |

# Phase 3: Risk of Bias and Applicability Judgments

# DOMAIN 1: PATIENT SELECTION

## A. Risk of Bias

**Describe methods of patient selection:**Study conducted at Daspoort PoliClinic, Gauteng, South Africa over 6 months. 1,617 women aged 25-85 screened using CBE and Breast AI (Abstract). Additionally 1,730 educated during rural village screening events, with 530 subsequently screened at clinic. Final analysis performed on 203 patients (Table 2) - paper does NOT explain discrepancy between 1,617 screened and 203 analyzed. Prospective comparative cohort design. Inclusion: women 25-85 years with/without breast cancer history/symptoms. Exclusions: age <25 or >85, unwilling to consent. Enrollment appears to be convenience sampling from clinic attendees who agreed to participate after education. MASSIVE ATTRITION: 1,414 patients (87%) excluded between screening and analysis with NO flow diagram or explanation. Page reference: p.1, p.4

| Signaling Question | Answer |
| --- | --- |
| ❖ Was a consecutive or random sample enrolled? | UNCLEAR |
| ❖ Was a case-control design avoided? | YES |
| ❖ Did study avoid inappropriate exclusions? | UNCLEAR |

**Could the selection of patients have introduced bias?: RISK: HIGH**

**Justification:** The study appears to use convenience sampling from willing clinic attendees rather than consecutive/random enrollment. Most critically, there is MASSIVE unexplained attrition: 1,617 women screened but only 203 analyzed (87% excluded). No flow diagram provided and no explanation given for exclusion of 87% of screened population. This creates high risk of selection bias as analyzed sample may not be representative. Exclusions for age and consent are appropriate, but the 1,414 patient loss is unjustified. The population was predominantly urban (Gauteng Province), limiting generalizability to rural settings as authors acknowledge.

## B. Concerns regarding applicability

**Is there concern that the included patients do not match the review question?: CONCERN: LOW**

**Justification:** The study population matches the review question well. This represents AI-enabled clinical decision support using point-of-care ultrasound for breast cancer screening in a primary care polyclinic in a low- and middle-income country (South Africa). The POC context with portable ultrasound (Clarius wireless probes) in a resource-limited primary care facility aligns with the review scope. Women presented with breast-related symptoms or for screening, representing a real-world clinical population.

# DOMAIN 2: INDEX TEST(S)

## A. Risk of Bias

**Describe the index test:**Breast AI application (Malherbe 2021) - Android-based AI software registered with SAHPRA as Type A Medical Device. AI trained/validated on 40,000+ histologically confirmed breast cancer masses over 3 years, achieving 97.6% prior accuracy. Images acquired using wireless FDA-approved Clarius POCUS probes by qualified GP (HPCSA registered) experienced in sonographic imaging. AI provided risk stratification (0-25% range in cohort) and BI-RADS grading (2,3,4,5). LIMITATIONS: Blinding protocol not explicitly described - unclear if operator knew clinical findings when acquiring images. Specific threshold values for BI-RADS classifications NOT explicitly pre-specified for this study. Equipment compatibility unclear - AI trained on 40,000+ images but may not have been from portable Clarius devices. Page reference: p.4, p.6

| Signaling Question | Answer |
| --- | --- |
| ❖ Interpreted without knowledge of reference? | UNCLEAR |
| ❖ If threshold used, was it pre-specified? | UNCLEAR |

**Could the conduct of the index test have introduced bias?: RISK: UNCLEAR**

**Justification:** The automated nature of AI interpretation suggests the algorithm was blind to reference standard results (though no reference was actually applied). However, operator blinding is not addressed. While AI was previously validated achieving 97.6% accuracy on 40,000+ cases, the specific thresholds for this study's BI-RADS classifications and risk percentages are not explicitly stated as pre-specified. The operator (GP experienced in sonographic imaging) conducted breast ultrasounds, but their training in using this specific AI system and whether clinical judgment influenced image acquisition is not detailed. Use of portable Clarius POCUS probes may differ from equipment used in AI's original training/validation - potential performance impact not addressed.

## B. Concerns regarding applicability

**Is there concern about the index test applicability?: CONCERN: LOW**

**Justification:** The index test is highly applicable to POC imaging. Breast AI app is SAHPRA-registered medical device used with FDA-approved portable Clarius POCUS probes operated by GP in primary care polyclinic - represents realistic POC implementation. Risk stratification (BI-RADS) and automated AI interpretation align with intended use for clinical decision support. Setting (resource-limited LMIC primary care) matches target context for AI-assisted POC breast screening. The workflow is directly relevant to review question.

# DOMAIN 3: REFERENCE STANDARD

## A. Risk of Bias

**Describe the reference standard:**COMPLETE ABSENCE OF REFERENCE STANDARD: NO reference standard was applied to ANY patient. NO histopathological confirmation performed. NO gold standard verification of ANY kind. Study compared two index tests (CBE by nurses vs Breast AI ultrasound by GP) but did NOT verify results against tissue diagnosis, imaging follow-up, or any accepted reference standard. This is NOT differential or partial verification bias - it is COMPLETE ABSENCE of verification. Without reference standard, TRUE disease status unknown for all 203 patients, making it impossible to calculate sensitivity, specificity, PPV, NPV, or any diagnostic accuracy metric. Study can only assess AGREEMENT between CBE and Breast AI (McNemar's test), NOT accuracy of either test. Target condition (breast cancer) cannot be confirmed in any patient. Breast AI detected 4 additional suspicious cases vs CBE (5 vs 2 positives), but whether these were true positives or false positives is UNKNOWN. Page reference: p.5 (Results)

| Signaling Question | Answer |
| --- | --- |
| ❖ Is reference standard likely to correctly classify? | NO |
| ❖ Interpreted without knowledge of index test? | NO |

**Could the reference standard have introduced bias?: RISK: HIGH**

**Justification:** There is NO reference standard in this study - this represents complete absence of verification, which is more severe than differential or partial verification bias. No patients received histopathological confirmation, imaging follow-up, or any gold standard verification. The study compares agreement between two screening tests (CBE and Breast AI) but cannot assess diagnostic accuracy of either because true disease status is unknown. This is a fundamental methodological flaw that makes the study unsuitable for evaluating diagnostic test performance. Without knowing which patients actually have breast cancer, sensitivity and specificity cannot be calculated. The study can only demonstrate that Breast AI flags more cases as suspicious than CBE, but whether this represents improved detection (true positives) or increased false alarms is impossible to determine.

## B. Concerns regarding applicability

**Is there concern about reference standard applicability?: CONCERN: HIGH**

**Justification:** The complete absence of any reference standard represents a high applicability concern. Without histopathological confirmation or any accepted gold standard for breast cancer diagnosis, the target condition itself cannot be confirmed. This is not a matter of using an imperfect reference - there is NO reference at all. The study cannot inform clinical decision-making about diagnostic accuracy because true disease status is unknown. While the setting and population are applicable to POC screening, the lack of outcome verification means results cannot be generalized or applied to real-world diagnostic decisions.

# DOMAIN 4: FLOW AND TIMING

## A. Risk of Bias

**Patient flow:**SEVERE PATIENT FLOW PROBLEMS: (1) COMPLETE ABSENCE OF VERIFICATION - No patients received reference standard of any kind. All 203 received both index tests (CBE and Breast AI) but NONE received histopathology or gold standard verification. (2) MASSIVE UNEXPLAINED EXCLUSIONS - 1,617 women screened but only 203 analyzed (87% excluded). No flow diagram provided. No explanation for excluding 1,414 patients. (3) NO UNIFORM REFERENCE - Not only differential verification, but ZERO verification. Study reports that all 203 patients completed both screening tests (CBE and Breast AI), but without reference standard verification, true disease status unknown for all. No information on time interval between tests. Study design prevents assessment of diagnostic accuracy - can only assess agreement between two imperfect screening tests. Page reference: p.4-5

| Signaling Question | Answer |
| --- | --- |
| ❖ Appropriate interval between tests? | NO |
| ❖ Did all patients receive reference standard? | NO |
| ❖ Did patients receive same reference standard? | NO |
| ❖ Were all patients included in analysis? | NO |

**Could the patient flow have introduced bias?: RISK: HIGH**

**Justification:** SEVERE BIAS on multiple fronts: (1) Complete absence of reference standard - NO patients received gold standard verification of disease status, making diagnostic accuracy assessment impossible. (2) Massive unexplained exclusions - 87% of screened population (1,414/1,617 patients) excluded from analysis without justification or flow diagram. This creates severe selection bias. (3) No time interval information provided between index tests or between testing and any theoretical follow-up. The study fundamentally cannot address diagnostic accuracy because it lacks the necessary verification pathway. It is a comparison study demonstrating agreement between two screening tests, not a diagnostic accuracy study.

# OVERALL ASSESSMENT SUMMARY

**VERY HIGH RISK OF BIAS - FUNDAMENTAL METHODOLOGICAL FLAWS**

| Domain | Signaling Questions | Risk of Bias | Applicability |
| --- | --- | --- | --- |
| D1: Patient Selection | 1.1 Unclear 1.2 Yes 1.3 Unclear | HIGH | LOW |
| D2: Index Test | 2.1 Unclear 2.2 Unclear | UNCLEAR | LOW |
| D3: Reference Standard | 3.1 No 3.2 No | HIGH | HIGH |
| D4: Flow and Timing | 4.1 No 4.2 No 4.3 No 4.4 No | HIGH | N/A |

**Overall Risk of Bias: VERY HIGH**

**Justification:** This study has VERY HIGH risk of bias with 3 domains rated HIGH (Patient Selection, Reference Standard, Flow and Timing). The most critical flaw is the COMPLETE ABSENCE OF ANY REFERENCE STANDARD - no histopathological confirmation, no imaging follow-up, no gold standard verification of any kind was performed. Without knowing true disease status, the study cannot assess diagnostic accuracy - it can only measure agreement between two screening tests (CBE and Breast AI). Additionally, there is massive unexplained patient attrition (1,617 screened → 203 analyzed = 87% excluded) with no flow diagram or justification. These fundamental methodological limitations mean this study cannot provide valid estimates of sensitivity, specificity, or diagnostic accuracy for the Breast AI system. The study essentially demonstrates that Breast AI flags more suspicious cases than CBE (5 vs 2), but whether these additional detections represent true positives (improved cancer detection) or false positives (unnecessary workup) is completely unknown.

**Overall Applicability Concern: MODERATE**

**Justification:** The study has mixed applicability. The population (women at primary care polyclinic in LMIC), setting (POC with portable ultrasound), and intended use (breast cancer screening with AI decision support) match the review question well. However, the COMPLETE ABSENCE of any reference standard creates a high applicability concern for Domain 3, as the target condition (breast cancer) cannot be confirmed. Without verification, results cannot inform clinical decisions about diagnostic accuracy or guide implementation. The study demonstrates workflow feasibility but not clinical validity.

## Critical Methodological Flaws

**1. COMPLETE ABSENCE OF REFERENCE STANDARD (SEVERITY: FATAL):**NO histopathology, NO imaging follow-up, NO gold standard verification of ANY kind for ANY patient. True disease status unknown for all 203 patients. Study compares two index tests (CBE vs Breast AI) but cannot validate either against actual cancer diagnosis. This is more severe than partial or differential verification - it is COMPLETE absence of verification. Cannot calculate sensitivity, specificity, PPV, NPV, or any diagnostic accuracy metric. Breast AI detected 4 additional suspicious cases vs CBE, but impossible to determine if these are true positives or false positives.

**2. MASSIVE UNEXPLAINED EXCLUSIONS (SEVERITY: SEVERE):**1,617 women screened → 203 analyzed (87% excluded = 1,414 patients lost). NO flow diagram provided. NO explanation for exclusions. Creates severe selection bias - analyzed sample likely not representative of screened population.

**3. STUDY DESIGN MISCHARACTERIZATION:**This is a COMPARISON/AGREEMENT study, NOT a diagnostic accuracy study. Appropriate for assessing whether two tests agree (McNemar test), but inappropriate for evaluating diagnostic performance without gold standard.

**4. POTENTIAL CONFLICTS OF INTEREST:**Funded by AstraZeneca. Corresponding author email: kathryn@medsol.ai (suggests possible connection to Breast AI technology). Authors state no relevant disclosures but potential conflict should be noted.

## Limited Study Value

**Despite fundamental flaws preventing diagnostic accuracy assessment, study provides MINIMAL value:

• POC workflow feasibility:** Demonstrates GP can operate Clarius POCUS with Breast AI in primary care setting
**• Technical compatibility:** Shows Breast AI can process images from portable Clarius devices
**• Comparison finding:** Breast AI flagged 5/203 (2.46%) as positive vs CBE 2/203 (0.99%) - but clinical significance unknown

**CANNOT INFORM:** Clinical decision-making, implementation decisions, cost-effectiveness, patient outcomes, diagnostic accuracy, screening program design

## What Study Reports (Unreliable for Accuracy)

Sample: 203 patients (202 female, 1 male)

**CBE Results:**• Positive: 2 (0.99%)
• Negative: 201 (99.01%)

**Breast AI Results:**• Positive: 5 (2.46%)
• Negative: 198 (97.04%)
• Risk range: 0-25%
• No BI-RADS 5 cases

**Comparison:**• Breast AI detected 4 additional suspicious cases vs CBE
• McNemar test assessed agreement between tests

**CRITICAL LIMITATION:** Without reference standard, impossible to determine if these additional detections represent:
 → BENEFIT (true positives = improved cancer detection)
 → HARM (false positives = unnecessary anxiety/workup)

QUADAS-2

**Marquez et al. 2025**Performance of chest X-ray with computer-aided detection powered by deep learning-based artificial intelligence for tuberculosis presumptive identification during case finding in the Philippines  *Study ID: Marquez_2025 | Assessment Date: 2025-11-28*

____________________________________________________________________________________________________

# Phase 1: State the Review Question

*Patients (setting, intended use of index test, presentation, prior testing):*

| Individuals aged ≥15 years screened for tuberculosis through USAID's TBIHSS project in four regions of Philippines (Regions 3, 4A, 7, and National Capital Region) between May 2021 and March 2024. Setting: Point-of-care and community - three screening pathways: (1) Active case-finding in communities (ACF-comm, 58%), (2) Active case-finding in workplaces (ACF-WP, 31%), (3) Intensified case-finding in hospitals (ICF, 11%). Intended use: AI-CAD for TB screening using CXR to identify presumptive TB cases requiring confirmatory testing. Presentation: Community members, workplace employees, and hospital attendees screened through TB case-finding activities. Population characteristics: Mean age 50 years, 55% male, 26% with prior TB treatment history. Total screened: 52,840 individuals; CXR performed: 47,817 (90.5%). CRITICAL VERIFICATION BIAS: Only 5,740/47,817 (12%) received both CXR and reference standard mWRD. Exclusions: 40,486 CAD-negative/symptom-negative individuals not tested per programmatic algorithm (84.7% of CXR recipients); 629 CAD-positive individuals unable to provide sputum; 962 pending results. Final analytical sample: 5,740 with both tests - severely biased composition: 4,306 CAD-positive (75%) + 1,432 CAD-negative but symptomatic/radiologist-flagged (25%). This is NOT a representative screening population but a highly enriched subset. High TB-burden country setting (Philippines). |
| --- |

*Index test(s):*

| qXR version 3 (Qure.ai, India) - deep learning-based computer-aided detection (AI-CAD) software for chest X-ray analysis. All screened individuals received postero-anterior CXR with DICOM images processed by qXR, providing abnormality score 0-1. Classification: Score ≥0.50 = TB presumptive (CAD-positive); Score <0.50 = TB negative (CAD-negative). Threshold: Pre-specified at 0.50 based on 2020 pilot implementation experience, set to align with Philippine national context, remained unchanged throughout 2021-2024 scale-up implementation. AI-CAD interpretation automated without knowledge of mWRD results - CXR performed before sputum collection in screening workflow. WHO-evaluated commercial AI-CAD software designed for TB screening in programmatic settings. STRENGTH: Threshold pre-specified from pilot, not optimized on test data (though study does perform retrospective threshold analysis in Table 3, primary analysis used pre-specified 0.50). Automated processing eliminates review bias. Real-world programmatic implementation. |
| --- |

*Reference standard and target condition:*

| Molecular WHO-recommended rapid diagnostic test (mWRD), specifically GeneXpert. Positive mWRD = MTB detected (rifampicin-resistant or rifampicin-susceptible). CRITICAL FLAW - SEVERE PARTIAL VERIFICATION BIAS: Reference standard NOT applied to all participants. Per programmatic screening algorithm, ONLY TB-presumptive individuals (identified by CXR AI-CAD score ≥0.50 OR TB signs/symptoms OR official radiologist reading) were referred for mWRD testing. Individuals TB-negative by both symptoms AND CXR (AI-CAD and radiologist) did NOT undergo mWRD per national TB program policy. Results: 4,306/4,935 CAD-positive individuals received mWRD (87%); Only 1,432/41,920 CAD-negative individuals received mWRD (3.4%) - specifically those flagged presumptive by symptoms/radiologist reading. Positivity rate: 17% among CAD-positive vs 2% among CAD-negative tested. INCORPORATION BIAS: Reference standard application directly dependent on index test results. 40,486 CAD-negative/symptom-negative individuals (84.7% of CXR recipients) systematically excluded from mWRD - true false-negative rate CANNOT be determined. Authors acknowledge limitations using 'pseudo-sensitivity' and 'pseudo-specificity' terminology. Target condition: Tuberculosis (MTB detection). |
| --- |

# Phase 2: Draw a Flow Diagram for the Primary Study

| PATIENT FLOW DIAGRAM - PROGRAMMATIC TB SCREENING  STUDY PERIOD: May 2021 - March 2024 (3 years) SETTING: Four regions of Philippines (Regions 3, 4A, 7, NCR) PROJECT: USAID TB Innovations and Health Systems Strengthening (TBIHSS) Context: High TB-burden country, programmatic case-finding activities  ↓ TOTAL SCREENED: 52,840 individuals aged ≥15 years  SCREENING PATHWAYS: ├─→ ACF-comm (Active case-finding in communities): 58% ├─→ ACF-WP (Active case-finding in workplaces): 31% └─→ ICF (Intensified case-finding in hospitals): 11%  POPULATION: Mean age 50, 55% male, 26% prior TB history  ↓ RECEIVED CHEST X-RAY: 47,817 (90.5% of screened)  CXR NOT PERFORMED (5,023 individuals): • Recent external CXR completed • Under age 15 years • Refusal  ↓ ═══════════════════════════════════════════════════════════════════════ ALL 47,817 RECEIVED INDEX TEST: AI-CAD (qXR v3) ═══════════════════════════════════════════════════════════════════════  Equipment: Digital postero-anterior CXR (DICOM images) AI Software: qXR version 3 (Qure.ai, India)  • WHO-evaluated commercial AI-CAD  • Deep learning-based abnormality detection Processing: Automated score 0-1 Threshold: ≥0.50 = CAD-positive (TB presumptive)  <0.50 = CAD-negative (TB negative) Pre-specification: Threshold set in 2020 pilot, unchanged 2021-2024  INDEX TEST RESULTS: ├─→ CAD-POSITIVE (≥0.50): 4,935 individuals (10.3%) └─→ CAD-NEGATIVE (<0.50): 42,882 individuals (89.7%)  ↓ ═══════════════════════════════════════════════════════════════════════ CRITICAL: SEVERE PARTIAL VERIFICATION BIAS ═══════════════════════════════════════════════════════════════════════  PROGRAMMATIC ALGORITHM (Per National TB Policy): Only TB-presumptive individuals receive mWRD testing  TB-PRESUMPTIVE CRITERIA: 1. CAD-positive (AI-CAD score ≥0.50), OR 2. TB signs/symptoms, OR 3. Official radiologist reading suggests TB  TB-NEGATIVE CRITERIA: CAD-negative AND No symptoms AND Radiologist confirms negative → NO mWRD testing performed  ═══════════════════════════════════════════════════════════════════════ REFERENCE STANDARD PATHWAY (DIFFERENTIAL VERIFICATION) ═══════════════════════════════════════════════════════════════════════  PATHWAY 1: CAD-POSITIVE → mWRD Testing CAD-positive: 4,935 individuals ├─→ mWRD completed: 4,306 (87.3%) ├─→ Could not provide sputum: 629 (12.7%) └─→ mWRD positivity rate: 17%  PATHWAY 2: CAD-NEGATIVE → Selective mWRD Testing CAD-negative: 42,882 individuals  SUBSET A: CAD-negative BUT symptomatic/radiologist-flagged ├─→ TB-presumptive by symptoms/radiologist: ~1,432 ├─→ These DID receive mWRD └─→ mWRD positivity rate: 2% (33/1,432)  SUBSET B: CAD-negative AND asymptomatic AND radiologist-confirmed negative ├─→ TB-negative by all criteria: 40,486 (94.4% of CAD-negatives) ├─→ These DID NOT receive mWRD └─→ TRUE DISEASE STATUS UNKNOWN  Additional exclusions: 962 pending results at data cutoff  ↓ ═══════════════════════════════════════════════════════════════════════ VERIFICATION SUMMARY ═══════════════════════════════════════════════════════════════════════  RECEIVED BOTH TESTS (CXR + mWRD): 5,740 (12.0% of CXR recipients) ├─→ CAD-positive tested: 4,306 (75.0% of analytical sample) └─→ CAD-negative tested: 1,432 (24.9% of analytical sample)  • These are NOT representative CAD-negatives  • Highly selected: symptomatic or radiologist-flagged  • Only 3.4% of total CAD-negatives (1,432/42,882)  NOT VERIFIED: 42,077 (88.0% of CXR recipients) ├─→ CAD-positive not tested: 629 (sputum issues) ├─→ CAD-negative not tested: 40,486 (deemed TB-negative) └─→ Pending results: 962  ANALYTICAL SAMPLE COMPOSITION (n=5,740): • TB-positive: 767 (13.4%)  - From CAD-positive: 734 (95.7% of positives)  - From CAD-negative: 33 (4.3% of positives) • TB-negative: 4,973 (86.6%)  - From CAD-positive: 3,572 (71.8% of negatives)  - From CAD-negative: 1,399 (28.1% of negatives)  ═══════════════════════════════════════════════════════════════════════ REPORTED PERFORMANCE (PSEUDO-METRICS - BIASED) ═══════════════════════════════════════════════════════════════════════  AUTHORS ACKNOWLEDGE: "Pseudo-sensitivity" and "pseudo-specificity" based on partial testing subset, NOT true population metrics  Pseudo-sensitivity: 95.6% (734/767 TB-positive detected) Pseudo-specificity: 28.1% (1,399/4,973 TB-negative) AUROC: 0.82  CRITICAL INTERPRETATION ISSUES: 1. Sensitivity LIKELY OVERESTIMATED  • 40,486 untested CAD-negatives may contain missed TB cases  • True false-negative rate unknown  2. Specificity LIKELY UNDERESTIMATED  • CAD-positive group enriched with true TB (17% prevalence)  • Untested CAD-negative group likely lower prevalence  3. Sample composition SEVERELY BIASED  • 75% CAD-positive (vs 10.3% in screened population)  • CAD-negative tested (25%) are symptomatic subset, not representative  AUTHORS STATE: "Testing more CAD-negative individuals might influence the overall metrics and provide a more precise overview of the actual performance of the AI-CAD model" (p.12)  AUTHORS STATE: "Diagnosing more CAD-positive than CAD-negative would lead to an overestimation of sensitivity and an underestimation of specificity" (p.12)  ═══════════════════════════════════════════════════════════════════════ STUDY VALUE (Despite Verification Bias) ═══════════════════════════════════════════════════════════════════════  STRENGTHS: • Large programmatic implementation (47,817 CXR) • Pre-specified threshold from 2020 pilot (not optimized on test data) • Real-world operational data from high-burden setting • Appropriate transparency about limitations • Multiple screening pathways (community, workplace, hospital)  PROVIDES USEFUL DATA ON: • Feasibility of AI-CAD in programmatic implementation • Workflow integration in national TB program • Operational performance in resource-limited setting • Real-world referral patterns  CANNOT PROVIDE: • Unbiased diagnostic accuracy estimates • True sensitivity/specificity • Reliable false-negative rate • Generalizable performance metrics |
| --- |

# Phase 3: Risk of Bias and Applicability Judgments

# DOMAIN 1: PATIENT SELECTION

## A. Risk of Bias

**Describe methods of patient selection:**Retrospective cross-sectional study using data from TB case-finding activities (USAID TBIHSS project) in four Philippine regions, May 2021-March 2024. Of 52,840 screened, 47,817 (90.5%) underwent CXR. Three pathways: active case-finding in communities (58%), workplaces (31%), and intensified case-finding in hospitals (11%). Inclusion: age ≥15 years with complete CXR and mWRD results. Exclusions: 5,023 no CXR (recent external CXR, age <15, refusal); 40,486 CAD-negative/symptom-negative not referred for mWRD per national algorithm (84.7% of CXR recipients); 629 CAD-positive unable to provide sputum. Final sample: 5,740 (12% of CXR recipients). CRITICAL: Enrollment method within screening sites not explicitly described - appears operational/convenience sampling. Massive exclusion of CAD-negative individuals creates severely biased sample. Page reference: p.1, p.3-5

| Signaling Question | Answer |
| --- | --- |
| ❖ Was a consecutive or random sample enrolled? | UNCLEAR |
| ❖ Was a case-control design avoided? | YES |
| ❖ Did study avoid inappropriate exclusions? | NO |

**Could the selection of patients have introduced bias?: RISK: HIGH**

**Justification:** The study has HIGH risk of selection bias due to inappropriate exclusion of the vast majority of the screened population. Specifically, 40,486/47,817 (84.7%) of individuals who underwent CXR were excluded because they tested CAD-negative and were not referred for mWRD testing per the programmatic algorithm. This creates severe verification bias where the reference standard was only applied to a non-representative subset. The analytical sample of 5,740 individuals represents only 12% of those who received CXR, consisting almost entirely of CAD-positive individuals (4,306/5,740 = 75%) plus symptomatic/radiologist-flagged CAD-negative individuals (1,432/5,740 = 25%). This sampling structure makes it impossible to accurately estimate true sensitivity or specificity.

## B. Concerns regarding applicability

**Is there concern that the included patients do not match the review question?: CONCERN: LOW**

**Justification:** The study population is highly applicable to real-world TB screening in point-of-care and community settings. Participants were recruited from active case-finding in communities (58%), workplaces (31%), and health facilities (11%) in the Philippines, a high TB-burden country. The population included adults ≥15 years with typical demographic distribution (mean age 50, 55% male) and mixed TB risk factors including 26% with prior TB treatment history. This reflects the intended use population for AI-CAD TB screening tools in programmatic implementation in resource-limited settings.

# DOMAIN 2: INDEX TEST(S)

## A. Risk of Bias

**Describe the index test:**qXR version 3 (Qure.ai, India) - deep learning-based AI-CAD software for CXR analysis. All screened individuals received postero-anterior CXR with DICOM images processed by qXR, providing abnormality score 0-1. Threshold ≥0.50 = CAD-positive (TB presumptive); <0.50 = CAD-negative (TB negative). Threshold pre-specified at 0.50 based on 2020 pilot implementation experience, set to align with Philippine national context, remained unchanged throughout 2021-2024 scale-up. AI-CAD interpretation automated without knowledge of mWRD results - CXR performed before sputum collection. WHO-evaluated commercial software. STRENGTH: Pre-specified threshold avoids optimization bias (though study does retrospective threshold analysis in Table 3, primary analysis used 0.50). Automated processing eliminates review bias. Page reference: p.3, Fig. 1

| Signaling Question | Answer |
| --- | --- |
| ❖ Interpreted without knowledge of reference? | YES |
| ❖ If threshold used, was it pre-specified? | YES |

**Could the conduct of the index test have introduced bias?: RISK: LOW**

**Justification:** The index test (qXR AI-CAD) was conducted appropriately with low risk of bias. The AI system automatically processed CXR images at the point of screening, before any reference standard (mWRD) testing occurred, eliminating review bias. The classification threshold of 0.50 was pre-specified based on 2020 pilot data and applied consistently throughout the 2021-2024 implementation period, avoiding threshold optimization bias on the test data. While the study does perform retrospective threshold analysis to identify optimal cut-points (Table 3), the primary analysis used the pre-specified 0.50 threshold.

## B. Concerns regarding applicability

**Is there concern about the index test applicability?: CONCERN: LOW**

**Justification:** The index test is highly applicable to point-of-care TB screening. qXR version 3 is a commercially available, WHO-evaluated AI-CAD software designed specifically for TB screening in programmatic settings. The software was used as intended: analyzing digital postero-anterior CXR DICOM images and providing automated abnormality scores. The threshold of 0.50 was calibrated for the Philippine national context during pilot implementation. This represents realistic point-of-care implementation of AI-CAD for TB screening in a high-burden, resource-limited setting.

# DOMAIN 3: REFERENCE STANDARD

## A. Risk of Bias

**Describe the reference standard:**Molecular WHO-recommended rapid diagnostic test (mWRD), specifically GeneXpert. Positive = MTB detected (rifampicin-resistant or susceptible). CRITICAL FLAW - SEVERE PARTIAL VERIFICATION: Reference NOT applied to all participants. Per programmatic algorithm, ONLY TB-presumptive individuals (CXR AI-CAD ≥0.50 OR TB symptoms OR radiologist reading) received mWRD. TB-negative by both symptoms AND CXR did NOT undergo mWRD per national policy. Results: 4,306/4,935 CAD-positive received mWRD (87%); only 1,432/42,882 CAD-negative received mWRD (3.4%) - those flagged by symptoms/radiologist. Positivity: 17% among CAD-positive vs 2% among CAD-negative tested. INCORPORATION BIAS: Reference application directly dependent on index test. 40,486 CAD-negative/symptom-negative individuals (84.7% of CXR recipients) excluded from mWRD - true false-negative rate CANNOT be determined. Authors use 'pseudo-sensitivity/specificity' terminology. Page reference: p.1-4, Fig. 1

| Signaling Question | Answer |
| --- | --- |
| ❖ Is reference standard likely to correctly classify? | YES |
| ❖ Interpreted without knowledge of index test? | NO |

**Could the reference standard have introduced bias?: RISK: HIGH**

**Justification:** The reference standard (mWRD/GeneXpert) itself is appropriate for TB diagnosis and likely classifies disease correctly when applied. However, there is HIGH risk of bias due to INCORPORATION BIAS - the decision to apply the reference standard was directly dependent on the index test results. Only TB-presumptive individuals (including CAD-positive) received mWRD testing, while CAD-negative/symptom-negative individuals did not. This creates a fundamental bias where the index test result determines whether the reference standard is applied. Additionally, 40,486/47,817 (84.7%) CAD-negative individuals were not verified, preventing accurate assessment of false-negative rate.

## B. Concerns regarding applicability

**Is there concern about reference standard applicability?: CONCERN: LOW**

**Justification:** The reference standard (mWRD/GeneXpert) is appropriate and applicable to TB diagnosis. GeneXpert is a WHO-recommended molecular diagnostic test that serves as the reference standard for TB detection in programmatic settings. The test appropriately identifies MTB presence. The concern is not with the reference standard itself, but rather with its selective application to the population (which is addressed under Risk of Bias).

# DOMAIN 4: FLOW AND TIMING

## A. Risk of Bias

**Patient flow:**SEVERE PARTIAL VERIFICATION BIAS: Only 12% of individuals who received index test also received reference standard (5,740/47,817), with decision to test directly contingent on index test results and clinical findings. 40,486 CAD-negative/symptom-negative individuals (84.7% of CXR recipients) systematically excluded from mWRD testing - true false-negative rate cannot be determined. Authors explicitly acknowledge using 'pseudo-sensitivity' and 'pseudo-specificity' based on 'partial testing', noting that 'diagnosing more CAD-positive than CAD-negative would lead to overestimation of sensitivity and underestimation of specificity.' CAD-negative individuals tested (n=1,432) represent only highly selected symptomatic/radiologist-flagged subset, yielding 2% positivity vs 17% among CAD-positive. Same reference standard (mWRD) applied to all who were tested. Time interval appropriate (CXR before sputum). Page reference: p.1, p.4, p.12, Fig. 1

| Signaling Question | Answer |
| --- | --- |
| ❖ Appropriate interval between tests? | YES |
| ❖ Did all patients receive reference standard? | NO |
| ❖ Did patients receive same reference standard? | YES |
| ❖ Were all patients included in analysis? | NO |

**Could the patient flow have introduced bias?: RISK: HIGH**

**Justification:** This domain has HIGH risk of bias due to severe PARTIAL VERIFICATION BIAS. Only 12% of individuals who received the index test also received the reference standard (5,740/47,817), with the decision to test being directly contingent on index test results and clinical findings. The 40,486 CAD-negative/symptom-negative individuals (84.7% of CXR recipients) were systematically excluded from mWRD testing, meaning the true false-negative rate of AI-CAD cannot be determined. The authors explicitly acknowledge this limitation, stating estimates are 'pseudo-sensitivity' and 'pseudo-specificity' based on 'partial testing,' and noting that 'diagnosing more CAD-positive than CAD-negative would lead to an overestimation of sensitivity and an underestimation of specificity.' The CAD-negative individuals who were tested (n=1,432) represent only a highly selected subset with symptoms or radiologist concerns, yielding 2% positivity versus 17% among CAD-positive individuals.

# OVERALL ASSESSMENT SUMMARY

**HIGH RISK OF BIAS - SEVERE PARTIAL VERIFICATION**

| Domain | Signaling Questions | Risk of Bias | Applicability |
| --- | --- | --- | --- |
| D1: Patient Selection | 1.1 Unclear 1.2 Yes 1.3 No | HIGH | LOW |
| D2: Index Test | 2.1 Yes 2.2 Yes | LOW | LOW |
| D3: Reference Standard | 3.1 Yes 3.2 No | HIGH | LOW |
| D4: Flow and Timing | 4.1 Yes 4.2 No 4.3 Yes 4.4 No | HIGH | N/A |

**Overall Risk of Bias: HIGH**

**Justification:** This study has HIGH overall risk of bias, with 3 of 4 domains rated as HIGH risk. The primary methodological flaw is severe PARTIAL VERIFICATION BIAS: only 12% of individuals who received the index test (CXR with AI-CAD) also received the reference standard (mWRD), with testing contingent on index test results and clinical findings. Specifically, 40,486/47,817 (84.7%) CAD-negative/symptom-negative individuals were not tested per programmatic algorithm, making it impossible to accurately estimate true diagnostic accuracy metrics. The authors appropriately acknowledge these limitations using 'pseudo-sensitivity' and 'pseudo-specificity' terminology and explicitly stating that sensitivity is likely overestimated and specificity underestimated due to verification bias. The analytical sample is severely non-representative, consisting of 75% CAD-positive individuals plus 25% symptomatic/radiologist-flagged CAD-negative individuals.

**Overall Applicability Concern: LOW**

**Justification:** Despite methodological limitations, the study has excellent applicability to real-world point-of-care TB screening. The population (active case-finding in communities, workplaces, and hospitals in high TB-burden Philippines), setting (programmatic implementation with digital CXR), and index test (commercially available WHO-evaluated qXR AI-CAD software) all match the review question well. The study represents realistic operational implementation in resource-limited settings where AI-CAD would be deployed.

## Critical Biases Identified

**1. PARTIAL VERIFICATION BIAS (SEVERITY: SEVERE):**Only 5,740/47,817 (12%) who received CXR also received reference standard mWRD. Decision to test directly contingent on index test results: 87% of CAD-positives tested vs only 3.4% of CAD-negatives. 40,486 CAD-negative/symptom-negative individuals (84.7% of CXR recipients) systematically excluded from mWRD per programmatic algorithm. True false-negative rate cannot be determined. Authors acknowledge estimates are "pseudo-sensitivity" and "pseudo-specificity" based on "partial testing."

**2. INCORPORATION BIAS (SEVERITY: SEVERE):**Reference standard application directly dependent on index test results. TB-presumptive criteria included CAD-positive (≥0.50), creating circularity where index test determines whether reference standard is applied.

**3. SELECTION BIAS (SEVERITY: SEVERE):**Analytical sample severely non-representative: 75% CAD-positive (vs 10.3% in screened population). CAD-negative tested (25%) are symptomatic/radiologist-flagged subset, not representative of all CAD-negatives. Sample enriched with disease (13.4% TB prevalence in analytical sample vs likely much lower in untested CAD-negatives).

**AUTHORS EXPLICITLY ACKNOWLEDGE:**• "Pseudo-sensitivity" and "pseudo-specificity" based on partial testing
• "Diagnosing more CAD-positive than CAD-negative would lead to an overestimation of sensitivity and an underestimation of specificity" (p.12)
• "Testing more CAD-negative individuals might influence the overall metrics and provide a more precise overview of the actual performance of the AI-CAD model" (p.12)

## Methodological Strengths

**Despite severe verification bias, study has important strengths:

• Pre-specified threshold:** 0.50 cut-off set in 2020 pilot, not optimized on 2021-2024 test data
**• Appropriate transparency:** Authors explicitly acknowledge limitations using "pseudo" terminology
**• Large scale:** 47,817 CXR performed in programmatic implementation
**• Real-world data:** Operational performance in actual TB program workflows
**• Multiple pathways:** Community, workplace, and hospital screening contexts
**• Appropriate methods:** Precision-recall curves for imbalanced data, sensitivity analyses

## Reported Performance (PSEUDO-METRICS - BIASED)

**WARNING: These estimates are UNRELIABLE due to verification bias**Based on 5,740 individuals with both CXR and mWRD (12% of CXR recipients):

**Pseudo-Sensitivity:** 95.6% (734/767 TB-positive detected)
 → LIKELY OVERESTIMATED (40,486 untested CAD-negatives may contain missed cases)

**Pseudo-Specificity:** 28.1% (1,399/4,973 TB-negative)
 → LIKELY UNDERESTIMATED (CAD-positive group enriched with disease)

**AUROC:** 0.82

**Sample Composition (Biased):**• TB prevalence in analytical sample: 13.4% (767/5,740)
• CAD-positive proportion: 75% (vs 10.3% in screened population)
• CAD-negative tested: Only symptomatic/radiologist-flagged (3.4% of all CAD-negatives)

**Positivity Rates:**• Among CAD-positive tested: 17% (734/4,306)
• Among CAD-negative tested: 2% (33/1,432)
• Among untested CAD-negatives: UNKNOWN (true false-negative rate undetectable)

**CONFIDENCE IN RESULTS:** Low to very low - severe partial verification bias and non-representative sample make these performance estimates unreliable for clinical decision-making

## Study Value Despite Limitations

**What this study CAN inform:

✓ Feasibility:** AI-CAD can be successfully integrated into programmatic TB screening
**✓ Workflow:** Digital CXR with automated AI processing viable in operational settings
**✓ Scale:** 47,817 CXR processed over 3 years demonstrates scalability
**✓ Context:** Real-world performance data from high-burden setting (Philippines)
**✓ Referral patterns:** 10.3% CAD-positive referral rate from community screening

**What this study CANNOT inform:**✗ True diagnostic accuracy (sensitivity/specificity)
✗ False-negative rate among CAD-negative individuals
✗ Performance in unselected screening populations
✗ Cost-effectiveness (due to biased performance estimates)
✗ Optimal threshold selection (retrospective analysis in Table 3 also subject to bias)

QUADAS-2

**Nath et al. 2024**A multicentre study to evaluate the diagnostic performance of a novel CAD software, DecXpert, for radiological diagnosis of tuberculosis in the northern Indian population *Study ID: Nath_2024 | Assessment Date: 2025-11-28*

____________________________________________________________________________________________________

# Phase 1: State the Review Question

*Patients (setting, intended use of index test, presentation, prior testing):*

| Symptomatic individuals presenting to 12 primary health care (PHC) centers and 1 tertiary care (TC) center in northern India. Setting: Point-of-care - primary health care centers in high TB-burden region. Enrollment: January 2018-January 2022 (PHC centers), April 2022-November 2023 (TC center), prospective passive case-finding. Intended use: CAD-based TB screening for clinical decision support to identify TB requiring confirmatory testing. Presentation: Patients with fever, cough, expectoration, or constitutional symptoms for >2 weeks. Prior testing: None specified. Initial screening: 4,495 individuals. Exclusions (132 = 2.9%): 81 unproductive cough without BAL, 29 declined participation, 17 pregnant, 4 prior TB treatment, 1 inconclusive results. FINAL ANALYSIS: 4,363 patients. Population: Median age 43.1 years, 49.6% male, 50.4% female. Disease prevalence: 2,345/4,363 TB-positive (53.7%), 2,018/4,363 TB-negative (46.3%). High-burden setting without HIV co-infection. NOTE: Enrollment method (consecutive vs convenience) NOT explicitly stated despite prospective design. Multi-site 6-year enrollment introduces potential selection variability. |
| --- |

*Index test(s):*

| DecXpert version 1.4 - computer-aided detection (CAD) software based on deep convolutional neural networks with self-attention mechanisms. Training: 9,876 separate CXR images (4,932 TB, 4,944 non-TB) NOT included in validation cohort. Algorithm: Generates cumulative abnormality score 0-100. Classification threshold: Score ≥50 = TB positive; score <50 = TB negative. Image processing: CXRs resized to 224×224 pixels. Development constraints: Sensitivity >80%, specificity >75%, ≤2 million parameters. Automated CAD system processes images computationally without access to reference standard results. CRITICAL LIMITATION: Threshold of 50 was used but paper does NOT clearly state whether this was PRE-SPECIFIED before validation study or derived from this data. If threshold optimized on validation data, reported performance would be inflated. Lack of pre-specification documentation creates uncertainty about threshold optimization bias. Applicability strengths: Compatible with 7 CXR vendors, 500MB-16GB RAM, multiple Windows versions, all 5 CXR perspectives (PA/AP/lateral/decubitus/oblique). Real-world deployment tested at 6 remote northern India locations with on-site technician training. |
| --- |

*Reference standard and target condition:*

| GeneXpert MTB/RIF molecular testing - WHO-endorsed nucleic acid amplification test for Mycobacterium tuberculosis detection. UNIFORM APPLICATION: All 4,363 included patients received GeneXpert MTB/RIF on sputum or bronchoalveolar lavage (BAL) samples. NO differential verification bias - same reference standard for all patients. Results: 2,345 TB-positive, 2,018 TB-negative. Study explicitly used GeneXpert as 'gold standard molecular reference technique.' Automated molecular test detects MTB DNA/RNA computationally - results generated independently of DecXpert scores or CXR findings, eliminating incorporation bias. Authors acknowledge GeneXpert 'not perfect diagnostic tool' - false-negatives possible in extrapulmonary TB, drug-resistant TB, or low bacterial load cases. However, for pulmonary TB detection in symptomatic patients with productive cough/BAL (target population), GeneXpert has high sensitivity (95%+) and specificity (98%+). Appropriate reference standard for target condition. COMPARATOR (not reference): Three board-certified radiologists (3, 5, 6+ years experience) independently interpreted CXRs with 75% majority vote required for classification. Target condition: Active pulmonary tuberculosis requiring treatment. Timing between CXR and GeneXpert: NOT stated (unclear risk for interval bias). |
| --- |

# Phase 2: Draw a Flow Diagram for the Primary Study

| PATIENT FLOW - PROSPECTIVE MULTICENTER STUDY  STUDY PERIOD: ~6 years total • PHC centers (12 sites): January 2018 - January 2022 • TC center (1 site): April 2022 - November 2023  SETTING: Northern India (high TB burden) • 12 primary health care (PHC) centers • 1 tertiary care (TC) center  ↓ SCREENED: 4,495 individuals Inclusion: Fever, cough, expectoration, or constitutional symptoms >2 weeks  ↓ EXCLUSIONS: 132 (2.9%) • 81: Unproductive cough without available BAL sample • 29: Declined participation • 17: Pregnant females • 4: History of previous TB treatment • 1: Inconclusive CXR or GeneXpert results  ↓ ENROLLED: 4,363 individuals (97.1%)  ↓ ═══════════════════════════════════════════════════════════════════ ALL PATIENTS RECEIVED BOTH INDEX TEST AND REFERENCE STANDARD ═══════════════════════════════════════════════════════════════════  INDEX TEST: DecXpert v1.4 CAD Software All 4,363 received chest X-ray analyzed by DecXpert • Abnormality score: 0-100 • Threshold: ≥50 (TB positive), <50 (TB negative) • Automated computational processing • No access to reference standard during interpretation  REFERENCE STANDARD: GeneXpert MTB/RIF All 4,363 received GeneXpert molecular testing • Sample: Sputum or BAL • WHO-endorsed nucleic acid amplification test • Automated molecular detection of MTB DNA/RNA • Independent of DecXpert/CXR findings  CRITICAL STRENGTH: UNIFORM VERIFICATION • 100% of enrolled patients received reference standard • NO differential verification bias • NO partial verification bias • Same reference standard (GeneXpert) for all patients  ═══════════════════════════════════════════════════════════════════ RESULTS (n=4,363) ═══════════════════════════════════════════════════════════════════  REFERENCE STANDARD (GeneXpert): • TB-positive: 2,345 (53.7%) • TB-negative: 2,018 (46.3%)  2×2 TABLE:  GeneXpert+ GeneXpert- Total DecXpert+ (≥50) 2,095 214 2,309 DecXpert- (<50) 250 1,804 2,054 Total 2,345 2,018 4,363  PERFORMANCE (at threshold 50): • Sensitivity: 89.3% (2,095/2,345) • Specificity: 89.4% (1,804/2,018) • PPV: 90.7% (2,095/2,309) • NPV: 87.8% (1,804/2,054) • Accuracy: 89.4% (3,899/4,363) • AUC: 0.95 (95% CI: 0.94-0.96)  COMPARATOR: Radiologist Interpretation Three board-certified radiologists (3, 5, 6+ years experience) Independent interpretation with 75% majority vote • Sensitivity: 85.5% (2,005/2,345) • Specificity: 78.6% (1,586/2,018)  DecXpert vs Radiologists: • DecXpert: Sens 89.3%, Spec 89.4% • Radiologists: Sens 85.5%, Spec 78.6% • DecXpert outperformed human readers  ═══════════════════════════════════════════════════════════════════ METHODOLOGICAL QUALITY ISSUES ═══════════════════════════════════════════════════════════════════  UNCLEAR ISSUES (Documentation gaps, not evident flaws):  1. ENROLLMENT METHOD (Domain 1):  • "Prospectively enrolled" stated but NOT consecutive/random  • 6-year multi-site enrollment → potential selection variability  • Passive case-finding (symptom-driven presentation)  2. THRESHOLD PRE-SPECIFICATION (Domain 2):  • Threshold of 50 used throughout  • NOT clearly stated if pre-specified before validation  • If optimized on this data → inflated performance  • Algorithm development constraints mentioned (sens >80%, spec >75%)  • But specific threshold=50 justification absent  3. TIMING BETWEEN TESTS (Domain 4):  • CXR-to-GeneXpert interval NOT stated  • Unlikely to be major issue (both typically same visit)  • But documentation gap prevents certainty  METHODOLOGICAL STRENGTHS:  ✓ Large sample: N=4,363 (largest CAD TB validation to date) ✓ Prospective multicenter design (13 sites) ✓ Uniform reference standard (100% received GeneXpert) ✓ NO differential verification bias ✓ Minimal exclusions (2.9%) ✓ Transparent flow reporting (STARD-like diagram) ✓ Comparison with radiologists ✓ Real-world deployment testing ✓ Compatible with diverse hardware/software ✓ Cost-effectiveness analysis included ✓ Explainability visualization (Fig.5) - shows model attends to   clinically relevant lung regions, not artifacts/text  LIMITATIONS:  ✗ Training/validation split not externally validated ✗ No external validation cohort (different population) ✗ Single population (northern India, no HIV) ✗ Threshold pre-specification not documented ✗ Timing between tests not stated ✗ Enrollment method unclear |
| --- |

# Phase 3: Risk of Bias and Applicability Judgments

# DOMAIN 1: PATIENT SELECTION

## A. Risk of Bias

**Describe methods of patient selection:**Prospective passive case-finding multicenter study across 12 PHC centers and 1 TC center in northern India. Enrollment: Jan 2018-Jan 2022 (PHC), Apr 2022-Nov 2023 (TC). Total 4,495 screened, 132 excluded (2.9%), final 4,363 analyzed. Inclusion: fever, cough, expectoration, or constitutional symptoms >2 weeks. Exclusions: unproductive cough without BAL (81), declined (29), pregnant (17), prior TB treatment (4), inconclusive (1). Prospective cohort design - all symptomatic patients evaluated with both CXR and GeneXpert. NOT case-control. LIMITATION: Enrollment described as 'prospectively enrolled' but NOT explicitly consecutive or random. Six-year multi-site enrollment introduces potential selection variability. Page reference: p.2, p.11

| Signaling Question | Answer |
| --- | --- |
| ❖ Was a consecutive or random sample enrolled? | UNCLEAR |
| ❖ Was a case-control design avoided? | YES |
| ❖ Did study avoid inappropriate exclusions? | YES |

**Could the selection of patients have introduced bias?: RISK: UNCLEAR**

**Justification:** While the study design was prospective cohort (avoiding case-control bias) and exclusions were appropriate (only 2.9% excluded), the enrollment method is not clearly described as consecutive or random. The study was conducted over nearly 6 years across 13 different sites with staggered enrollment periods, which introduces potential for selection variability. The term passive case-finding suggests patients presented with symptoms rather than active screening, but this does not confirm consecutive enrollment. Without explicit confirmation of consecutive or random sampling, selection bias cannot be ruled out.

## B. Concerns regarding applicability

**Is there concern that the included patients do not match the review question?: CONCERN: LOW**

**Justification:** The population is highly applicable to POC imaging for TB clinical decision support. Patients were symptomatic individuals presenting to primary health care centers and one tertiary center in a high TB-burden region (northern India) without HIV co-infection. The median age was 43.1 years with balanced gender distribution (49.6% male, 50.4% female). This represents the intended use population for CAD-based TB screening in resource-limited, high-burden settings. The prevalence of TB in the cohort (2,345/4,363 = 53.7%) reflects a symptomatic population consistent with POC screening scenarios.

# DOMAIN 2: INDEX TEST

## A. Risk of Bias

**Describe the index test:**DecXpert version 1.4 - CAD software based on deep CNNs with self-attention mechanisms. Trained on 9,876 separate CXR images (4,932 TB, 4,944 non-TB) not included in validation cohort. Algorithm generates abnormality score 0-100, threshold 50 for TB classification. Automated CAD processes images computationally without access to reference standard. Development constraints: sensitivity >80%, specificity >75%, ≤2 million parameters. CRITICAL: Threshold of 50 used but NOT explicitly stated as pre-specified before validation study. If threshold optimized on this data, performance would be inflated. Lack of pre-specification documentation creates uncertainty about threshold optimization bias. Page reference: p.11-12

| Signaling Question | Answer |
| --- | --- |
| ❖ Interpreted without knowledge of reference? | YES |
| ❖ If threshold used, was it pre-specified? | UNCLEAR |

**Could the conduct of the index test have introduced bias?: RISK: UNCLEAR**

**Justification:** While the AI system inherently interpreted images without knowledge of reference standard results (addressing question 2.1), the threshold specification is problematic. The paper uses a threshold of 50 on a 0-100 scale but does not explicitly state this was pre-specified before the validation study. The algorithm was developed with performance constraints but the specific operating threshold justification is absent. If the threshold was optimized on validation data, this would inflate reported performance metrics. The lack of clear pre-specification documentation creates uncertainty about potential threshold optimization bias.

## B. Concerns regarding applicability

**Is there concern about the index test applicability?: CONCERN: LOW**

**Justification:** DecXpert was designed specifically for TB screening in resource-limited settings and was evaluated in its intended deployment context. The software demonstrated compatibility with 7 CXR vendors, various computational hardware (500MB to 16GB RAM), multiple Windows versions, and all 5 CXR perspectives (PA, AP, lateral, decubitus, oblique). The workflow integration assessment at 6 remote locations in northern India with on-site technician training confirms applicability to POC implementation. The AI tool was evaluated in the clinical context for which it was developed, supporting low applicability concern.

# DOMAIN 3: REFERENCE STANDARD

## A. Risk of Bias

**Describe the reference standard:**GeneXpert MTB/RIF molecular testing - WHO-endorsed nucleic acid amplification test for M. tuberculosis. ALL 4,363 patients received GeneXpert on sputum/BAL samples. Results: 2,345 TB-positive, 2,018 TB-negative. Study used GeneXpert as 'gold standard molecular reference technique.' Automated molecular test detects MTB DNA/RNA computationally - results independent of DecXpert scores/CXR findings, eliminating incorporation bias. Authors acknowledge GeneXpert limitations (false-negatives in extrapulmonary TB, drug-resistant TB, low bacterial load), but for pulmonary TB in symptomatic patients with productive cough/BAL, GeneXpert has high sensitivity (95%+) and specificity (98%+). Appropriate reference standard. NO differential verification - same test for all patients. Page reference: p.2, p.11, p.13

| Signaling Question | Answer |
| --- | --- |
| ❖ Is reference standard likely to correctly classify? | YES |
| ❖ Interpreted without knowledge of index test? | YES |

**Could the reference standard have introduced bias?: RISK: LOW**

**Justification:** GeneXpert MTB/RIF is an established, WHO-endorsed molecular diagnostic that provides objective, automated results for pulmonary TB detection. All 4,363 included patients received the same reference standard (GeneXpert), avoiding differential verification bias. The molecular test results are independent of both DecXpert and radiologist interpretations, eliminating incorporation bias. While GeneXpert has known limitations for certain TB presentations, these limitations are explicitly acknowledged and do not substantially bias results for pulmonary TB in symptomatic patients, which was the target population.

## B. Concerns regarding applicability

**Is there concern about the reference standard applicability?: CONCERN: LOW**

**Justification:** GeneXpert MTB/RIF is the WHO-endorsed molecular reference standard for TB diagnosis and is appropriate for the target condition (active pulmonary TB). The test is widely used in clinical practice and research. For the symptomatic population with productive cough or BAL samples evaluated in this study, GeneXpert provides highly accurate microbiological confirmation of TB.

# DOMAIN 4: FLOW AND TIMING

## A. Risk of Bias

**Patient flow:**STRENGTH - UNIFORM VERIFICATION: All 4,363 enrolled patients received BOTH index test (DecXpert CXR analysis) AND reference standard (GeneXpert MTB/RIF). NO differential verification bias - same reference standard for all patients. NO partial verification bias - 100% received reference standard. Minimal exclusions (132/4,495 = 2.9%) with appropriate reasons. All 4,363 included in 2×2 analysis. LIMITATION: Timing between CXR and GeneXpert testing NOT stated. Interval between tests unclear - could introduce disease progression bias if prolonged, though unlikely given both typically performed same clinical visit. Page reference: p.2, Fig.1

| Signaling Question | Answer |
| --- | --- |
| ❖ Appropriate interval between tests? | UNCLEAR |
| ❖ Did all patients receive reference standard? | YES |
| ❖ Did patients receive same reference standard? | YES |
| ❖ Were all patients included in analysis? | YES |

**Could the patient flow have introduced bias?: RISK: UNCLEAR**

**Justification:** This domain demonstrates significant methodological strengths but one documentation gap. STRENGTHS: All 4,363 patients received the same reference standard (GeneXpert), avoiding differential and partial verification bias - this is a critical strength. Minimal exclusions (2.9%) were appropriate and well-documented. All enrolled patients were included in the 2×2 analysis. LIMITATION: The time interval between CXR and GeneXpert testing is not stated in the paper. While both tests are typically performed during the same clinical visit in TB screening workflows, making prolonged intervals unlikely, the lack of explicit documentation prevents certainty about potential interval bias.

# OVERALL ASSESSMENT SUMMARY

**MODERATE RISK OF BIAS - DOCUMENTATION GAPS**

| Domain | Signaling Questions | Risk of Bias | Applicability |
| --- | --- | --- | --- |
| D1: Patient Selection | 1.1 Unclear 1.2 Yes 1.3 Yes | UNCLEAR | LOW |
| D2: Index Test | 2.1 Yes 2.2 Unclear | UNCLEAR | LOW |
| D3: Reference Standard | 3.1 Yes 3.2 Yes | LOW | LOW |
| D4: Flow and Timing | 4.1 Unclear 4.2 Yes 4.3 Yes 4.4 Yes | UNCLEAR | N/A |

**Overall Risk of Bias: MODERATE**

**Justification:** The study has MODERATE overall risk of bias with 3 domains rated UNCLEAR (driven by insufficient documentation rather than evident bias) and 1 domain rated LOW. UNCLEAR ratings: (1) Patient Selection - prospective enrollment stated but consecutive/random sampling not confirmed, 6-year multi-site enrollment introduces potential selection variability; (2) Index Test - threshold of 50 used but pre-specification before validation not documented, if optimized on this data performance estimates would be inflated; (3) Flow and Timing - all patients received same reference standard (excellent), but CXR-to-GeneXpert interval not stated. LOW rating: Reference Standard - all 4,363 patients received same WHO-endorsed molecular test with automated objective interpretation. Notable strengths: largest CAD TB validation to date (N=4,363), prospective multicenter design, uniform reference standard application (100%), minimal exclusions (2.9%), transparent flow reporting. The UNCLEAR ratings stem from reporting gaps rather than evident methodological flaws.

**Overall Applicability Concern: LOW**

**Justification:** All applicable domains demonstrate low applicability concerns. The study evaluated a CAD system designed for TB screening in resource-limited, high-burden settings among symptomatic patients presenting to primary health care centers in northern India. The AI tool was assessed using its intended deployment workflow across diverse hardware and software configurations with real-world implementation testing. The reference standard (GeneXpert MTB/RIF) provides microbiologically-confirmed TB diagnosis appropriate for the review scope. The population, intervention, and reference standard align well with POC imaging for clinical decision support.

## Key Methodological Strengths

**1. LARGEST CAD TB VALIDATION TO DATE:**N=4,363 patients - substantially larger than most CAD studies. Provides narrow confidence intervals and robust performance estimates.

**2. UNIFORM REFERENCE STANDARD (CRITICAL STRENGTH):**100% of enrolled patients received GeneXpert MTB/RIF. NO differential verification bias. NO partial verification bias. Same test for all patients.

**3. PROSPECTIVE MULTICENTER DESIGN:**13 sites across northern India (12 PHC, 1 TC). Real-world heterogeneity in patient populations and CXR equipment.

**4. MINIMAL EXCLUSIONS:**Only 132/4,495 (2.9%) excluded with appropriate clinical reasons. Transparent STARD-like flow diagram.

**5. COMPARISON WITH RADIOLOGISTS:**Three board-certified radiologists (3, 5, 6+ years) independently interpreted CXRs. DecXpert outperformed (Sens 89.3% vs 85.5%, Spec 89.4% vs 78.6%).

**6. REAL-WORLD DEPLOYMENT TESTING:**Evaluated across 7 CXR vendors, 500MB-16GB RAM, multiple Windows versions, all 5 CXR perspectives. On-site technician training at 6 remote locations.

## Key Limitations (Documentation Gaps)

**1. Enrollment method unclear** (consecutive vs convenience not stated)
**2. Threshold pre-specification not documented** (potential optimization bias)
**3. Timing between tests not stated** (CXR-to-GeneXpert interval)
**4. No external validation** (different population/geography)
**5. Single population** (northern India, no HIV co-infection)

## Reported Performance (Reliable)

**Sample: 4,363 patients (2,345 TB+, 2,018 TB-)**TB Prevalence: 53.7%

**DecXpert Performance (at threshold 50):**• Sensitivity: 89.3% (2,095/2,345)
• Specificity: 89.4% (1,804/2,018)
• PPV: 90.7%
• NPV: 87.8%
• Accuracy: 89.4%
• AUC: 0.95 (95% CI: 0.94-0.96)

**Radiologist Performance (75% majority vote):**• Sensitivity: 85.5%
• Specificity: 78.6%

**INTERPRETATION:**Estimates likely reliable due to uniform reference standard and large sample. UNCLEAR ratings reflect documentation gaps, not evident bias. Threshold optimization concern exists but cannot be confirmed. DecXpert demonstrated non-inferior to superior performance vs radiologists.

QUADAS-2

**Nothnagel & Aslam 2024**Evaluating the benefits of machine learning for diagnosing deep vein thrombosis compared with gold standard ultrasound: a feasibility study *Study ID: Nothnagel_2024 | Assessment Date: 2025-11-28*

____________________________________________________________________________________________________

# Phase 1: State the Review Question

*Patients (setting, intended use of index test, presentation, prior testing):*

| Patients with suspected DVT requiring ultrasound scan, recruited consecutively at a hospital in Berlin, Germany, over 3.5-month period in 2022. Total enrolled: 91 participants. Setting: Point-of-care - hospital setting with mixed patient sources: 46% inpatients, 2% directly scheduled, 52% referred from primary care sector. Intended use: AI-guided POCUS for DVT diagnosis by non-specialist operators with minimal training. Presentation: Symptoms suggestive of DVT with clinical indication for diagnostic ultrasound per DVT diagnostic algorithm. Clinical context: 59% referred with genuine DVT suspicion; 41% underwent scans as exclusion diagnostics before treating other conditions (cellulitis, musculoskeletal issues). Inclusion criteria: Age ≥18 years, capacity to consent, symptoms suggestive of DVT, DVT algorithm indicating need for diagnostic ultrasound. Power calculation: Minimum 7 positive DVT cases needed for 80% power. Population demographics: Mean BMI 26 (range 15.8-47.3). STRENGTH: Consecutive enrollment over defined period. LIMITATION: Single-center, single non-specialist operator. |
| --- |

*Index test(s):*

| AI-guided POCUS using ThinkSono Guidance app (ThinkSono GmbH, Class 1 CE certification), installed on smartphone connected to Clarius L7 HD3 linear handheld ultrasound probe. Operator: Single non-specialist without formal DVT diagnostic scan experience, following 1-hour training session. Protocol: App guides users through two-region POCUS directing compressions in groin areas and knee pit. AI technology: Convolutional neural network (U-Net architecture) for vessel analysis with auxiliary branches for anatomical location prediction. Processing: B-mode ultrasound images resampled to 128×128 pixels, producing segmentation masks and categorical location labels within 25 milliseconds. Recording: Four 10-second compression sequences recorded per scan, uploaded to cloud dashboard. Interpretation: Five remote specialists (blinded to patient attributes, clinical indications, and reference results) assessed image quality using ACEP score (≥3 deemed sufficient) and provided diagnoses: compressible, incompressible, or indeterminate. STRENGTHS: Remote specialists blinded, previously validated system with pre-specified thresholds, CE-certified. LIMITATIONS: 18% protocol deviations/technical errors preventing upload, 9% inadequate image quality, 15% indeterminate results - substantial exclusions. |
| --- |

*Reference standard and target condition:*

| Formal DVT diagnostic duplex scan recommended by NICE guidelines. Protocol: Compressions at multiple points on proximal leg every few centimeters from groin to knee pit along large veins, coupled with Doppler ultrasound to demonstrate normal blood movement. Interpretation: Qualified physician blinded to index scan outcomes provided report after reference scan completion, indicating presence or absence of DVT. STRENGTH: All 91 enrolled patients underwent both index test (AI-guided POCUS) and reference standard (formal duplex) - NO differential verification, NO partial verification. Appropriate gold standard per NICE guidelines. Blinded interpretation eliminates review bias. LIMITATION: Time interval between index and reference tests not explicitly stated, though both performed during same clinical encounter suggesting same-day testing. Target condition: Deep vein thrombosis (DVT) in lower extremity. |
| --- |

# Phase 2: Draw a Flow Diagram for the Primary Study

| PATIENT FLOW DIAGRAM  STUDY PERIOD: 3.5 months in 2022 SETTING: Hospital in Berlin, Germany DESIGN: Prospective consecutive enrollment  ↓ ELIGIBLE: Patients with suspected DVT requiring ultrasound scan • Inclusion: Age ≥18 years, capacity to consent, DVT symptoms,  clinical algorithm indicating need for diagnostic ultrasound • Power calculation: Minimum 7 positive DVT cases for 80% power  ↓ ENROLLED: 91 patients (consecutive enrollment)  PATIENT SOURCES: ├─→ Inpatients: 42 (46%) ├─→ Direct scheduled: 2 (2%) └─→ Primary care referrals: 47 (52%)  CLINICAL INDICATIONS: ├─→ Genuine DVT suspicion: 54 (59%) └─→ Exclusion diagnostics*: 37 (41%)  *Before treating cellulitis or musculoskeletal issues  POPULATION: Mean BMI 26 (range 15.8-47.3)  ↓ ═══════════════════════════════════════════════════════════════════════ ALL 91 PATIENTS RECEIVED BOTH TESTS ═══════════════════════════════════════════════════════════════════════  INDEX TEST: AI-Guided POCUS (ThinkSono Guidance) Equipment: Clarius L7 HD3 linear handheld probe + smartphone app Operator: Single non-specialist (1-hour training, no prior DVT scan experience) AI: U-Net CNN for vessel analysis (25ms processing) Protocol: Two-region POCUS, four 10-second compression sequences Upload: Cloud dashboard for remote specialist review  REFERENCE STANDARD: NICE-Recommended Duplex Ultrasound Operator: Qualified physician Protocol: Comprehensive compressions (groin to knee pit) + Doppler Blinding: Physician blinded to index scan results Timing: Same clinical encounter (exact interval not stated)  ✓ NO VERIFICATION BIAS: All 91 received both tests ✓ NO DIFFERENTIAL VERIFICATION: Same reference for all  ↓ ═══════════════════════════════════════════════════════════════════════ CRITICAL ISSUE: SUBSTANTIAL POST-TEST EXCLUSIONS ═══════════════════════════════════════════════════════════════════════  EXCLUSION CASCADE (INDEX TEST SIDE):  ENROLLED: 91 patients  ↓ EXCLUSION ROUND 1: Upload Issues (16/91 = 18%) ├─→ Protocol deviations: 13 └─→ Technical errors: 3  • Fragmented scans  • Scans not correctly transferred to cloud  ↓ UPLOADED SUCCESSFULLY: 75 (82% of enrolled)  ↓ EXCLUSION ROUND 2: Inadequate Image Quality (7/75 = 9%) • ACEP score <3 (insufficient quality for diagnosis)  ↓ ADEQUATE QUALITY: 68 (75% of enrolled, 91% of uploaded)  ↓ EXCLUSION ROUND 3: Indeterminate/Other (10/68 = 15%) ├─→ Other pathological findings (e.g., Baker's cyst) ├─→ Disagreement among remote reviewers └─→ Unable to provide compressibility assessment  ↓ FINAL DIAGNOSTIC ASSESSMENT: 58 (64% of enrolled)  TOTAL EXCLUDED: 33/91 (36% of enrolled) ├─→ Protocol/technical: 16 (18%) ├─→ Inadequate quality: 7 (8%) └─→ Indeterminate: 10 (11%)  ↓ ═══════════════════════════════════════════════════════════════════════ DIAGNOSTIC ACCURACY ANALYSIS (n=58) ═══════════════════════════════════════════════════════════════════════  INDEX TEST RESULTS (AI-POCUS): Remote specialists provided compressibility assessment  REFERENCE STANDARD RESULTS (Duplex): All 58 also had reference duplex results  2×2 TABLE (n=58): • True Positives (DVT+, detected): ~5 (9% of 58 ≈ 5.2) • False Positives (DVT-, flagged): ~5 (estimated from 91% specificity) • False Negatives (DVT+, missed): 0 • True Negatives (DVT-, cleared): ~48  PERFORMANCE (n=58 only): • Sensitivity: 100% (95% CI: 99.12-100%) • Specificity: 91% • Perfect sensitivity with ~5 DVT cases  ⚠️ CRITICAL INTERPRETATION ISSUE: Performance metrics apply ONLY to 64% successfully analyzed 36% excluded patients may have systematically different characteristics: • More difficult anatomy (BMI range 15.8-47.3) • Technical challenges (protocol deviations, upload failures) • Pathological complexity (other findings, indeterminate results)  ═══════════════════════════════════════════════════════════════════════ RISK STRATIFICATION APPROACH (Study Acknowledges Limitation) ═══════════════════════════════════════════════════════════════════════  Study categorizes patients into risk groups:  LOW RISK (48/91 = 53%): • Adequate quality scans with clear compressibility assessment • These contribute to diagnostic accuracy metrics  HIGH RISK (43/91 = 47%): • All excluded cases (33 from exclusion cascade) • Plus 10 from other reasons • Require further investigation/referral • Cannot contribute to accuracy assessment  This acknowledges the limitation but doesn't eliminate the bias Real-world "intention-to-diagnose" performance likely lower  ↓ ═══════════════════════════════════════════════════════════════════════ EXCLUSION BIAS IMPACT ═══════════════════════════════════════════════════════════════════════  POTENTIAL SPECTRUM BIAS: Excluded patients may represent: • More challenging anatomy (higher BMI, difficult compression) • More complex pathology (concurrent conditions) • Technical learning curve issues (single operator, early cases)  REPORTED METRICS (Sensitivity 100%, Specificity 91%): → Apply to "best case" 64% with successful scans → May overestimate real-world performance → "Intention-to-diagnose" analysis would include all 91  AUTHORS ACKNOWLEDGE: Study appropriately flags all excluded/indeterminate as "high risk" requiring further investigation, but this represents significant reduction in evaluable population introducing exclusion bias |
| --- |

# Phase 3: Risk of Bias and Applicability Judgments

# DOMAIN 1: PATIENT SELECTION

## A. Risk of Bias

**Describe methods of patient selection:**Patients with suspected DVT requiring ultrasound scan recruited consecutively at hospital in Berlin, Germany, over 3.5-month period in 2022. Total: 91 participants. Inclusion criteria: age ≥18 years, capacity to consent, symptoms suggestive of DVT, DVT algorithm indicating need for diagnostic ultrasound. Patient sources: 46% inpatients, 2% directly scheduled, 52% primary care referrals. Clinical context: 59% genuine DVT suspicion, 41% exclusion diagnostics before treating other conditions (cellulitis, musculoskeletal). Power calculation: minimum 7 positive DVT cases needed. Prospective cohort design. No inappropriate exclusions described. Page reference: p.2, p.4-5

| Signaling Question | Answer |
| --- | --- |
| ❖ Was a consecutive or random sample enrolled? | YES |
| ❖ Was a case-control design avoided? | YES |
| ❖ Did study avoid inappropriate exclusions? | YES |

**Could the selection of patients have introduced bias?: RISK: LOW**

**Justification:** The study employed consecutive enrollment over a defined 3.5-month period in 2022 at a hospital in Berlin, Germany. The inclusion criteria were clinically appropriate and designed to capture patients requiring DVT diagnostic workup per established clinical algorithms. The prospective cohort design avoided case-control sampling that could artificially inflate diagnostic accuracy. The patient population was representative of clinical practice, including a mix of inpatients (46%) and primary care referrals (52%), which enhances generalizability to real-world point-of-care settings.

## B. Concerns regarding applicability

**Is there concern that the included patients do not match the review question?: CONCERN: LOW**

**Justification:** The study population is appropriate for the review question on AI-based CDSS in point-of-care imaging. Patients presented with suspected DVT and were evaluated using AI-guided point-of-care ultrasound by non-specialists, which directly matches the intended POC workflow. The mix of inpatient and primary care referral patients represents the spectrum of patients who might benefit from POC DVT diagnosis in diverse clinical settings.

# DOMAIN 2: INDEX TEST(S)

## A. Risk of Bias

**Describe the index test:**AI-guided POCUS using ThinkSono Guidance app (ThinkSono GmbH, Class 1 CE certification) on smartphone connected to Clarius L7 HD3 linear handheld probe. Scans by single non-specialist without formal DVT scan experience, 1-hour training. App guides two-region POCUS with compressions in groin/knee pit. AI: U-Net CNN for vessel analysis, processes B-mode images (128×128 pixels) producing segmentation masks and location labels in 25ms. Four 10-second compression sequences recorded, uploaded to cloud. Five remote specialists (blinded to patient attributes, clinical indications, reference results) assessed image quality (ACEP ≥3) and provided diagnosis: compressible, incompressible, indeterminate. Thresholds pre-specified based on previously validated system. STRENGTHS: Blinded remote review, pre-specified thresholds, prior validation. Page reference: p.3-4

| Signaling Question | Answer |
| --- | --- |
| ❖ Interpreted without knowledge of reference? | YES |
| ❖ If threshold used, was it pre-specified? | YES |

**Could the conduct of the index test have introduced bias?: RISK: LOW**

**Justification:** Remote specialists interpreting the index test were explicitly blinded to patient attributes, clinical indications, and reference standard results, only knowing which extremity was examined. This eliminates review bias in the index test interpretation. The diagnostic thresholds for image quality (ACEP ≥3) and classification categories were pre-specified in the methodology and not optimized on the study data. The ThinkSono Guidance app was a previously validated system with documented prior validation studies, meaning thresholds were externally established rather than derived from this dataset.

## B. Concerns regarding applicability

**Is there concern about the index test applicability?: CONCERN: LOW**

**Justification:** The index test is directly applicable to the review question. ThinkSono Guidance is a CE-certified AI-guided POCUS application designed for point-of-care DVT diagnosis by non-specialists. The study used the app under its intended purpose by healthcare professionals with only 1-hour training, which reflects the realistic workflow for primary care or POC implementation. The handheld ultrasound probe (Clarius L7 HD3) is appropriate for POC settings.

# DOMAIN 3: REFERENCE STANDARD

## A. Risk of Bias

**Describe the reference standard:**Formal DVT diagnostic duplex scan per NICE guidelines. Protocol: Compressions at multiple points on proximal leg every few centimeters from groin to knee pit along large veins, coupled with Doppler ultrasound to demonstrate normal blood movement. Qualified physician blinded to index scan outcomes provided report after reference scan completion, indicating presence/absence of DVT. STRENGTH: All 91 enrolled patients underwent both index test (AI-guided POCUS) and reference standard (formal duplex) - NO differential verification, NO partial verification. Appropriate gold standard per NICE guidelines. Blinded interpretation eliminates review bias. LIMITATION: Time interval not explicitly stated, though both performed during same clinical encounter suggesting same-day testing. Page reference: p.3, p.5

| Signaling Question | Answer |
| --- | --- |
| ❖ Is reference standard likely to correctly classify? | YES |
| ❖ Interpreted without knowledge of index test? | YES |

**Could the reference standard have introduced bias?: RISK: LOW**

**Justification:** The reference standard (formal duplex ultrasound per NICE guidelines) is the accepted gold standard for DVT diagnosis and is likely to correctly classify disease status. The qualified physician performing the reference scan was explicitly blinded to the index scan outcomes, eliminating review bias. All 91 enrolled patients received the same reference standard regardless of index test results, avoiding differential verification bias. The comprehensive protocol with multiple compression points and Doppler assessment ensures thorough evaluation.

## B. Concerns regarding applicability

**Is there concern about reference standard applicability?: CONCERN: LOW**

**Justification:** The reference standard (NICE-recommended formal duplex ultrasound) is appropriate and applicable for DVT diagnosis. This is the established gold standard for confirming or excluding DVT in clinical practice. The comprehensive protocol with compression ultrasound and Doppler assessment matches standard diagnostic procedures.

# DOMAIN 4: FLOW AND TIMING

## A. Risk of Bias

**Patient flow:**All 91 enrolled patients underwent both index test (AI-guided POCUS) and reference standard (formal duplex). HOWEVER, substantial post-test exclusions occurred. Only 58/91 (64%) included in final diagnostic accuracy analysis. Exclusions: 16/91 (18%) protocol deviations or technical errors preventing upload (13 protocol deviations, 3 technical errors with fragmented scans or transfer failures); 7/75 (9%) inadequate image quality (ACEP <3); 10/68 (15%) indeterminate findings, other pathologies (e.g. Baker's cyst), or reviewer disagreement. Total excluded: 33/91 (36%). Creates exclusion bias as excluded cases may have systematically different characteristics (difficult anatomy, higher BMI, complex pathology). Time interval not explicitly stated but both tests performed during same clinical encounter. Page reference: p.2, p.5-6, Table 3, Figure 2

| Signaling Question | Answer |
| --- | --- |
| ❖ Appropriate interval between tests? | UNCLEAR |
| ❖ Did all patients receive reference standard? | YES |
| ❖ Did patients receive same reference standard? | YES |
| ❖ Were all patients included in analysis? | NO |

**Could the patient flow have introduced bias?: RISK: HIGH**

**Justification:** Substantial patient exclusion occurred, with only 58/91 (64%) patients included in the final diagnostic accuracy analysis. The excluded 33/91 (36%) patients were removed due to protocol deviations (N=13), technical errors (N=3), inadequate image quality (N=7), and indeterminate findings or reviewer disagreement (N=10). This creates exclusion bias because the excluded cases may have systematically different characteristics (e.g., more difficult anatomy, larger body habitus as mean BMI was 26 with maximum 47.3) that could affect diagnostic performance. The reported sensitivity (100%) and specificity (91%) apply only to the 64% of patients successfully analyzed, potentially overestimating performance in a real-world 'intention-to-diagnose' population. The study acknowledges this limitation by categorizing all excluded cases as 'high risk' requiring further investigation, but this represents a significant reduction in the evaluable population that introduces potential spectrum bias and exclusion bias.

# OVERALL ASSESSMENT SUMMARY

**MODERATE RISK OF BIAS - Substantial Exclusions**

| Domain | Signaling Questions | Risk of Bias | Applicability |
| --- | --- | --- | --- |
| D1: Patient Selection | 1.1 Yes 1.2 Yes 1.3 Yes | LOW | LOW |
| D2: Index Test | 2.1 Yes 2.2 Yes | LOW | LOW |
| D3: Reference Standard | 3.1 Yes 3.2 Yes | LOW | LOW |
| D4: Flow and Timing | 4.1 Unclear 4.2 Yes 4.3 Yes 4.4 No | HIGH | N/A |

**Overall Risk of Bias: MODERATE**

**Justification:** This study demonstrates generally sound methodology with important strengths: consecutive enrollment of 91 patients over 3.5 months, appropriate blinding of both index test interpreters and reference standard physician, use of an established gold standard reference (NICE-recommended duplex ultrasound), and application of the same reference standard to all enrolled patients. The study appropriately avoided verification bias by ensuring all patients received the gold standard reference regardless of index test findings. However, the primary methodological concern is substantial exclusion bias in Domain 4. Only 58/91 patients (64%) contributed to the final diagnostic accuracy analysis, with 36% excluded due to protocol deviations, technical errors, inadequate image quality, or indeterminate results. This exclusion may create spectrum bias if the excluded patients represent systematically more difficult cases. The study's perfect sensitivity (100%) and high specificity (91%) should be interpreted cautiously as they may not reflect performance in an 'intention-to-diagnose' population where all attempted scans must yield actionable results. Additional limitations include small sample size with only approximately 5-6 true positive DVT cases, which limits precision of sensitivity estimates, and the single-center, single-operator design which limits generalizability.

**Overall Applicability Concern: LOW**

**Justification:** The study population, index test, and reference standard all align well with the review question on AI-based clinical decision support systems in point-of-care imaging. The ThinkSono Guidance app represents a CE-certified AI-POCUS system specifically designed for non-specialist operators in primary care settings, directly matching the intended POC implementation context. The patient population included primary care referrals (52%) and the realistic 1-hour training protocol reflects achievable preparation for POC operators.

## Key Methodological Strengths

**✓ Consecutive enrollment:** All eligible patients over 3.5 months enrolled prospectively
**✓ No verification bias:** All 91 patients received both index test AND reference standard
**✓ No differential verification:** Same gold standard applied to all patients regardless of index results
**✓ Appropriate blinding:** Remote specialists blinded to patient info and reference results; reference physician blinded to index results
**✓ Pre-specified thresholds:** Previously validated system, not optimized on test data
**✓ Gold standard reference:** NICE-recommended formal duplex ultrasound
**✓ Real-world POC context:** Non-specialist operator, 1-hour training, handheld device, primary care referrals

## Critical Limitation: Exclusion Bias

**SUBSTANTIAL POST-TEST EXCLUSIONS (36% of enrolled patients):

Exclusion Cascade:**• Enrolled: 91 patients
• Protocol deviations/technical errors: -16 (18%)
• Inadequate image quality (ACEP <3): -7 (9% of uploaded)
• Indeterminate/other pathology/disagreement: -10 (15% of adequate quality)
• Final analysis: 58 (64%)

**Exclusion Types:
1.** Technical/Protocol Issues (16 patients): Fragmented scans, upload failures, protocol deviations
**2.** Quality Issues (7 patients): ACEP score <3, insufficient image quality for diagnosis
**3.** Indeterminate (10 patients): Other pathology (Baker's cyst), reviewer disagreement, unable to assess compressibility

**Potential Spectrum Bias:**Excluded patients may represent:
• More challenging anatomy (BMI range 15.8-47.3, mean 26)
• More difficult compressions (higher BMI, edema)
• Complex pathology (concurrent conditions)
• Technical learning curve (single operator, early cases)

**Impact on Results:**• Reported metrics (100% sens, 91% spec) apply only to successfully analyzed 64%
• May overestimate real-world "intention-to-diagnose" performance
• Study acknowledges by categorizing excluded cases as "high risk" requiring referral
• However, this represents significant reduction in evaluable population

## Reported Performance (n=58 only)

**Applies to 64% of enrolled patients with successful scans

Sensitivity:** 100% (95% CI: 99.12-100%)
• Based on ~5 DVT-positive cases (9% of 58)
• Zero false negatives in successfully analyzed scans
• Small sample limits precision

**Specificity:** 91%
• ~5 false positives among ~53 DVT-negative cases

**Risk Stratification Approach:**• LOW RISK (48/91 = 53%): Clear compressibility assessment, contribute to accuracy metrics
• HIGH RISK (43/91 = 47%): All excluded cases + indeterminate, require further investigation

**INTERPRETATION:** Perfect sensitivity impressive but based on small number of positives (~5 cases). High specificity (91%) reasonable. However, 36% exclusion rate means real-world "intention-to-diagnose" performance likely lower. Study appropriately acknowledges limitation.

## Study Value

**What this study demonstrates:

✓ POC feasibility:** AI-guided POCUS by non-specialist (1-hour training) is feasible
**✓ Methodological rigor:** No verification bias, appropriate blinding, gold standard reference
**✓ Real-world context:** Mix of inpatients and primary care referrals
**✓ CE-certified technology:** Previously validated system with pre-specified thresholds

**Limitations to consider:

✗ High exclusion rate:** 36% could not be analyzed (technical, quality, indeterminate)
**✗ Small positive sample:** Only ~5 DVT cases limits sensitivity precision
**✗ Single center/operator:** Limits generalizability
**✗ Performance in "best cases":** Metrics may not reflect all-comers intention-to-diagnose

QUADAS-2

**Papachristou et al. 2024**Evaluation of an artificial intelligence-based decision support for the detection of cutaneous melanoma in primary care: a prospective real-life clinical trial *Study ID: Papachristou_2024 | Assessment Date: 2025-11-28*

____________________________________________________________________________________________________

# Phase 1: State the Review Question

*Patients (setting, intended use of index test, presentation, prior testing):*

| Patients aged ≥18 years presenting with skin lesions for which primary care physician (PCP) had any suspicion of melanoma (ranging from 'appears benign but cannot fully exclude melanoma' to 'undoubtedly a melanoma'). Setting: Point-of-care - 36 primary care centres (PCCs) across seven regions in southern Sweden, May-December 2022. Intended use: AI-based clinical decision support (Dermalyser smartphone app) for melanoma detection to assist PCPs in triage/referral decisions. Presentation: Patients presenting to primary care with concerning skin lesions during routine clinical visits. Training: 138 PCPs (90 certified GPs, 48 resident trainees) trained on app use before study. Enrolled: 262 lesions assessed; Final: 253 lesions in 228 patients (125 women, 103 men, mean age 54 years). Exclusions: 2 declined, 3 inclusion errors, 4 lost to follow-up (9/262 = 3.4% excluded). Additional exclusions: Other cutaneous malignancies only (not target condition), damaged/tattooed skin, inaccessible locations, dense hair, Fitzpatrick V-VI skin types (algorithm not trained on these), poor image quality. Disease prevalence: 21 melanomas detected (8.3% of 253 lesions): 11 invasive, 10 in situ. All invasive melanomas thin (Breslow 0.1-1.1mm, 91% T1a). Population predominantly Fitzpatrick I-II. LIMITATION: Enrollment method not explicitly stated as consecutive; 7/36 centres contributed no lesions, participating centres 1-33 lesions each (suggests variable selection). |
| --- |

*Index test(s):*

| Dermalyser smartphone app (AI Medical Technology, Stockholm, Sweden) - AI-based clinical decision support tool for melanoma detection. Algorithm: Machine learning trained in silico prior to study on extensive dermoscopic images, achieving AUROC 0.94 in pretrial validation. FIXED algorithm with no further training or modification during study. Technical: Algorithm produces continuous value 0-1 (melanoma probability), presented to user as dichotomous outcome: 'evidence of melanoma detected' or 'no evidence of melanoma detected' based on pre-specified cutoff. Threshold: Pre-specified prior to data inclusion, selected from in silico ROC curve to achieve target sensitivity 95% and specificity 78%. Equipment: Standardized - camera-equipped smartphones (iPhone SE 2020, iOS 14/15) connected to polarized light contact dermoscopes (Heine iC1 or DermLite DL3) via phone case adapters. Protocol: PCPs photographed lesions dermoscopically. CRITICAL: PCPs recorded clinical assessment and management decision BEFORE applying app; app outcome NOT allowed to affect clinical management or be communicated to patients. STRENGTHS: Pre-specified threshold from pretrial validation (not optimized on study data), fixed algorithm, automated interpretation (no human review bias), blinded to reference standard (app used at presentation before diagnosis obtained). No remuneration to centres or patients. |
| --- |

*Reference standard and target condition:*

| Final clinical or histopathological tumour diagnosis from patient medical records. CRITICAL - DIFFERENTIAL VERIFICATION BIAS: Two different reference standards applied: (1) Histopathology: 134/253 lesions (53%) - lesions excised (at PCC or surgical clinic) with histopathological analysis. (2) Clinical diagnosis only: 119/253 lesions (47%) - referred to dermatologist for evaluation without excision. Decision criteria: Lesions deemed 'undoubtedly benign' by dermatologist were NOT excised per Swedish standard clinical practice. All 21 detected melanomas received histopathological confirmation. However, 119 lesions classified as benign based ONLY on dermatologist clinical assessment without histopathological verification. BIAS CONSEQUENCE: If any melanomas among 119 clinically-diagnosed lesions were missed by dermatologist, they would be misclassified as true negatives, artificially inflating app's specificity and NPV. Relatively short 8-month study period means delayed melanoma diagnoses would not be captured. Authors provide ethical justification for not excising all lesions. Target condition: Melanoma (malignant melanoma, including invasive melanoma and melanoma in situ). Follow-up: Medical records reviewed 2-9 months after index visit (mean 5 months, SD 1.5) to capture delayed diagnoses. |
| --- |

# Phase 2: Draw a Flow Diagram for the Primary Study

| PATIENT FLOW DIAGRAM - PROSPECTIVE MULTICENTRE STUDY  STUDY PERIOD: May - December 2022 (8 months) SETTING: 36 Primary Care Centres (PCCs), 7 regions, southern Sweden OPERATORS: 138 Primary Care Physicians ├─→ 90 Certified General Practitioners └─→ 48 Resident Trainees Training: On-site training on Dermalyser app before study initiation  ↓ ELIGIBLE: Patients aged ≥18 years presenting with skin lesions for which PCP had ANY suspicion of melanoma (from "appears benign but cannot fully exclude" to "undoubtedly melanoma")  EXCLUSIONS (Pre-enrollment): • Lesions exclusively suspected of other cutaneous malignancies (BCC, SCC) • Lesions on damaged or tattooed skin • Lesions on inaccessible body locations (e.g., between fingers) • Lesions covered with dense hair (prevents sufficient image quality) • Patients with Fitzpatrick skin types V-VI (lack of training data) • Poor-quality dermoscopic images  ↓ ASSESSED FOR MELANOMA SUSPICION: 262 lesions in 237 patients  PARTICIPATION: • 7/36 centres (19%) contributed NO lesions • 29/36 centres (81%) contributed 1-33 lesions each • Variable inclusion rates across centres (suggests selection variability)  ↓ POST-ENROLLMENT EXCLUSIONS: 9 lesions (3.4%) ├─→ Declined participation: 2 lesions (in 2 patients) ├─→ Inclusion errors: 3 lesions (in 3 patients) └─→ Lost to follow-up: 4 lesions (in 4 patients)  ↓ FINAL STUDY POPULATION: 253 lesions in 228 patients • 125 women (54.8%), 103 men (45.2%) • Mean age: 54 years • Predominantly Fitzpatrick I-II skin types • Disease prevalence: 21 melanomas (8.3%)  - 11 invasive melanomas (all thin: Breslow 0.1-1.1mm, 91% T1a)  - 10 melanomas in situ  ↓ ═══════════════════════════════════════════════════════════════════════ ALL 253 LESIONS RECEIVED INDEX TEST ═══════════════════════════════════════════════════════════════════════  INDEX TEST: Dermalyser AI App  Equipment: Standardized ├─→ Smartphone: iPhone SE 2020 (iOS 14/15) ├─→ Dermoscope: Heine iC1 or DermLite DL3 (polarized light contact) └─→ Connection: Phone case adapters  AI Algorithm: • Trained in silico prior to study (extensive dermoscopic images) • Pretrial validation: AUROC 0.94 • FIXED - no modification during study • Continuous output: 0-1 (melanoma probability) • Dichotomous presentation: "Evidence detected" vs "No evidence" • Pre-specified threshold: Target sens 95%, spec 78%  (determined from pretrial ROC curve BEFORE data collection)  CRITICAL BLINDING PROCEDURE: 1. PCP examined lesion clinically 2. PCP recorded assessment and management decision 3. PCP photographed lesion dermoscopically 4. Dermalyser app analyzed image (automatic) 5. App outcome NOT communicated to patient 6. App outcome NOT allowed to affect clinical management  Result: App interpretation completely independent of reference  ↓ ═══════════════════════════════════════════════════════════════════════ DIFFERENTIAL VERIFICATION BIAS (CRITICAL FLAW) ═══════════════════════════════════════════════════════════════════════  REFERENCE STANDARD PATHWAY 1: Histopathology (n=134, 53%)  Lesions EXCISED (at PCC or surgical clinic) → Histopathological analysis performed → Definitive diagnosis obtained  This group includes: • ALL 21 melanomas (100% confirmed histopathologically)  - 11 invasive melanomas  - 10 melanomas in situ • 113 benign lesions with histopathological confirmation  REFERENCE STANDARD PATHWAY 2: Clinical Diagnosis Only (n=119, 47%)  Lesions referred to DERMATOLOGIST for evaluation → Dermatologist clinical assessment → Deemed "undoubtedly benign" → NOT EXCISED (per Swedish clinical practice) → NO histopathological confirmation  ⚠️ CRITICAL ASSUMPTION: All 119 lesions truly benign  VERIFICATION DECISION CRITERION: Based on clinical suspicion at dermatologist level (NOT randomized, NOT uniform)  ═══════════════════════════════════════════════════════════════════════ BIAS CONSEQUENCE ═══════════════════════════════════════════════════════════════════════  RISK: Melanomas missed by dermatologist clinical assessment  IF any melanomas exist among 119 clinically-diagnosed lesions: → They would be counted as "benign" (false negative by dermatologist) → Would be classified as TRUE NEGATIVES in 2×2 table → Artificially INFLATES app's specificity → Artificially INFLATES app's NPV → Cannot be detected in this study design  FACTORS LIMITING DETECTION OF MISSED MELANOMAS: • No histopathological confirmation for 47% of lesions • Study period only 8 months (insufficient for delayed diagnosis) • Follow-up 2-9 months after index visit (mean 5 months, SD 1.5) • Thin melanomas can have unremarkable dermoscopic appearance  (1 in situ melanoma missed by app had "unremarkable appearance")  AUTHORS ACKNOWLEDGE: "We cannot exclude the possibility that the reference diagnosis might be incorrect for some lesions, particularly those that were not histopathologically analysed" (p.132)  Ethical justification provided: "excising all lesions...would have resulted in overtreatment with substantial costs and patient discomfort" (p.132)  ═══════════════════════════════════════════════════════════════════════ REPORTED DIAGNOSTIC PERFORMANCE (Potentially Biased) ═══════════════════════════════════════════════════════════════════════  Based on 253 lesions (21 melanomas, 232 benign)  All Melanomas (n=21): • AUROC: 0.960 (95% CI: 0.928-0.980) • Maximum sensitivity: 95.2% • Maximum specificity: 84.5% • At pre-specified cutoff:  - Sensitivity: 95.2% (20/21 detected)  - Specificity: 60.3% (140/232 correct negatives)  - PPV: 17.9%  - NPV: 99.3%  Invasive Melanomas Only (n=11): • AUROC: 0.988 (95% CI: 0.965-0.997) • Maximum sensitivity: 100% (all 11 detected) • Maximum specificity: 92.6%  Missed Melanoma: • 1/10 melanomas in situ missed by app • Patient #16: "unremarkable dermoscopic appearance"  ⚠️ INTERPRETATION CAUTION: Specificity and NPV may be overestimated due to differential verification bias (47% benign lesions not histopathologically confirmed) |
| --- |

# Phase 3: Risk of Bias and Applicability Judgments

# DOMAIN 1: PATIENT SELECTION

## A. Risk of Bias

**Describe methods of patient selection:**Prospective multicentre clinical trial at 36 PCCs across 7 regions in southern Sweden, May-December 2022. Total 138 PCPs (90 certified GPs, 48 resident trainees) trained to enrol participants. Patients aged ≥18 years presenting with skin lesions for which PCP had any suspicion of melanoma were eligible. Total 262 lesions in 237 patients assessed; 2 declined, 3 inclusion errors, 4 lost to follow-up; Final 253 lesions in 228 patients (125 women, 103 men, mean age 54). Exclusions: other cutaneous malignancies only, damaged/tattooed skin, inaccessible locations, dense hair, Fitzpatrick V-VI (algorithm not trained), poor image quality. CONCERN: Enrollment method not explicitly stated as consecutive or random; 7/36 centres contributed no lesions; participating centres 1-33 lesions each (suggests variable selection). Page reference: p.126-128, Figure 1

| Signaling Question | Answer |
| --- | --- |
| ❖ Was a consecutive or random sample enrolled? | UNCLEAR |
| ❖ Was a case-control design avoided? | YES |
| ❖ Did study avoid inappropriate exclusions? | YES |

**Could the selection of patients have introduced bias?: RISK: UNCLEAR**

**Justification:** While the study employed a prospective multicentre design with minimal exclusions (9/262 lesions, 3.4%), the enrollment method is not explicitly stated as consecutive or random. The paper describes patients as 'eligible for inclusion' but does not confirm that all eligible patients were consecutively enrolled. The significant variation in inclusion rates across centres (0-33 lesions per centre, with 7/36 centres contributing zero lesions) suggests potential selection variability that could introduce spectrum bias. However, there is no explicit evidence of convenience sampling, so the risk cannot be definitively rated as High.

## B. Concerns regarding applicability

**Is there concern that the included patients do not match the review question?: CONCERN: LOW**

**Justification:** This study directly matches the review's target population: real-world primary care patients presenting with skin lesions of concern for melanoma in a point-of-care setting. The population is representative of where AI-based melanoma detection tools would be deployed (primary care in Sweden). The mean age (54 years), sex distribution (55% female), and lesion characteristics are typical of primary care dermatology presentations. The only limitation is exclusion of Fitzpatrick V-VI skin types, which limits generalizability to diverse populations, but this is explicitly acknowledged.

# DOMAIN 2: INDEX TEST(S)

## A. Risk of Bias

**Describe the index test:**Dermalyser smartphone app (AI Medical Technology, Stockholm) - AI-based melanoma detection tool. ML algorithm trained in silico prior to study on extensive dermoscopic images (pretrial AUROC 0.94). Algorithm FIXED prior to data collection with no modification during study. Technical: continuous value 0-1 (melanoma probability), dichotomous output ('evidence detected' vs 'no evidence') based on pre-specified cutoff. Threshold determined PRIOR to data inclusion from in silico pretrial ROC curve, targeting sensitivity 95% and specificity 78%. Equipment: standardized - iPhone SE 2020 (iOS 14/15) + polarized light contact dermoscopes (Heine iC1 or DermLite DL3). CRITICAL BLINDING: PCPs recorded clinical assessment/management BEFORE applying app; app outcome NOT allowed to affect management or be communicated to patients. App used at presentation before diagnosis obtained. Page reference: p.126-127

| Signaling Question | Answer |
| --- | --- |
| ❖ Interpreted without knowledge of reference? | YES |
| ❖ If threshold used, was it pre-specified? | YES |

**Could the conduct of the index test have introduced bias?: RISK: LOW**

**Justification:** Both signaling questions are answered 'Yes.' The index test (AI app) was interpreted prospectively and automatically by a fixed algorithm that had no knowledge of reference standard results - this is inherent to the study design where the app was used at the point of care before diagnostic workup was completed. The threshold for the dichotomous output was explicitly pre-specified prior to data collection based on pretrial validation, not optimized on the study data. This eliminates both review bias and optimistic bias from post-hoc threshold selection.

## B. Concerns regarding applicability

**Is there concern about the index test applicability?: CONCERN: LOW**

**Justification:** The index test was used exactly as intended in real-world primary care: dermoscopic images taken by PCPs using standard clinical equipment, processed by the same fixed algorithm without modification. The testing protocol (dermoscopy + smartphone app) is directly applicable to point-of-care settings where the tool would be deployed. PCPs were specifically instructed not to let the app affect management, ensuring the study captured true index test performance rather than a human-AI hybrid decision.

# DOMAIN 3: REFERENCE STANDARD

## A. Risk of Bias

**Describe the reference standard:**Final clinical or histopathological tumour diagnosis from medical records. DIFFERENTIAL VERIFICATION: Two different reference standards: (1) Histopathology - 134/253 lesions (53%) excised with histopathological analysis; (2) Clinical diagnosis only - 119/253 lesions (47%) referred to dermatologist, deemed 'undoubtedly benign', NOT excised. All 21 detected melanomas received histopathological confirmation. HOWEVER, 119 lesions classified as benign based ONLY on dermatologist clinical assessment without histopathological verification. Decision based on clinical suspicion at dermatologist level (not uniform). BIAS CONSEQUENCE: If any melanomas among 119 clinically-diagnosed lesions were missed by dermatologist, they would be misclassified as true negatives, artificially inflating specificity and NPV. Short 8-month study period insufficient to capture delayed melanoma diagnoses. Authors acknowledge limitation and provide ethical justification. Follow-up 2-9 months (mean 5, SD 1.5). Page reference: p.127, p.132

| Signaling Question | Answer |
| --- | --- |
| ❖ Is reference standard likely to correctly classify? | NO |
| ❖ Interpreted without knowledge of index test? | YES |

**Could the reference standard have introduced bias?: RISK: HIGH**

**Justification:** HIGH risk due to differential verification bias. While histopathology (gold standard) was applied to 134/253 lesions (53%), the remaining 119/253 lesions (47%) received only clinical dermatologist assessment without histopathological confirmation. These lesions were deemed 'undoubtedly benign' and not excised per Swedish clinical practice. While all 21 detected melanomas were histopathologically confirmed, any melanomas missed by dermatologist clinical assessment among the 119 non-excised lesions would be misclassified as benign (false negatives), artificially inflating the app's specificity and NPV. The relatively short study period (8 months) means delayed melanoma diagnoses would not be captured. Authors acknowledge this limitation and note it is 'impossible to exclude the possibility that the reference diagnosis might be incorrect for some lesions, particularly those that were not histopathologically analysed.'

## B. Concerns regarding applicability

**Is there concern about reference standard applicability?: CONCERN: LOW**

**Justification:** For the 134 lesions that received it, histopathology is the appropriate gold standard for melanoma diagnosis. Clinical dermatologist assessment for the 119 non-excised lesions represents standard clinical practice in Sweden and is reasonable for lesions with very low suspicion. The target condition definition (melanoma including in situ and invasive) is appropriate. The main concern is not with the reference standard itself but with its differential application (addressed under Risk of Bias).

# DOMAIN 4: FLOW AND TIMING

## A. Risk of Bias

**Patient flow:**All 253 lesions received index test (Dermalyser app) at presentation. All 253 also received reference diagnosis (either histopathology or clinical assessment). HOWEVER, differential verification present: 134/253 (53%) received histopathological reference, 119/253 (47%) received clinical dermatologist assessment only. Decision about which reference standard NOT random or uniform - based on clinical suspicion at dermatologist level. Lesions deemed 'undoubtedly benign' not excised. All 21 melanomas histopathologically confirmed. Creates bias: if any melanomas among 119 clinically-diagnosed lesions were missed, they would be misclassified as true negatives, artificially inflating specificity/NPV. Minimal loss to follow-up (4/262 = 1.6%). Excellent retention (98.4%). STARD-compliant flow diagram provided. Time interval not explicitly stated but app used at presentation, follow-up 2-9 months (mean 5, SD 1.5). Page reference: p.127-128, Figure 1, p.132

| Signaling Question | Answer |
| --- | --- |
| ❖ Appropriate interval between tests? | YES |
| ❖ Did all patients receive reference standard? | YES |
| ❖ Did patients receive same reference standard? | NO |
| ❖ Were all patients included in analysis? | YES |

**Could the patient flow have introduced bias?: RISK: HIGH**

**Justification:** HIGH risk due to clear differential verification bias. The study explicitly states that 134/253 lesions (53%) received histopathological reference and 119/253 lesions (47%) received clinical dermatologist assessment only. The decision about which reference standard to apply was not random or uniform - it was based on clinical suspicion at the dermatologist level. Lesions deemed 'undoubtedly benign' by dermatologists were not excised. This creates a methodological concern: if any melanomas among the 119 clinically-diagnosed lesions were missed by the dermatologist, they would be misclassified as true negatives, artificially inflating the app's specificity and NPV. While the authors provide ethical justification for not excising all lesions, and all 21 detected melanomas did receive histopathological confirmation, the risk of missed melanomas in the non-excised group cannot be excluded. The relatively short study period (8 months) means delayed melanoma diagnoses would not be captured.

# OVERALL ASSESSMENT SUMMARY

**HIGH RISK OF BIAS - DIFFERENTIAL VERIFICATION**

| Domain | Signaling Questions | Risk of Bias | Applicability |
| --- | --- | --- | --- |
| D1: Patient Selection | 1.1 Unclear 1.2 Yes 1.3 Yes | UNCLEAR | LOW |
| D2: Index Test | 2.1 Yes 2.2 Yes | LOW | LOW |
| D3: Reference Standard | 3.1 No 3.2 Yes | HIGH | LOW |
| D4: Flow and Timing | 4.1 Yes 4.2 Yes 4.3 No 4.4 Yes | HIGH | N/A |

**Overall Risk of Bias: HIGH**

**Justification:** Two domains (Domain 3: Reference Standard, Domain 4: Flow and Timing) are rated High risk due to differential verification bias. While this study has significant methodological strengths - prospective design, pre-specified threshold, blinded index test interpretation, minimal loss to follow-up (1.6%), and clear STARD-compliant flow diagram - the use of different reference standards for different patients (histopathology vs. clinical diagnosis based on clinical suspicion) is a fundamental bias that affects the validity of accuracy estimates. Specifically: 119/253 lesions (47%) were classified based only on dermatologist clinical assessment without histopathological confirmation. While all 21 detected melanomas were histopathologically confirmed, any false negative clinical diagnoses among the non-excised lesions would go undetected, potentially inflating specificity and NPV. The authors acknowledge this limitation and provide ethical justification.

**Overall Applicability Concern: LOW**

**Justification:** All three applicable domains have Low applicability concern. This study directly matches the review question: AI-based clinical decision support for melanoma detection in point-of-care primary care settings. The population (real-world primary care patients with skin lesions of concern), index test (smartphone dermoscopy app used as intended), and reference standard definitions (histopathology for melanoma) are all highly applicable. The main limitation is the Swedish/Northern European population with predominantly lighter skin types, limiting generalizability to more diverse populations.

## Critical Biases Identified

**1. DIFFERENTIAL VERIFICATION BIAS (SEVERITY: HIGH):**Different reference standards applied based on clinical suspicion. 134/253 (53%) received histopathology (gold standard); 119/253 (47%) received clinical dermatologist assessment only without tissue confirmation. Lesions deemed "undoubtedly benign" by dermatologist were not excised per Swedish clinical practice. Creates risk: if any melanomas exist among 119 clinically-diagnosed lesions, they would be misclassified as benign (false negatives), artificially inflating specificity and NPV. All 21 detected melanomas were histopathologically confirmed, but missed melanomas in non-excised group cannot be excluded. Short 8-month study insufficient to capture delayed diagnoses.

**2. POTENTIAL SELECTION BIAS (SEVERITY: UNCLEAR):**Enrollment method not explicitly stated as consecutive. Variable inclusion across centres: 7/36 centres contributed no lesions, participating centres 1-33 lesions each. Suggests potential selection variability.

**3. SPECTRUM BIAS RISK (SEVERITY: MODERATE):**Fitzpatrick V-VI skin types excluded (algorithm not trained on these). Study population predominantly Fitzpatrick I-II (Northern European). Limits generalizability to diverse populations.

## Significant Methodological Strengths

**Despite differential verification bias, this study has important strengths:

✓ Prospective real-life design:** One of few prospective studies of AI melanoma detection in primary care
**✓ Pre-specified threshold:** Cutoff determined PRIOR to data collection from pretrial validation (not optimized on study data)
**✓ Fixed algorithm:** No modification during study period (eliminates training contamination)
**✓ Appropriate blinding:** App applied before diagnosis known; PCPs blinded to app output for management decisions
**✓ Multicentre:** 36 PCCs, 138 physicians (enhances generalizability)
**✓ Excellent retention:** 98.4% of eligible lesions analyzed (9/262 excluded = 3.4%)
**✓ STARD compliance:** Clear flow diagram and methods reporting
**✓ Clinical validity:** All 21 detected melanomas histopathologically confirmed
**✓ Real-world POC setting:** Primary care physicians using smartphone app during routine visits

## Reported Performance (Potentially Biased)

**Specificity and NPV may be overestimated due to differential verification

All Melanomas (n=21):**• AUROC: 0.960 (95% CI: 0.928-0.980)
• At pre-specified cutoff:
 - Sensitivity: 95.2% (20/21 detected)
 - Specificity: 60.3% (140/232)
 - PPV: 17.9%
 - NPV: 99.3%

**Invasive Melanomas Only (n=11):**• AUROC: 0.988 (95% CI: 0.965-0.997)
• Sensitivity: 100% (all 11 detected)
• Specificity: 92.6% at maximum

**Missed Melanoma:**• 1/10 melanomas in situ missed
• Patient #16: "unremarkable dermoscopic appearance"

**Sample Characteristics:**• All invasive melanomas THIN (Breslow 0.1-1.1mm)
• 91% classified as T1a
• Only 21 melanomas total (limits sensitivity precision)

**CRITICAL CAVEAT:** 119/253 (47%) benign lesions diagnosed by clinical assessment only (no histopathology). If any melanomas were missed by dermatologist among these 119, they would be misclassified as true negatives, inflating specificity (60.3%) and NPV (99.3%).

## Study Value and Limitations

**What this study demonstrates:

✓ POC feasibility:** AI dermoscopy app can be used by PCPs in routine practice
**✓ High sensitivity:** Detected 20/21 melanomas (95.2%); 100% of invasive melanomas
**✓ Real-world multicentre:** 36 centres, 138 physicians, 8-month period
**✓ Rigorous methodology:** Pre-specified threshold, fixed algorithm, appropriate blinding

**Important limitations:

✗ Differential verification:** 47% benign lesions not histopathologically confirmed
**✗ Small sample:** Only 21 melanomas (11 invasive, 10 in situ)
**✗ Thin melanomas only:** All invasive melanomas were thin (Breslow 0.1-1.1mm, 91% T1a)
**✗ Limited diversity:** Fitzpatrick V-VI excluded, predominantly I-II population
**✗ Short follow-up:** 8-month study insufficient for delayed melanoma detection
**✗ Enrollment unclear:** Not explicitly consecutive, variable centre participation

QUADAS-2

**Poli et al. 2024**Development and Clinical Validation of Visual Inspection With Acetic Acid Application-Artificial Intelligence Tool Using Cervical Images in Screen-and-Treat Visual Screening for Cervical Cancer in South India: A Pilot Study *Study ID: Poli_2024 | Assessment Date: 2025-11-28*

____________________________________________________________________________________________________

# Phase 1: State the Review Question

*Patients (setting, intended use of index test, presentation, prior testing):*

| Women aged >25 years attending opportunistic cervical cancer screening at community-level cancer screening clinics in Telangana, India, December 2021-May 2022. Setting: Point-of-care - nurse-led community-level screening program in rural low-resource setting (nurses with 3-12 years VIA experience). Intended use: AI-based clinical decision support (VIA-AI tool) to classify cervical lesions after VIA testing for screen-and-treat triage decisions. Sequential recruitment: 3,010 eligible women enrolled; 2,052 women with cervical images analyzed (958/3,010 = 32% excluded for missing images - reasons not detailed). Population: Mean age 39.53±9.43 years (range 25-70); Age distribution: 25-29 (17.3%), 30-34 (14.3%), 35-39 (19%), 40-44 (14.6%), 45-49 (17%), 50-59 (14.3%), >60 (3.5%). Rural background, low-resource LMIC setting. Disease prevalence: 88/2,052 (4.29%) confirmed CIN1+ disease. CRITICAL LIMITATION - VERIFICATION BIAS: Only 183/2,052 (8.9%) received histopathology (Swede score >4); 1,869/2,052 (91.1%) classified based on colposcopy alone without tissue confirmation. High exclusion rate (32%) concerning if image quality correlated with disease status (characteristics of excluded vs included patients not reported). |
| --- |

*Index test(s):*

| VIA-AI tool developed using deep learning (transfer learning with EfficientNet B3 and EfficientNet V2 CNN models) trained on 2,186 low-power colposcopy images from locally sourced image banks. During Phase II clinical validation: (1) VIA screening performed first, (2) Colposcopy examination with Swede score, (3) Images captured from video colposcope monitor (6× magnification) using Android smartphone cameras (20-megapixel) held parallel at 2 feet distance, (4) One image per woman captured 1 minute after 4% acetic acid application. AI software ran captured images in BLINDED manner without clinical details, producing classification: positive (requiring treatment) or negative. AI categories: (1) normal/no treatment, (2) abnormal requiring ablative treatment, (3) requiring excision treatment, (4) suspicious cancer requiring definitive treatment. Classification based on acetowhite area intensity and margin scores (binary Score 0 vs Score 1). Thresholds pre-specified during Phase I algorithm development (80:20 train/test split). Internal cross-validation (Phase I): 78% accuracy intensity scores, 72% margin scores, 82% invasive cancer identification. STRENGTHS: Automated interpretation, blinded to reference, pre-specified thresholds. POTENTIAL LIMITATION: Developed and tested by same research group in similar populations (internal validation overfitting risk); smartphone capture from monitor (not direct digital) introduces variability but reflects real-world conditions. |
| --- |

*Reference standard and target condition:*

| CRITICAL FLAW - DIFFERENTIAL & PARTIAL VERIFICATION BIAS: Two different reference standards applied based on colposcopy findings. (1) Histopathology: Only 183/2,052 (8.9%) women with Swede score >4 underwent cervical biopsy for histologic confirmation. Biopsies performed on same day as index test. Of 183 biopsied: 88 confirmed CIN1+, 95 negative. (2) Colposcopy only: Remaining 1,869/2,052 (91.1%) women with Swede score ≤4 classified based SOLELY on colposcopy assessment without tissue confirmation. SEVERE BIAS CONSEQUENCE: If AI correctly identified CIN1+ case that colposcopy missed (Swede ≤4), this would be counted as AI FALSE POSITIVE because patient would NOT receive biopsy to confirm disease. True specificity likely LOWER than reported (97.6%) because some AI-positive/colposcopy-negative cases may have been true positives. Cannot detect false negatives among 91% unbiopsied women. Decision criterion: Based on colposcopy Swede score, NOT randomized or uniform. Study acknowledges: 'cervical biopsy for histologic confirmation was performed on women with a Swede score greater than four.' Target condition: CIN1+ (cervical intraepithelial neoplasia grade 1 or higher, including CIN1, CIN2, CIN3, invasive cancer). Reference operator: Colposcopist with unspecified experience, Swede scoring system used. |
| --- |

# Phase 2: Draw a Flow Diagram for the Primary Study

| PATIENT FLOW DIAGRAM - PILOT STUDY (PHASE II CLINICAL VALIDATION)  STUDY PERIOD: December 2021 - May 2022 (6 months) SETTING: Community-level cancer screening clinics  Telangana, India (rural, low-resource LMIC) PROGRAM: Ongoing opportunistic screen-and-treat visual screening OPERATORS: Nurses with 3-12 years VIA screening experience  ↓ ELIGIBLE: Women aged >25 years attending screening clinics  Sequential recruitment  ↓ ENROLLED: Approximately 3,010 women  ↓ ⚠️ SUBSTANTIAL EXCLUSIONS (Unclear Reasons)  ↓ EXCLUDED: 958 women (31.8% of enrolled) Primary reason: Missing cervical images • Specific breakdown NOT provided • Reasons for missing images NOT detailed • Characteristics of excluded vs included NOT reported • CONCERN: If image quality correlated with disease status → selection bias  ↓ FINAL ANALYTICAL SAMPLE: 2,052 women  POPULATION CHARACTERISTICS: • Mean age: 39.53 ± 9.43 years (range 25-70) • Age distribution:  - 25-29: 17.3%  - 30-34: 14.3%  - 35-39: 19.0%  - 40-44: 14.6%  - 45-49: 17.0%  - 50-59: 14.3%  - >60: 3.5% • Rural background • Opportunistic screening attendees  ↓ ═══════════════════════════════════════════════════════════════════════ ALL 2,052 WOMEN RECEIVED STANDARDIZED SCREENING PROTOCOL ═══════════════════════════════════════════════════════════════════════  STEP 1: VIA Screening (Visual Inspection with Acetic Acid) • 4% acetic acid application • Visual inspection by trained nurse  STEP 2: Colposcopy Examination • Video colposcope at 6× magnification • Swede score assessment (0-10 scale)  - Acetowhite area intensity  - Margins and surface  - Lesion size  - Suspicious for invasion  - Suspicious for cancer  STEP 3: Image Capture (INDEX TEST) • Timing: 1 minute after acetic acid application • Method: Android smartphone camera (20-megapixel resolution) • Position: Held parallel at 2 feet distance from video colposcope monitor • Captured: One image per woman • NOTE: Indirect capture (smartphone from monitor) not direct digital  STEP 4: AI Analysis (INDEX TEST) • VIA-AI software (EfficientNet B3/V2 CNN models) • Trained on 2,186 images from local image banks • BLINDED analysis (no clinical details provided to AI) • Classification output:  1. Normal / No treatment required  2. Abnormal requiring ablative treatment  3. Requiring excision treatment  4. Suspicious cancer requiring definitive treatment • Binary scores: Intensity (0 vs 1), Margins (0 vs 1)  ↓ ═══════════════════════════════════════════════════════════════════════ CRITICAL: DIFFERENTIAL & PARTIAL VERIFICATION BIAS ═══════════════════════════════════════════════════════════════════════  VERIFICATION DECISION CRITERION: Colposcopy Swede Score  PATHWAY 1: High Suspicion (Swede Score >4) → BIOPSY PERFORMED (n=183, 8.9%) → Histopathology (GOLD STANDARD) → Results:  • CIN1+: 88 (48.1% of biopsied)  • Negative: 95 (51.9% of biopsied)  Disease breakdown (88 CIN1+ cases): • CIN1: number not specified • CIN2+: number not specified • Invasive cancer: included but number not specified  PATHWAY 2: Low Suspicion (Swede Score ≤4) → NO BIOPSY PERFORMED (n=1,869, 91.1%) → Colposcopy assessment ONLY (not gold standard) → Assumed negative based on colposcopy alone → NO histopathologic confirmation  ═══════════════════════════════════════════════════════════════════════ SEVERE BIAS CONSEQUENCE ═══════════════════════════════════════════════════════════════════════  PROBLEM: Cannot detect false negatives among 91% unbiopsied women  SCENARIO 1: AI correctly identifies CIN1+ case  + Colposcopy misses it (Swede ≤4)  = No biopsy performed  = Counted as AI FALSE POSITIVE (incorrectly)  = True disease status unknown  SCENARIO 2: AI misses CIN1+ case  + Colposcopy also misses it (Swede ≤4)  = No biopsy performed  = Both tests appear concordant negative  = False negative undetectable  RESULT: • True specificity likely LOWER than reported (97.56%) • AI-positive/colposcopy-negative cases may be true positives • Sensitivity estimates may be misleading (denominator uncertain)  STUDY ACKNOWLEDGES: "Cervical biopsy for histologic confirmation was performed on women with a Swede score greater than four" (p.4)  But does NOT acknowledge implications for bias  ═══════════════════════════════════════════════════════════════════════ REPORTED PERFORMANCE (Against "Final Diagnosis") ═══════════════════════════════════════════════════════════════════════  Based on 2,052 women Disease prevalence: 88/2,052 (4.29%) confirmed CIN1+  VIA-AI Performance: • Sensitivity: 62.50% (95% CI: 51.53-72.60) • Specificity: 97.56% (95% CI: 96.77-98.19) • AUC: 0.7585  ⚠️ INTERPRETATION ISSUES: 1. Sensitivity based on only 88 disease-positive cases (wide CI) 2. Specificity likely INFLATED (91% not histopathologically confirmed) 3. "Final diagnosis" for 1,869 women is colposcopy, not histopathology 4. True false-negative rate unknown  ERROR ANALYSIS (Valuable transparency): 402/2,052 (20%) incorrect AI interpretations Causes: • Thick mucus: 24% • Inflammation/squamous metaplasia: 20% • Glare spots: 15.5% • Poor image quality: 15% • Ectropion: 10.6%  ═══════════════════════════════════════════════════════════════════════ SUMMARY OF BIAS ISSUES ═══════════════════════════════════════════════════════════════════════  1. PARTIAL VERIFICATION BIAS:  Only 8.9% received gold standard histopathology  2. DIFFERENTIAL VERIFICATION BIAS:  Different reference standards based on colposcopy findings  (not randomized or uniform)  3. INCORPORATION BIAS:  Reference standard pathway determined by another test (colposcopy)  4. SUBSTANTIAL EXCLUSIONS:  32% excluded for missing images (reasons unclear)  5. POTENTIAL SPECTRUM BIAS:  Developed and tested in same geographic region/population |
| --- |

# Phase 3: Risk of Bias and Applicability Judgments

# DOMAIN 1: PATIENT SELECTION

## A. Risk of Bias

**Describe methods of patient selection:**Exploratory interventional study, Phase II clinical validation. Approximately 3,010 eligible women sequentially recruited and screened, December 2021-May 2022, community-level cancer screening clinics, Telangana, India. Recruitment from ongoing opportunistic screen-and-treat visual cervical screening program. Women >25 years attending screening clinics eligible. Rural background population. Data from 2,052 women with cervical images analyzed; those with missing images excluded. Exclusions: 958/3,010 (32%) women excluded - specific reasons not provided. High exclusion rate concerning. Characteristics of excluded vs included patients NOT reported to assess if exclusions random. Prospective cohort design. Page reference: p.4, p.5

| Signaling Question | Answer |
| --- | --- |
| ❖ Was a consecutive or random sample enrolled? | YES |
| ❖ Was a case-control design avoided? | YES |
| ❖ Did study avoid inappropriate exclusions? | UNCLEAR |

**Could the selection of patients have introduced bias?: RISK: UNCLEAR**

**Justification:** Questions 1.1 and 1.2 were answered 'Yes' (consecutive enrollment, cohort design). However, Question 1.3 is Unclear due to a substantial exclusion rate: 958/3,010 (32%) women were excluded, primarily for missing cervical images. The high exclusion rate is concerning because the reasons for missing images are not detailed - if technical difficulties with image capture were related to cervical pathology (e.g., heavy bleeding, large lesions, anatomical variations), this could introduce selection bias. The paper does not report characteristics of excluded vs. included patients to assess whether exclusions were random.

## B. Concerns regarding applicability

**Is there concern that the included patients do not match the review question?: CONCERN: LOW**

**Justification:** The study population matches the review question well. The women attended point-of-care cervical screening in a community-level, low-resource setting in South India - exactly the intended use case for this AI decision support tool. The opportunistic screening setting with nurse-led implementation reflects the real-world application of AI-assisted screening in low- and middle-income countries where the technology is designed to be deployed. The age range and rural population characteristics align with cervical cancer screening guidelines.

# DOMAIN 2: INDEX TEST(S)

## A. Risk of Bias

**Describe the index test:**VIA-AI tool: deep learning (transfer learning EfficientNet B3/V2 CNNs) trained on 2,186 low-power colposcopy images from local image banks. Phase II validation: VIA screening → colposcopy with Swede score → image capture from video colposcope monitor (6× magnification) using Android smartphone (20-megapixel) at 2 feet → one image 1 minute after 4% acetic acid. AI ran images BLINDED without clinical details, producing classification: positive (requiring treatment) or negative. Categories: normal/no treatment, abnormal requiring ablative treatment, requiring excision, suspicious cancer. Classification based on acetowhite intensity and margin scores (binary 0 vs 1). Thresholds pre-specified during Phase I development (80:20 train/test). Internal cross-validation: 78% accuracy intensity, 72% margins, 82% invasive cancer. NOTE: Same research group developed and tested in similar populations (internal validation overfitting risk). Page reference: p.3, p.4, p.10

| Signaling Question | Answer |
| --- | --- |
| ❖ Interpreted without knowledge of reference? | YES |
| ❖ If threshold used, was it pre-specified? | YES |

**Could the conduct of the index test have introduced bias?: RISK: LOW**

**Justification:** Both signaling questions were answered 'Yes.' The AI interpretation was automated and blinded to reference standard results. The classification thresholds were pre-specified during Phase I development on separate training/validation datasets before Phase II clinical testing. However, it should be noted that the AI tool was developed and tested by the same research group in similar populations, which could represent internal validation overfitting. The use of smartphone cameras to capture images from a video colposcope monitor (rather than direct digital capture) may introduce variability, but this reflects real-world implementation conditions and does not constitute bias in interpretation.

## B. Concerns regarding applicability

**Is there concern about the index test applicability?: CONCERN: LOW**

**Justification:** The VIA-AI tool was specifically designed for point-of-care use in low-resource settings, with a mobile application for phones/tablets that nurses and health workers can use at health centers. The imaging setup (video colposcope with smartphone image capture) reflects practical constraints of the intended deployment setting. The AI was developed using images from women in the same geographic region to match local morphology and ethnicity. The implementation by trained nurses with 3-12 years of VIA experience aligns with the intended user profile for screen-and-treat programs in low- and middle-income countries.

# DOMAIN 3: REFERENCE STANDARD

## A. Risk of Bias

**Describe the reference standard:**DIFFERENTIAL & PARTIAL VERIFICATION: Two reference standards applied based on colposcopy findings. (1) Histopathology: Only 183/2,052 (8.9%) women with Swede score >4 underwent cervical biopsy (gold standard). Biopsies same day as index test. Of 183: 88 confirmed CIN1+, 95 negative. (2) Colposcopy only: 1,869/2,052 (91.1%) women with Swede ≤4 classified based SOLELY on colposcopy without tissue confirmation. SEVERE BIAS: If AI correctly identified CIN1+ case that colposcopy missed (Swede ≤4), counted as AI FALSE POSITIVE because no biopsy to confirm disease. True specificity likely LOWER than reported (97.6%). Cannot detect false negatives among 91% unbiopsied. Decision: Based on colposcopy Swede score, NOT uniform. Study states: 'cervical biopsy for histologic confirmation was performed on women with a Swede score greater than four.' Target: CIN1+ (CIN1, CIN2, CIN3, invasive cancer). Page reference: p.4

| Signaling Question | Answer |
| --- | --- |
| ❖ Is reference standard likely to correctly classify? | NO |
| ❖ Interpreted without knowledge of index test? | YES |

**Could the reference standard have introduced bias?: RISK: HIGH**

**Justification:** HIGH risk due to severe partial and differential verification bias. Only 183/2,052 (8.9%) women received the gold standard histopathology, while 1,869/2,052 (91.1%) were classified based solely on colposcopy without tissue confirmation. The decision to biopsy was based on colposcopy Swede score (>4), not randomized or uniform. This creates fundamental bias: if AI correctly identified a CIN1+ case that colposcopy missed (Swede ≤4), it would be counted as a false positive because no biopsy would confirm the disease. Specificity is likely inflated because some AI-positive/colposcopy-negative cases may have been true positives that went undetected. Cannot assess false negatives among 91% unbiopsied women.

## B. Concerns regarding applicability

**Is there concern about reference standard applicability?: CONCERN: LOW**

**Justification:** For the 183 women who received it, histopathology is the appropriate gold standard for cervical neoplasia diagnosis. Colposcopy with Swede scoring for the remaining 1,869 women represents standard clinical practice in resource-limited settings where universal biopsy is not feasible. The target condition definition (CIN1+) is appropriate for cervical cancer screening. The main concern is not with the reference standard itself but with its differential application (addressed under Risk of Bias).

# DOMAIN 4: FLOW AND TIMING

## A. Risk of Bias

**Patient flow:**All 2,052 women received index test (VIA-AI) and reference standard, BUT with differential verification creating severe bias. Only 183/2,052 (8.9%) received histopathology (gold standard); 1,869/2,052 (91.1%) received colposcopy only. Decision based on Swede score, not uniform. Time interval appropriate (biopsy same day as index test). HOWEVER, substantial pre-analytical exclusions: 958/3,010 (32%) enrolled women excluded for missing images (specific reasons not provided). Creates flow bias if excluded patients systematically different. Final analysis included all 2,052 with images in 2×2 table, but reference standard non-uniform. Cannot detect AI false positives that are true positives missed by colposcopy, nor false negatives among unbiopsied majority. Page reference: p.4, p.5

| Signaling Question | Answer |
| --- | --- |
| ❖ Appropriate interval between tests? | YES |
| ❖ Did all patients receive reference standard? | NO |
| ❖ Did patients receive same reference standard? | NO |
| ❖ Were all patients included in analysis? | NO |

**Could the patient flow have introduced bias?: RISK: HIGH**

**Justification:** HIGH risk due to multiple flow problems: (1) Differential verification - only 183/2,052 (8.9%) received gold standard histopathology, 1,869/2,052 (91.1%) received colposcopy only, decision based on Swede score not uniform; (2) Partial verification - vast majority (91%) not verified with gold standard; (3) Substantial exclusions - 958/3,010 (32%) enrolled women excluded for missing images without detailed explanation of reasons or comparison of characteristics. These issues create severe bias: cannot detect false negatives among unbiopsied majority, AI-positive/colposcopy-negative cases may be misclassified false positives when they are actually true positives. Reported specificity (97.56%) likely inflated.

# OVERALL ASSESSMENT SUMMARY

**HIGH RISK OF BIAS - SEVERE VERIFICATION BIAS**

| Domain | Signaling Questions | Risk of Bias | Applicability |
| --- | --- | --- | --- |
| D1: Patient Selection | 1.1 Yes 1.2 Yes 1.3 Unclear | UNCLEAR | LOW |
| D2: Index Test | 2.1 Yes 2.2 Yes | LOW | LOW |
| D3: Reference Standard | 3.1 No 3.2 Yes | HIGH | LOW |
| D4: Flow and Timing | 4.1 Yes 4.2 No 4.3 No 4.4 No | HIGH | N/A |

**Overall Risk of Bias: HIGH**

**Justification:** HIGH RISK: Two domains rated High for Risk of Bias. Domain 3 (Reference Standard) is High due to verification bias - only 8.9% of patients (183/2,052) received histopathologic confirmation, with 91.1% classified based on colposcopy alone. Domain 4 (Flow and Timing) is High due to differential verification (different reference standards based on clinical findings) and substantial exclusions (32% of enrolled patients excluded from analysis). These methodological limitations mean the reported sensitivity (62.5%) and specificity (97.6%) may be biased - specificity is likely inflated because false negatives among the unbiopsied majority cannot be detected, and AI-positive/colposcopy-negative cases may be misclassified as false positives when they could be true positives.

**Overall Applicability Concern: LOW**

**Justification:** All applicable domains have Low applicability concern. This study addresses AI-based cervical cancer screening in exactly the intended context: community-level, nurse-led, point-of-care screening in a low-resource LMIC setting (South India). The VIA-AI tool was designed for this specific application, the operators (nurses) match the intended user profile, and the population (rural women attending opportunistic screening) matches the target population for screen-and-treat programs in resource-limited settings.

## Critical Biases Identified

**1. PARTIAL VERIFICATION BIAS (SEVERITY: SEVERE):**Only 183/2,052 (8.9%) women received gold standard histopathology. The remaining 1,869/2,052 (91.1%) were classified based solely on colposcopy without tissue confirmation. True disease status unknown for 91% of cohort. False negatives among unbiopsied majority cannot be detected.

**2. DIFFERENTIAL VERIFICATION BIAS (SEVERITY: SEVERE):**Different reference standards applied based on colposcopy Swede score: Swede >4 → biopsy (gold standard); Swede ≤4 → colposcopy only (not gold standard). Decision NOT randomized or uniform. Creates fundamental bias in accuracy assessment.

**3. INCORPORATION BIAS (SEVERITY: MODERATE):**Reference standard pathway determined by colposcopy test results. If AI correctly identifies CIN1+ that colposcopy misses (Swede ≤4), patient receives NO biopsy → counted as AI false positive (incorrect classification). True positives misclassified as false positives.

**4. SUBSTANTIAL EXCLUSIONS (SEVERITY: UNCLEAR):**958/3,010 (32%) enrolled women excluded for missing cervical images. Specific reasons NOT provided. Characteristics of excluded vs included patients NOT reported. Could introduce selection bias if image quality correlated with disease status (e.g., bleeding from lesions, anatomical variations).

## Reported Performance (LIKELY BIASED)

**Specificity likely INFLATED due to verification bias**Based on 2,052 women:
• Disease prevalence: 88/2,052 (4.29%) confirmed CIN1+
• Only 8.9% received histopathology (gold standard)
• 91.1% classified by colposcopy alone

**VIA-AI Performance:**• Sensitivity: 62.50% (95% CI: 51.53-72.60)
 → Based on only 88 disease-positive cases (wide CI)
 → Modest sensitivity

• Specificity: 97.56% (95% CI: 96.77-98.19)
 → LIKELY INFLATED
 → 91% not histopathologically confirmed
 → AI-positive/colposcopy-negative may be true positives

• AUC: 0.7585 (moderate discrimination)

**Error Analysis (20% incorrect interpretations):**Valuable transparency about AI limitations:
• Thick mucus: 24%
• Inflammation/squamous metaplasia: 20%
• Glare spots: 15.5%
• Poor image quality: 15%
• Ectropion: 10.6%

**CRITICAL LIMITATION:** "Final diagnosis" for 1,869 women is colposcopy assessment, NOT histopathology. Cannot assess true diagnostic accuracy without uniform gold standard verification.

## Study Value and Limitations

**What this pilot study demonstrates:

✓ POC feasibility:** AI-assisted VIA screening implementable in community-level, nurse-led programs
**✓ LMIC context:** Real-world low-resource setting in rural South India
**✓ Transparency:** Excellent error analysis identifying specific AI failure modes
**✓ Local development:** AI trained on images from same geographic region (matches local morphology)
**✓ Pragmatic design:** Smartphone capture from colposcope monitor reflects real-world constraints

**Critical limitations:

✗ Severe verification bias:** Only 8.9% received gold standard (91% unverified)
**✗ Differential verification:** Reference standard based on colposcopy Swede score (not uniform)
**✗ Inflated specificity:** Cannot detect true positives missed by colposcopy
**✗ High exclusion rate:** 32% excluded for missing images (reasons unclear)
**✗ Small disease sample:** Only 88 CIN1+ cases (wide sensitivity CI)
**✗ Modest sensitivity:** 62.5% means 37.5% of disease missed
**✗ Internal validation:** Same group developed and tested (overfitting risk)
**✗ Limited generalizability:** Single geographic region, needs external validation

QUADAS-2

**Yang et al. 2019**Kankanet: An artificial neural network-based object detection smartphone application and mobile microscope as a point-of-care diagnostic aid for soil-transmitted helminthiases *Study ID: Yang_2019 | Assessment Date: 2025-11-28*

____________________________________________________________________________________________________

# Phase 1: State the Review Question

*Patients (setting, intended use of index test, presentation, prior testing):*

Participants from two rural villages in Madagascar (Mangevo n=238 total population, Ambinanindranofotaka n=327 total population) near Ranomafana National Park, June 8-18, 2018. Setting: Point-of-care - extremely remote rural setting (accessible only by 14 hours of footpaths), resource-limited, STH-endemic area. Intended use: Smartphone-based AI microscopy (Kankanet) for field diagnosis of soil-transmitted helminthiases by non-specialists without laboratory access. Enrollment: CONVENIENCE/VOLUNTEER sample - all village residents given oral presentation about STH, subjects aged ≥16 who gave voluntary consent enrolled (parents consented for assenting children). NOT consecutive or random sampling. Enrolled: 113 participants who provided fecal samples. Sample size: Pre-calculated at 115 based on hookworm prevalence (33.1%) targeting 90% sensitivity with 10% margin of error. Exclusion: Cognitive impairment only (appropriate for consent). Population: Over 95% households engage in subsistence farming. CRITICAL LIMITATION - SELECTION BIAS: Self-selected volunteers after STH presentation may create spectrum bias (symptomatic or health-conscious individuals more likely to participate, asymptomatic individuals or those unable to attend excluded). Non-random enrollment fundamentally limits generalizability.

*Index test(s):*

Two index tests evaluated: (1) USB Video Class (UVC) smartphone microscopy (Magnification Endoscope, Jiusion Tech + Digital Microscope Stand, iTez) read by parasitologists, (2) Kankanet - AI-based object detection system using TensorFlow with Single Shot Detection meta-architecture and MobileNet feature extractor. AI training: 2,078 images (597 microscope images, 1,481 UVC images) of A. lumbricoides, T. trichiura, and hookworm eggs. Two models trained: Model 1 (microscope images only, n=597), Model 2 (all images, n=2,078). Validation: Separate evaluation set of 186 images. Detection confidence threshold: 0.60 (TensorFlow demo application default). Training stopping: Loss <0.01 (default configuration). Equipment: Commercially available ($69.82 total cost), operates offline without internet. Blinding: Clinical information and results from other analyses NOT made available to slide readers during analysis. STRENGTH: Automated AI interpretation, blinded to reference. LIMITATION - THRESHOLD UNCERTAINTY: 0.60 threshold described as 'per original app' default, but not explicitly stated as pre-specified before data collection. Potential overfitting: Same population used for image acquisition and clinical validation. Whether any parameters were optimized during study not explicitly reported.

*Reference standard and target condition:*

'Modified gold standard' defined as any positive result from parasitologist reading of THREE standard microscopy preparations: (1) Kato-Katz (KK) from fresh stool, (2) spontaneous sedimentation technique in tube (SSTT) from 10% formalin-preserved stool, (3) Merthiolate-Iodine-Formaldehyde (MIF) from 10% formalin-preserved stool. Performed by: Trained parasitologists at Institut Pasteur de Madagascar. Each fecal sample produced three slides for analysis. Intensity measurement: Egg counts on KK slides × coefficient of 24. LIMITATION - Hookworm: KK readings NOT obtainable due to egg disintegration during transport to laboratory facility (Table 2 footnote). Composite reference increases sensitivity by using multiple methods. KK established performance: Sensitivity 0.97 (A. lumbricoides), 0.65 (hookworm), 0.91 (T. trichiura); Specificity 0.96, 0.94, 0.94 respectively. BLINDING UNCERTAINTY: Paper states clinical information not available to slide readers during analysis, but appears to refer to index test readers. NOT explicitly stated whether parasitologists reading standard microscopy were blinded to UVC/Kankanet results. Given field logistics, blinding may have been impractical. Target conditions: Soil-transmitted helminthiases (A. lumbricoides, T. trichiura, hookworm). All 113 participants received complete reference standard (no differential verification).

# Phase 2: Draw a Flow Diagram for the Primary Study

```

PATIENT FLOW DIAGRAM - PROSPECTIVE CROSS-SECTIONAL STUDY

STUDY PERIOD: June 8-18, 2018 (11 days)
SETTING: Two rural villages near Ranomafana National Park, Madagascar
• Mangevo: 238 total population
• Ambinanindranofotaka: 327 total population
• Access: 14 hours by footpaths only (extremely remote)
• Population: >95% households subsistence farming
• Context: STH-endemic area, resource-limited, no laboratory access
    ↓
═══════════════════════════════════════════════════════════════════════
CRITICAL ISSUE: VOLUNTEER/CONVENIENCE SAMPLING (NOT CONSECUTIVE/RANDOM)
═══════════════════════════════════════════════════════════════════════

RECRUITMENT APPROACH:
1. All village residents given oral presentation about STH
2. Voluntary participation solicited
3. Subjects aged ≥16 who gave consent enrolled
4. Parents consented for assenting children

⚠️ SELECTION BIAS CONCERN:
• Self-selection after STH education presentation
• May enrich for symptomatic or health-conscious individuals
• May underrepresent asymptomatic individuals
• May exclude those unable to attend presentation
• NOT consecutive or random enrollment mechanism

SAMPLE SIZE CALCULATION (Pre-specified):
• Target: 115 participants
• Based on: Hookworm prevalence 33.1% (June 2016 data, Ifanadiana)
• Goal: 90% predicted sensitivity
• Margin of error: 10%
    ↓
ELIGIBLE: Village residents aged ≥16 years
Exclusion criterion: Cognitive impairment (consent capacity)
    ↓
ENROLLED: 113 participants who provided fecal samples
(Achieved 98% of target sample size)

⚠️ Population characteristics not detailed
⚠️ Number screened/approached not reported
⚠️ Reasons for non-participation unknown
    ↓
═══════════════════════════════════════════════════════════════════════
ALL 113 PARTICIPANTS RECEIVED BOTH INDEX TESTS AND REFERENCE STANDARD
═══════════════════════════════════════════════════════════════════════

✓ NO DIFFERENTIAL VERIFICATION
✓ NO PARTIAL VERIFICATION
✓ COMPLETE PATIENT FLOW
✓ NO POST-ENROLLMENT EXCLUSIONS
    ↓
INDEX TEST 1: UVC Smartphone Microscopy
Equipment: USB Video Class smartphone microscope attachment
• Magnification Endoscope (Jiusion Tech)
• Digital Microscope Stand (iTez)
• Total cost: $69.82
Interpretation: Read by trained parasitologists
Blinding: Clinical information and other test results NOT available

INDEX TEST 2: Kankanet AI System
Technology:
• TensorFlow framework
• Single Shot Detection (SSD) meta-architecture
• MobileNet feature extractor
• Android application
• Operates offline (no internet required)

AI Training Dataset:
• Total: 2,078 images
  - 597 microscope images
  - 1,481 UVC images
• Helminth species: A. lumbricoides, T. trichiura, hookworm
• Training/evaluation split: Separate sets
  - Training: 2,078 images
  - Evaluation: 186 images

Model Development:
• Model 1: Trained on microscope images only (n=597)
• Model 2: Trained on all images (n=2,078)
• Stopping criterion: Loss <0.01 (default configuration)
• Data augmentation: Per default protocols

Detection Parameters:
• Confidence threshold: 0.60
  (TensorFlow demo application default)
  ⚠️ NOT explicitly stated as pre-specified before data collection
• Output: Bounding boxes + confidence scores

Interpretation: Automated (inherently blinded to reference)
    ↓
REFERENCE STANDARD: "Modified Gold Standard"
(Composite of THREE microscopy methods)

Method 1: Kato-Katz (KK)
• Sample: Fresh stool
• Intensity: Egg counts × 24
• ⚠️ Hookworm limitation: KK readings NOT obtainable
  (eggs disintegrate during transport to lab)

Method 2: Spontaneous Sedimentation Technique in Tube (SSTT)
• Sample: 10% formalin-preserved stool

Method 3: Merthiolate-Iodine-Formaldehyde (MIF)
• Sample: 10% formalin-preserved stool

Interpretation:
• Performed by: Trained parasitologists
• Institution: Institut Pasteur de Madagascar
• Positive: ANY positive result from 3 methods
• Each sample: 3 slides analyzed

⚠️ BLINDING UNCERTAINTY:
• NOT explicitly stated if parasitologists were blinded to index test results
• Logistics in field setting may make blinding impractical

Rationale for Composite Reference:
• Multiple methods increase sensitivity
• KK established performance:
  - A. lumbricoides: Sens 0.97, Spec 0.96
  - Hookworm: Sens 0.65, Spec 0.94
  - T. trichiura: Sens 0.91, Spec 0.94
    ↓
═══════════════════════════════════════════════════════════════════════
COMPLETE DATA ANALYSIS - ALL 113 PARTICIPANTS INCLUDED
═══════════════════════════════════════════════════════════════════════

Disease Prevalence (by modified gold standard):
• A. lumbricoides: 79/113 (69.9%)
• T. trichiura: 101/113 (89.4%)
• Hookworm: 22/113 (19.5%)

Sample Size Adequacy:
• A. lumbricoides: 79 positive (adequate)
• T. trichiura: 101 positive (adequate)
• Hookworm: 22 positive (LIMITED - may affect precision)

AI Evaluation Dataset:
• 186 evaluation images (separate from training)
• Species distribution not detailed
• Modest sample size for ML validation
    ↓
REPORTED DIAGNOSTIC ACCURACY (Model 2 on microscope images):

A. lumbricoides:
• Sensitivity: 85.7%
• Specificity: 87.5%

T. trichiura:
• Sensitivity: 100.0%
• Specificity: 100.0%

Hookworm:
• Sensitivity: 66.7%
• Specificity: 100.0%
• ⚠️ Based on only 22 positive cases (limited precision)
• ⚠️ KK reference unavailable for hookworm

═══════════════════════════════════════════════════════════════════════
METHODOLOGICAL STRENGTHS
═══════════════════════════════════════════════════════════════════════

✓ Prospective design
✓ Pre-specified sample size calculation
✓ Complete reference standard for all participants (no verification bias)
✓ Complete patient flow (no missing data or post-enrollment exclusions)
✓ Field validation in intended POC setting
✓ Transparent methods reporting
✓ Data availability (OSF database)
✓ Appropriate blinding of index test interpretation
✓ Separate training/evaluation image sets

═══════════════════════════════════════════════════════════════════════
METHODOLOGICAL LIMITATIONS
═══════════════════════════════════════════════════════════════════════

✗ VOLUNTEER/CONVENIENCE SAMPLING (HIGH RISK):
  Selection bias - non-random enrollment after STH presentation
  May create spectrum bias toward symptomatic individuals

✗ THRESHOLD PRE-SPECIFICATION UNCLEAR:
  0.60 confidence threshold described as default
  Not explicitly stated as pre-specified before data collection
  Whether parameters optimized during study not reported

✗ REFERENCE BLINDING UNCERTAIN:
  Not explicitly stated if parasitologists blinded to index results
  May introduce review bias

✗ POTENTIAL OVERFITTING:
  Same population for training images and clinical validation
  Single geographic location limits external validity

✗ LIMITED HOOKWORM DATA:
  Only 22 positive cases
  KK reference unavailable (egg disintegration)

✗ MODEST EVALUATION SET:
  186 images for ML validation (small by ML standards)

✗ SINGLE-SITE STUDY:
  Two villages in one region
  Generalizability uncertain

```

# Phase 3: Risk of Bias and Applicability Judgments

# DOMAIN 1: PATIENT SELECTION

## A. Risk of Bias

**Describe methods of patient selection:**
Enrolled participants from two rural Madagascar villages (Mangevo n=238, Ambinanindranofotaka n=327) near Ranomafana National Park, June 8-18, 2018. All residents given oral presentation about STH; subjects aged ≥16 who gave voluntary consent enrolled (parents consented for assenting children). CONVENIENCE/VOLUNTEER sample, NOT consecutive or random sampling. Sample size calculated at 115 based on hookworm prevalence (33.1%), targeting 90% sensitivity with 10% margin; study enrolled 113. Exclusion: Cognitive impairment only (appropriate). CRITICAL LIMITATION: Self-selection after STH presentation may create spectrum bias - symptomatic or health-conscious individuals more likely to participate, asymptomatic individuals or those unable to attend excluded. Non-random enrollment fundamentally limits generalizability. Page reference: p.4, p.7-8

| Signaling Question | Answer |
| --- | --- |
| ❖ Was a consecutive or random sample enrolled? | NO |
| ❖ Was a case-control design avoided? | YES |
| ❖ Did study avoid inappropriate exclusions? | YES |

**Could the selection of patients have introduced bias?: RISK: HIGH**

**Justification:** This study has high risk of selection bias due to the volunteer/convenience sampling approach. Participants self-selected after a community presentation about STH, which may create spectrum bias - those with symptoms or concerns about helminth infection may be more likely to participate, while asymptomatic individuals or those unable to attend the presentation would be excluded. The study did not implement consecutive or random enrollment from the target population. Sample size (n=113) was adequate for the primary objective based on power calculations, but the non-random selection limits generalizability.

## B. Concerns regarding applicability

**Is there concern that the included patients do not match the review question?: CONCERN: LOW**

**Justification:** The study population closely matches the intended use case for point-of-care STH diagnosis: rural villagers in a resource-limited, STH-endemic setting in Madagascar accessible only by 14 hours of footpaths. The population reflects real-world conditions where such a diagnostic tool would be deployed. Over 95% of households engage in subsistence farming, representing the typical population needing field-deployable STH diagnostics.

# DOMAIN 2: INDEX TEST(S)

## A. Risk of Bias

**Describe the index test:**
Two index tests: (1) UVC smartphone microscopy (Jiusion Tech equipment, $69.82 total) read by parasitologists, (2) Kankanet AI system using TensorFlow with SSD meta-architecture and MobileNet feature extractor. AI trained on 2,078 images (597 microscope, 1,481 UVC) of A. lumbricoides, T. trichiura, hookworm eggs. Two models: Model 1 (microscope only, n=597), Model 2 (all images, n=2,078). Validated on separate 186 images. Detection confidence threshold 0.60 (TensorFlow demo default). Training stopped at loss <0.01 (default configuration). Clinical information NOT available to slide readers. STRENGTH: Automated AI blinded to reference. UNCERTAINTY: 0.60 threshold described as 'per original app' default but not explicitly stated as pre-specified before data collection. Separate training/evaluation sets appropriate, but same population used for both, creating potential overfitting to local characteristics. Whether any parameters optimized during study not explicitly stated. Page reference: p.5-7

| Signaling Question | Answer |
| --- | --- |
| ❖ Interpreted without knowledge of reference? | YES |
| ❖ If threshold used, was it pre-specified? | UNCLEAR |

**Could the conduct of the index test have introduced bias?: RISK: UNCLEAR**

**Justification:** Blinding of the index test interpretation was appropriately maintained. However, there is uncertainty regarding whether the 0.60 confidence threshold was truly pre-specified or could have been influenced by preliminary results. The training and evaluation image sets were separate (2,078 training vs 186 evaluation images), which is methodologically appropriate, but the study used the same population for both image acquisition and clinical validation, creating potential for overfitting to local image characteristics. Additionally, the stopping criterion for model training (loss <0.01) and data augmentation methods followed default protocols, but whether any optimization occurred during the study is not explicitly stated.

## B. Concerns regarding applicability

**Is there concern about the index test applicability?: CONCERN: LOW**

**Justification:** The index test (smartphone-based UVC microscopy with AI interpretation) was specifically designed for point-of-care use in resource-limited settings and was tested in the intended environment - rural Madagascar villages without standard laboratory access. The system operates offline without internet connection, uses commercially available equipment ($69.82 total), and can be operated by non-specialists, matching the intended use case for POC STH diagnosis.

# DOMAIN 3: REFERENCE STANDARD

## A. Risk of Bias

**Describe the reference standard:**
'Modified gold standard' defined as ANY positive result from parasitologist reading of THREE standard microscopy preparations: (1) Kato-Katz (KK) from fresh stool, (2) spontaneous sedimentation technique in tube (SSTT) from 10% formalin-preserved stool, (3) Merthiolate-Iodine-Formaldehyde (MIF) from 10% formalin-preserved stool. Performed by trained parasitologists at Institut Pasteur de Madagascar. Each sample produced three slides. Intensity measured by KK egg counts × 24. LIMITATION: Hookworm KK readings NOT obtainable due to egg disintegration during transport. Composite reference increases sensitivity. KK established performance (sens/spec): A. lumbricoides 0.97/0.96, hookworm 0.65/0.94, T. trichiura 0.91/0.94. BLINDING UNCERTAINTY: Paper states clinical information not available to slide readers, but appears to refer to index test readers. NOT explicitly stated whether parasitologists reading standard microscopy were blinded to UVC/Kankanet results. Field logistics may make blinding impractical. Page reference: p.4, p.8

| Signaling Question | Answer |
| --- | --- |
| ❖ Is reference standard likely to correctly classify? | YES |
| ❖ Interpreted without knowledge of index test? | UNCLEAR |

**Could the reference standard have introduced bias?: RISK: UNCLEAR**

**Justification:** The composite reference standard combining KK, SSTT, and MIF methods with expert parasitologist interpretation is appropriate for STH diagnosis and likely to correctly classify disease status. Using multiple methods increases sensitivity as intended. However, the paper does not explicitly state whether parasitologists reading standard microscopy were blinded to UVC/Kankanet results. The statement about clinical information not being available appears to refer to index test readers. Given the field setting and logistics of processing samples at Institut Pasteur de Madagascar, blinding may have been impractical, creating potential for review bias.

## B. Concerns regarding applicability

**Is there concern about reference standard applicability?: CONCERN: LOW**

**Justification:** The composite reference standard (KK, SSTT, MIF) with expert parasitologist interpretation represents appropriate and established methods for STH diagnosis. These are the accepted reference standards for helminth detection. The target conditions (A. lumbricoides, T. trichiura, hookworm) are clearly defined. The only limitation is the hookworm KK readings being unavailable due to egg disintegration during transport, but this is a practical constraint rather than an applicability issue.

# DOMAIN 4: FLOW AND TIMING

## A. Risk of Bias

**Patient flow:**
All 113 enrolled participants received both index tests (UVC microscopy and Kankanet AI) and the complete reference standard (composite of KK, SSTT, MIF methods). Complete patient flow with NO missing data or post-enrollment exclusions. All 113 included in analysis. Time interval appropriate - samples collected in field (June 8-18, 2018) and processed by parasitologists at Institut Pasteur de Madagascar. Same samples used for both index tests and reference standard, eliminating interval-related disease progression issues. NO differential verification (all received same composite reference) and NO partial verification (all received complete reference). Excellent study completion with 100% of enrolled participants analyzed. Page reference: p.4, p.8

| Signaling Question | Answer |
| --- | --- |
| ❖ Appropriate interval between tests? | YES |
| ❖ Did all patients receive reference standard? | YES |
| ❖ Did patients receive same reference standard? | YES |
| ❖ Were all patients included in analysis? | YES |

**Could the patient flow have introduced bias?: RISK: LOW**

**Justification:** LOW risk - this domain demonstrates excellent methodological quality. All 113 enrolled participants received both the index tests and the complete reference standard. There was no differential verification (all received the same composite reference of KK, SSTT, and MIF) and no partial verification (all received the complete reference standard). There were no missing data or post-enrollment exclusions, with 100% of enrolled participants included in the analysis. The time interval was appropriate as samples were processed systematically. This represents one of the study's major strengths.

# OVERALL ASSESSMENT SUMMARY

**HIGH RISK OF BIAS - VOLUNTEER SAMPLING**

| Domain | Signaling Questions | Risk of Bias | Applicability |
| --- | --- | --- | --- |
| D1: Patient Selection | 1.1 No 1.2 Yes 1.3 Yes | HIGH | LOW |
| D2: Index Test | 2.1 Yes 2.2 Unclear | UNCLEAR | LOW |
| D3: Reference Standard | 3.1 Yes 3.2 Unclear | UNCLEAR | LOW |
| D4: Flow and Timing | 4.1 Yes 4.2 Yes 4.3 Yes 4.4 Yes | LOW | N/A |

**Overall Risk of Bias: HIGH**

**Justification:** The study has 1 HIGH risk domain (Patient Selection due to volunteer/convenience sampling) and 2 UNCLEAR risk domains (Index Test threshold pre-specification uncertainty; Reference Standard blinding uncertainty). The non-random enrollment is a fundamental methodological limitation that affects the generalizability of diagnostic accuracy estimates. Participants self-selected after a community presentation about STH, which may create spectrum bias toward symptomatic or health-conscious individuals. However, the study demonstrates excellent flow and timing (Domain 4 LOW) with complete reference standard applied to all participants and no verification bias.

**Overall Applicability Concern: LOW**

**Justification:** Despite methodological limitations, this study has low applicability concerns for POC STH diagnosis. The population (rural Malagasy villagers in STH-endemic area), index test (smartphone-based UVC microscopy with offline AI), and reference standard (established parasitological methods) closely match the intended use case. The study was conducted in real-world field conditions representative of where such technology would be deployed - extremely remote setting (14 hours by footpaths) with no laboratory access.

## Critical Biases and Limitations

**1. SELECTION BIAS / VOLUNTEER BIAS (SEVERITY: HIGH):**
Participants self-selected after community presentation about STH. This convenience/volunteer sampling (NOT consecutive or random) may create spectrum bias. Symptomatic or health-conscious individuals more likely to participate; asymptomatic individuals or those unable to attend presentation excluded. Fundamentally limits generalizability of diagnostic accuracy estimates.

**2. THRESHOLD PRE-SPECIFICATION UNCERTAINTY (SEVERITY: UNCLEAR):**
Detection confidence threshold (0.60) described as TensorFlow demo application default, but NOT explicitly stated as pre-specified before data collection. Training stopping criterion (loss <0.01) and augmentation methods followed defaults, but whether any parameters were optimized during study not reported. Creates uncertainty about potential optimistic bias from post-hoc threshold selection.

**3. POTENTIAL REVIEW BIAS (SEVERITY: UNCLEAR):**
Insufficient documentation of whether reference standard parasitologists were blinded to index test results. Paper states clinical information not available to slide readers, but appears to refer to index test readers. Field logistics may make blinding impractical. Could introduce review bias if parasitologists aware of index test findings.

**4. LIMITED GENERALIZABILITY (SEVERITY: MODERATE):**
Single geographic location (two villages in one region of Madagascar). Same population used for training images and clinical validation creates potential overfitting to local characteristics. Performance in other settings uncertain. External validation needed.

## Significant Methodological Strengths

**Despite selection bias, this study has important strengths:

✓ Prospective design:** Pre-specified sample size calculation before enrollment
**✓ Complete verification:** All 113 participants received complete reference standard (no differential/partial verification)
**✓ Complete patient flow:** 100% of enrolled analyzed, no missing data, no post-enrollment exclusions
**✓ Field validation:** Real-world POC setting in intended environment (extremely remote, no lab access)
**✓ Appropriate blinding:** Index test interpreters blinded to clinical information and reference results
**✓ Composite reference:** Three microscopy methods (KK, SSTT, MIF) increase sensitivity
**✓ Separate datasets:** Training (2,078 images) and evaluation (186 images) sets appropriately separated
**✓ Transparent reporting:** Data availability (OSF database), hyperparameters reported
**✓ Low-cost equipment:** $69.82 total, commercially available, offline operation

## Reported Diagnostic Accuracy

**Model 2 Performance (trained on all 2,078 images, tested on microscope images):

A. lumbricoides (79/113 positive = 69.9%):**
• Sensitivity: 85.7%
• Specificity: 87.5%
• Adequate sample size

**T. trichiura (101/113 positive = 89.4%):**
• Sensitivity: 100.0%
• Specificity: 100.0%
• Perfect performance
• Adequate sample size

**Hookworm (22/113 positive = 19.5%):**
• Sensitivity: 66.7%
• Specificity: 100.0%
• LIMITED: Only 22 positive cases (affects precision)
• KK reference unavailable (egg disintegration)

**Sample Sizes:**
• Total participants: 113
• Training images: 2,078
• Evaluation images: 186 (modest for ML validation)

**INTERPRETATION:** Performance estimates should be interpreted cautiously due to volunteer sampling bias. Actual performance in unselected populations may differ. Hookworm results particularly limited by small positive sample (n=22) and missing KK reference.

## Study Value and Context

**What this study demonstrates:

✓ Proof-of-concept:** AI-based smartphone microscopy feasible for STH detection in extremely remote field settings
**✓ Low-cost solution:** $69.82 equipment, commercially available, offline operation
**✓ Real-world validation:** Tested in intended environment (no lab access, 14-hour footpath access)
**✓ Non-specialist use:** Designed for operation by community health workers
**✓ Complete verification:** No verification bias - all received gold standard

**Important limitations:

✗ Volunteer sampling:** Non-random enrollment creates selection bias, limits generalizability
**✗ Single site:** Two villages in one Madagascar region, external validation needed
**✗ Threshold uncertainty:** Pre-specification not explicitly documented
**✗ Blinding unclear:** Reference standard blinding not documented
**✗ Hookworm limited:** Only 22 positive cases, KK unavailable
**✗ Modest evaluation:** 186 images for ML validation (small by ML standards)
**✗ Potential overfitting:** Same population for training and validation

QUADAS-2

**Yu et al. 2023**
Patient-level performance evaluation of a smartphone-based malaria diagnostic application *Study ID: Yu_2023 | Assessment Date: 2025-11-28*

____________________________________________________________________________________________________

# Phase 1: State the Review Question

*Patients (setting, intended use of index test, presentation, prior testing):*

CASE-CONTROL STUDY organized by FIND. Enrolled 190 patients total from two primary hospitals in Sudan (Alsororab [SOR] site, Gezira Slanj [GS] site), 40 and 50 km north of Khartoum, October 2020-March 2021. Setting: Point-of-care - rural malaria-endemic primary hospitals in resource-limited area. Intended use: Malaria Screener Android app for automated thick blood smear analysis as POC diagnostic aid. Presentation: Febrile patients presenting with suspected malaria. Sample size: Pre-calculated to recruit ~100 malaria-positive (cases) by on-site microscopy and ~90 malaria-negative (controls) to achieve 95% power with 95% CI ±10%. Enrollment: 'Consecutively until reaching calculated numbers (190 total, 95 from each site)'. Inclusion: Age ≥5 years. Exclusions: Severe disease/comorbidities (CNS or cardiovascular per WHO guidelines), anti-malarial treatment in past 4 weeks (appropriate). Results by microscopy: 100 positive (52.6%), 90 negative (47.4%); Among positives: 61 P. falciparum, 38 P. vivax, 1 mixed infection. CRITICAL FLAW - CASE-CONTROL DESIGN: Artificially selected predetermined numbers of diseased and non-diseased patients rather than consecutive presenting patients. Creates SPECTRUM BIAS by potentially including clearly positive cases and clearly negative controls while excluding borderline/difficult cases encountered in real practice. Artificially inflates diagnostic accuracy estimates. ADDITIONAL FLAW - SUBSTANTIAL EXCLUSIONS: Only 85/190 (44.7%) analyzed by Malaria Screener - excluded all P. vivax cases (38% of positives) and used only SOR site for testing. Limits generalizability.

*Index test(s):*

Malaria Screener version 1.6.6 (Android mobile application, Google Play Store). Semi-automated workflow: Samsung Galaxy A10 smartphone mounted onto Olympus CX23 microscope eyepiece, microscopist identifies suitable fields of view (FoVs) from Giemsa-stained thick blood smears, captures images, app processes automatically using machine learning and computer vision algorithms. Workflow continues until user-specified WBC threshold met (minimum 1000 WBC count used). Deep learning classifier provides confidence scores for parasite candidates; image-level scores computed as mean of object-level scores; patient-level scores computed as mean of image-level scores. Total images: 2,944 thick smear (15.5/patient average), 875 thin smear (4.6/patient). CRITICAL FLAW - THRESHOLD NOT PRE-SPECIFIED: 'Slides from GS site were used to fine-tune system parameters. Fine-tuning mainly consisted of analysing ROC to find confidence threshold leading to highest patient-level accuracy.' Threshold optimized on GS site data (n=95), then tested on SOR site data (n=85). Post-study experiment tried 'different patient-level classification method' using 'threshold based on number of parasite candidates' which dramatically improved results (from 74.1% to 91.8% accuracy). Introduces risk of overfitting and optimistically biased accuracy. STRENGTH: AI blinded to reference (automated processing). Equipment: Standard Android smartphone (~$150) + affordable microscope adapter ($10-100), accessible for resource-limited settings. Post-study also evaluated PlasmodiumVF-Net (PVF-Net), newly developed algorithm trained on Bangladesh data.

*Reference standard and target condition:*

Two reference standards: (1) Expert microscopy, (2) Nested PCR. Microscopy: Blood smears stained with 3% Giemsa for 45 minutes per WHO standard. Reading protocol: Site microscopists at each hospital examined first, then verified by second microscopist at University of Khartoum Institute of Endemic Diseases. Obare method calculator determined agreement; third expert reading performed when discrepancy. 'All microscopists were WHO Level 1 expert microscopists.' INTER-OBSERVER VARIABILITY: Among 100 positive reads, first two microscopists reached consensus for only 27 patients (27%), discordant diagnoses for 73 patients (73%) requiring third reader arbitration per Obare calculator. PCR: Nested PCR performed on dried blood spots using QIAamp DNA extraction kit, published primers (Snounou et al.), positive controls (P. falciparum 3D7 strain, P. vivax Sudanese isolate) plus negative controls. PCR confirmed microscopy diagnosis for 178/190 patients (93.7%), detected parasites in 11 microscopy-negative patients. Compared to PCR, WHO Level 1 microscopists achieved 94.2% accuracy. STRENGTH: All 190 patients received both microscopy AND PCR (no differential verification). High-quality reference with WHO Level 1 experts and multi-reader protocol. TRANSPARENCY: Study openly reports high inter-observer variability. Target condition: Malaria (Plasmodium species, primarily P. falciparum and P. vivax in Sudan).

# Phase 2: Draw a Flow Diagram for the Primary Study

```

PATIENT FLOW DIAGRAM - CASE-CONTROL STUDY

STUDY PERIOD: October 2020 - March 2021 (6 months)
STUDY DESIGN: Case-control organized by FIND
SETTING: Two primary hospitals in rural Sudan
• Alsororab (SOR) site: 40 km north of Khartoum
• Gezira Slanj (GS) site: 50 km north of Khartoum
• Malaria-endemic area, resource-limited setting
    ↓
═══════════════════════════════════════════════════════════════════════
CRITICAL FLAW: CASE-CONTROL DESIGN (NOT CONSECUTIVE COHORT)
═══════════════════════════════════════════════════════════════════════

SAMPLE SIZE CALCULATION (Pre-specified):
• Target: ~100 malaria-positive (cases) by on-site microscopy
         ~90 malaria-negative (controls)
• Goal: 95% power, 95% CI ±10%

ENROLLMENT APPROACH:
"Patients were enrolled consecutively until reaching the
calculated numbers (190 patients in total, 95 from each site)"

⚠️ SPECTRUM BIAS CONCERN:
• Predetermined case/control ratio (NOT natural presentation prevalence)
• May include "clearly positive" cases and "clearly negative" controls
• May exclude borderline or difficult-to-diagnose cases
• Artificially inflates diagnostic accuracy vs real clinical practice
• Does NOT represent consecutive presenting patients
    ↓
ELIGIBLE: Febrile patients presenting with suspected malaria
Inclusion: Age ≥5 years
Exclusion:
• Severe disease or comorbidities (CNS/cardiovascular per WHO)
• Anti-malarial treatment in past 4 weeks
    ↓
ENROLLED: 190 patients
• SOR site: 95 patients
• GS site: 95 patients
    ↓
═══════════════════════════════════════════════════════════════════════
ALL 190 PATIENTS RECEIVED BOTH REFERENCE STANDARDS
═══════════════════════════════════════════════════════════════════════

✓ NO DIFFERENTIAL VERIFICATION
✓ NO PARTIAL VERIFICATION
✓ Complete reference standard for all patients

REFERENCE STANDARD 1: Expert Microscopy

Protocol:
1. Blood smears stained: 3% Giemsa for 45 minutes (WHO standard)
2. First reading: Site microscopist at hospital
3. Second reading: Microscopist at University of Khartoum
                   Institute of Endemic Diseases
4. Agreement assessment: Obare method calculator
5. Third reading: When discrepancy detected

All microscopists: WHO Level 1 expert level

⚠️ HIGH INTER-OBSERVER VARIABILITY:
Among 100 microscopy-positive patients:
• Consensus between first two readers: 27/100 (27%)
• Discordant diagnoses: 73/100 (73%)
• Required third reader arbitration: 73 patients

REFERENCE STANDARD 2: Nested PCR

Sample: Dried blood spots
Extraction: QIAamp DNA extraction kit
Method: Published primers (Snounou et al.)
Controls:
• Positive: P. falciparum (3D7 strain)
           P. vivax (Sudanese isolate)
• Negative controls included

REFERENCE STANDARD COMPARISON:
• PCR confirmed microscopy: 178/190 patients (93.7%)
• PCR detected parasites in microscopy-negative: 11 patients
• WHO Level 1 microscopist accuracy vs PCR: 94.2%
    ↓
DISEASE STATUS BY EXPERT MICROSCOPY (190 patients):

POSITIVE: 100 patients (52.6%)
├─→ P. falciparum: 61 patients (61%)
├─→ P. vivax: 38 patients (38%)
└─→ Mixed (P. falciparum + P. vivax): 1 patient (1%)

NEGATIVE: 90 patients (47.4%)
    ↓
═══════════════════════════════════════════════════════════════════════
CRITICAL: SUBSTANTIAL EXCLUSIONS FOR INDEX TEST ANALYSIS
═══════════════════════════════════════════════════════════════════════

ENROLLED: 190 patients at 2 sites

EXCLUSION CASCADE:

EXCLUSION 1: Site-based split
├─→ GS site (n=95): Used for THRESHOLD CALIBRATION
└─→ SOR site (n=95): Available for testing

⚠️ THRESHOLD OPTIMIZATION:
"Slides from GS site were used to fine-tune system parameters.
Fine-tuning mainly consisted of analysing ROC to find confidence
threshold leading to highest patient-level accuracy"

EXCLUSION 2: Species-based exclusion (from SOR site)
├─→ P. vivax cases: EXCLUDED (species-specific limitation)
│   → This excluded 38% of positive cases from enrolled cohort
└─→ Mixed infections: EXCLUDED

FINAL TEST SET (Malaria Screener): 85/190 (44.7%)
• From SOR site only
• P. falciparum positive: 40 patients
• Negative: 45 patients

EXCLUDED FROM ANALYSIS: 105/190 (55.3%)
├─→ GS site (entire site): 95 patients (used for calibration)
├─→ P. vivax cases: ~38 patients
└─→ Mixed infections: ~1 patient

⚠️ CONSEQUENCES:
• Only 44.7% of enrolled patients analyzed
• Excludes major disease species (P. vivax = 38% of positives)
• Uses one site only for calibration, other site for testing
• Creates SELECTION EFFECTS and limits generalizability
• Does NOT represent full clinical spectrum in endemic region
    ↓
═══════════════════════════════════════════════════════════════════════
INDEX TEST: MALARIA SCREENER (n=85 from SOR site)
═══════════════════════════════════════════════════════════════════════

Equipment:
• Smartphone: Samsung Galaxy A10 (~$150)
• Microscope: Olympus CX23 (standard light microscope)
• Adapter: Smartphone mount on eyepiece ($10-100)
• Total: Accessible for resource-limited settings

Software:
• Malaria Screener v1.6.6
• Android mobile application
• Downloaded from Google Play Store

Workflow (Semi-automated):
1. Microscopist prepares Giemsa-stained thick blood smear
2. Microscopist identifies suitable fields of view (FoVs)
3. Microscopist captures images using smartphone
4. App processes images automatically:
   • Machine learning algorithms
   • Computer vision analysis
   • Deep learning classifier
5. Continues until WBC threshold met (minimum 1000 WBCs)

Image Analysis:
• Deep learning classifier: Confidence scores per parasite candidate
• Image-level score: Mean of object-level scores
• Patient-level score: Mean of image-level scores

Images Collected (all 190 patients):
• Thick smear: 2,944 images (15.5 per patient average)
• Thin smear: 875 images (4.6 per patient average)

Threshold Determination (⚠️ NOT PRE-SPECIFIED):
• GS site data (n=95): ROC analysis to find threshold
                       maximizing patient-level accuracy
• Threshold: Optimized on calibration set, applied to test set
• Risk: Overfitting to calibration data

Post-Study Exploration:
• Alternative patient-level classification method tried
• New threshold: Based on number of parasite candidates
• Result: Improved from 74.1% to 91.8% accuracy
• ⚠️ Indicates performance sensitive to threshold selection
    ↓
═══════════════════════════════════════════════════════════════════════
REPORTED PERFORMANCE (n=85 from SOR site only)
═══════════════════════════════════════════════════════════════════════

Population:
• P. falciparum positive: 40 patients
• Negative: 45 patients
• Species: P. falciparum only (P. vivax excluded)

Malaria Screener Performance (Primary Analysis):
• Sensitivity: 100% (95% CI: 91.2-100)
  → Perfect sensitivity BUT based on only 40 positive cases
  → Wide CI lower bound (91.2%) indicates uncertainty

• Specificity: 51.1% (95% CI: 35.8-66.3)
  → LOW specificity = HIGH false positive rate
  → Wide CI indicates limited sample size
  → Likely due to parasite-like staining artifacts

• Overall Accuracy: 74.1%

Post-Study Experiment (Alternative Threshold):
• Method: Threshold based on parasite candidate counts (not scores)
• Sensitivity: 100%
• Specificity: 84.4%
• Overall Accuracy: 91.8%

⚠️ Post-study improvement suggests:
• Original performance dependent on threshold selection
• Exploratory rather than confirmatory analysis
• Results should be interpreted cautiously

PlasmodiumVF-Net (PVF-Net) - Additional Post-Study Evaluation:
• Newly developed algorithm
• Trained on Bangladesh data
• Evaluated on same Sudan dataset
• Performance details in post-study analysis

═══════════════════════════════════════════════════════════════════════
SUMMARY OF METHODOLOGICAL ISSUES
═══════════════════════════════════════════════════════════════════════

1. CASE-CONTROL DESIGN (HIGH RISK):
   • Predetermined case/control ratio
   • Spectrum bias - may overestimate accuracy
   • Not consecutive presenting patients

2. THRESHOLD OPTIMIZATION (HIGH RISK):
   • NOT pre-specified
   • Optimized on GS site data using ROC analysis
   • Risk of overfitting to calibration data
   • Post-study experiments show sensitivity to threshold

3. SUBSTANTIAL EXCLUSIONS (HIGH RISK):
   • Only 85/190 (44.7%) analyzed
   • Excluded entire GS site (used for calibration)
   • Excluded all P. vivax cases (38% of positives)
   • Selection effects limit generalizability

4. SMALL TEST SET:
   • Only 40 P. falciparum positive cases
   • Wide confidence intervals
   • Limited precision of estimates

5. HIGH FALSE POSITIVE RATE:
   • Specificity 51.1% (main analysis)
   • 49% of negatives incorrectly flagged as positive
   • Study attributes to staining artifacts

```

# Phase 3: Risk of Bias and Applicability Judgments

# DOMAIN 1: PATIENT SELECTION

## A. Risk of Bias

**Describe methods of patient selection:**
Case-control study organized by FIND. Enrolled 190 patients from two primary hospitals in Sudan (Alsororab [SOR], Gezira Slanj [GS]), 40 and 50 km north of Khartoum, October 2020-March 2021. Sample size calculated to recruit ~100 malaria-positive (cases) by on-site microscopy and ~90 malaria-negative (controls) to achieve 95% power with 95% CI ±10%. Patients 'enrolled consecutively until reaching calculated numbers (190 total, 95 from each site)'. Inclusion: Age ≥5 years. Exclusions: Severe disease/comorbidities (CNS/cardiovascular per WHO), anti-malarial treatment in past 4 weeks (appropriate). Results: 100 positive (61 P. falciparum, 38 P. vivax, 1 mixed), 90 negative. CRITICAL FLAW: Case-control design artificially selected predetermined numbers of diseased/non-diseased rather than consecutive presenting patients. Creates SPECTRUM BIAS by potentially including clearly positive cases and clearly negative controls while excluding borderline/difficult cases, artificially inflating diagnostic accuracy vs real clinical practice. Page reference: p.2-3, p.5

| Signaling Question | Answer |
| --- | --- |
| ❖ Was a consecutive or random sample enrolled? | YES (but case-control) |
| ❖ Was a case-control design avoided? | NO |
| ❖ Did study avoid inappropriate exclusions? | YES |

**Could the selection of patients have introduced bias?: RISK: HIGH**

**Justification:** The study employed a case-control design, which introduces spectrum bias by artificially selecting predetermined numbers of diseased and non-diseased patients rather than evaluating consecutive presenting patients with suspected malaria. The study explicitly recruited ~100 positive and ~90 negative patients based on initial on-site microscopy, rather than enrolling an unselected cohort of febrile patients presenting for evaluation. Case-control designs in diagnostic accuracy studies tend to overestimate test performance because they often include clearly positive cases and clearly negative controls, potentially excluding borderline or difficult-to-diagnose cases that would be encountered in real clinical practice. This artificially inflates diagnostic accuracy estimates compared to what would be observed in routine clinical use.

## B. Concerns regarding applicability

**Is there concern that the included patients do not match the review question?: CONCERN: LOW**

**Justification:** The study population is highly applicable to the review question of AI in point-of-care imaging. The study was conducted at two primary hospitals in rural malaria-endemic areas near Khartoum, Sudan, representing a resource-limited field setting where point-of-care diagnostics are most needed. Patients were febrile individuals presenting with suspected malaria, which matches the intended use population for the Malaria Screener application. The setting (rural hospitals, 40-50 km from urban center) represents realistic deployment conditions for smartphone-based malaria diagnostics.

# DOMAIN 2: INDEX TEST(S)

## A. Risk of Bias

**Describe the index test:**
Malaria Screener v1.6.6 (Android app, Google Play Store). Semi-automated: Samsung Galaxy A10 smartphone mounted on Olympus CX23 microscope eyepiece, microscopist identifies FoVs from Giemsa-stained thick blood smears, captures images, app processes automatically using ML/CV algorithms. Continues until minimum 1000 WBC count. Deep learning classifier provides confidence scores per parasite candidate; image-level scores = mean of object-level scores; patient-level scores = mean of image-level scores. Images: 2,944 thick (15.5/patient), 875 thin (4.6/patient). CRITICAL FLAW - THRESHOLD NOT PRE-SPECIFIED: 'Slides from GS site were used to fine-tune system parameters. Fine-tuning mainly consisted of analysing ROC to find confidence threshold leading to highest patient-level accuracy.' Threshold optimized on GS site (n=95), tested on SOR site (n=85). Post-study experiment with 'different patient-level classification method' using 'threshold based on parasite candidates' dramatically improved results (74.1% to 91.8% accuracy). Introduces overfitting risk and optimistic bias. STRENGTH: AI blinded to reference (automated). Equipment accessible ($150 smartphone + $10-100 adapter). Post-study also evaluated PVF-Net (trained on Bangladesh data). Page reference: p.2-4, p.5-6

| Signaling Question | Answer |
| --- | --- |
| ❖ Interpreted without knowledge of reference? | YES |
| ❖ If threshold used, was it pre-specified? | NO |

**Could the conduct of the index test have introduced bias?: RISK: HIGH**

**Justification:** Although the AI system was blinded to reference standard results (2.1 = Yes), the classification threshold was NOT pre-specified and was instead optimized on part of the study data (2.2 = No). The threshold was determined by analyzing ROC curves on GS site data to maximize patient-level accuracy, then applied to SOR site data for evaluation. This introduces risk of overfitting to the calibration dataset and may produce optimistically biased accuracy estimates. Furthermore, the post-study experiment introduced an additional ad-hoc patient-level classification method using parasite count thresholds that dramatically improved performance (from 74.1% to 91.8% accuracy), indicating the initially reported performance was dependent on threshold selection methodology. While using a separate calibration set (GS site) from the test set (SOR site) partially mitigates overfitting concerns, the threshold was still data-derived rather than pre-specified based on prior validation studies.

## B. Concerns regarding applicability

**Is there concern about the index test applicability?: CONCERN: LOW**

**Justification:** The index test setup is directly applicable to point-of-care imaging in resource-limited settings. The application runs on a standard Android smartphone (Samsung Galaxy A10, ~$150) with an affordable microscope adapter ($10-100), making it accessible in low-resource settings. The semi-automated workflow where microscopists select FoVs and the app analyzes them represents a realistic deployment scenario for field use. The system was tested on thick blood smears prepared with standard WHO Giemsa staining protocols, which is the standard approach in malaria-endemic regions.

# DOMAIN 3: REFERENCE STANDARD

## A. Risk of Bias

**Describe the reference standard:**
Two reference standards: (1) Expert microscopy, (2) Nested PCR. Microscopy: Blood smears stained 3% Giemsa 45 min per WHO standard. Reading: Site microscopist first, then verified by second microscopist at University of Khartoum Institute of Endemic Diseases. Obare method calculator determined agreement; third expert reading when discrepancy. 'All microscopists were WHO Level 1 expert microscopists.' INTER-OBSERVER VARIABILITY: Among 100 positive reads, first two microscopists reached consensus for only 27 patients (27%), discordant diagnoses for 73 patients (73%) requiring third reader arbitration. PCR: Nested PCR on dried blood spots using QIAamp DNA extraction, published primers (Snounou et al.), positive controls (P. falciparum 3D7, P. vivax Sudanese isolate) plus negative controls. PCR confirmed microscopy for 178/190 patients (93.7%), detected parasites in 11 microscopy-negative patients. WHO Level 1 microscopists achieved 94.2% accuracy vs PCR. STRENGTH: All 190 patients received BOTH microscopy AND PCR (no differential verification). High-quality multi-reader protocol. Transparent reporting of inter-observer variability. Page reference: p.3-4, p.7

| Signaling Question | Answer |
| --- | --- |
| ❖ Is reference standard likely to correctly classify? | YES |
| ❖ Interpreted without knowledge of index test? | YES |

**Could the reference standard have introduced bias?: RISK: LOW**

**Justification:** The composite reference standard combining expert microscopy (WHO Level 1 microscopists with multi-reader protocol) and nested PCR is appropriate and likely to correctly classify malaria. The multi-reader protocol with Obare method calculator and third-reader arbitration for discordant cases enhances reliability. PCR confirmation for all 190 patients provides molecular verification. The study transparently reports the high inter-observer variability among microscopists (73% discordance rate), which is valuable for understanding reference standard limitations. There is no indication that reference standard readers had knowledge of index test results. The reference standard is robust and appropriate for this setting.

## B. Concerns regarding applicability

**Is there concern about reference standard applicability?: CONCERN: LOW**

**Justification:** The reference standards (expert microscopy per WHO protocols and nested PCR) are appropriate and applicable for malaria diagnosis. WHO Level 1 microscopy represents the established field standard in endemic regions, while PCR provides molecular confirmation. The target condition (malaria caused by Plasmodium species) is clearly defined. The methods match those used in clinical practice and research in malaria-endemic areas.

# DOMAIN 4: FLOW AND TIMING

## A. Risk of Bias

**Patient flow:**
All 190 enrolled patients received both reference standards (expert microscopy and PCR) - no differential or partial verification. Time interval appropriate (same blood samples used for all tests). HOWEVER, substantial exclusions for Malaria Screener analysis: Only 85/190 patients (44.7%) included. GS site (n=95) used entirely for threshold calibration, excluded from test set. From SOR site (n=95), only P. falciparum cases analyzed - excluded all P. vivax cases (representing 38% of positive cases in enrolled cohort) and mixed infections. Final test set: 40 P. falciparum positive + 45 negative from SOR site only. Creates SELECTION EFFECTS: Analyzed population does NOT represent full clinical spectrum in endemic region where P. vivax represents 38% of malaria. Using one site only for calibration and other for testing may introduce site-specific biases. Fundamentally limits generalizability. Page reference: p.3-5, p.7

| Signaling Question | Answer |
| --- | --- |
| ❖ Appropriate interval between tests? | YES |
| ❖ Did all patients receive reference standard? | YES |
| ❖ Did patients receive same reference standard? | YES |
| ❖ Were all patients included in analysis? | NO |

**Could the patient flow have introduced bias?: RISK: HIGH**

**Justification:** HIGH risk due to substantial exclusions. Only 85/190 patients (44.7%) were included in Malaria Screener analysis. The exclusion of P. vivax cases (representing 38% of positive cases in this endemic region) and use of one site only for calibration limits generalizability and introduces selection effects. The GS site (n=95) was used entirely for threshold optimization and excluded from the test set, while only P. falciparum cases from SOR site were analyzed. This means the evaluated population does not represent the full clinical spectrum of malaria in Sudan where both P. falciparum and P. vivax are endemic. The analyzed sample (40 P. falciparum positives, 45 negatives) is also small, limiting precision of performance estimates.

# OVERALL ASSESSMENT SUMMARY

**HIGH RISK OF BIAS - MULTIPLE FLAWS**

| Domain | Signaling Questions | Risk of Bias | Applicability |
| --- | --- | --- | --- |
| D1: Patient Selection | 1.1 Yes (case-control) 1.2 No 1.3 Yes | HIGH | LOW |
| D2: Index Test | 2.1 Yes 2.2 No | HIGH | LOW |
| D3: Reference Standard | 3.1 Yes 3.2 Yes | LOW | LOW |
| D4: Flow and Timing | 4.1 Yes 4.2 Yes 4.3 Yes 4.4 No | HIGH | N/A |

**Overall Risk of Bias: HIGH**

**Justification:** This study has HIGH overall risk of bias due to methodological concerns in three of four domains: (1) Domain 1 (Patient Selection): HIGH - Case-control design artificially selecting predetermined numbers of malaria-positive cases and negative controls introduces spectrum bias that may overestimate diagnostic accuracy. (2) Domain 2 (Index Test): HIGH - The classification threshold was optimized on part of the study data (GS site) rather than pre-specified, risking overfitted performance estimates. The post-study experiment with alternative thresholding further suggests performance is sensitive to threshold selection. (3) Domain 4 (Flow and Timing): HIGH - Only 85/190 patients (44.7%) were included in Malaria Screener analysis. The exclusion of P. vivax cases (representing 38% of positive cases in this endemic region) and use of one site only for calibration limits generalizability and introduces selection effects. Additional concerns include spectrum bias from case-control design, small sample size (only 40 P. falciparum positives tested), and post-hoc analyses suggesting exploratory rather than confirmatory findings.

**Overall Applicability Concern: LOW**

**Justification:** Despite the methodological limitations, this study has high applicability to the review question. The study was conducted in the intended deployment setting (rural malaria-endemic areas in Sudan), used affordable equipment accessible in resource-limited settings (standard smartphone, basic microscope), evaluated a point-of-care imaging workflow, and compared against appropriate reference standards (WHO Level 1 microscopy, PCR). The patient population (febrile patients presenting with suspected malaria), healthcare setting (primary hospitals in endemic areas), and test conditions closely match the intended clinical use of AI-assisted malaria screening.

## Critical Biases and Limitations

**1. SPECTRUM BIAS FROM CASE-CONTROL DESIGN (SEVERITY: HIGH):**
Study artificially selected predetermined numbers of malaria-positive cases (~100) and negative controls (~90) rather than consecutive presenting patients. Case-control designs tend to include clearly positive cases and clearly negative controls, potentially excluding borderline or difficult-to-diagnose cases encountered in real practice. Artificially inflates diagnostic accuracy estimates compared to routine clinical use.

**2. THRESHOLD OPTIMIZATION BIAS (SEVERITY: HIGH):**
Threshold NOT pre-specified. GS site data (n=95) used to analyze ROC curves and find confidence threshold maximizing patient-level accuracy. Threshold then applied to SOR site (n=85). Introduces overfitting risk to calibration data. Post-study experiment with alternative classification method (parasite count threshold) dramatically improved results from 74.1% to 91.8% accuracy, indicating initial performance dependent on threshold selection. While separate calibration/test sets partially mitigate concerns, threshold was data-derived not externally pre-specified.

**3. SUBSTANTIAL EXCLUSIONS / SELECTION EFFECTS (SEVERITY: HIGH):**
Only 85/190 (44.7%) analyzed by Malaria Screener. Excluded: (1) Entire GS site (n=95) used for calibration, (2) All P. vivax cases representing 38% of positive cases in enrolled cohort, (3) Mixed infections. Final test set: 40 P. falciparum + 45 negative from SOR site only. Analyzed population does NOT represent full clinical spectrum in endemic region where P. vivax is major disease burden (38% of positives). Limits generalizability.

**4. SMALL SAMPLE SIZE (SEVERITY: MODERATE):**
Only 40 P. falciparum positive cases in test set. While sensitivity 100% (95% CI: 91.2-100) appears excellent, wide CI lower bound (91.2%) indicates substantial uncertainty. Specificity 51.1% (95% CI: 35.8-66.3) has even wider CI reflecting limited sample. Limits precision of estimates.

## Significant Methodological Strengths

**Despite multiple high-risk biases, this study has important strengths:

✓ First patient-level evaluation:** First study to evaluate smartphone-based malaria diagnostic at patient level (not just image level)
**✓ Field environment:** Conducted in natural field setting in endemic region (rural Sudan)
**✓ High-quality reference:** WHO Level 1 microscopists with multi-reader protocol + PCR verification
**✓ Complete reference verification:** All 190 patients received both microscopy AND PCR (no differential verification)
**✓ Transparent reporting:** Openly reports inter-observer variability (73% discordance) among expert microscopists
**✓ Data availability:** Publicly available dataset for independent analysis
**✓ Accessible technology:** Standard smartphone (~$150) + affordable adapter ($10-100), realistic for resource-limited settings
**✓ Appropriate exclusions:** Severe disease, comorbidities, recent treatment - clinically justified

## Reported Performance

**Based on 85 patients (40 P. falciparum +, 45 negative) from SOR site only

Primary Analysis (Malaria Screener):**
• Sensitivity: 100% (95% CI: 91.2-100)
 → Perfect sensitivity BUT only 40 positive cases
 → Wide CI lower bound (91.2%) = substantial uncertainty

• Specificity: 51.1% (95% CI: 35.8-66.3)
 → LOW specificity = HIGH false positive rate (49% negatives flagged)
 → Wide CI indicates limited sample
 → Study attributes to parasite-like staining artifacts

• Overall Accuracy: 74.1%

**Post-Study Experiment (Alternative Threshold):**
• Method: Parasite candidate count threshold (not confidence scores)
• Sensitivity: 100%
• Specificity: 84.4% (improved from 51.1%)
• Overall Accuracy: 91.8% (improved from 74.1%)
• Indicates performance sensitive to threshold selection
• Exploratory analysis, should be interpreted cautiously

**Reference Standard Performance:**
• WHO Level 1 microscopists vs PCR: 94.2% accuracy
• Inter-observer variability: 73% discordance requiring arbitration

**CRITICAL LIMITATIONS:** Performance estimates apply only to P. falciparum (P. vivax excluded - 38% of positives). Small sample (40 positives) limits precision. Case-control design may overestimate accuracy. Threshold optimization may produce optimistic bias.

## Study Value and Context

**What this study demonstrates:

✓ First patient-level validation:** Pioneering evaluation of smartphone malaria diagnostics at patient level
**✓ Field feasibility:** Demonstrates AI-assisted microscopy works in endemic field settings
**✓ Accessible technology:** Low-cost equipment suitable for resource-limited areas
**✓ High sensitivity:** Perfect sensitivity (100%) for P. falciparum detection
**✓ Reference quality:** High-quality multi-reader microscopy + PCR verification

**Important limitations:

✗ Case-control design:** Spectrum bias, may overestimate accuracy vs consecutive patients
**✗ Threshold optimization:** NOT pre-specified, optimized on study data (overfitting risk)
**✗ Species exclusion:** P. vivax excluded (38% of positives) - does not represent full spectrum
**✗ Small test set:** Only 40 P. falciparum positives, wide confidence intervals
**✗ Low specificity:** 51.1% means 49% false positives (high overtreatment risk)
**✗ Site-based split:** One site for calibration, one for testing (potential site bias)
**✗ Post-hoc analyses:** Alternative thresholding suggests exploratory not confirmatory study

QUADAS-2

**Zhu et al. 2024**
Implementation of deep learning artificial intelligence in vision-threatening disease screenings for an underserved community during COVID-19 *Study ID: Zhu_2024 | Assessment Date: 2025-11-28*

____________________________________________________________________________________________________

# Phase 1: State the Review Question

*Patients (setting, intended use of index test, presentation, prior testing):*

Prospective study recruiting participants from five supervised student-run community vision screenings in West New York, New Jersey (64.4% Hispanic; 19.7% poverty rate), August 1 - November 2, 2021. Total 385 eyes from 195 screening participants included. Setting: Point-of-care - free community vision screenings in underserved area during COVID-19 pandemic. Intended use: SELENA+ cloud-based AI for automated screening of three vision-threatening diseases (VTDs): age-related macular degeneration (AMD), diabetic retinopathy (DR), and glaucoma. Presentation: Community members attending free vision screenings. Recruitment: Screenings advertised via social media and word of mouth, free and open to public. Demographics: Mean age 52.43±14.5 years, 40.0% female, 75.4% Hispanic (147/195), 15.3% African American (30/195), 6.7% White (13/195), 2.6% Asian (5/195). Equipment: Portable with mobile Wi-Fi connectivity. Operators: Trained medical student volunteers (non-specialists). CRITICAL FLAW - CONVENIENCE SAMPLING: NOT consecutive or random enrollment. Participants self-selected by attending free screenings. Creates SELECTION BIAS - individuals attending free screenings may differ systematically from general population (more eye complaints, better health-seeking behavior, greater vision awareness). May not represent full disease spectrum. UNCLEAR EXCLUSIONS: No explicit inclusion/exclusion criteria reported. Number approached versus enrolled not stated. Cannot assess if any exclusions were inappropriate.

*Index test(s):*

SELENA+ (EyRIS Pte Ltd, Singapore) - cloud-based deep learning AI (DLAI) system using convolutional neural networks to detect three vision-threatening diseases: AMD, DR, glaucoma. Imaging: Non-mydriatic 45-degree color retinal fundus images captured by trained medical student volunteers using Canon CR-2 Plus AF camera. Both macula-centered and optic nerve-centered posterior segment images taken per eye. Workflow: Images uploaded to HIPAA-compliant cloud platform (Box™), then input into cloud-based DLAI via encrypted mobile Wi-Fi hotspot. Output: For each eye, DLAI reported referable/non-referable/ungradable for each VTD. Referral criteria: Eyes with lesions consistent with DR, suspicious glaucoma features on optic disc, and/or at least intermediate dry AMD. Quality control: DLAI displayed popup warning if images appeared incorrectly placed. Grading time: 35.6±13.3 seconds (time to submit images and generate report). STRENGTH: Automated AI interpretation inherently blinded to reference standard. UNCLEAR - THRESHOLD PRE-SPECIFICATION: SELENA+ uses pre-trained thresholds for classifying referable/non-referable/ungradable. Paper references previous validation studies (ref 24, 25) but does NOT explicitly state these thresholds were pre-specified or describe specific threshold values used. Cannot confirm whether same validated thresholds used without modification for this population. CRITICAL LIMITATION - HIGH UNGRADABLE RATE: DLAI marked 38.9% of eyes (150/385) ungradable compared to only 2.6% (10/385) by human graders. Among ungradable with quality ≤3, 84/140 had ≥1+ cataract. High rate limits clinical utility and may introduce exclusion bias if handled inappropriately in accuracy calculations.

*Reference standard and target condition:*

Gold standard diagnosis established through three-tiered human grading process: (1) On-site certified tele-ophthalmology grader (BS, PhD), (2) Remote clinical ophthalmologist (MH, MD), (3) Senior ophthalmologist adjudicating disputed findings between two primary graders. Image quality graded 1-5 scale based on gamma (exposure) and alignment. Grading used standardized classifications: Age-Related Eye Disease Studies (AREES) categories for AMD, International Clinical Diabetic Retinopathy Disease Severity Scale (ICDRSS) for DR, cup-to-disc ratio and nerve fiber layer for glaucoma suspect. Inter-grader agreement: Cohen's kappa 0.946 ('almost perfect' agreement between tele-ophthalmology grader and clinical ophthalmologist). Grading time: 129±41.0 seconds (tele-ophthalmology grader), 68±21.9 seconds (clinical ophthalmologist). Additional testing: For suspected pathology, OCT-B and visual field testing performed but NOT used as definitive reference standard (for patient management, not study reference). BLINDING: Human graders established gold standard following existing tele-ophthalmology screening protocols. Clinical ophthalmologist graded remotely while DLAI ran on separate laptop via mobile Wi-Fi. No indication human graders saw AI results before assessment. Parallel independent grading pathways. CRITICAL LIMITATION - IMPERFECT REFERENCE STANDARD: Reference was expert fundus photograph interpretation, NOT definitive diagnostic testing (e.g., histopathology, comprehensive clinical exam with additional imaging). While OCT and visual field performed for 'suspected pathology,' these NOT systematically applied to all as reference standard. Fundus photo interpretation alone has inherent limitations for diagnosing glaucoma, early AMD, some DR. High inter-grader agreement (κ=0.946) suggests consistency but NOT necessarily accuracy. Target conditions: Age-related macular degeneration, diabetic retinopathy, glaucoma.

# Phase 2: Draw a Flow Diagram for the Primary Study

```

PATIENT FLOW DIAGRAM - PROSPECTIVE COMMUNITY SCREENING STUDY

STUDY PERIOD: August 1 - November 2, 2021 (3 months)
SETTING: Five supervised student-run community vision screenings
LOCATION: West New York, New Jersey
• Community demographics: 64.4% Hispanic, 19.7% poverty rate
• Context: COVID-19 pandemic period
• Underserved population with limited eye care access
    ↓
═══════════════════════════════════════════════════════════════════════
CRITICAL FLAW: CONVENIENCE SAMPLING (NOT CONSECUTIVE/RANDOM)
═══════════════════════════════════════════════════════════════════════

RECRUITMENT METHOD:
• Free vision screenings open to public
• Advertised via social media and word of mouth
• Self-referred participants
• Screenings supervised by medical students

⚠️ SELECTION BIAS CONCERN:
• Participants self-selected by attending free screenings
• NOT consecutive or random enrollment
• May differ systematically from general population:
  - More eye complaints
  - Better health-seeking behavior
  - Greater awareness of vision problems
• May not represent full disease spectrum
• Spectrum bias risk

⚠️ UNCLEAR EXCLUSIONS:
• No explicit inclusion/exclusion criteria reported
• Number of individuals approached/screened NOT stated
• Number excluded and reasons NOT reported
• Cannot assess representativeness or appropriateness
    ↓
ELIGIBLE: Community members attending free vision screenings
(Number approached/screened: NOT REPORTED)
    ↓
ENROLLED: 195 participants (385 eyes analyzed)

DEMOGRAPHICS:
• Age: Mean 52.43±14.5 years
• Sex: 40.0% female
• Ethnicity:
  - Hispanic: 147/195 (75.4%)
  - African American: 30/195 (15.3%)
  - White: 13/195 (6.7%)
  - Asian: 5/195 (2.6%)

⚠️ No information on excluded individuals
⚠️ No flow diagram showing enrollment cascade
    ↓
═══════════════════════════════════════════════════════════════════════
ALL 195 PARTICIPANTS (385 EYES) RECEIVED BOTH INDEX TEST & REFERENCE
═══════════════════════════════════════════════════════════════════════

✓ NO DIFFERENTIAL VERIFICATION
✓ NO PARTIAL VERIFICATION
✓ Appropriate time interval (parallel grading, same images)

INDEX TEST: SELENA+ Cloud-Based AI System

Technology:
• SELENA+ (EyRIS Pte Ltd, Singapore)
• Deep learning AI (DLAI) system
• Convolutional neural networks
• Cloud-based processing via encrypted mobile Wi-Fi

Imaging Equipment:
• Canon CR-2 Plus AF non-mydriatic fundus camera
• 45-degree color retinal fundus images
• Portable equipment with mobile Wi-Fi hotspot
• HIPAA-compliant cloud platform (Box™)

Image Acquisition:
• Operators: Trained medical student volunteers (non-specialists)
• Protocol: Both macula-centered AND optic nerve-centered images
• Two images per eye (posterior segment)
• Quality control: AI popup warning for incorrectly placed images

Diseases Screened (Three Vision-Threatening Diseases):
1. Age-related macular degeneration (AMD)
2. Diabetic retinopathy (DR)
3. Glaucoma

AI Output (per eye, per disease):
• Referable
• Non-referable
• Ungradable

Referral Criteria:
• DR: Lesions consistent with diabetic retinopathy
• Glaucoma: Suspicious features on optic disc
• AMD: At least intermediate dry AMD

Processing Time: 35.6±13.3 seconds
(Time to submit images and generate report)

THRESHOLD PRE-SPECIFICATION: ⚠️ UNCLEAR
• SELENA+ uses pre-trained thresholds (previously validated)
• Paper references prior validation studies (ref 24, 25)
• BUT: Does NOT explicitly state thresholds pre-specified
• Does NOT describe specific threshold values used
• Cannot confirm same validated thresholds used without modification

BLINDING: ✓ YES
• Automated AI interpretation
• Inherently blinded to reference standard
• Cannot "know" human grader interpretations
    ↓
REFERENCE STANDARD: Three-Tiered Human Grading

Grading Protocol (Three-Level Review):

TIER 1: On-site certified tele-ophthalmology grader (BS, PhD)
• Grading time: 129±41.0 seconds per eye

TIER 2: Remote clinical ophthalmologist (MH, MD)
• Grading time: 68±21.9 seconds per eye

TIER 3: Senior ophthalmologist
• Role: Adjudicated disputed findings between Tier 1 & 2
• Final arbiter for disagreements

Image Quality Assessment:
• 1-5 scale based on gamma (exposure) and alignment

Standardized Classification Systems:
• AMD: Age-Related Eye Disease Studies (AREDS) categories
• DR: International Clinical Diabetic Retinopathy Disease
       Severity Scale (ICDRSS)
• Glaucoma: Cup-to-disc ratio, nerve fiber layer assessment

Inter-Grader Agreement:
• Cohen's kappa: 0.946 between Tier 1 and Tier 2
• Interpretation: "Almost perfect" agreement
• ⚠️ High consistency BUT does NOT guarantee accuracy

Blinding: ✓ YES
• Human graders followed existing tele-ophthalmology protocols
• Clinical ophthalmologist graded remotely
• DLAI ran on separate laptop via mobile Wi-Fi
• Parallel independent grading pathways
• No indication graders saw AI results before assessment

⚠️ IMPERFECT REFERENCE STANDARD:
• Based on fundus photograph interpretation ONLY
• NOT definitive diagnostic testing (no histopathology)
• NOT comprehensive clinical examination
• Limitations for diagnosing:
  - Glaucoma (requires comprehensive exam, IOP, visual fields)
  - Early AMD (may need OCT for confirmation)
  - Some DR (fundus photos alone may miss early changes)

Additional Testing (NOT part of study reference):
• OCT-B and visual field testing performed for suspected pathology
• Used for patient management, NOT as definitive study reference
• NOT systematically applied to all participants
    ↓
═══════════════════════════════════════════════════════════════════════
CRITICAL ISSUE: HIGH UNGRADABLE RATE BY AI (38.9%)
═══════════════════════════════════════════════════════════════════════

UNGRADABLE IMAGES:

AI (SELENA+): 150/385 eyes (38.9%)
• Among quality ≤3: 84/140 had ≥1+ cataract
• High rate attributed to image quality issues

HUMAN GRADERS: 10/385 eyes (2.6%)
• Truly ungradable by gold standard

COMPARISON: AI marked 15× more eyes ungradable than humans
• 150 vs 10 eyes (38.9% vs 2.6%)

⚠️ POTENTIAL EXCLUSION BIAS:
• Handling of 38.9% ungradable in accuracy calculations UNCLEAR
• Paper does NOT explicitly describe how ungradables analyzed
• If excluded: Introduces exclusion bias
• If included as incorrect: Artificially lowers accuracy
• Ungradable eyes may systematically differ (cataracts, poor quality)
    ↓
DISEASE PREVALENCE (by reference standard):

Total Eyes Analyzed: 385 from 195 participants

GLAUCOMA SUSPECT:
• Referable: 21/195 participants (10.8%)
• Adequate sample size for analysis

DIABETIC RETINOPATHY:
• Referable: 2/195 participants (1.0%)
• ⚠️ VERY LOW PREVALENCE
• ⚠️ Only 2 positive cases - unreliable sensitivity estimates

AGE-RELATED MACULAR DEGENERATION:
• Referable: 6/195 participants (3.1%)
• ⚠️ LOW PREVALENCE
• ⚠️ Only 6 positive cases - unreliable sensitivity estimates

Sample Size Implications:
• DR sensitivity confidence interval: 100%-100% (Table 1)
  → Based on only 2 cases, extremely wide uncertainty
• AMD sensitivity confidence interval: 0.0%-86.7% (Table 1)
  → Based on only 6 cases, spans from 0 to 87%
• Glaucoma: Adequate sample (n=21) for reliable estimates
    ↓
═══════════════════════════════════════════════════════════════════════
REPORTED PERFORMANCE (details from study Tables 1-2)
═══════════════════════════════════════════════════════════════════════

⚠️ UNCLEAR: How 150/385 (38.9%) ungradable eyes handled in analysis

Inter-Grader Agreement (Human Reference):
• Cohen's kappa: 0.946 (almost perfect)
• Between tele-ophthalmology grader and clinical ophthalmologist

Processing Speed:
• AI: 35.6±13.3 seconds
• Tele-ophthalmology grader: 129±41.0 seconds (3.6× slower)
• Clinical ophthalmologist: 68±21.9 seconds (1.9× slower)

Disease-Specific Performance:
• Glaucoma: Adequate data (n=21 positive)
• DR: Limited data (n=2 positive) - wide confidence intervals
• AMD: Limited data (n=6 positive) - wide confidence intervals

═══════════════════════════════════════════════════════════════════════
SUMMARY OF METHODOLOGICAL ISSUES
═══════════════════════════════════════════════════════════════════════

1. CONVENIENCE SAMPLING (HIGH RISK):
   • Self-referred participants at free screenings
   • NOT consecutive or random enrollment
   • Selection bias - may not represent general population
   • Spectrum bias risk

2. UNCLEAR EXCLUSIONS:
   • No explicit inclusion/exclusion criteria
   • Number approached/screened not reported
   • Cannot assess appropriateness

3. THRESHOLD PRE-SPECIFICATION UNCLEAR:
   • Pre-trained thresholds from prior validation
   • But NOT explicitly stated as pre-specified for this study
   • Specific threshold values not described

4. IMPERFECT REFERENCE STANDARD:
   • Fundus photo interpretation only
   • NOT definitive diagnostic testing
   • Inherent limitations for glaucoma, early AMD, some DR
   • High agreement (κ=0.946) shows consistency not accuracy

5. HIGH UNGRADABLE RATE (38.9%):
   • AI marked 150/385 eyes ungradable
   • 15× higher than human graders (10/385)
   • Handling in accuracy calculations UNCLEAR
   • Potential exclusion bias

6. LOW DISEASE PREVALENCE FOR DR/AMD:
   • DR: Only 2/195 (1.0%) - unreliable sensitivity
   • AMD: Only 6/195 (3.1%) - unreliable sensitivity
   • Wide confidence intervals (Table 1)

7. NO CONSORT/STARD FLOW DIAGRAM:
   • Cannot trace patient flow clearly
   • Enrollment cascade not documented

```

# Phase 3: Risk of Bias and Applicability Judgments

# DOMAIN 1: PATIENT SELECTION

## A. Risk of Bias

**Describe methods of patient selection:**
Prospective study recruiting from five supervised student-run community vision screenings in West New York, NJ (64.4% Hispanic; 19.7% poverty rate), August 1 - November 2, 2021. Total 385 eyes from 195 participants. Vision screenings free and open to public, advertised via social media and word of mouth. CONVENIENCE SAMPLING - NOT consecutive or random enrollment. Participants self-selected by attending free screenings. Demographics: Mean age 52.43±14.5 years, 40.0% female, 75.4% Hispanic (147/195), 15.3% African American (30/195), 6.7% White (13/195), 2.6% Asian (5/195). Inclusion/exclusion criteria NOT explicitly stated. Number of individuals approached versus enrolled NOT reported. Cannot assess if any exclusions inappropriate. CRITICAL FLAW: Self-selection introduces SELECTION BIAS - individuals attending free vision screenings may differ systematically from general target population (more eye complaints, better health-seeking behavior, greater vision awareness). May not represent full disease spectrum. Page reference: p.1591, p.1592

| Signaling Question | Answer |
| --- | --- |
| ❖ Was a consecutive or random sample enrolled? | NO |
| ❖ Was a case-control design avoided? | YES |
| ❖ Did study avoid inappropriate exclusions? | UNCLEAR |

**Could the selection of patients have introduced bias?: RISK: HIGH**

**Justification:** The study used convenience sampling through self-referred participants at free community screenings advertised via social media and word of mouth. This introduces selection bias because individuals who attend free vision screenings may differ systematically from the general target population - they may have more eye complaints, better health-seeking behavior, or greater awareness of vision problems. Question 1.1 is answered 'No' because enrollment was neither consecutive nor random. Additionally, the lack of explicit inclusion/exclusion criteria and absence of information about any individuals who were approached but not enrolled makes it impossible to assess representativeness or potential spectrum bias.

## B. Concerns regarding applicability

**Is there concern that the included patients do not match the review question?: CONCERN: LOW**

**Justification:** The study population matches the review question well. This was a real-world point-of-care screening application in an underserved, primarily Hispanic community (75.4% Hispanic) in a community setting during COVID-19. The mean age was 52.43±14.5 years with 40.0% female participants. The setting (community screenings using portable equipment with mobile Wi-Fi) represents an authentic point-of-care context for AI-assisted vision screening in underserved populations. This is directly applicable to understanding AI performance in non-traditional, resource-limited POC settings.

# DOMAIN 2: INDEX TEST(S)

## A. Risk of Bias

**Describe the index test:**
SELENA+ (EyRIS Pte Ltd, Singapore) - cloud-based deep learning AI system using CNNs to detect three vision-threatening diseases: AMD, DR, glaucoma. Non-mydriatic 45-degree color retinal fundus images captured by trained medical student volunteers using Canon CR-2 Plus AF camera. Both macula-centered and optic nerve-centered images per eye. Images uploaded to HIPAA-compliant cloud platform (Box™), input into cloud-based DLAI via encrypted mobile Wi-Fi hotspot. Output per eye: referable/non-referable/ungradable for each VTD. Referral criteria: Lesions consistent with DR, suspicious glaucoma features on optic disc, at least intermediate dry AMD. Quality control: AI popup for incorrectly placed images. Grading time: 35.6±13.3 seconds. STRENGTH: Automated AI inherently blinded to reference. UNCLEAR - THRESHOLD: SELENA+ uses pre-trained thresholds for classification. Paper references previous validation studies (ref 24, 25) but does NOT explicitly state thresholds were pre-specified or describe specific values. Cannot confirm same validated thresholds used without modification. CRITICAL LIMITATION - HIGH UNGRADABLE: 150/385 eyes (38.9%) marked ungradable by AI vs only 10/385 (2.6%) by humans. Among quality ≤3, 84/140 had ≥1+ cataract. High rate limits utility and may introduce exclusion bias. Page reference: p.1591, p.1592-1593

| Signaling Question | Answer |
| --- | --- |
| ❖ Interpreted without knowledge of reference? | YES |
| ❖ If threshold used, was it pre-specified? | UNCLEAR |

**Could the conduct of the index test have introduced bias?: RISK: UNCLEAR**

**Justification:** The AI system (SELENA+) automatically interpreted images without knowledge of the reference standard, satisfying question 2.1. However, question 2.2 is 'Unclear' because while SELENA+ is a previously validated system with established thresholds for VTD detection (references 24, 25), the paper does not explicitly confirm that pre-specified thresholds were used without modification or optimization for this specific population. The paper does not state the specific probability thresholds for referral decisions. Since question 2.2 is unclear and question 2.1 is 'Yes,' the overall risk is UNCLEAR rather than HIGH.

## B. Concerns regarding applicability

**Is there concern about the index test applicability?: CONCERN: LOW**

**Justification:** The index test closely matches typical point-of-care AI applications. The DLAI was implemented in a real-world community screening setting using non-mydriatic fundus photography, portable equipment, and mobile Wi-Fi connectivity. Medical student volunteers operated the imaging equipment after brief training, reflecting realistic deployment conditions where specialized personnel may not be available. The cloud-based AI requiring only internet connectivity represents a scalable POC solution applicable to the review question.

# DOMAIN 3: REFERENCE STANDARD

## A. Risk of Bias

**Describe the reference standard:**
Gold standard diagnosis established through three-tiered human grading: (1) On-site certified tele-ophthalmology grader (BS, PhD), (2) Remote clinical ophthalmologist (MH, MD), (3) Senior ophthalmologist adjudicating disputed findings. Image quality graded 1-5 scale based on gamma (exposure) and alignment. Standardized classifications: Age-Related Eye Disease Studies (AREDS) for AMD, International Clinical Diabetic Retinopathy Disease Severity Scale (ICDRSS) for DR, cup-to-disc ratio and nerve fiber layer for glaucoma. Inter-grader agreement: Cohen's kappa 0.946 ('almost perfect' between tele-ophthalmology grader and clinical ophthalmologist). Grading time: 129±41.0 sec (tele-ophthalmology), 68±21.9 sec (clinical ophthalmologist). Additional testing: OCT-B and visual field for suspected pathology but NOT used as definitive reference (patient management, not study reference). BLINDING: Human graders followed existing protocols, clinical ophthalmologist graded remotely, DLAI ran separately. Parallel independent grading, no indication graders saw AI results. CRITICAL LIMITATION - IMPERFECT REFERENCE: Expert fundus photo interpretation, NOT definitive diagnostic testing (no histopathology, no comprehensive clinical exam). OCT/visual fields for 'suspected pathology' but NOT systematically applied to all. Fundus photos alone have inherent limitations for glaucoma, early AMD, some DR. High agreement (κ=0.946) shows consistency NOT necessarily accuracy. Page reference: p.1591-1592, p.1594

| Signaling Question | Answer |
| --- | --- |
| ❖ Is reference standard likely to correctly classify? | UNCLEAR |
| ❖ Interpreted without knowledge of index test? | YES |

**Could the reference standard have introduced bias?: RISK: UNCLEAR**

**Justification:** The reference standard was clinical expert interpretation of fundus images using standardized grading scales (AREDS, ICDRSS), not definitive diagnostic testing (e.g., histopathology, comprehensive clinical exam with additional imaging). While OCT and visual field testing were performed for 'suspected pathology,' these were not systematically applied to all patients as the reference standard. Fundus photograph interpretation alone has inherent limitations for diagnosing glaucoma, early AMD, and some DR. The high inter-grader agreement (κ=0.946) suggests consistency but not necessarily accuracy. This represents an imperfect reference standard that may misclassify conditions, particularly glaucoma which requires comprehensive examination beyond fundus photography. However, human graders were appropriately blinded to AI results.

## B. Concerns regarding applicability

**Is there concern about reference standard applicability?: CONCERN: LOW**

**Justification:** The reference standard (expert fundus photograph interpretation using standardized grading scales) is appropriate for vision screening contexts and matches typical tele-ophthalmology protocols. The three-tiered grading process with adjudication represents rigorous quality control. The target conditions (AMD, DR, glaucoma) are clearly defined using established classification systems (AREDS, ICDRSS). While not definitive diagnostic testing, this represents an appropriate reference for a screening study in a community setting.

# DOMAIN 4: FLOW AND TIMING

## A. Risk of Bias

**Patient flow:**
All 195 participants (385 eyes) received both index test (SELENA+ AI) and reference standard (three-tiered human grading). NO differential verification - all received same reference regardless of AI results. NO partial verification - all received complete reference. Time interval appropriate - parallel grading on same images, no interval between tests. STRENGTH: Complete verification without bias. HOWEVER, UNCLEAR - HANDLING OF UNGRADABLES: AI marked 150/385 eyes (38.9%) ungradable compared to 10/385 (2.6%) by humans. Paper does NOT explicitly describe how these 38.9% ungradable eyes were handled in accuracy calculations. If excluded from analysis: Introduces potential EXCLUSION BIAS (ungradable eyes with cataracts may systematically differ). If included as incorrect: Artificially lowers AI accuracy. Among quality ≤3, 84/140 ungradables had ≥1+ cataract. Cannot determine from methods whether analysis included or excluded ungradables, creating uncertainty about bias in flow. No formal CONSORT/STARD flow diagram to trace patient handling. Page reference: p.1591-1593

| Signaling Question | Answer |
| --- | --- |
| ❖ Appropriate interval between tests? | YES |
| ❖ Did all patients receive reference standard? | YES |
| ❖ Did patients receive same reference standard? | YES |
| ❖ Were all patients included in analysis? | UNCLEAR |

**Could the patient flow have introduced bias?: RISK: UNCLEAR**

**Justification:** The study has excellent verification practices - all 195 participants (385 eyes) received both the index test and reference standard with no differential or partial verification. Time interval was appropriate (parallel grading on same images). However, there is UNCLEAR risk due to the handling of ungradable images. The AI marked 150/385 eyes (38.9%) as ungradable compared to only 10/385 (2.6%) by human graders - a 15-fold difference. The paper does not explicitly describe how these ungradable eyes were handled in accuracy calculations. If excluded, this introduces potential exclusion bias since ungradable eyes (many with cataracts) may systematically differ from gradable eyes. The lack of a formal flow diagram makes it impossible to trace patient handling definitively.

# OVERALL ASSESSMENT SUMMARY

**HIGH RISK OF BIAS - CONVENIENCE SAMPLING + UNCERTAINTIES**

| Domain | Signaling Questions | Risk of Bias | Applicability |
| --- | --- | --- | --- |
| D1: Patient Selection | 1.1 No 1.2 Yes 1.3 Unclear | HIGH | LOW |
| D2: Index Test | 2.1 Yes 2.2 Unclear | UNCLEAR | LOW |
| D3: Reference Standard | 3.1 Unclear 3.2 Yes | UNCLEAR | LOW |
| D4: Flow and Timing | 4.1 Yes 4.2 Yes 4.3 Yes 4.4 Unclear | UNCLEAR | N/A |

**Overall Risk of Bias: HIGH**

**Justification:** This study has HIGH overall risk of bias due to one HIGH-risk domain (Patient Selection) and three UNCLEAR domains. Domain 1 (Patient Selection): HIGH - Convenience sampling through self-referred participants at free community screenings introduces selection bias. Participants who attend may differ systematically from the general population (more eye complaints, better health-seeking behavior). Enrollment was neither consecutive nor random. Domain 2 (Index Test): UNCLEAR - While the AI was appropriately blinded, threshold pre-specification is unclear. The paper does not explicitly state whether pre-specified thresholds were used. Domain 3 (Reference Standard): UNCLEAR - The reference standard relied on expert fundus photograph interpretation rather than definitive diagnostic testing. This represents an imperfect reference standard that may misclassify conditions, particularly glaucoma which requires comprehensive examination beyond fundus photography. Domain 4 (Flow & Timing): UNCLEAR - The handling of the high ungradable rate (38.9%) in accuracy calculations is not explicitly described, creating uncertainty about potential exclusion bias.

**Overall Applicability Concern: LOW**

**Justification:** Despite methodological limitations, this study has strong applicability to the review question. The study evaluated a cloud-based AI system (SELENA+) in a real-world point-of-care community screening setting in an underserved population. Key applicability strengths include: deployment via mobile Wi-Fi hotspot without fixed internet infrastructure, operation by trained medical students rather than ophthalmology specialists, use of non-mydriatic imaging in a non-clinical environment, and targeting an underserved Hispanic population (75.4%) with limited access to eye care. These conditions authentically represent POC AI deployment scenarios in resource-limited settings, making findings directly relevant to understanding real-world AI performance in clinical decision support for vision screening.

## Critical Biases and Limitations

**1. SELECTION BIAS FROM CONVENIENCE SAMPLING (SEVERITY: HIGH):**
Participants self-selected by attending free community screenings advertised via social media and word of mouth. NOT consecutive or random enrollment. Individuals attending free vision screenings may differ systematically from general population - may have more eye complaints, better health-seeking behavior, greater awareness of vision problems. Creates spectrum bias risk as enrolled population may not represent full disease spectrum. No explicit inclusion/exclusion criteria. Number approached versus enrolled not reported.

**2. IMPERFECT REFERENCE STANDARD (SEVERITY: UNCLEAR):**
Reference was expert fundus photograph interpretation using standardized scales (AREDS, ICDRSS), NOT definitive diagnostic testing. No histopathology, no systematic comprehensive clinical examination. While OCT and visual field testing performed for "suspected pathology," these NOT systematically applied to all as reference. Fundus photo interpretation alone has inherent limitations for diagnosing glaucoma (requires IOP, visual fields, comprehensive exam), early AMD (may need OCT), and some DR. High inter-grader agreement (κ=0.946) shows consistency but NOT necessarily accuracy. May misclassify true disease status.

**3. HIGH UNGRADABLE RATE - UNCLEAR HANDLING (SEVERITY: UNCLEAR):**
AI marked 150/385 eyes (38.9%) ungradable compared to only 10/385 (2.6%) by human graders - 15-fold difference. Among quality ≤3, 84/140 ungradables had ≥1+ cataract. Paper does NOT explicitly describe how these 38.9% were handled in accuracy calculations. If excluded: Introduces exclusion bias (ungradable eyes with cataracts may systematically differ). If included as incorrect: Artificially lowers AI accuracy. Creates uncertainty about true performance.

**4. THRESHOLD PRE-SPECIFICATION UNCERTAIN (SEVERITY: UNCLEAR):**
SELENA+ uses pre-trained thresholds from prior validation (references 24, 25), but paper does NOT explicitly state these thresholds were pre-specified for current study or describe specific threshold values. Cannot confirm same validated thresholds used without modification or optimization for this population. Creates uncertainty about potential optimistic bias.

**5. VERY LOW DISEASE PREVALENCE FOR DR/AMD (SEVERITY: MODERATE):**
DR: Only 2/195 (1.0%) referable - extremely low prevalence. AMD: Only 6/195 (3.1%) referable - low prevalence. Small disease-positive samples yield unreliable sensitivity estimates with very wide confidence intervals. Table 1 shows DR sensitivity CI: 100%-100% (only 2 cases), AMD sensitivity CI: 0.0%-86.7% (only 6 cases). Limits precision and generalizability of disease-specific performance.

## Significant Methodological Strengths

**Despite biases, this study has important strengths:

✓ Prospective real-world evaluation:** Not retrospective dataset validation, authentic POC conditions
**✓ Complete verification:** All 195 participants (385 eyes) received both index test and reference (no differential/partial verification)
**✓ High inter-rater reliability:** Cohen's kappa 0.946 between human graders (almost perfect agreement)
**✓ Novel deployment setting:** Underserved community screening using mobile Wi-Fi, portable equipment
**✓ Non-specialist operators:** Trained medical students operated equipment (realistic deployment)
**✓ Comprehensive VTD coverage:** Simultaneous screening for AMD, DR, and glaucoma
**✓ Appropriate timing:** No interval between index test and reference (parallel grading on same images)
**✓ Three-tiered reference:** Rigorous multi-level grading with adjudication
**✓ Underserved population:** 75.4% Hispanic, 19.7% poverty rate - high applicability

## Reported Performance Context

**Performance metrics subject to multiple biases

Sample Composition (385 eyes, 195 participants):**
• Glaucoma suspect: 21/195 (10.8%) - ADEQUATE sample
• DR referable: 2/195 (1.0%) - VERY LOW (unreliable estimates)
• AMD referable: 6/195 (3.1%) - LOW (unreliable estimates)

**Ungradable Rate (Critical Limitation):**
• AI (SELENA+): 150/385 eyes (38.9%)
• Human graders: 10/385 eyes (2.6%)
• Ratio: AI marked 15× more ungradable than humans
• Among quality ≤3: 84/140 had ≥1+ cataract
• Handling in accuracy calculations UNCLEAR

**Processing Speed:**
• AI: 35.6±13.3 seconds
• Tele-ophthalmology grader: 129±41.0 seconds (3.6× slower)
• Clinical ophthalmologist: 68±21.9 seconds (1.9× slower)

**Confidence Interval Widths (Table 1):**
• DR sensitivity: 100%-100% (based on only 2 cases)
 → Extremely wide uncertainty despite appearing "perfect"
• AMD sensitivity: 0.0%-86.7% (based on only 6 cases)
 → Spans from 0% to 87%, essentially uninformative
• Glaucoma: Adequate sample (n=21) for reliable estimates

**INTERPRETATION:** Performance should be interpreted cautiously due to: (1) Convenience sampling bias, (2) Imperfect reference standard, (3) Unclear handling of 38.9% ungradable, (4) Very low DR/AMD prevalence limiting precision, (5) Threshold pre-specification uncertainty. Actual performance in unselected populations with definitive diagnosis may differ.

## Study Value and Context

**What this study demonstrates:

✓ Real-world feasibility:** Cloud-based AI can be deployed in community screenings using mobile Wi-Fi
**✓ Underserved populations:** Demonstrates implementation in Hispanic community (75.4%) with limited access
**✓ Non-specialist operation:** Medical students can operate system after training
**✓ Multi-disease screening:** Simultaneous VTD detection (AMD, DR, glaucoma) from fundus photos
**✓ Processing speed:** AI significantly faster than human graders (35 vs 68-129 seconds)

**Important limitations:

✗ Convenience sampling:** Selection bias, not representative of general population
**✗ Imperfect reference:** Fundus photo interpretation only, not definitive diagnosis
**✗ High ungradable rate:** 38.9% ungradable (15× higher than humans), limits clinical utility
**✗ Low DR/AMD prevalence:** Only 2 DR and 6 AMD cases, unreliable sensitivity estimates
**✗ Threshold unclear:** Pre-specification not explicitly documented
**✗ Ungradable handling unclear:** How 38.9% handled in analysis not described
**✗ No flow diagram:** Patient handling not clearly traceable
