# Supplementary material for: AI in Point-of-Care Imaging for Clinical Decision Support: Systematic Review of Diagnostic Accuracy, Task-Shifting, and Explainability
Source: JMIR AI. 2026 Apr 27;5:e80928. doi: 10.2196/80928 (PMC13119389; doi:10.2196/80928)
Supplement: Multimedia Appendix 5 — Sensitivity analysis data and results. [file ai-v5-e80928-s005.docx]

**Sensitivity Analysis**

Artificial Intelligence in Point-of-Care Imaging for Clinical Decision Support: Systematic Review of Diagnostic Accuracy, Task-Shifting, and Explainability

**Overview**

This sensitivity analysis was conducted to assess the robustness of diagnostic performance findings to methodological quality. Given that 70% (14/20) of included studies were rated as high or very high overall risk of bias per QUADAS-2 assessment, we restricted the analysis to studies with low to moderate overall risk of bias (n=6). We compared sensitivity, specificity, and area under the receiver operating characteristic curve (AUC) between the full analysis (n=20) and the sensitivity analysis (n=6) to determine whether methodological limitations substantially influenced reported performance estimates.

**Table S6.1: Study Inclusion for Sensitivity Analysis**

| **Study** | **Clinical Condition** | **QUADAS-2 Overall Risk** | **Included in Sensitivity Analysis** |
| --- | --- | --- | --- |
| **LOW RISK** |  |  |  |
| **Heydon et al., 2021** | Diabetic retinopathy | Low | Yes |
| **Kazemzadeh et al., 2024** | Tuberculosis | Low | Yes |
| **LOW-MODERATE RISK** |  |  |  |
| **Iacob et al., 2025** | Cardiac pathology | Low-Moderate | Yes |
| **MODERATE RISK** |  |  |  |
| **Chen et al., 2023** | Ophthalmic emergencies | Moderate | Yes |
| **Nath et al., 2024** | Tuberculosis | Moderate | Yes |
| **Nothnagel et al., 2024** | Deep vein thrombosis | Moderate | Yes |
| **HIGH RISK** |  |  |  |
| **Avgerinos et al., 2025** | Deep vein thrombosis | High | No |
| **Jaremko et al., 2023** | Hip dysplasia | High | No |
| **Jayaraman et al., 2025** | Tuberculosis | High | No |
| **Love et al., 2018** | Breast masses | High | No |
| **Marquez et al., 2025** | Tuberculosis | High | No |
| **Papachristou et al., 2024** | Melanoma | High | No |
| **Poli et al., 2024** | Cervical cancer | High | No |
| **Yang et al., 2019** | Helminthiases | High | No |
| **Yu et al., 2023** | Malaria | High | No |
| **Zhu et al., 2024** | Vision-threatening diseases | High | No |
| **VERY HIGH RISK** |  |  |  |
| **Berg et al., 2023** | Breast cancer | Very High | No |
| **Cao et al., 2025** | Tuberculosis | Very High | No |
| **Fergus et al., 2023** | Pressure ulcers | Very High | No |
| **Malherbe et al., 2025** | Breast cancer | Very High | No |

**Summary: 6 studies included (30%), 14 studies excluded (70%)**

**Table S6.2: Diagnostic Performance of Low to Moderate Risk-of-Bias Studies**

| **Study** | **Condition** | **N** | **Sensitivity % (95% CI)** | **Specificity % (95% CI)** | **AUC (95% CI)** | **PPV %** | **NPV %** |
| --- | --- | --- | --- | --- | --- | --- | --- |
| Chen et al., 2023 | Ophthalmic emergencies | 364 | 90.0-96.2 (86.4-93.6) | NR | 0.98 (0.97-1.00) | NR | NR |
| Heydon et al., 2021 | Diabetic retinopathy | 30,405 | 95.7 (94.8-96.5) | 54.0 (53.4-54.5) | NR | 14 | 99 |
| Iacob et al., 2025 | Cardiac pathology | 1,780 | 89.9 (87.2-92.2) | 96.5 (95.3-97.5) | 0.94 (0.92-0.96) | 93 | 95 |
| Kazemzadeh et al., 2024 | Tuberculosis | 1,827 | 87 (82-92) | 70 (67-72) | 0.87 (0.84-0.90) | NR | NR |
| Nath et al., 2024 | Tuberculosis | 4,363 | 88 (85-93) | 85 (82-91) | 0.85 (0.82-0.87) | 88 | 85 |
| Nothnagel et al., 2024 | DVT | 58 | 100 (99.1-100) | 90.6 (90.5-91.7) | NR | NR | NR |

**Summary Statistics:**

Sensitivity: Range 87.0-100.0%, Median 90.0% (6/6 studies reported)

Specificity: Range 54.0-96.5%, Median 85.0% (5/6 studies reported)

AUC: Range 0.85-0.98, Median 0.91 (4/6 studies reported)

*AUC = Area under the receiver operating characteristic curve; CI = Confidence interval; DVT = Deep vein thrombosis; NR = Not reported; NPV = Negative predictive value; PPV = Positive predictive value*

**Table S6.3: Comparison of Main Analysis vs Sensitivity Analysis**

| **Performance Metric** | **Main Analysis (n=20)** | **Sensitivity Analysis (n=6)** | **Difference** |
| --- | --- | --- | --- |
| **SENSITIVITY** |  |  |  |
| **Number reporting** | 18 | 6 | −12 |
| **Range** | 62.5% to 100.0% | 87.0% to 100.0% | Lower bound +24.5 pp |
| **Median** | 92.0% | 90.0% | −2.0 pp |
| **SPECIFICITY** |  |  |  |
| **Number reporting** | 17 | 5 | −12 |
| **Range** | 28.1% to 100.0% | 54.0% to 96.5% | Lower bound +25.9 pp |
| **Median** | 90.6% | 85.0% | −5.6 pp |
| **AUC** |  |  |  |
| **Number reporting** | 10 | 4 | −6 |
| **Range** | 0.63 to 1.00 | 0.85 to 0.98 | Lower bound +0.22 |
| **Median** | 0.87 | 0.91 | +0.04 |

*AUC = Area under the receiver operating characteristic curve; pp = percentage points*

**Interpretation**

The sensitivity analysis demonstrates that diagnostic performance estimates remain robust when restricted to studies with lower risk of bias. Key findings include:

**Stability of Central Estimates:** Median AUC increased slightly from 0.87 to 0.91, while median sensitivity and specificity showed minimal changes (−2.0 and −5.6 percentage points, respectively). This stability suggests that the primary conclusions regarding diagnostic accuracy are not driven by methodologically weaker studies.

**Elimination of Low-Performing Outliers:** The narrowing of performance ranges in the sensitivity analysis (sensitivity lower bound increased from 62.5% to 87.0%; specificity lower bound increased from 28.1% to 54.0%) resulted from exclusion of studies with both high risk of bias and notably lower performance (Fergus et al., 2023, AUC 0.63-0.93; Poli et al., 2024, sensitivity 62.5%; Marquez et al., 2025, specificity 28.1%; Zhu et al., 2024, sensitivity 63.2%). This pattern suggests that methodological limitations may have contributed to underestimated rather than overestimated performance in some studies.

**Implications for Review Conclusions:** Despite 70% of studies carrying high or very high risk of bias, the core finding that AI systems demonstrate generally high diagnostic accuracy (sensitivity 87-100%, AUC 0.85-0.98 in methodologically stronger studies) remains supported. However, the substantial proportion of lower-quality studies limits confidence in secondary outcomes such as clinical workflow impacts, cost-effectiveness, and performance generalizability across diverse healthcare settings—domains where measurement quality and reporting rigor are particularly critical.

**Limitations of Sensitivity Analysis:** The small number of low-to-moderate risk studies (n=6) limits statistical power and spans only 5 clinical conditions (tuberculosis n=2, diabetic retinopathy n=1, cardiac pathology n=1, ophthalmic emergencies n=1, DVT n=1), reducing the evidence base for several key review questions including cross-modality comparisons and performance in resource-limited settings.

*END OF SUPPLEMENTARY MATERIAL 6*
