# Supplementary material for: AI in Point-of-Care Imaging for Clinical Decision Support: Systematic Review of Diagnostic Accuracy, Task-Shifting, and Explainability
Source: JMIR AI. 2026 Apr 27;5:e80928. doi: 10.2196/80928 (PMC13119389; doi:10.2196/80928)
Supplement: Multimedia Appendix 6 — Reporting bias assessment details. [file ai-v5-e80928-s006.docx]

**Reporting Bias Assessment**

Artificial Intelligence in Point-of-Care Imaging for Clinical Decision Support: Systematic Review of Diagnostic Accuracy, Task-Shifting, and Explainability

**Overview**

This reporting bias assessment provides a qualitative evaluation of potential biases affecting the systematic review evidence base. Because the review employed narrative synthesis rather than meta-analysis due to substantial clinical and methodological heterogeneity across included studies (12 distinct conditions, 6 imaging modalities, diverse study designs and settings), formal statistical methods for assessing publication bias (such as Deeks' funnel plot asymmetry testing) were not feasible. Such methods require sufficient homogeneity to conduct meta-analysis and an adequate number of studies examining the same condition with comparable methodology. Instead, this assessment examines: (1) literature search comprehensiveness, (2) patterns potentially indicative of publication bias (commercial system predominance, small-study effects), and (3) selective outcome reporting within included studies. The assessment characterizes limitations affecting confidence in the evidence base and informs interpretation of review findings.

**Table S7.1: Outcome Reporting Completeness by Study**

| **Author, Year** | **Sens** | **Spec** | **AUC** | **PPV** | **NPV** | **Sens 95% CI** | **Spec 95% CI** |
| --- | --- | --- | --- | --- | --- | --- | --- |
| Avgerinos et al., 2025 | Y | Y | N | Y | Y | N | N |
| Berg et al., 2023 | Y | Y | Y | N | Y | Y | Y |
| Cao et al., 2025 | Y | Y | N | N | N | Y | Y |
| Chen et al., 2023 | Y | N | Y | N | N | Y | N |
| Fergus et al., 2023 | Y | N | Y | Y | N | N | N |
| Heydon et al., 2021 | Y | Y | N | Y | Y | Y | Y |
| Iacob et al., 2025 | Y | Y | Y | Y | Y | Y | Y |
| Jaremko et al., 2023 | N | Y | N | N | N | N | N |
| Jayaraman et al., 2025 | Y | Y | N | N | N | Y | Y |
| Kazemzadeh et al., 2024 | Y | Y | Y | N | N | Y | Y |
| Love et al., 2018 | Y | Y | Y | N | N | N | N |
| Malherbe et al., 2025 | N | N | N | N | N | N | N |
| Marquez et al., 2025 | Y | Y | Y | Y | Y | Y | Y |
| Nath et al., 2024 | Y | Y | Y | Y | Y | Y | Y |
| Nothnagel et al., 2024 | Y | Y | N | N | N | Y | Y |
| Papachristou et al., 2024 | Y | Y | Y | Y | Y | N | N |
| Poli et al., 2024 | Y | Y | Y | Y | Y | Y | Y |
| Yang et al., 2019 | Y | Y | N | Y | Y | N | N |
| Yu et al., 2023 | Y | Y | N | N | N | Y | Y |
| Zhu et al., 2024 | Y | Y | N | Y | Y | Y | Y |

*Legend: Y = Reported, N = Not Reported*

Summary: Core diagnostic accuracy metrics (sensitivity, specificity) were well reported across most studies (90% and 85% respectively), while secondary metrics and precision estimates showed moderate to substantial gaps. One study (Malherbe et al., 2025) reported no quantitative diagnostic performance metrics.

**Table S7.2: Summary of Outcome Reporting Completeness**

| **Metric** | **Studies Reporting** | **Percentage** |
| --- | --- | --- |
| Sensitivity | 18/20 | 90% |
| Specificity | 17/20 | 85% |
| AUC | 10/20 | 50% |
| PPV | 10/20 | 50% |
| NPV | 10/20 | 50% |
| Sensitivity 95% CI | 13/20 | 65% |
| Specificity 95% CI | 12/20 | 60% |

**Interpretation:** Primary diagnostic metrics (sensitivity, specificity) demonstrated high reporting completeness (85-90%), suggesting low risk of selective non-reporting of core outcomes. However, moderate reporting gaps for secondary metrics (AUC, PPV, NPV: 50% reporting) and precision estimates (confidence intervals: 60-65% reporting) limit meta-analytic potential and interpretation of estimate uncertainty. The pattern does not suggest systematic suppression of unfavorable results, as both favorable and unfavorable performance estimates are represented across all metric categories.

**Table S7.3: Study Characteristics Relevant to Publication Bias**

| **Author, Year** | **Sample Size** | **Size Category** | **Commercial Status** | **Performance (Sens/Spec)** |
| --- | --- | --- | --- | --- |
| Avgerinos et al., 2025 | 53 | Small | Commercial | 100% / 95.7% |
| Berg et al., 2023 | 758 | Medium | Commercial | 95% / 79% |
| Cao et al., 2025 | 3,705 | Large | Commercial | 92.1% / 94.5% |
| Chen et al., 2023 | 364 | Medium | Research | 93.1% / NR |
| Fergus et al., 2023 | 216 | Medium | Research | 70% / NR |
| Heydon et al., 2021 | 30,405 | Large | Commercial | 95.7% / 54% |
| Iacob et al., 2025 | 1,780 | Large | Commercial | 89.9% / 96.5% |
| Jaremko et al., 2023 | 306 | Medium | Commercial | NR / 100% |
| Jayaraman et al., 2025 | 25,598 | Large | Commercial | 98% / 96.9% |
| Kazemzadeh et al., 2024 | 1,827 | Large | Research | 87% / 70% |
| Love et al., 2018 | 32 | Small | Research | 100% / 100% |
| Malherbe et al., 2025 | 203 | Medium | Commercial | NR / NR |
| Marquez et al., 2025 | 5,740 | Large | Commercial | 95.6% / 28.1% |
| Nath et al., 2024 | 4,363 | Large | Research | 88% / 85% |
| Nothnagel et al., 2024 | 58 | Small | Commercial | 100% / 90.6% |
| Papachristou et al., 2024 | 253 | Medium | Commercial | 95.2% / 84.5% |
| Poli et al., 2024 | 2,052 | Large | Research | 62.5% / 97.6% |
| Yang et al., 2019 | 113 | Small | Open source | 85.7% / 87.5% |
| Yu et al., 2023 | 137* | Small | Research | 93.5% / 71.1% |
| Zhu et al., 2024 | 385 | Medium | Commercial | 63.2% / 94.5% |

**Midpoint of reported range (85-189)*

*Notes: Size categories defined as Small (N<200), Medium (N=200-1000), Large (N>1000). Commercial status categorized as Commercial (vendor-developed systems with commercial intent), Research (academic prototypes), or Open source (publicly available code). Performance values represent point estimates where reported; NR indicates metric not reported.*

**Table S7.4: Small-Study Effects Analysis**

| **Size Category** | **N Studies** | **Sensitivity Range** | **Specificity Range** | **Median Sens** | **Median Spec** |
| --- | --- | --- | --- | --- | --- |
| Small (<200) | 5 | 85.7-100% | 71.1-100% | 100.0% | 90.6% |
| Medium (200-1000) | 7 | 63.2-95.2% | 79.0-100% | 93.1% | 89.5% |
| Large (>1000) | 8 | 62.5-98.0% | 28.1-97.6% | 91.0% | 89.8% |

**Interpretation:** Small studies demonstrated systematically higher and less variable performance compared to larger studies. The sensitivity range in small studies (85.7-100%, span 14.3 percentage points) was narrower than in large studies (62.5-98.0%, span 35.5 percentage points), suggesting possible selective publication or reporting of favorable results from smaller studies. The median sensitivity in small studies (100%) exceeded that of both medium studies (93.1%) and large studies (91.0%). For specificity, small studies showed higher median performance (90.6%) compared to medium studies (89.5%) and large studies (89.8%), though the differences were modest. Small studies again showed narrower specificity ranges compared to large studies, which exhibited marked heterogeneity (28.1-97.6%). These patterns are consistent with small-study effects, where smaller studies preferentially report positive findings, potentially due to publication bias, selective outcome reporting, or genuinely different performance in highly selected small populations. However, the limited number of studies per category (5-8) and substantial clinical heterogeneity limit definitive conclusions about small-study bias.

**Overall Assessment**

**Reporting Bias Risk:** Moderate

**Key Findings:**

**1. Search comprehensiveness:** The systematic search covered four major bibliographic databases (PubMed, Scopus, IEEE Xplore, Web of Science) with comprehensive search strategies. However, grey literature (conference abstracts, dissertations, technical reports), preprint servers (medRxiv, arXiv), and clinical trial registries were not systematically searched. Language restrictions (English only) may have excluded relevant non-English studies. The absence of grey literature searches may have missed unpublished negative or null findings, particularly from pilot studies or early-stage validations that failed to reach peer-reviewed publication.

**2. Publication bias indicators:** Commercial AI systems predominated (60%, n=12/20), potentially introducing bias favoring positive results from vendor-sponsored research, as commercial entities may be less likely to publish unfavorable validations. Small-study effects were evident: small studies (N<200) showed higher median sensitivity (100.0% vs 93.1% in medium studies) and narrower performance ranges (sensitivity span 14.3 vs 35.5 percentage points in large studies), consistent with preferential publication of favorable small-study results. However, eight large studies (40%) provide counterbalance, including studies with modest performance (Marquez et al.: 28.1% specificity; Poli et al.: 62.5% sensitivity), suggesting that negative findings are represented in the evidence base.

**3. Selective outcome reporting:** Core metrics (sensitivity 90%, specificity 85%) were well reported, but moderate gaps existed for AUC (50%), PPV/NPV (50%), and confidence intervals (60-65%). One study reported no diagnostic metrics. The pattern does not strongly suggest outcome-selective reporting bias, as both favorable and unfavorable results were reported across all metric categories. However, the absence of precision estimates in 35-40% of studies limits assessment of estimate uncertainty.

**4. Small-study effects:** The narrower performance ranges and systematically higher metrics in small studies (N<200) compared to large studies (N>1000) suggest possible small-study effects. However, with only 5 small studies across 12 distinct conditions, this pattern may reflect clinical heterogeneity rather than bias. The presence of negative findings in larger studies (e.g., Heydon et al.: 54% specificity; Marquez et al.: 28.1% specificity) suggests that unfavorable results are published even in larger samples.

**Conclusion:** The evidence base demonstrates moderate risk of reporting bias. While core diagnostic metrics were well reported and major databases were systematically searched, the absence of grey literature, predominance of commercial systems, and evidence of small-study effects introduce moderate uncertainty about whether the evidence represents the full spectrum of AI performance in point-of-care imaging. However, the narrative synthesis approach, substantial clinical and methodological heterogeneity, and representation of both favorable and unfavorable findings across study sizes limit the potential impact of these biases on review conclusions. Unlike meta-analytic reviews where publication bias can systematically inflate pooled estimates, the descriptive synthesis approach is less susceptible to such bias, though conclusions about generalizability and consistency of findings may be affected. Readers should interpret findings recognizing these limitations, particularly when extrapolating to unpublished or non-commercial AI systems, smaller validation studies, or non-English language evidence.

*END OF SUPPLEMENTARY MATERIAL 7*
