# Supplementary material for: AI in Point-of-Care Imaging for Clinical Decision Support: Systematic Review of Diagnostic Accuracy, Task-Shifting, and Explainability
Source: JMIR AI. 2026 Apr 27;5:e80928. doi: 10.2196/80928 (PMC13119389; doi:10.2196/80928)
Supplement: Multimedia Appendix 7 — Complete extracted data for all 20 included studies. [file ai-v5-e80928-s007.docx]

**Complete Extracted Data**

Artificial Intelligence in Point-of-Care Imaging for Clinical Decision Support: Systematic Review of Diagnostic Accuracy, Task-Shifting, and Explainability

**Total Studies: 20**

**Section 1: Study Registry**

| **Study_ID** | **First_Author** | **Year** | **Country** | **Continent** | **Income_Level** | **Condition** | **Setting** | **N_Analyzed** |
| --- | --- | --- | --- | --- | --- | --- | --- | --- |
| Avgerinos_2025 | Avgerinos | 2025 | Greece | Europe | HIC | Deep vein thrombosis (DVT) | Emergency department, tertiary hospital | 53 |
| Berg_2023 | Berg | 2023 | Mexico | North America | UMIC | Breast cancer (invasive ductal carcinoma, DCIS) | Hospital | 758 masses (300 women) |
| Cao_2025 | Cao | 2025 | China | Asia | UMIC | Tuberculosis (TB) | Township health centers (primary care) | 3705 |
| Chen_2023 | Chen | 2023 | China | Asia | UMIC | Ophthalmic emergencies (18 diagnoses) | ED; Primary health care (pilot) | 364 |
| Fergus_2023 | Fergus | 2023 | United Kingdom | Europe | HIC | Pressure ulcers (Cat I-IV, DTI, Unstageable) | Domiciliary/Community (home care) | 216 images (50 patients) |
| Heydon_2021 | Heydon | 2021 | United Kingdom | Europe | HIC | Diabetic retinopathy | NHS Diabetic Eye Screening Programme | 30,405 |
| Iacob_2025 | Iacob | 2025 | Romania | Europe | HIC | Cardiac pathology (LVEF <50%, valvular, pericardial) | Primary care clinic | 1780 |
| Jaremko_2023 | Jaremko | 2023 | Canada | North America | HIC | Developmental dysplasia of the hip (DDH) | Primary care clinics (3 sites) | 306 |
| Jayaraman_2025 | Jayaraman | 2025 | India | Asia | LMIC | Tuberculosis (TB) | Mobile diagnostic units (4 vans) | 25,598 |
| Kazemzadeh_2024 | Kazemzadeh | 2024 | Zambia | Africa | LMIC | Tuberculosis (TB) | Three health facilities in Lusaka | 1827 |
| Love_2018 | Love | 2018 | Mexico | North America | UMIC | Palpable breast masses | Government hospital (resource-limited) | 32 |
| Malherbe_2025 | Malherbe | 2025 | South Africa | Africa | UMIC | Breast cancer (screening) | Primary care clinic | 203 |
| Marquez_2025 | Marquez | 2025 | Philippines | Asia | LMIC | Tuberculosis (TB) | Mixed (communities, workplaces, hospitals) | 5740 |
| Nath_2024 | Nath | 2024 | India | Asia | LMIC | Tuberculosis (TB) | 12 PHC + 1 tertiary center | 4363 |
| Nothnagel_2024 | Nothnagel | 2024 | Germany | Europe | HIC | Deep vein thrombosis (DVT) | Hospital (Berlin) | 58 |
| Papachristou_2024 | Papachristou | 2024 | Sweden | Europe | HIC | Cutaneous melanoma | 36 primary care centres | 253 lesions |
| Poli_2024 | Poli | 2024 | India | Asia | LMIC | Cervical precancer and cancer | Community screening clinics | 2052 |
| Yang_2019 | Yang | 2019 | Madagascar | Africa | LIC | Soil-transmitted helminthiases | Rural villages | 113 samples; 186 images |
| Yu_2023 | Yu | 2023 | Sudan | Africa | LIC | Malaria (P. falciparum, P. vivax) | Two primary hospitals, rural | 85; 189 |
| Zhu_2024 | Zhu | 2024 | United States | North America | HIC | Vision-threatening diseases (AMD, DR, glaucoma) | Community vision screenings | 385 eyes (195 participants) |

**Section 2: Study Design**

| **Study_ID** | **Study_Design** | **Prospective_Retro** | **Single_Multi** | **N_Sites** | **Enrollment_Period** | **Enrollment_Method** | **Sample_Size** | **Sample_Unit** |
| --- | --- | --- | --- | --- | --- | --- | --- | --- |
| Avgerinos_2025 | NR | Prospective | Single | 1 | 12 months | NR | 53 | Patients |
| Berg_2023 | Diagnostic accuracy study | Prospective | Multi | 2 | Dec 2017 - May 2021 | NR | 758 masses (300 women) | Masses/Lesions |
| Cao_2025 | Prospective cohort | Prospective | Multi | 13 | June-Dec 2020 | NR | 3705 | Patients |
| Chen_2023 | Cross-sectional | Retro (main); Prosp (pilot) | Multi | 5 | Jan-Oct 2021; Feb-Sept 2022 | NR | 364 | Patients |
| Fergus_2023 | Clinical trial | Prospective | Single | 1 | March-Dec 2021 | NR | 216 images (50 pts) | Images |
| Heydon_2021 | Diagnostic accuracy study | Prospective | Multi | 3 | Jan-April 2017 | Consecutive | 30,405 | Screening episodes |
| Iacob_2025 | Diagnostic accuracy (cross-sectional) | Prospective | Single | 1 | NR | NR | 1780 | Patients |
| Jaremko_2023 | Implementation study | NR | Multi | 3 | Feb 2021-Mar 2022 | NR | 306 (369 scans) | Patients |
| Jayaraman_2025 | NR | NR | Multi | 4 | Jan-Dec 2022 | NR | 25,598 | Patients |
| Kazemzadeh_2024 | Prospective observational | Prospective | Multi | 3 | Nov 2021-Feb 2023 | NR | 1827 | Patients |
| Love_2018 | Pilot study | NR | Single | 1 | March 2016 | NR | 32 | Masses/Patients |
| Malherbe_2025 | Prospective comparative cohort | Prospective | Single | 1 | 6 months | Convenience | 203 | Patients |
| Marquez_2025 | Retrospective cross-sectional | Retrospective | Multi | 4 regions | May 2021-Mar 2024 | NR | 5740 | Patients |
| Nath_2024 | Diagnostic accuracy study | Prospective | Multi | 13 | Jan 2018-Nov 2023 | NR | 4363 | Patients |
| Nothnagel_2024 | Feasibility study | NR | Single | 1 | 2022 (3.5 months) | Consecutive | 58 | Patients |
| Papachristou_2024 | Prospective multicentre trial | Prospective | Multi | 29 | May-Dec 2022 | NR | 253 lesions | Lesions |
| Poli_2024 | Exploratory intervention | NR | NR | NR | Dec 2021-May 2022 | Sequential | 2052 | Patients |
| Yang_2019 | Diagnostic accuracy / Pilot | Prospective | Multi | 2 | June 8-18, 2018 | Volunteer/convenience | 113 samples | Samples |
| Yu_2023 | Diagnostic accuracy (case-control) | Case-control | Multi | 2 | Oct 2020-Mar 2021 | Consecutive | 85; 189 | Patients |
| Zhu_2024 | NR | Prospective | Multi | 5 | Aug-Nov 2021 | Convenience | 385 eyes | Eyes |

**Section 3: Population**

| **Study_ID** | **Target_Condition** | **Presentation** | **Age_Mean** | **Age_Range** | **Male_%** | **N_Analyzed** | **Prevalence** |
| --- | --- | --- | --- | --- | --- | --- | --- |
| Avgerinos_2025 | Deep vein thrombosis | Symptomatic (suspected DVT) | 55.6 yrs | NR | 55% | 53 | 11.3% |
| Berg_2023 | Breast cancer | Symptomatic (palpable lump) | 50.0 yrs | 18-92 yrs | 0% | 758 masses | 7.4% lesion; 16.3% participant |
| Cao_2025 | Tuberculosis | Mixed (symptomatic + screening) | NR | ≥15 yrs | 54.84% | 3705 | 2.05% |
| Chen_2023 | Ophthalmic emergencies | Symptomatic (acute <1 month) | NR | NR | NR | 364 | Urgent 19.1%, Semi 51.0%, Non 29.9% |
| Fergus_2023 | Pressure ulcers | Routine district nurse visits | NR | NR | NR | 216 images | 100% |
| Heydon_2021 | Diabetic retinopathy | Screening (T1 and T2 diabetes) | 60-66 yrs | 12-107 yrs | 54-57% | 30,405 | ~7.3% referable DR |
| Iacob_2025 | Cardiac pathology | Mixed (high CV risk or symptoms) | 62 yrs | 40-75 yrs | 54% | 1780 | 32.9% |
| Jaremko_2023 | DDH | Screening (wellness visits) | 45 days | 3-193 days | 49% | 306 | 2.0% |
| Jayaraman_2025 | Tuberculosis | Mixed screening | 48 yrs | 14-100+ yrs | 49.98% | 25,598 | 4.02-4.10% |
| Kazemzadeh_2024 | Tuberculosis | Symptomatic (TB symptoms, contacts, HIV) | NR | ≥18 yrs | 46.7% | 1827 | 10.5% |
| Love_2018 | Palpable breast masses | Symptomatic | NR | 18-67 yrs | 0% | 32 | 6% |
| Malherbe_2025 | Breast cancer | Mixed | NR | 25-85 yrs | 0.49% | 203 | NR (no ref standard) |
| Marquez_2025 | Tuberculosis | Mixed | 50 yrs | 15-91 yrs | 55% | 5740 | 13.1% |
| Nath_2024 | Tuberculosis | Symptomatic (fever, cough >2 wks) | 43.1 yrs | ≥15 yrs | 49.6% | 4363 | 53.7% |
| Nothnagel_2024 | DVT | Symptomatic | 69.7 yrs | ≥18 yrs | 41% | 58 | ~9% |
| Papachristou_2024 | Melanoma | Symptomatic (suspicious lesions) | 54-55 yrs | 20-90 yrs | 45% | 253 lesions | 8.3% |
| Poli_2024 | Cervical precancer/cancer | Screening (opportunistic) | 39.53 yrs | 25-70 yrs | 0% | 2052 | 4.29% |
| Yang_2019 | Soil-transmitted helminthiases | NR | NR | 16+ | NR | 113 | A.l.70%; T.t.89%; Hook 19% |
| Yu_2023 | Malaria | Symptomatic (suspected) | 29.8 yrs | ≥5 yrs | 54.2% | 85; 189 | 52.6% |
| Zhu_2024 | Vision-threatening diseases | Screening (community) | 52.43 yrs | NR | 60% | 385 eyes | AMD 3.1%; DR 1.0%; Glauc 20.5% |

**Section 4: POC Setting**

| **Study_ID** | **Facility_Type** | **Geographic** | **Resource_Context** | **Healthcare_System** | **Income_Level** | **POC_Justification** | **Specialist_Avail** |
| --- | --- | --- | --- | --- | --- | --- | --- |
| Avgerinos_2025 | Hospital - ED | NR | NR | NR | HIC | NR | NR |
| Berg_2023 | Hospital | NR | NR | NR | UMIC | NR | NR |
| Cao_2025 | Township health centers | Rural | NR | NR | UMIC | NR | NR |
| Chen_2023 | Hospital ED; Primary care | NR | NR | NR | UMIC | NR | NR |
| Fergus_2023 | Domiciliary/Community | NR | NR | Public (NHS) | HIC | NR | NR |
| Heydon_2021 | Screening programme | NR | NR | Public (NHS) | HIC | NR | NR |
| Iacob_2025 | Primary care clinic | NR | NR | NR | HIC | NR | NR |
| Jaremko_2023 | Primary care clinics | Towns within 150km | NR | NR | HIC | Portable US by lightly trained providers with AI | NR |
| Jayaraman_2025 | Mobile diagnostic units | Remote, rural | Low-resource | NR | LMIC | NR | NR |
| Kazemzadeh_2024 | Health facilities | NR | High TB/HIV burden | NR | LMIC | NR | NR |
| Love_2018 | Government hospital | NR | Resource-limited | Public | UMIC | Average 9-month wait for US due to limited radiologists | Limited |
| Malherbe_2025 | Primary care clinic | Urban | NR | NR | UMIC | NR | NR |
| Marquez_2025 | Mixed (community/workplace/hospital) | NR | NR | NR | LMIC | NR | NR |
| Nath_2024 | Mixed (12 PHC + 1 TC) | 6 remote locations | Resource-limited | NR | LMIC | NR | Limited |
| Nothnagel_2024 | Hospital | NR | NR | NR | HIC | NR | NR |
| Papachristou_2024 | Primary care centres | 7 regions | NR | NR | HIC | AI benefit greatest among PCPs | NR |
| Poli_2024 | Community screening clinics | Rural | LMIC | NR | LMIC | Limited expert colposcopists | Limited |
| Yang_2019 | Community (rural villages) | Rural (14h footpath) | Low-resource | NR | LIC | Microscopy equipment days' hike away | None |
| Yu_2023 | Primary hospitals | Rural (40-50km) | NR | NR | LIC | Potential for resource-limited deployment | NR |
| Zhu_2024 | Community (screening events) | NR | Underserved | NR | HIC | 5.7 ophthalmologists per 100,000 | Limited |

**Section 5: Operator Characteristics**

| **Study_ID** | **Operator_Primary** | **Operator_All** | **N_Ops** | **Prior_Experience** | **Training** | **Training_Duration** | **Hours** | **Training_Format** | **Competency** |
| --- | --- | --- | --- | --- | --- | --- | --- | --- | --- |
| Avgerinos_2025 | Non-specialists | 1 researcher, 2 residents | 3 | None | Yes | 1 hr + 2 practice scans | 1 | App use/scanning | Yes |
| Berg_2023 | Radiologist; coordinators | 1 radiologist + 2 coordinators | 3 | Radiologist: 5 yrs; Coords: none | Yes | 30 min | 0.5 | PowerPoint | NR |
| Cao_2025 | Local radiologists | Local radiologists | NR | NR | NR | NR | NR | NR | NR |
| Chen_2023 | Patients; trained staff | Patients, staff, ophthalmologists | NR | Mixed | NR | NR | NR | NR | NR |
| Fergus_2023 | District nurses | District nurses, specialist nurses | NR | Varied | NR | NR | NR | NR | NR |
| Heydon_2021 | Trained graders | Primary, secondary, tertiary graders | NR | Trained | NR | NR | NR | NR | NR |
| Iacob_2025 | Family physicians | Family physicians (trained) | NR | Non-specialist | Yes | 6 months | NR | Didactic+supervised | Yes |
| Jaremko_2023 | Nurses and FPs | 2 RN, 4 LPN, 3 sonographers, 4 MDs | 13 | Non-specialist | Yes | 2-4 scanning days | NR | Video, PPT, demo | NR |
| Jayaraman_2025 | Expert radiologist | Expert radiologist (15 yrs) | NR | 15 yrs CXR | NR | NR | NR | NR | NR |
| Kazemzadeh_2024 | NR | NR | NR | NR | NR | NR | NR | NR | NR |
| Love_2018 | Minimally trained HCWs | Student, nurse, intern | 3 | None | Yes | ~30 min | 0.5 | PowerPoint | NR |
| Malherbe_2025 | GP with US exp | GP (US); Nurse (CBE) | NR | GP with US exp | NR | NR | NR | NR | NR |
| Marquez_2025 | Trained health workers | Trained health workers | NR | NR | NR | NR | NR | NR | NR |
| Nath_2024 | X-ray technicians | On-site technicians | NR | NR | Yes | NR | NR | NR | NR |
| Nothnagel_2024 | Non-specialist; Remote specialists | HCPs; 5 remote experts | 5 | None formal | Yes | 1 hour | 1 | ThinkSono training | NR |
| Papachristou_2024 | PCPs | 90 GPs + 48 residents | 138 | Varied | Yes | NR (on-site) | NR | On-site instruction | NR |
| Poli_2024 | Trained nurses | Nurses 3-12 yrs VIA exp | NR | 3-12 yrs VIA | NR | NR | NR | NR | NR |
| Yang_2019 | Local HCWs | Local HCWs | NR | NR | NR | NR | NR | NR | NR |
| Yu_2023 | Microscopists | Site microscopists; WHO experts | NR | NR | Yes | One short session | NR | Manual+online | NR |
| Zhu_2024 | Medical students | Students; Grader+ophthalmologist | NR | None | Yes | NR | NR | Equipment training | NR |

**Section 6: AI System**

| **Study_ID** | **AI_Name** | **AI_Developer** | **Architecture_Category** | **Architecture_Specific** | **Commercial_Status** | **Regulatory_Status** |
| --- | --- | --- | --- | --- | --- | --- |
| Avgerinos_2025 | ThinkSono Guidance | ThinkSono GmbH | NR | NR | Commercial | CE Class IIb |
| Berg_2023 | Koios DS | Koios Medical | NR | NR | Commercial | NR |
| Cao_2025 | JF CXR-1 v3.0 | JF Healthcare | Deep learning | NR | Commercial | NR |
| Chen_2023 | EE-Explorer | Zhongshan Ophthalmic Center | Deep learning | DenseNet201+InceptionV3+XGBoost | Research | NR |
| Fergus_2023 | Faster R-CNN | Liverpool John Moores Univ | Deep learning | Faster R-CNN with ResNet101 | Research | NR |
| Heydon_2021 | EyeArt v2.1.0 | Eyenuk | ML/deep learning | NR | Commercial | CE marked |
| Iacob_2025 | Wis+ AI platform | NR | Deep learning | CNN | Commercial | NR |
| Jaremko_2023 | MEDO-Hip | MEDO.ai | Deep learning | UNet-like CNN | Commercial | FDA-cleared |
| Jayaraman_2025 | Genki v1 | DeepTek Medical Imaging | Deep learning | UNET-based CNN with Xception | Commercial | NR |
| Kazemzadeh_2024 | Google TB AI | Google | NR | NR | Research | NR |
| Love_2018 | Triage-CADx system | AI Strategy | Deep learning | CNN (Inception-v3) | Research | NR |
| Malherbe_2025 | Breast AI | NR (likely MedSol.ai) | NR | NR | Commercial | SAHPRA Type A |
| Marquez_2025 | qXR version 3 | Qure.ai | NR | NR | Commercial | WHO-evaluated |
| Nath_2024 | DecXpert v1.4 | IIT Kanpur/SGPGIMS | Deep learning | Deep CNN with self-attention | Research | NR |
| Nothnagel_2024 | ThinkSono Guidance | ThinkSono GmbH | Deep learning | U-Net CNN | Commercial | CE Class 1 |
| Papachristou_2024 | Dermalyser® | AI Medical Technology | Deep learning | CNN | Commercial | NR |
| Poli_2024 | VIA-AI | PHFI / Joly AI | Deep learning | EfficientNet B3/V2 | Research | NR |
| Yang_2019 | Kankanet | Stony Brook University | Deep learning | SSD + MobileNet CNN | Research | NR |
| Yu_2023 | Malaria Screener v1.6.6 | NLM/NIH | Deep learning | NR | Research | NR |
| Zhu_2024 | SELENA+ | EyRIS Pte Ltd | Deep learning | CNN | Commercial | NR |

**Section 7: AI Output**

| **Study_ID** | **Output_Type** | **Output_Classes** | **Threshold** | **Processing_Time** | **Real_Time** | **Offline_Capable** |
| --- | --- | --- | --- | --- | --- | --- |
| Avgerinos_2025 | Guidance | NR | NR | NR | Yes | NR |
| Berg_2023 | Classification | Benign, probably benign, suspicious, malignant | NR | NR | NR | NR |
| Cao_2025 | Classification (0-1) | Normal, active TB, prior TB, pneumonia, nodule, other | 0.5 | NR | NR | NR |
| Chen_2023 | Multi-class triage; multi-label dx | Urgent, semiurgent, nonurgent; 18 labels | NR | NR | NR | NR |
| Fergus_2023 | Classification + bounding box | Cat I, II, III, IV, DTI, Unstageable | @.75 confidence | 2-3 sec | Yes | No |
| Heydon_2021 | Binary classification | Test-positive, Test-negative | NR | NR | NR | NR |
| Iacob_2025 | Binary + measurement | LVEF <50%; valvular; pericardial effusion | LVEF <50% | NR | Yes | NR |
| Jaremko_2023 | Multi-class + segmentation | Healthy, FU Recommended, Inconclusive | Graf methodology | <1 min | Yes | No |
| Jayaraman_2025 | Binary classification | TB suggestive, TB not suggestive | Default operating point | <1 min | Yes | Yes |
| Kazemzadeh_2024 | Classification (0-1) | Positive/Negative | 0.305 (high-sens), 0.465 (balanced) | <1 min | Yes | Yes |
| Love_2018 | Classification (0-1) + Binary | Suspicious (red) / Benign (green) | NR | NR | NR | NR |
| Malherbe_2025 | Risk stratification + BI-RADS | BI-RADS 2, 3, 4, 5 + risk % | NR | NR | NR | NR |
| Marquez_2025 | Classification (0-1) | TB presumptive / TB negative | 0.50 | NR | NR | NR |
| Nath_2024 | Classification (0-100) | TB positive / TB negative | 50 | <1 min | Yes | Yes |
| Nothnagel_2024 | Guidance + Segmentation | Segmentation masks + location labels | ACEP ≥3 | 25 ms | Yes | No |
| Papachristou_2024 | Classification (0-1→binary) | Melanoma detected / No melanoma | Pre-specified | NR | NR | NR |
| Poli_2024 | Multi-class | Normal, Abnormal (ablative, excision, cancer) | NR | NR | Yes | NR |
| Yang_2019 | Object detection + confidence | A. lumbricoides, T. trichiura, Hookworm | 0.60 confidence | Real-time | Yes | Yes |
| Yu_2023 | Binary + confidence | Positive/Negative (malaria) | Calibrated from ROC | 5-15 min/smear | Yes | NR |
| Zhu_2024 | Multi-class | Referable, Non-referable, Ungradable | NR | 35.6±13.3 s | Yes | No |

**Section 8: Imaging**

| **Study_ID** | **Modality_Primary** | **Modality_Subtype** | **Device_Type** | **Image_Acquisition_By** | **Protocol_Standardized** |
| --- | --- | --- | --- | --- | --- |
| Avgerinos_2025 | Ultrasound | Compression ultrasound | Handheld (Clarius HD3 L7) | Non-specialists | Yes |
| Berg_2023 | Ultrasound | Breast ultrasound | Portable / Cart-based | Radiologist and coordinators | Yes |
| Cao_2025 | X-ray | Digital chest X-ray | NR | NR | NR |
| Chen_2023 | Photography | Smartphone + slit-lamp | Portable + Standard | Patients; trained staff | NR |
| Fergus_2023 | Photography | Mobile phone photography | Mobile phones | District nurses | No |
| Heydon_2021 | Digital retinal imaging | Fundus photography | NR | NR | Yes (NHS DESP) |
| Iacob_2025 | Ultrasound | Focused Cardiac Ultrasound | Cart-based (Sonoscape P60) | Family physicians | Yes (5 views) |
| Jaremko_2023 | Ultrasound | Hip ultrasound (Graf) | Handheld (Philips Lumify) | Nurses, LPNs, physicians | Yes |
| Jayaraman_2025 | X-ray | Digital chest X-ray | Mobile (in vans) | NR | NR |
| Kazemzadeh_2024 | X-ray | Digital chest X-ray | Digital CXR machine | NR | Yes |
| Love_2018 | Ultrasound | Breast ultrasound | Portable/Cart-based | Minimally trained HCWs | Yes |
| Malherbe_2025 | Ultrasound | Breast POCUS | Handheld (Clarius) | GP with US experience | NR |
| Marquez_2025 | X-ray | Postero-anterior CXR | NR | NR | Yes |
| Nath_2024 | X-ray | Digital chest X-ray | Digital CXR | NR | Yes |
| Nothnagel_2024 | Ultrasound | POCUS (compression) | Handheld (Clarius L7 HD3) | Non-specialist | Yes |
| Papachristou_2024 | Dermoscopy | Smartphone dermoscopy | Handheld | Primary care physicians | NR |
| Poli_2024 | Colposcopy/Photography | Low-power colposcopy (VIA) | Smartphone/Mobile | Trained nurses | Yes |
| Yang_2019 | Microscopy | UVC smartphone microscopy | Mobile (smartphone) | NR | NR |
| Yu_2023 | Microscopy | Giemsa-stained blood smear | Smartphone on microscope | Microscopists | Yes (WHO) |
| Zhu_2024 | Retinal fundus photography | Non-mydriatic 45° fundus | Cart-based | Medical students | Yes |

**Section 9: Reference Standard**

| **Study_ID** | **Reference_Type** | **Reference_Details** | **Reference_Expert** | **Ref_Blinding** | **Index_Blinding** | **Verification_%** |
| --- | --- | --- | --- | --- | --- | --- |
| Avgerinos_2025 | Duplex US or D-dimer + follow-up | Full leg DUS with compression and Doppler | On-call radiologist | No | Yes | 68% |
| Berg_2023 | Differential: histopath/radiologist | Histopath for BI-RADS ≥4A; Radiologist for BI-RADS 1-3 | Specialist radiologist | Yes | Yes | NR |
| Cao_2025 | Expert panel (radiological) | Panel of 1 radiologist + 2 experts (>10 yrs) | Expert panel | No | Yes | 6.35% |
| Chen_2023 | Expert ophthalmologist consensus | Independent labeling by 2 ophthalmologists | Ophthalmologists | Yes | Yes | 100% |
| Fergus_2023 | Expert assessment (specialist nurse) | Specialist nurses reviewed AI classifications | Specialist nurses | No | Yes | 100% |
| Heydon_2021 | Human grading (NHS DESP) | Multi-tier grading (primary, secondary, arbitration) | Trained graders | Yes | Yes | 100% |
| Iacob_2025 | Cardiologist echocardiography | Comprehensive echo per ASE/EACVI guidelines | Blinded cardiologist | Yes | Yes | 100% |
| Jaremko_2023 | Differential: Orthopedic assessment | Orthopedic specialist assessment | Pediatric orthopedist | No | Yes | 2% |
| Jayaraman_2025 | Radiologist CXR interpretation | Expert radiologist classified scans | Expert radiologist | No | Yes | 100% (imperfect) |
| Kazemzadeh_2024 | Microbiological (culture/Xpert Ultra) | M. tuberculosis on culture OR Xpert Ultra | CIDRZ laboratory | Yes | Yes | 100% |
| Love_2018 | Differential: histopath/radiologist | US-guided biopsy for BI-RADS ≥4a | Radiologist | Yes | Yes | 44% |
| Malherbe_2025 | NONE - NO REF STANDARD | Compares CBE vs AI without gold standard | N/A | N/A | NR | 0% |
| Marquez_2025 | Microbiological (mWRD/GeneXpert) | Positive mWRD indicates MTB presence | Laboratory | N/A | Yes | 12% |
| Nath_2024 | Microbiological (GeneXpert) | WHO-endorsed molecular test for MTB | Laboratory | N/A | Yes | 100% |
| Nothnagel_2024 | Formal duplex scan | Compressions at multiple points + Doppler | Qualified physician | Yes | Yes | 64% |
| Papachristou_2024 | Differential: Histopath/Dermatologist | Histopath for excised; Clinical dx for benign | Pathologists/Dermatologists | Yes | Yes | 53% |
| Poli_2024 | Differential: Histopath/Colposcopy | Histopath for Swede >4; Colposcopy for others | Expert colposcopist | Yes | Yes | 8.9% |
| Yang_2019 | Composite standard microscopy | Any positive from KK, SSTT, or MIF | Trained parasitologists | Unclear | Yes | 100% |
| Yu_2023 | Expert microscopy + Nested PCR | WHO Level 1 microscopists; PCR confirmation | WHO experts; CIDRZ lab | Yes | Yes | 100% |
| Zhu_2024 | Expert fundus photo interpretation | Three-tiered grading | Tele-grader + ophthalmologists | Yes | Yes | 100% |

**Section 10: Diagnostic Performance**

| **Study_ID** | **Sensitivity** | **Sens_CI** | **Specificity** | **Spec_CI** | **AUC** | **PPV** | **NPV** | **Accuracy** |
| --- | --- | --- | --- | --- | --- | --- | --- | --- |
| Avgerinos_2025 | 100% | NR | 95.7% | NR | NR | 75% | 100% | NR |
| Berg_2023 | 95% (SOC); 95% (Portable) | 89-100% | 79% (SOC); 48% (Portable) | 76-82%; 44-53% | 0.95; 0.92 | NR | 99% | NR |
| Cao_2025 | 92.11% | 86.04%-98.17% | 94.54% | 93.81%-95.28% | NR | NR | NR | NR |
| Chen_2023 | Urg 90%, Semi 94.7%, Non 96.2% | 86.4%-93.6% | NR | NR | 0.982; 0.988 | NR | NR | 94.3% |
| Fergus_2023 | NR (Recall: 0.6997) | NR | NR | NR | 0.63-0.93 | Prec: 0.68 | NR | NR |
| Heydon_2021 | 95.7% (ref); 100% (R2,R3) | 94.8%-96.5% | 68% (R0M0); 54% (non-ref) | 67%-69% | NR | 14% | 99% | NR |
| Iacob_2025 | 89.91% | 87.18%-92.23% | 96.49% | 95.28%-97.46% | 0.94; 0.91 | 92.61% | 95.13% | 94.33% |
| Jaremko_2023 | NR | NR | 100% (ortho referrals) | NR | NR | NR | NR | NR |
| Jayaraman_2025 | 98% | 97.0%-98.8% | 96.9% | 96.6%-97.1% | NR | NR | NR | 96.9% |
| Kazemzadeh_2024 | 87% | 82-92% | 70% | 67-72% | 0.87 | NR | NR | NR |
| Love_2018 | 100% (2/2) | NR | 100% (30/30) | NR | 1.0 | NR | NR | NR |
| Malherbe_2025 | NR (no ref standard) | NR | NR | NR | NR | NR | NR | 97.6% (prior) |
| Marquez_2025 | Pseudo: 95.6% | 95.1-96.1% | Pseudo: 28.1% | 26.9-29.2% | 0.82 | 16.6% | 97.8% | NR |
| Nath_2024 | 88% | 85-93% | 85% | 82-91% | 0.85; 0.91 | 88% | 85% | NR |
| Nothnagel_2024 | 100% | 99.12-100% | 90.57% | 90.48-91.66% | NR | NR | NR | NR |
| Papachristou_2024 | 95.2% | NR | 84.5% (best); 60.3% (predef) | NR | 0.960 | 35.9%; 17.9% | 99.5%; 99.3% | NR |
| Poli_2024 | 62.50% | 51.53-72.60% | 97.56% | 96.77-98.19% | 0.76 | 53.40% | 98.31% | 76% |
| Yang_2019 | A.l.85.7%, T.t.100%, Hook 66.7% | A.l.0.63-0.77 | A.l.87.5%, T.t.100%, Hook 100% | NR | NR | A.l.85.7% | A.l.87.5% | NR |
| Yu_2023 | MS:100%; Post:92.5%; PVF:86.9% | Various | MS:51.1%; Post:91.1%; PVF:78.9% | Various | NR | NR | NR | MS:74%; Post:92%; PVF:83% |
| Zhu_2024 | All:63.2%; AMD:33.3%; DR:100%; Glauc:64.7% | Various | All:94.5%; AMD:97.9%; DR:95.9%; Glauc:90.5% | Various | NR | All:32.0% | All:98.4% | NR |

**Section 11: Comparator**

| **Study_ID** | **Comparator_Included** | **Comparator_Type** | **Comparator_Desc** | **Comp_Sens** | **Comp_Spec** | **AI_vs_Comparator** |
| --- | --- | --- | --- | --- | --- | --- |
| Avgerinos_2025 | Yes | D-dimer testing | D-dimer blood test | 100% | 36.2% | ThinkSono higher specificity (95.7% vs 36.2%) |
| Berg_2023 | Yes | Expert radiologist | Specialist radiologist (5 yrs exp) | 100% | 87% | AI AUC 0.95 vs Radiologist 0.98; P=0.10 |
| Cao_2025 | Yes | Human (local radiologists) | Local radiologists at township centers | 32.89% | 99.28% | AI higher sens (92% vs 33%, P<0.05); lower spec |
| Chen_2023 | Yes | Human (triage nurses) | Triage nurses | 58-88.7% | NR | AI 94.3% accuracy vs nurses 82.4% (P<.001) |
| Fergus_2023 | Yes | Human (specialist nurses) | Specialist nurses | NR | NR | NR (no direct statistical comparison) |
| Heydon_2021 | Yes | Human (reference standard) | Human grading per NHS DESP | N/A | N/A | AI reduces human grading workload by ~50% |
| Iacob_2025 | Yes | Human (Cardiologist) | Blinded cardiologist echo | N/A | N/A | κ=0.88 excellent agreement |
| Jaremko_2023 | Yes (external) | Historical comparison | Norwegian/UK conventional US programs | 17% FU, 3% tx | NR | Similar FU (14% vs 17%) and tx (2% vs 3%) |
| Jayaraman_2025 | Yes | Human (radiologist) | Expert radiologist (15 yrs exp) | N/A | N/A | AI evaluated against radiologist interpretation |
| Kazemzadeh_2024 | Yes | Human (radiologists) | 10 India-based radiologists (9.1±2 yrs) | 76% | 82% | AI superior sens (87% vs 76%, P<.001), inferior spec |
| Love_2018 | Yes | Human (radiologist) | Hospital radiologist (3 yrs exp) | NR | NR | "as accurate as specialist radiologists" |
| Malherbe_2025 | Yes | Human (CBE by nurse) | Registered clinical nurse CBE | NR | NR | AI identified 4 additional positive cases vs CBE |
| Marquez_2025 | Yes | Reference standard | mWRD/GeneXpert testing | N/A | N/A | AI provides screening/triage |
| Nath_2024 | Yes | Human (3 radiologists) | 3 board-certified radiologists | 71% (PPV) | 83.9% (NPV) | DecXpert 88% sens vs radiologists 71% sens |
| Nothnagel_2024 | Yes | Human (formal duplex) | Qualified physician duplex scan | N/A | N/A | AI-guided POCUS vs gold standard duplex |
| Papachristou_2024 | Yes | Human (PCPs) | 138 PCPs | PPV 23.5% | NPV 95.5% | App OR 26.55 vs PCP OR 3.35 |
| Poli_2024 | Yes | Human (colposcopist) | Expert colposcopist (Swede score) | NR | NR | AI compared against VIA, Swede, histopath |
| Yang_2019 | Yes | Human (parasitologist) | Trained parasitologist microscopy | 0.22-0.83 | 0.92-0.97 | Kankanet lower sens but high for A.l. (70%) |
| Yu_2023 | Yes | Human (WHO microscopists) | WHO Level 1 expert microscopists | N/A | N/A | 94.2% accuracy of microscopists vs PCR |
| Zhu_2024 | Yes | Human (expert graders) | 3 human graders | N/A | N/A | κ=0.754 (DLAI vs gold standard) |

**Section 12: Explainability (XAI)**

| **Study_ID** | **XAI_Mentioned** | **XAI_Terms** | **Heatmap** | **GradCAM** | **Other_XAI** | **Shown_Clinicians** | **Decision_Impact** | **XAI_Cascade_Level** |
| --- | --- | --- | --- | --- | --- | --- | --- | --- |
| Avgerinos_2025 | No | NR | No | No | None | NR | NR | 0 |
| Berg_2023 | No | NR | No | No | None | NR | NR | 0 |
| Cao_2025 | No | NR | No | No | None | NR | NR | 0 |
| Chen_2023 | Yes | Grad-CAM, SHAP | Yes | Yes | SHAP | NR | NR | 1 |
| Fergus_2023 | No | NR | No | No | None | NR | NR | 0 |
| Heydon_2021 | No | NR | No | No | None | NR | NR | 0 |
| Iacob_2025 | No | NR | No | No | None | NR | NR | 0 |
| Jaremko_2023 | No | NR | No | No | Segmentation (technical) | NR | NR | 0 |
| Jayaraman_2025 | Yes | Heatmaps, outlines | Yes | No | Attention mechanism; bounding boxes | Yes | NR | 2 |
| Kazemzadeh_2024 | No | NR | No | No | None | NR | NR | 0 |
| Love_2018 | No | NR | No | No | None | NR | NR | 0 |
| Malherbe_2025 | No | NR | No | No | None | NR | NR | 0 |
| Marquez_2025 | No | NR | No | No | None | NR | NR | 0 |
| Nath_2024 | Yes | Visualization | Yes | No | Visual attention condensers | NR | NR | 1 |
| Nothnagel_2024 | No | NR | No | No | None | NR | NR | 0 |
| Papachristou_2024 | No | NR | No | No | None | NR | NR | 0 |
| Poli_2024 | Yes | Grad-CAM | Yes (dev) | Yes | None | NR | NR | 1 |
| Yang_2019 | Yes | Bounding box, confidence | No | No | Bounding box + label + % | Yes | NR | 2 |
| Yu_2023 | No | NR | No | No | None | NR | NR | 0 |
| Zhu_2024 | No | NR | No | No | None | NR | NR | 0 |

**Section 13: Clinical Outcomes**

| **Study_ID** | **Outcomes_Beyond_Accuracy** | **Referrals_Doc** | **Time_to_Dx** | **Time_Value** | **Cost_Analysis** | **Cost_Savings** | **Workflow_Impact** | **Clinical_Impact_Level** |
| --- | --- | --- | --- | --- | --- | --- | --- | --- |
| Avgerinos_2025 | Yes | NR | Yes | Median 37 min 25 sec | NR | NR | 32% discharged without DUS | 1 |
| Berg_2023 | Yes | NR | NR | NR | NR | NR | Could triage 38%-67% without referral | 1 |
| Cao_2025 | No | Yes | NR | NR | NR | NR | Low radiologist acceptance (13.27%) | 1 |
| Chen_2023 | Yes | Yes | NR | NR | NR | NR | Hierarchical referral; 88% willing to use triage | 2 |
| Fergus_2023 | Yes | NR | Yes | 2-3 seconds | NR | NR | Photography did not impact normal practice | 1 |
| Heydon_2021 | Yes | NR | NR | NR | Yes | £0.5M per 100,000 episodes | ~50% workload reduction for graders | 1 |
| Iacob_2025 | Yes | Yes | NR | NR | Yes | ~€78,000 per 1000 patients | 40% avoided unnecessary specialist evaluation | 2 |
| Jaremko_2023 | Yes | NR | Yes | <1 min (AI) | NR | NR | FU rates decreased from 30-40% to 14% | 2 |
| Jayaraman_2025 | Yes | Yes | Yes | <1 min triaged | NR | NR | Reduces workload; reduces sputum tests | 1 |
| Kazemzadeh_2024 | No | NR | Yes | <1 min inference | NR | NR | NR (AI not used clinically) | 0 |
| Love_2018 | No | NR | NR | NR | NR | NR | NR (pilot study) | 0 |
| Malherbe_2025 | Yes | NR | Yes | 6 mo-1 yr to 1 week | NR | NR | Reduced unnecessary surgical referrals | 2 |
| Marquez_2025 | Yes | NR | NR | NR | NR | NR | mWRD tests saved at higher thresholds (42%) | 1 |
| Nath_2024 | Yes | NR | Yes | <1 min vs 2 hrs GeneXpert | Yes | $6.29 vs $17.14 per TB case | 1.23x increased TB case detection; 63% cost reduction | 1 |
| Nothnagel_2024 | Yes | NR | NR | NR | NR | NR | 53% could avoid formal USS | 1 |
| Papachristou_2024 | Yes | NR | NR | NR | NR | NR | 55.3% lesions may not have needed referral | 1 |
| Poli_2024 | Yes | NR | NR | NR | NR | NR | AI reduced FP rate of VIA by 61% | 1 |
| Yang_2019 | Yes | NR | NR | NR | Yes | $0.56 vs $1.33/person | NR | 0 |
| Yu_2023 | Yes | NR | Yes | 5-15 min/smear | Yes | $150-250 setup | Not necessarily time-saving | 1 |
| Zhu_2024 | Yes | Yes | Yes | 35.6s vs 129s (grader) | NR | NR | DLAI speed can increase screening reach | 1 |

**Section 14: Limitations**

| **Study_ID** | **Sample_Size_Limit** | **Selection_Bias_Limit** | **Single_Site_Limit** | **Generalizability_Limit** | **Ref_Standard_Limit** | **Author_Limitations** |
| --- | --- | --- | --- | --- | --- | --- |
| Avgerinos_2025 | Yes | Yes | Yes | Yes | Yes (diff verif) | Small sample, few positive cases, operator availability bias |
| Berg_2023 | Moderate (56 malig) | Yes (38% exclusions) | No | Yes (AI not trained on portable) | Yes (diff verif) | Reduced performance with portable US; BI-RADS 3 exclusions |
| Cao_2025 | Moderate (76 TB) | Yes (7.6% excluded) | No | Yes | Yes (imperfect ref) | No microbiological confirmation; low radiologist acceptance |
| Chen_2023 | No | Unclear | No | Yes (China only) | No | Limited disease spectrum; threshold optimization; single-country |
| Fergus_2023 | Yes (50 pts) | Yes (79% excluded) | Yes | Yes (UK, no Cat IV) | Yes (review bias) | Small sample; 79% images excluded; image quality variance |
| Heydon_2021 | No | No | No | Unclear | Yes (human grading) | Reference standard is human grading not clinical outcomes |
| Iacob_2025 | No | Unclear | Yes | Yes (Romania) | No | Single center, Caucasian population, borderline case limitations |
| Jaremko_2023 | Yes (6 positive) | Unclear | No | Yes (Canada) | Yes (severe) | Only 2% received gold standard; beta software; learning curve |
| Jayaraman_2025 | No | Unclear | No | Yes (Chennai) | Yes (imperfect ref) | Radiologist CXR as reference (not microbiological); review bias |
| Kazemzadeh_2024 | No | Unclear | No | Unclear (Zambia) | No | Enrollment not consecutive; grid artifacts; neither met WHO 90% sens |
| Love_2018 | Yes (32 pts, 2 cancers) | Unclear | Yes | Yes (Mexico) | Yes (diff verif) | Small sample; differential verification; transducer mismatch |
| Malherbe_2025 | No | Yes (87% excluded) | Yes | Yes (urban Gauteng) | CRITICAL: NO REF | NO REFERENCE STANDARD; massive unexplained attrition |
| Marquez_2025 | No | Yes (84.7% excluded) | No | Yes (Philippines) | Yes (severe) | Severe partial verification bias; 84.7% CAD-negatives not tested |
| Nath_2024 | No | Unclear | No | Yes (North India) | No | GeneXpert not perfect; threshold pre-specification unclear |
| Nothnagel_2024 | Yes (58, ~5 DVT+) | No | Yes | Yes (Germany) | No | 36% exclusion rate; single operator; few positive cases |
| Papachristou_2024 | Moderate (21 melanomas) | Unclear | No | Yes (Sweden; Fitz I-IV) | Yes (47% clinical only) | Differential verification; Fitzpatrick V-VI excluded; COI |
| Poli_2024 | Moderate (88 CIN1+) | Yes (32% excluded) | Yes | Yes (Telangana) | Yes (severe) | 20% image interpretation errors; only 8.9% received histopath |
| Yang_2019 | Yes (113 samples) | Yes (volunteer) | No | Yes (Madagascar) | No | Low UVC image quality; T. trichiura/hookworm thin membranes |
| Yu_2023 | Yes (40 positive) | Yes (case-control) | No | Yes (Sudan) | No | Case-control design; threshold not pre-specified; P. vivax excluded |
| Zhu_2024 | Yes (AMD 6; DR 2) | Yes (convenience) | No | Unclear | Yes (imperfect) | High ungradable rate (38.9%); low AMD/DR prevalence |

**Section 15: Task-Shifting**

| **Study_ID** | **Task_From** | **Task_To** | **AI_Role** | **Training_Required** | **Performance_Achieved** | **Task_Shift_Quote** |
| --- | --- | --- | --- | --- | --- | --- |
| Avgerinos_2025 | NR | Non-US trained providers | Enables non-experts for remote review | 1 hr + 2 practice scans | NR | AI aids non-experts in acquiring valid US images |
| Berg_2023 | Specialist radiologist | Minimally trained coordinators | Enable AI interpretation by non-specialists | 30-min PowerPoint | AUC 0.78 (min trained) vs 0.98 (rad) | AI performance reduced with untrained observer |
| Cao_2025 | NR | NR | NR | NR | NR | NR |
| Chen_2023 | Ophthalmologists | Patients (self-triage); trained staff | Remote self-triage and primary diagnosis | NR | AI 94.3% vs nurses 82.4% | Assist primary diagnosis in unspecialized facilities |
| Fergus_2023 | NR | District nurses (with AI) | Makes PU management more accessible | NR | NR | More accessible to wider group of HCPs |
| Heydon_2021 | Primary human grader | ARIAS (automated system) | Replacement of primary human grader | NR | NR | Replacement of primary grader most cost-effective |
| Iacob_2025 | Cardiologists | Family physicians (with AI) | Standardized echo measurements | 6-month curriculum (>50 exams) | 89.91% sens, 96.49% spec | Allows GPs to standardize cardiac measurements |
| Jaremko_2023 | Sonographer + radiologist | Nurses, LPNs, family physicians | AI image quality + automated classification | Video, PPT, 2-4 supervised days | FU 14%, tx 2% | Primary care staff with brief training can perform DDH screening |
| Jayaraman_2025 | NR | NR | NR | NR | NR | NR |
| Kazemzadeh_2024 | NR | NR | NR | NR | NR | NR |
| Love_2018 | Trained radiologists | Minimally trained HCWs | AI-based CADx for triage | 30 minutes (PowerPoint) | 100% sens, 100% spec | Minimally trained HCW can capture images equivalent to radiologists |
| Malherbe_2025 | NR | NR | NR | NR | NR | NR |
| Marquez_2025 | NR | NR | NR | NR | NR | NR |
| Nath_2024 | Radiologists | X-ray technicians | CAD replaces human readers | Simple 4-step process | 88% sens, 85% spec | WHO: CAD can replace human readers for TB screening |
| Nothnagel_2024 | Radiologists, US techs | HCPs without formal US training | AI guides acquisition; remote specialists dx | 1 hour training | 100% sens, 91% spec | Non-specialists can capture quality images using AI-guided POCUS |
| Papachristou_2024 | Dermatologists | Primary care physicians | AI decision support for melanoma detection | On-site instruction | 95.2% sens, 84.5% spec | AI benefit greatest among PCPs |
| Poli_2024 | Expert colposcopists | Trained nurses (mid-level) | Decision-making guidance for nurses | 3-12 yrs VIA experience | 76% accuracy | AI for decision-making without direct physician supervision |
| Yang_2019 | Trained parasitologists | Local health care workers | Training aid for STH egg identification | NR | A.l. 85.7%/87.5% | Kankanet as diagnostic training aid for HCWs |
| Yu_2023 | NR | NR | NR | One short online session | MS 74%; Post 92%; PVF 83% | NR |
| Zhu_2024 | NR | Medical students (imaging) | Computer-aided diagnosis with oversight | Equipment training | Sens 63.2%, Spec 94.5% | Automated algorithms can solve limited eye care access |

**Section 16: Integration**

| **Study_ID** | **AI_Imaging_Match** | **AI_POC_Suitability** | **AI_Hardware_Compatible** | **Integration_Model** | **Integration_Success** | **Pathway_Change** |
| --- | --- | --- | --- | --- | --- | --- |
| Avgerinos_2025 | Yes (ThinkSono + Clarius) | NR | Yes (Android) | AI-guided scan → D-dimer → Remote review → DUS if positive | NR | 32% avoided DUS |
| Berg_2023 | No (trained on SOC, not portable) | NR | NR | NR | NR | NR |
| Cao_2025 | NR | NR | NR | CXR → Local radiologist → AI → Final dx → Suspected to CDC | NR | NR |
| Chen_2023 | Yes (smartphone + slit-lamp) | NR | Yes (mobile) | Self-triage → Direction to care → Primary dx (AI) → Treatment/Referral | Yes (99% triage accuracy) | Hierarchical referral |
| Fergus_2023 | Partial (30% performance drop) | NR | Yes (iOS/Android) | Photo → 4/5G transmission → TensorFlow → WordPress (2-3 sec) | NR | Automated classification |
| Heydon_2021 | Yes | NR | NR | Study done in parallel with usual care | NR | ~50% workload reduction |
| Iacob_2025 | Yes (Wis+ + Sonoscape) | NR | NR | FOCUS by FP → AI → Cardiologist echo if indicated | NR | 32.9% detected; 40% avoided referral |
| Jaremko_2023 | Yes (MEDO-Hip + Philips Lumify) | Yes (handheld + cloud) | Yes (Samsung tablet) | Wellness visit → US → AI (<1 min) → Normal/FU/Suboptimal → Referral if persistent | Yes (FU rates 14%) | 6 DDH detected; 4/6 no risk factors |
| Jayaraman_2025 | Yes (Genki + DICOM) | Yes (offline mode) | Yes (laptop) | Registration → CXR → Genki (<1 min) → Radiologist → Sputum if TB suggestive | Yes (25,598 screened) | TB suggestive referred for confirmation |
| Kazemzadeh_2024 | Yes (single frontal CXR) | Yes (CPU/GPU/cloud) | Yes (cloud-based) | CXR → Cloud upload → AI (<1 min) → Results not used clinically | NR (technical) | NR |
| Love_2018 | Yes (DICOM compatible) | Yes (portable + Windows) | Yes (Windows 7.0+) | Trainee acquires US → CADx → Red (suspicious) / Green (benign) | NR (pilot) | NR |
| Malherbe_2025 | Yes (Breast AI + Clarius) | Yes (Android + wireless) | Yes (Android) | CBE (nurse) + US/AI (GP) → Risk stratification → Referral if indicated | NR | Reduced referral time |
| Marquez_2025 | Yes (qXR for CXR DICOM) | NR | NR | Eligibility → CXR with AI-CAD → TB presumptive → mWRD; TB neg → symptoms/rad | NR (programmatic) | NR |
| Nath_2024 | Yes (7 CXR vendors) | Yes (offline/online) | Yes (500MB RAM, Win 2000+) | CXR → DecXpert (<1 min) → Score ≥50 → GeneXpert (2 hrs); <50 → Discharge | Yes (6 remote locations) | 63% cost reduction; 2.72x cost-effective |
| Nothnagel_2024 | Yes (ThinkSono + Clarius) | Yes (smartphone + handheld) | Yes (smartphone) | AI-guided POCUS → Cloud → Remote specialist → Risk stratification | 64% included for dx | 53% could avoid formal USS |
| Papachristou_2024 | Yes (Dermalyser + smartphone) | Yes (existing telederm equip) | Yes (iPhone SE) | PCP assesses → PCP decides → App applied (not used clinically) → Histopath/derm ref | NR (AI not used) | 55.3% may not need referral |
| Poli_2024 | Yes (VIA-AI for local colposcopy) | Yes (mobile/tablet) | Yes (Android) | VIA → colposcopy → image capture → AI prediction → treatment decision | 76% accuracy | AI reduced VIA FP by 61% |
| Yang_2019 | Yes (UVC + microscope) | Yes (offline; $69.82) | Yes (Android) | Fecal sample → SSTT slide → UVC imaging → Kankanet → Bounding box output | NR | NR |
| Yu_2023 | Yes (smartphone microscopy) | Yes (affordable; Google Play) | Yes (Samsung + Olympus) | Blood smear → Microscopist selects FoV → App captures → Repeat until threshold → Results | NR (first field eval) | "potential for resource-limited areas" |
| Zhu_2024 | Yes (SELENA+ cloud) | Yes (mobile Wi-Fi) | Yes (laptop + mobile Wi-Fi) | Fundus imaging → Upload to Box → Cloud DLAI → Report (35.6s) → Human verification | Yes (48/195 referred) | Deep learning increases screening efficiency |

**Section 17: QUADAS-2 Summary**

| **Study_ID** | **D1_RoB** | **D1_App** | **D2_RoB** | **D2_App** | **D3_RoB** | **D3_App** | **D4_RoB** | **Overall_Risk** | **Key_Bias_Issues** |
| --- | --- | --- | --- | --- | --- | --- | --- | --- | --- |
| Avgerinos_2025 | U | L | L | L | H | L | H | High | Partial/differential verification; incorporation bias; same radiologist |
| Berg_2023 | H | L | L | L | H | L | H | Very High | Differential verification; 38% exclusions; radiologist inflated |
| Cao_2025 | H | L | L | L | H | H | H | Very High | Differential verification (6.35%); imperfect ref standard; 7.6% excluded |
| Chen_2023 | U | L | H | L | L | L | L | Moderate | Threshold optimization; enrollment unclear |
| Fergus_2023 | U | L | H | L | H | L | H | Very High | Review/incorporation bias; threshold optimization; 79% exclusion |
| Heydon_2021 | L | L | U | L | L | L | L | Low | Threshold pre-specification unclear; otherwise strong |
| Iacob_2025 | U | L | L | L | L | L | L | Low-Moderate | Enrollment unclear; single center; Caucasian only |
| Jaremko_2023 | U | L | L | L | H | L | H | High | Severe verification bias (2%); differential; incorporation bias |
| Jayaraman_2025 | U | L | U | L | H | H | U | High | Inadequate ref standard (radiological); review bias |
| Kazemzadeh_2024 | L | L | L | L | L | L | L | Low | None major; minor enrollment issue; well-designed |
| Love_2018 | U | L | U | L | H | U | H | High | Differential verification (44%); sample size; threshold unclear |
| Malherbe_2025 | H | L | U | L | H | H | H | Very High | CRITICAL: NO REFERENCE STANDARD; 87% excluded; convenience |
| Marquez_2025 | H | L | L | L | H | L | H | High | Severe partial verification (12%); 84.7% CAD-neg not tested; pseudo-metrics |
| Nath_2024 | U | L | U | L | L | L | U | Moderate | Enrollment unclear; threshold unclear; reporting gaps |
| Nothnagel_2024 | L | L | L | L | L | L | H | Moderate | 36% exclusion bias; only 64% in 2×2 table; few positives |
| Papachristou_2024 | U | L | L | L | H | L | H | High | Differential verification (47% clinical only); enrollment unclear; COI |
| Poli_2024 | U | L | L | L | H | L | H | High | Verification bias (8.9% histopath); differential; 32% exclusion |
| Yang_2019 | H | L | U | L | U | L | L | High | Selection bias (volunteer); threshold unclear; ref blinding unclear |
| Yu_2023 | H | L | H | L | L | L | H | High | Case-control; threshold not pre-specified; 44.7% included; P.vivax excluded |
| Zhu_2024 | H | L | U | L | U | L | U | High | Selection bias (convenience); imperfect ref; 38.9% ungradable |

**Section 18: Derived Metrics**

| **Study_ID** | **XAI_Cascade_Level** | **Clinical_Impact_Level** | **Task_Shifting_Present** | **External_Validation** | **LMIC_Study** |
| --- | --- | --- | --- | --- | --- |
| Avgerinos_2025 | 0 | 1 | Yes | No | No |
| Berg_2023 | 0 | 1 | Yes | No | No |
| Cao_2025 | 0 | 1 | No | Yes | No |
| Chen_2023 | 1 | 2 | Yes | Yes | No |
| Fergus_2023 | 0 | 1 | Yes | Yes | No |
| Heydon_2021 | 0 | 1 | Yes | Yes | No |
| Iacob_2025 | 0 | 2 | Yes | No | No |
| Jaremko_2023 | 0 | 2 | Yes | No | No |
| Jayaraman_2025 | 2 | 1 | No | Yes | Yes |
| Kazemzadeh_2024 | 0 | 0 | No | Yes | Yes |
| Love_2018 | 0 | 0 | Yes | Yes | No |
| Malherbe_2025 | 0 | 2 | No | No | No |
| Marquez_2025 | 0 | 1 | No | Yes | Yes |
| Nath_2024 | 1 | 1 | Yes | Yes | Yes |
| Nothnagel_2024 | 0 | 1 | Yes | Yes | No |
| Papachristou_2024 | 0 | 1 | Yes | Yes | No |
| Poli_2024 | 1 | 1 | Yes | Yes | Yes |
| Yang_2019 | 2 | 0 | Yes | No | Yes |
| Yu_2023 | 0 | 1 | No | Yes | Yes |
| Zhu_2024 | 0 | 1 | No | Yes | No |

*END OF COMPLETE EXTRACTED DATA*
