# Supplementary material for: AI in Point-of-Care Imaging for Clinical Decision Support: Systematic Review of Diagnostic Accuracy, Task-Shifting, and Explainability
Source: JMIR AI. 2026 Apr 27;5:e80928. doi: 10.2196/80928 (PMC13119389; doi:10.2196/80928)
Supplement: Multimedia Appendix 8 — GRADE certainty of evidence assessment. GRADE: Grading of Recommendations, Assessment, Development, and Evaluation. [file ai-v5-e80928-s008.docx]

**GRADE Evidence Profile**

Artificial Intelligence in Point-of-Care Imaging for Clinical Decision Support: Systematic Review of Diagnostic Accuracy, Task-Shifting, and Explainability

**Summary Assessment**

**Clinical Question: What is the diagnostic accuracy of AI-based clinical decision support systems using point-of-care imaging?**

Setting: Point-of-care environments including primary care facilities (30%), community/screening programs (20%), emergency departments (10%), mobile diagnostic units, and home care

Population: Patients requiring diagnostic evaluation for 12 distinct conditions (tuberculosis, breast cancer/masses, deep vein thrombosis, diabetic retinopathy, and 8 other conditions) across 15 countries

Index Tests: 18 distinct AI systems applied to 6 POC imaging modalities (ultrasound 35%, chest X-ray 25%, photography 15%, fundus photography 10%, microscopy 10%, dermoscopy 5%)

Number of Studies: 20 diagnostic accuracy studies (N≈78,296 patients)

**Overall Certainty of Evidence: ⊕◯◯◯ VERY LOW**

**Summary of Findings**

• Median sensitivity: 92.0% (IQR 85.7-98.0%, range 62.5-100%)

• Median specificity: 90.6% (IQR 70.0-95.7%, range 28.1-100%)

• Median AUC: 0.87 (range 0.63-1.00, n=10 studies reporting)

• Task-shifting demonstrated in 65% of studies with minimal training (median 1 hour)

• Zero studies measured patient health outcomes

**GRADE Summary Table**

| **Domain** | **Assessment** | **Downgrade** | **Key Findings** |
| --- | --- | --- | --- |
| Risk of Bias | Very serious concern | -2 | 70% of studies at high/very high risk; verification bias (70%), selection bias (50%), threshold optimization (30%) |
| Indirectness | Serious concern | -1 | Extreme heterogeneity: 12 conditions, 6 modalities, 18 AI systems, 15 countries; POC contexts appropriate |
| Inconsistency | Serious concern | -1 | Sensitivity 62.5-100%, specificity 28.1-100%; even within TB cluster specificity varied 28.1-96.9% |
| Imprecision | Serious concern | -1 | 30% no CIs, 15% wide CIs (>20 points), 25% small studies (N<200) |
| Publication Bias | Serious concern | -1 | 60% commercial systems, no grey literature, small-study effects; core metrics well reported (≥85%) |
| Overall Certainty | VERY LOW | -6 | True performance may differ substantially from reported estimates; local validation essential |

**Detailed Domain Assessments**

**Domain 1: Risk of Bias (Very Serious Concern, -2)**

**QUADAS-2 Overall Risk Distribution:**

| **Risk Level** | **N Studies** | **Percentage** | **Studies** |
| --- | --- | --- | --- |
| Low risk | 2 | 10% | Heydon 2021, Kazemzadeh 2024 |
| Low-Moderate risk | 1 | 5% | Iacob 2025 |
| Moderate risk | 3 | 15% | Chen 2023, Nath 2024, Nothnagel 2024 |
| High risk | 10 | 50% | Avgerinos, Jaremko, Jayaraman, Love, Marquez, Papachristou, Poli, Yang, Yu, Zhu |
| Very High risk | 4 | 20% | Berg, Cao, Fergus, Malherbe |
| Combined High/Very High | 14 | 70% | — |

**Domain-Specific Risk of Bias Breakdown:**

• Patient Selection (Domain 1): 15% low risk, 35% high risk, 50% unclear — predominantly unclear due to inadequate enrollment reporting

• Index Test (Domain 2): 50% low risk, 15% high risk, 35% unclear — automated AI reduces conduct bias; high risk from post-hoc threshold optimization

• Reference Standard (Domain 3): 35% low risk, 55% high risk, 10% unclear — worst domain; high risk from differential verification

• Flow and Timing (Domain 4): 25% low risk, 60% high risk, 15% unclear — second-worst domain; high risk from substantial exclusions

**Most Common Methodological Flaws:**

• Verification bias (14/20, 70%): Differential or partial verification where only positive/suspicious cases received reference standard

• Selection bias (10/20, 50%): Non-consecutive or convenience sampling rather than consecutive enrollment

• Threshold optimization (6/20, 30%): Post-hoc threshold selection without pre-specification

• High exclusion rates (6/20, 30%): >20% of enrolled patients excluded from analysis

**Justification:** The finding that 70% of the evidence base carries high or very high risk of bias substantially exceeds the threshold for very serious concern (>60% per GRADE criteria). These pervasive methodological weaknesses create high likelihood of bias favoring AI performance.

**Domain 2: Indirectness (Serious Concern, -1)**

**Applicability Assessment:**

• Population: Direct applicability — 15 countries across 4 continents (Asia 30%, Europe 30%, Africa 20%, North America 20%)

• Index Test: Direct applicability — Device types: Handheld (40%), mobile phone-based (30%), portable (15%), cart-based (15%)

• Setting: Direct applicability — Primary care (30%), community/screening (20%), general hospitals (15%), emergency departments (10%)

**Heterogeneity as Major Indirectness Concern:**

• 12 distinct clinical conditions

• 6 imaging modalities: Ultrasound (7 studies), chest X-ray (5), photography (3), fundus photography (2), microscopy (2), dermoscopy (1)

• 18 distinct AI systems with unique architectures

• 15 countries spanning vastly different healthcare systems

• Zero studies validated AI systems across different resource contexts

**Domain 3: Inconsistency (Serious Concern, -1)**

**Performance Summary:**

| **Metric** | **Studies Reporting** | **Range** | **Median** | **IQR** |
| --- | --- | --- | --- | --- |
| Sensitivity | 18/20 (90%) | 62.5-100% | 92.0% | 85.7-98.0% |
| Specificity | 17/20 (85%) | 28.1-100% | 90.6% | 70.0-95.7% |
| AUC | 10/20 (50%) | 0.63-1.00 | 0.87 | — |

**Within-Condition Consistency (Tuberculosis Cluster, n=5):**

• Sensitivity range: 87% to 98% (11 percentage point spread) — relatively consistent

• Specificity range: 28.1% to 96.9% (68.8 percentage point spread) — highly variable

• Variation partially explained by different clinical priorities (screening vs. diagnosis) and prevalence contexts

**Domain 4: Imprecision (Serious Concern, -1)**

**Sample Size Distribution:**

| **Size Category** | **N Studies** | **Percentage** | **Studies** |
| --- | --- | --- | --- |
| Small (N<200) | 5 | 25% | Love (32), Avgerinos (53), Nothnagel (58), Yu (85-189), Yang (113) |
| Small-Medium (200-500) | 6 | 30% | Malherbe (203), Fergus (216), Papachristou (253), Jaremko (306), Chen (364), Zhu (385) |
| Medium-Large (500-2000) | 4 | 20% | Berg (758), Iacob (1,780), Kazemzadeh (1,827), Poli (2,052) |
| Large (N>2000) | 5 | 25% | Cao (3,705), Nath (4,363), Marquez (5,740), Jayaraman (25,598), Heydon (30,405) |

**Confidence Interval Reporting:**

• Sensitivity CIs: 13/18 studies (72%) — 5 studies with no CI reported

• Specificity CIs: 12/17 studies (71%) — 5 studies with no CI reported

• Studies with CI width >20 percentage points: 15% — indicating substantial imprecision

**Domain 5: Publication Bias (Serious Concern, -1)**

**Literature Search:**

• Databases searched: 4 major databases (PubMed, Scopus, IEEE Xplore, Web of Science)

• Grey literature: NOT searched — potential for missing unpublished negative findings

• Language restrictions: English only

**Commercial vs. Research System Distribution:**

• Commercial systems: 12/20 (60%) — potential bias toward favorable results

• Research prototypes: 7/20 (35%)

• Open-source: 1/20 (5%)

**Small-Study Effects:**

• Small studies (N<200): Sensitivity 85.7-100%, specificity 87.5-100% — consistently high

• Large studies (N>1000): Sensitivity 62.5-98.0%, specificity 28.1-97.6% — more variable, including low outliers

• Pattern consistent with preferential publication of favorable small-study results

**Interpretation**

We have very low certainty in these diagnostic accuracy estimates. The true performance of AI-assisted POC imaging systems may be substantially different from the reported values. This very low certainty reflects pervasive methodological limitations (70% high/very high risk of bias), extreme heterogeneity limiting applicability, substantial inconsistency in performance estimates, incomplete precision reporting, and potential publication bias. Clinical decision-making should proceed with caution, recognizing that local validation is essential before adoption and that real-world performance may differ substantially from published estimates.

**Clinical Implications**

• Despite promising median performance, evidence is insufficient to support confident widespread adoption without local validation

• The 13 studies demonstrating task-shifting with minimal training (median 1 hour) suggest potential for democratizing diagnostic capabilities, but methodological concerns limit confidence

• Zero studies measured patient health outcomes, leaving fundamental questions about clinical impact unanswered

• Implementation should proceed cautiously through pilot programs with rigorous local validation and outcome assessment

• Future research must address methodological gaps, conduct multi-context validation, and measure patient outcomes

**Recommendations for Future Research**

1. Address Risk of Bias: Implement consecutive enrollment, ensure complete verification, pre-specify thresholds, follow STARD-AI guidelines

2. Reduce Heterogeneity: Conduct condition-specific reviews, perform head-to-head AI comparisons, standardize outcome definitions

3. Improve Consistency: Pre-specify thresholds, report performance across multiple thresholds, validate in multiple cohorts

4. Enhance Precision: Always report 95% CIs, ensure adequate sample sizes, report exact counts

5. Mitigate Publication Bias: Register studies prospectively, search grey literature, require complete outcome reporting

6. Cross-Context Validation: Multi-country validation, explicit HIC vs LMIC testing, validation across different equipment

7. Patient Outcome Measurement: Implementation studies with patient outcomes, RCTs comparing AI-assisted vs standard care

*END OF SUPPLEMENTARY MATERIAL 8: GRADE EVIDENCE PROFILE*
